# Neuropeptidergic Signaling in the American Lobster *Homarus americanus*: New Insights from High-Throughput Nucleotide Sequencing

**DOI:** 10.1371/journal.pone.0145964

**Published:** 2015-12-30

**Authors:** Andrew E. Christie, Megan Chi, Tess J. Lameyer, Micah G. Pascual, Devlin N. Shea, Meredith E. Stanhope, David J. Schulz, Patsy S. Dickinson

**Affiliations:** 1 Békésy Laboratory of Neurobiology, Pacific Biosciences Research Center and Technology, 6500 College Station, University of Hawaii at Manoa, 1993 East-West Road, Honolulu, Hawaii, 96822, United States of America; 2 Department of Biology, Bowdoin College, 6500 College Station, Brunswick, Maine, 04672, United States of America; 3 Division of Biological Sciences, University of Missouri, 218A LeFevre Hall, Columbia, Missouri, 65211, United States of America; Wake Forest University, UNITED STATES

## Abstract

Peptides are the largest and most diverse class of molecules used for neurochemical communication, playing key roles in the control of essentially all aspects of physiology and behavior. The American lobster, *Homarus americanus*, is a crustacean of commercial and biomedical importance; lobster growth and reproduction are under neuropeptidergic control, and portions of the lobster nervous system serve as models for understanding the general principles underlying rhythmic motor behavior (including peptidergic neuromodulation). While a number of neuropeptides have been identified from *H*. *americanus*, and the effects of some have been investigated at the cellular/systems levels, little is currently known about the molecular components of neuropeptidergic signaling in the lobster. Here, a *H*. *americanus* neural transcriptome was generated and mined for sequences encoding putative peptide precursors and receptors; 35 precursor- and 41 receptor-encoding transcripts were identified. We predicted 194 distinct neuropeptides from the deduced precursor proteins, including members of the adipokinetic hormone-corazonin-like peptide, allatostatin A, allatostatin C, bursicon, CCHamide, corazonin, crustacean cardioactive peptide, crustacean hyperglycemic hormone (CHH), CHH precursor-related peptide, diuretic hormone 31, diuretic hormone 44, eclosion hormone, FLRFamide, GSEFLamide, insulin-like peptide, intocin, leucokinin, myosuppressin, neuroparsin, neuropeptide F, orcokinin, pigment dispersing hormone, proctolin, pyrokinin, SIFamide, sulfakinin and tachykinin-related peptide families. While some of the predicted peptides are known *H*. *americanus* isoforms, most are novel identifications, more than doubling the extant lobster neuropeptidome. The deduced receptor proteins are the first descriptions of *H*. *americanus* neuropeptide receptors, and include ones for most of the peptide groups mentioned earlier, as well as those for ecdysis-triggering hormone, red pigment concentrating hormone and short neuropeptide F. Multiple receptors were identified for most peptide families. These data represent the most complete description of the molecular underpinnings of peptidergic signaling in *H*. *americanus*, and will serve as a foundation for future gene-based studies of neuropeptidergic control in the lobster.

## Introduction

Due to its culinary appeal, the American lobster, *Homarus americanus*, is arguably one of the world’s most iconic crustaceans. In addition to being a mainstay for the economies of New England and Atlantic Canada, this decapod is a premiere model species for studies directed at understanding the general principles governing the generation, maintenance and modulation of rhythmically active behaviors, which include walking, chewing and breathing in humans. Specifically, the numerically simple neural circuits that drive the movements of the foregut musculature (the stomatogastric neural circuit) and the neurogenic heart (the cardiac circuit) have been used since the 1960s to investigate rhythmic pattern generators [1–8]. One of the most significant findings that has come from work conducted on the stomatogastric and cardiac systems is that an almost infinite number of distinct motor patterns can be generated from a “simple,” hard-wired neural circuit via the actions of neuromodulators, the largest single class of which is peptides [1–10].

While no peptide receptors have been characterized from *H*. *americanus*, a number of neuropeptides have been identified from this species [10]. Early peptide discovery in the lobster relied on the one-by-one biochemical isolation/purification and subsequent sequencing of these molecules [11,12]. Later, molecular cloning was employed for peptide identification in this species [13]. However, the vast majority of the known *H*. *americanus* neuropeptides have been identified via mass spectrometry using accurate mass matching and/or *de novo* tandem mass spectrometric sequencing [14–28]. The power of the mass spectral approach for peptide discovery in the lobster is exemplified by a study in which 84 peptides, 57 of which were novel, were identified from neural tissues collected from *H*. *americanus* [23].

Recently, a new methodology, *in silico* genome/transcriptome mining, has been used for the discovery and characterization of peptides and peptide receptors in crustaceans [29–46]. Using this approach, large numbers of peptides can rapidly be predicted for a species. For example, 176 peptides were recently deduced from a crayfish, *Procambarus clarkii*, transcriptome [36]. Similarly, peptide receptors can be identified and characterized using *in silico* genome/transcriptome mining, *e*.*g*., the 18 peptide receptors recently predicted from the transcriptome of the copepod, *Calanus finmarchicus* [40]. Although the peptidergic signaling systems of *H*. *americanus* have not previously been investigated using large-scale *in silico* transcriptome mining, the results obtained using this methodology on other crustaceans suggest that a wealth of information could be obtained if such a study were conducted. The resulting data would provide a strong foundation for gene-based studies of peptidergic neuromodulation at the molecular level, for understanding lobster physiology more broadly, and possibly for advancing aquaculture efforts for *H*. *americanus*, for example, by increasing our understanding of the control of growth and reproduction by peptide hormones.

In the study presented here, a transcriptome *de novo* assembled from sequences derived from *H*. *americanus* neural tissues was mined for putative neuropeptide precursor- and receptor-encoding transcripts using several well-vetted *in silico* workflows [29–38,40–43,47–55]. Thirty-five putative neuropeptide precursor-encoding transcripts were identified, enabling the prediction of 194 distinct mature peptide structures. Transcripts encoding putative receptors allowed for the prediction and characterization of 41 distinct neuropeptide receptor proteins. While some of the predicted peptides are known *H*. *americanus* isoforms, most are new discoveries, at least for the lobster. The receptors identified here are the first peptide receptors described from *H*. *americanus* and are among just a handful currently known from any crustacean. Taken collectively, these data represent the most complete description of the molecular components of the peptidergic signaling systems of *H*. *americanus*, and provide the first, and thus far only, large-scale resource for initiating gene-based studies of neuropeptidergic control in the American lobster.

## Results and Discussion

### Development of a *Homarus americanus* neural transcriptome

Despite the commercial and biomedical importance of the American lobster, few genomic/transcriptomic resources have been generated for this species. In fact, prior to the present study, these data were limited to a modest collection of expressed sequence tags [56,57]. To help fill the void in the extant *H*. *americanus* molecular resources, a transcriptome was generated from RNA obtained from multiple neural tissues, which included brain, ventral nerve cord, cardiac ganglion and stomatogastric nervous system. Sequencing of this transcriptome was done using the Illumina HiSeq platform, with 452,237,240 raw reads generated from the collective set of neural libraries. *De novo* assembly using a CLC Genomics Server 5.0.1 (CLC Bio) produced 60,273 distinct contigs with an average length of 1,656 bp. This Transcriptome Shotgun Assembly (TSA) project has been deposited at DDBJ/EMBL/GenBank under the **Accession No.** **GEBG00000000** **(BioProject No.** **PRJNA300643**; **BioSample No.** **SAMN04230440**). The version described in this paper is the first version, **GEBG01000000**; it is by far the largest single collection of nucleotide sequence data for *H*. *americanus*, and being derived solely from neural tissues, it provides the first significant resource for identifying nervous system transcripts of interest in this species, including those encoding neuropeptide precursors and receptors.

### Strategy and rational for the discovery of *Homarus* transcript involved in peptidergic signaling

Many peptidergic systems are highly conserved within the Arthropoda, with isoforms of most peptide families having been identified from one or more species in three of the four subphyla (*i*.*e*., the Crustacea, Hexapoda, Chelicerata and Myriapoda) that comprise this phylum; peptide sequence data for members of the Myriapoda are extremely limited, with only one study of myriapod peptides having been completed [51]. Even with limited data from the Myriapoda, current evidence suggests that members of the adipokinetic hormone (AKH)/red pigment concentrating hormone (RPCH), adipokinetic hormone-corazonin-like peptide (ACP), allatostatin A (AST-A), allatostatin C (AST-C), allatotropin, CCHamide, crustacean cardioactive peptide (CCAP), GSEFLamide, insulin-like peptide (ILP), intocin, proctolin, pyrokinin, short neuropeptide F (sNPF), SIFamide and sulfakinin families, or closely related peptides, are present in at least some members of all four arthropod subphyla [34–36,47,51,52,58].

Given the level of conservation of neuropeptides in the Arthropoda, the strategy we used for *in silico* mining of *H*. *americanus* peptide precursor- and receptor-encoding transcripts was one based on homology to known arthropod proteins. Specifically, to identify putative peptide precursor-encoding transcripts within the *H*. *americanus* transcriptome, BLAST searches using the tblastn algorithm (search of a translated nucleotide database using a protein query) were conducted using either known *H*. *americanus* pre/preprohormone sequences (*i*.*e*. those for AST-C [19], bursicon β [38], crustacean hyperglycemic hormone (CHH) [13,59] diuretic hormone 31 (DH31) [60], myosuppressin [61], orcokinin [18], SIFamide [62], sulfakinin [63] and tachykinin-related peptide (TRP) [17]), or precursor proteins from other crustacean/hexapod species [28,30,34,36,64–71], most commonly those from the crayfish *P*. *clarkii* [36,71], a member of the same infraorder (Astacidea) as *H*. *americanus*, as the initial input queries. We had confidence that this would be an effective strategy for identifying putative pre/preprohormone-encoding transcripts, as this approach has been highly successful for the discovery of peptide precursor transcripts in a variety of other arthropods [47–55], including a diverse collection of crustacean species [29–38,40–43]. A well-established bioinformatics workflow was employed for the prediction of mature peptide structures using the pre/preprohormone sequences deduced from the identified transcripts [29–43,47–55]; the presence of isoforms of peptides from known families within the deduced precursors was used to vet our transcript identifications.

Given the paucity of neuropeptide receptor sequences known for members of the Crustacea, a slightly different strategy was used for identifying transcripts encoding these types of proteins. For these BLAST searches, insect proteins [72–77], primarily those from the fruit fly *Drosophila melanogaster* [72], were used as the tblastn input queries; for one receptor, the allatostatin B (AST-B) receptor, a crustacean protein [65] was used as the tblastn query. The use of *Drosophila* proteins as query sequences allowed for confirmation of our identifications of the deduced *H*. *americanus* proteins (and hence transcripts) via reciprocal blastp (search of a protein database using a protein query) comparison to the annotated *D*. *melanogaster* proteins curated in FlyBase [78], one of the largest, most complete, and most thoroughly characterized single-species arthropod protein databases extant. The receptor identifications were further vetted by protein BLASTs against the non-redundant arthropod protein dataset curated in GenBank, which allowed for broad species comparisons, and by structural motif analysis using the online program InterPro [79–81]. Again, this general strategy is one that has proven highly successful for identifying transcripts encoding a variety of large proteins, including neuropeptide receptors [40], from other crustaceans [82–85].

### Identification of peptide-precursor encoding transcripts and prediction of putative mature peptide structures

Nearly 40 distinct peptide families are theorized to be present in crustaceans (for a review of most crustacean peptide groups see Christie et al. [10]). Many of these families are broadly conserved across the various taxa that comprise this subphylum. For example, isoforms of AST-A, AST-C, DH31, eclosion hormone (EH), neuropeptide F (NPF), proctolin, SIFamide, sulfakinin and TRP have been identified from one or more members of the classes Remipedia [29], Branchiopoda [39,41,67], Maxillopoda [30,31,40] and Malacostraca [33,36]. However, for at least some peptide groups, there appears to be much more limited phylogenetic conservation, *e*.*g*., DENamides have thus far been identified only from the cladoceran *Daphnia pulex* [67], with members of the DXXRLamide and FXGGXamide families currently known only from copepods [30,32,35].

Using known lobster pre/preprohormone sequences, as well as precursor protein sequences from the crayfish *P*. *clarkii*, and in a few cases sequences from hexapods, as templates, 35 putative peptide-encoding transcripts were identified within the *H*. *americanus* transcriptome (Table 1 and S1 Fig). These transcripts included ones for ACP, AST-A, AST-C, bursicon α, bursicon β, CCHamide, corazonin, CCAP, CHH, DH31, diuretic hormone 44 (DH44), EH, FLRFamide, GSEFLamide, ILP, intocin, leucokinin, myosuppressin, neuroparsin, NPF, orcokinin, pigment dispersing hormone (PDH), proctolin, pyrokinin, SIFamide, sulfakinin and TRP. For most peptide families, a single pre/preprohormone-encoding transcript was identified (Table 1 and S1 Fig). However, for a few peptide groups, multiple precursor-encoding transcripts were discovered. Specifically, four CHH-encoding transcripts were found within the *H*. *americanus* transcriptome, and two transcripts were identified as encoding AST-C, CCHamide, EH, leucokinin, and pyrokinin precursor proteins (Table 1 and S1 Fig). For only AKH/RPCH, AST-B, allatotropin, DENamide, DXXRLamide, ecdysis-triggering hormone (ETH), FXGGXamide, RYamide and sNPF were no preprohormone-encoding transcripts found (Table 1). As isoforms of AKH/RPCH, AST-B and sNPF have been identified via mass spectrometry from *H*. *americanus* neural tissues [23,26], genes encoding these groups must exist; the most likely explanation for their lack of detection here is that the *H*. *americanus* transcriptome searched does not represent 100% coverage. Whether or not members of the other peptide families are present in the lobster remains an open question, but given their detection in other crustaceans [30,31,35,36,39,40], genes encoding at least allatotropin, ETH and RYamide are also predicted to exist in this species. Given their apparent limited conservation within the Crustacea [30,32,35,67], *H*. *americanus* may well not possess genes for DENamide, DXXRLamide or FXGGXamide.

**Table 1:** *Homarus americanus* peptide precursor-encoding transcripts and their deduced proteins.

Peptide family	Transcript				Deduced protein		
	Identification No.	Length*	BLAST Score	E-value	Name	Length†	Type
ACP	DS01-Homarus1_Transcript_14565	810	119.78	8.2e-28	Prepro-ACP	104	F
AST-A	DS01-Homarus1_Transcript_6078	4531	517.69	1.9e-146	Prepro-AST-A	539	F
AST-B							
AST-C	DS01-Homarus1_Transcript_3087	1071	83.19	1.2e-16	Prepro-AST-C I	85	C
	DS01-Homarus1_Transcript_39	2105	174.87	2.1e-44	Prepro-AST-C II	105	F
Allatotropin							
Bursicon α	DS01-Homarus1_Transcript_10307	1634	297.75	3.1e-81	Pre-bursicon α	140	F
Bursicon β	DS01-Homarus1_Transcript_ 28875	1463	266.54	9.3e-72	Pre-bursicon β	144	C
CCHamide	DS01-Homarus1_Transcript_ 13319	3186	154.07	3.9e-38	Prepro-CCHamide I	116	F
	DS01-Homarus1_Transcript_12464	3876	102.06	5.9e-22	Prepro-CCHamide II	252	F
Corazonin	DS01-Homarus1_Transcript_40929	1172	39.66	0.001235	Prepro-corazonin	109	F
CCAP	DS01-Homarus1_Transcript_1513	1084	254.60	3.1e-68	Prepro-CCAP	140	F
CHH	DS01-Homarus1_Transcript_5405	1921	181.80	2.2e-46	Prepro-CHH I	119	F
	DS01-Homarus1_Transcript_49975	634	83.19	1.1e-16	Prepro-CHH II	127	F
	DS01-Homarus1_Transcript_1478	4825	186.42	9.1e-48	Prepro-CHH III	100	N
	DS01-Homarus1_Transcript_4035	1379	67.01	8.0e-12	Prepro-CHH IV	34	C
DENamide							
DH31	DS01-Homarus1_Transcript_2312	2897	190.66	4.9e-49	Prepro-DH31	131	F
DH44	DS01-Homarus1_Transcript_7828	1047	42.74	0.000212	Prepro-DH44	285	F
DXXRLamide							
ETH							
EH	DS01-Homarus1_Transcript_36484	977	117.86	3.1e-27	Pre-EH I	88	F
	DS01-Homarus1_Transcript_14893	2247	59.69	1.0e-9	Pre-EH II	82	F
FLRFamide	DS01-Homarus1_Transcript_3291	3051	138.27	1.5e-32	Prepro-FLRFamide	358	F
FXGGXamide							
GSEFLamide	DS01-Homarus1_Transcript_30037	1429	57.00	8.7e-9	Prepro-GSEFLamide	35	C
ILP	DS01-Homarus1_Transcript_51972	999	41.97	0.000360	Prepro-ILP	190	F
Intocin	DS01-Homarus1_Transcript_45955	882	220.71	6.2e-58	Prepro-intocin	154	F
Leucokinin	DS01-Homarus1_Transcript_36199	1525	369.78	5.6e-102	Prepro-leucokinin I	461	N
	DS01-Homarus1_Transcript_31470	2710	59.69	1.2e-8	Prepro-leucokinin II	104	C
Myosuppressin	DS01-Homarus1_Transcript_4887	1653	169.47	9.0e-43	Prepro-myosuppressin	100	F
Neuroparsin	DS01-Homarus1_Transcript_14196	725	111.31	2.9e-25	Pre-neuroparsin	98	F
NPF	DS01-Homarus1_Transcript_7995	1467	140.58	4.4e-34	Prepro-NPF	104	F
Orcokinin	DS01-Homarus1_Transcript_1885	1113	195.28	5.7e-50	Prepro-orcokinin	91	C
PDH	DS01-Homarus1_Transcript_4536	901	91.28	3.0e-19	Prepro-PDH	79	F
Proctolin	DS01-Homarus1_Transcript_4399	832	70.09	7.4e-13	Prepro-proctolin	88	F
Pyrokinin	DS01-Homarus1_Transcript_33295	769	65.85	1.4e-11	Prepro-pyrokinin I	256	I
	DS01-Homarus1_Transcript_33296	742	48.14	0.000003	Prepro-pyrokinin II	35	C
RPCH							
RYamide							
sNPF							
SIFamide	DS01-Homarus1_Transcript_635	909	109.00	1.4e-24	Prepro-SIFamide	50	C
Sulfakinin	DS01-Homarus1_Transcript_16600	1489	196.44	6.8e-51	Prepro-sulfakinin	120	F
TRP	DS01-Homarus1_Transcript_997	2071	310.07	2.0e-84	Prepro-TRP	202	F

*Length in nucleotides.

†Length in amino acids.

Protein type: F, full-length; N, amino-terminal partial; C, carboxyl-terminal partial; I, internal fragment.

Abbreviations: ACP, adipokinetic hormone-corazonin-like peptide; AST-A, allatostatin A; AST-B, allatostatin B; AST-C, allatostatin C; CCAP, crustacean cardioactive peptide; CHH, crustacean hyperglycemic hormone; DH31, diuretic hormone 31; DH44, diuretic hormone 44; ETH, ecdysis-triggering hormone; EH, eclosion hormone; ILP, insulin-like peptide; NPF, neuropeptide F; PDH, pigment dispersing hormone; RPCH, red pigment concentrating hormone; sNPF, short neuropeptide F; TRP, tachykinin-related peptide.

Query proteins used for tblastn searches: ACP, *Procambarus clarkii* prepro-ACP (deduced from **GBEV01011002** [36]); AST-A, *P*. *clarkii* prepro-AST-A (**BAE45266** [71]); AST-B, *P*. *clarkii* prepro-allatostatin B (deduced from **GBEV01040422** [36]); AST-C, *Litopenaeus vannamei* prepro-AST-C (deduced from **FE182974** [28]) and *H*. *americanus* prepro-AST-C-like peptide (deduced from **EY291152** [19]); *Tigriopus californicus* prepro-allatotropin (deduced from **JW513825**; [30]); bursicon α, *Homarus gammarus* pre-bursicon α (**ADI86242** [70]); bursicon β, *H*. *americanus* pre-bursicon β (deduced from **CN854188** [38]); CCHamide, *P*. *clarkii* prepro-CCHamide I (deduced from **GBEV01004199** [36]) and *P*. *clarkii* prepro-CCHamide II (deduced from **GBEV01015793** [36]); corazonin, *Daphnia pulex* prepro-corazonin (**EFX86608** [65]); CCAP, *H*. *gammarus* prepro-CCAP (**ABB46292** [64]); CHH, *H*. *americanus* prepro-CHH A (**P19806** [59]); DH31, *H*. *americanus* prepro-calcitonin-like diuretic hormone (**ACX46386** [60]); DH44, *Bombyx mori* prepro-DH44 (**BAG50375** [69]); DXXRLamide, *T*. *californicus* prepro-DXXRLamide Ia (deduced from **JW528324** [30]); ETH, *D*. *pulex* prepro-ETH [67]; EH, *P*. *clarkii* pre-EH (deduced from **GBEV01101142** [36]); FLRFamide, *P*. *clarkii* prepro-FLRFamide A (**BAE06262** [71]); FXGGXamide, *T*. *californicus* prepro-FXGGXamide Ia (deduced from **JV193177** [30]); GSEFLamide, *P*. *clarkii* prepro-GSEFLamide (deduced from **GBEV01013249** [36]); ILP, *D*. *pulex* prepro-ILP 2 (**EFX70023** [65]); intocin, *Pontastacus leptodactylus* prepro-intocin (deduced from **GAFS01000331** [36]); leucokinin, *P*. *clarkii* prepro-leucokinin (deduced from the combination of **GBEV01013648** and **GBEV01006537** [36]); myosuppressin, *H*. *americanus* prepro-myosuppressin (**ACX46385** [61]); neuroparsin, *P*. *clarkii* pre-neuroparsin II (deduced from **GBEV01000414** [36]); NPF, *P*. *clarkii* prepro-NPF I (deduced from **GBEV01005906** [36]); orcokinin, *H*. *americanus* prepro-orcokinin I (**ACB41787** [18]); PDH, *Orconectes limosus* prepro-PDH (**AAB26385** [66]); proctolin, *Penaeus monodon* prepro-proctolin (deduced from **JR22035** [34]); pyrokinin, *P*. *clarkii* prepro-pyrokinin (deduced from **GBEV01049409** [36]); RPCH, *Cherax quadricarinatus* prepro-RPCH (**AAV80404** [68]); RYamide, *P*. *clarkii* prepro-RYamide (deduced from **GBEV01010112** [36]); sNPF, *P*. *clarkii* prepro-sNPF (deduced from **GBEV01004780** [36]); SIFamide, *H*. *americanus* prepro-Val^1^-SIFamide (**ABV21807** [62]); sulfakinin, *H*. *americanus* prepro-sulfakinin (**ABQ95346** [63]); TRP, *H*. *americanus* prepro-TRP (**ACB41786** [17]).

**Fig 1:**
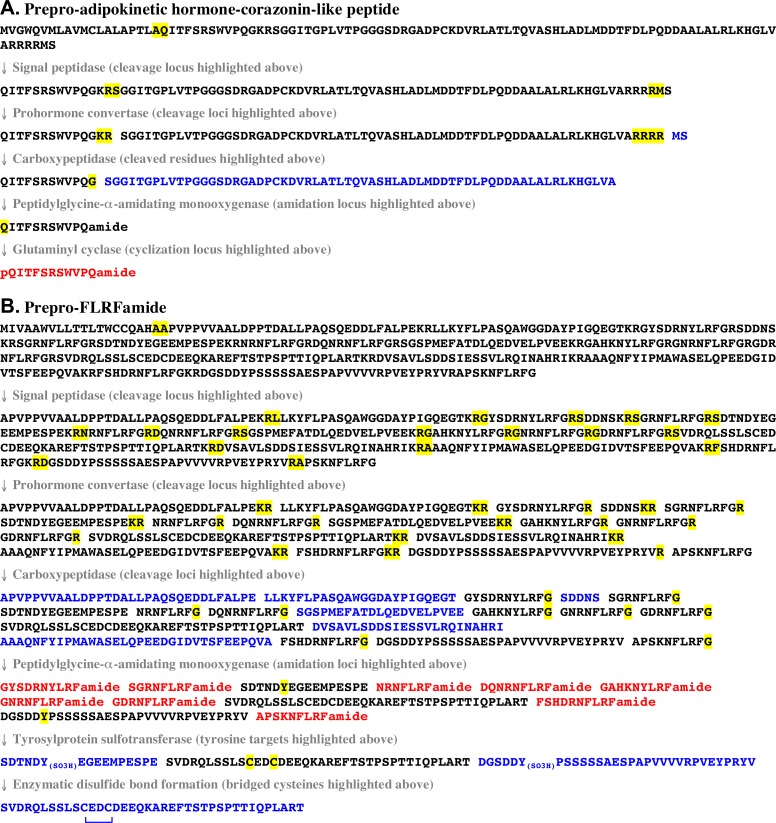
Two examples of the *in silico* workflow used for the prediction of putative mature *Homarus americanus* peptide structures. (A) Predicted processing scheme for prepro-adipokinetic hormone-corazonin-like peptide (ACP). The structure of the mature ACP isoform is shown in red, with the structures of two mature linker/precursor-related peptides shown in blue. In this schematic, the presence of a pyroglutamic acid in the putative mature ACP isoform is indicated by “pQ”. (B) Predicted processing scheme for prepro-FLRFamide. In this schematic, the structures of nine mature FLRFamide-like peptides are shown in red, with those of nine mature linker/precursor-related peptides shown in blue. Sulfated tyrosine residues in two of the linker/precursor-related sequences are indicated by “Y_(SO3H)_”. The presence of a disulfide bond between the cysteine residues in another of the linker/precursor-related peptides is indicated by an inverted blue bracket.

The identification of the transcripts just described allowed for the prediction of a new neuropeptidome for *H*. *americanus*. Here, each transcript was translated (Fig 1 and S2 Fig), and the deduced protein was subjected to a well-vetted peptide prediction workflow (Fig 1). First, each deduced pre/preprohormone was assessed for its completeness, *i*.*e*., was it a full-length protein, an amino (N)-terminal partial protein, a carboxyl (C)-terminal partial protein or an internal fragment of a protein. Full-length proteins were defined by having stop codons bracketing the open reading frame (ORF) in their transcript and possessing a functional signal peptide; putative full-length proteins did not have a stop codon located before the theorized “start” methionine, but a signal sequence was predicted starting with this residue. N-terminal partial proteins possessed no stop codon at the end of their ORFs, while C-terminal partial proteins lacked a stop codon before the ORF and did not display a “start” methionine (that produced a signal peptide). Internal protein fragments possessed no stop codon prior to their ORF and there was no evident start methionine; additionally, they lacked a stop codon at the end of their transcript’s putative coding sequence. The completeness of all deduced proteins, as well as their lengths and the lengths of the transcripts that encode them, is provided in Table 1.

Regardless of completeness, prediction of the structures (amino acid sequence and predicted post-translational modifications) of the mature peptides likely liberated from each precursor protein was accomplished via a bioinformatics workflow that used both freeware programs and homology to known arthropod pre/preprohormone processing schemes. Specifically, this workflow involved signal peptide prediction, prohormone convertase cleavage site identification, and prediction of post-translational modifications (*i*.*e*., C-terminal amidation, N-terminal cyclization of glutamine or glutamic acid to pyroglutamic acid, sulfation of tyrosine residues, and disulfide bond formation between cysteines). Fig 1 shows the predicted processing scheme for two of the *H*. *americanus* preprohormones (prepro-ACP and prepro-FLRFamide). Using this workflow, the structures of 194 distinct *H*. *americanus* peptides were predicted; these neuropeptides include one ACP, 23 AST-As, two AST-Cs, one bursicon α, one bursicon β, two CCHamides, one corazonin, one CCAP, four CHHs (two full-length and two partial), three isoforms of CHH precursor-related peptides (CPRP), one DH31, one DH44, two EHs, nine FLRFamides, two GSEFLamides, two ILPs (one A- and one B-chain peptide), one intocin, 13 leucokinins (12 full-length and one partial), one myosuppressin, one neuroparsin, one NPF, three orcokinins, one PDH, one proctolin, seven pyrokinins, one SIFamide (a partial peptide), two sulfakinins and one TRP (Table 2), as well as a large number of linker/precursor-related peptides whose structures do not place them into a formally recognized peptide family (S1 Table).

**Table 2:** *Homarus americanus* isoforms of neuropeptides from commonly recognized peptide families.

Family	Structure
ACP	**pQITFSRSWVPQa**
AST-A	**HSNYGFGLa**
	**TPGYAFGLa**
	**SDLYSFGLa**
	**SGSYNFGLa**
	**SKLYGFGLa**
	**PRNYAFGLa**
	**SQMYSFGLa**
	**PRDYAFGLa**
	**PTAYSFGLa**
	**ATSYGFGLa**
	**AGRYAFGLa**
	**TGPYAFGLa**
	**AGHYAFGLa**
	**ADPYAFGLa**
	**AGQYSFGLa**
	**SGVYSFGLa**
	**AGPYSFGLa**
	**AKYSFGIa**
	**SYDFGLa**
	*VGPYAFGLa*
	*AGPYAFGLa*
	*SGPYAFGLa*
	*SGPYSFGLa*
	AGGAYSFGLa
	ASPYAFGLa
	TPSYAFGLa
	EPYAFGLa
	SPYAFGLa
	SQYTFGLa
AST-B	STNWSSLRSAWa
	TNWNKFQGSWa
AST-C	*pQIRYHQ* *C* *YFNPIS* *C* *F*
	*SYWKQ* *C* *AFNAVS* *C* *Fa*
Bursicon α	**1**
Bursicon β	*2*
CCHamide	**HRVLKGG** **C** **LNYGHS** **C** **LGAHa**
	**S** **C** **SQFGHS** **C** **FGAHa**
Corazonin	*pQTFQYSRGWTNa*
CCAP	*PF* *C* *NAFTG* *C* *a*
CHH*	**AVFDSA** **C** **KGYYDREFWGKLSRV** **C** **WD** **C** **ENLFRQPGYQDK** **C** **SEG** **C** **FVTTDFTQ** **C** **VKALLLNVEEYNELAELVRa**
	**pQVFDQA** **C** **KGVYDRNLFKKLNRV** **C** **ED** **C** **YNLYRKPFIVTT** **C** **RQN** **C** **FEGDTFPR** **C** **VMDLGLDLELFLEFRDMIKa**
	**QVFDQACKGVYDRNLFKKLDRVCEDCYNLYRKPFVATTCR+**
	**+ENCYSNRVFRQCLDDLLMIDVIDEYVSNVQMVa**
	ASAWFTNDECPGVMGNRDLYEKVAWVCNDCANIFRNNDVGVMCKKDCFHTMDFLWCVYATERHGEIDQFRKWVSILRAa
	pQVFDQACKGVYDRNLFKKLDRVCEDCYNLYRKPFVATTCRENCYSNWVFRQCLDDLLLSDVIDEYVSNVQMVa
	pQVFDQACKGVYDRNLFKKLNRVCEDCYNLYRKPFIVTTCRENCYSNRVFRQCLDDLLLSDVIDEYVSNVQMVa
	pEVFDQACKGVYDRNLFKKLDRVCEDCYNLYRKPFVATTCRENCYSNWVFRQCLDDLLLSNVIDEYVSNVQM
CPRP	**RSVEGVSRMEKLLSSSNSPSSTPLGFLSQDHSVN**
	**RSWLIDGDEDLQLSQYHSLN**
	*RSVEGVSRMEKLLSSISPSSTPLGFLSQDHSVN*
	RSVEGASRMEKLLSSSNSPSSTPLGFLSQDHSVN
	RSVEGVSRMEKLLSSSISPSSTPLGFLSQDHSVN
	RSVEGVSRMEKLLSSISPSSMPLGFLSQDHSVN
DH31	*GLDLGLGRGFSGSQAAKHLMGLAAANFAGGPa*
DH44	**ASGLSLSIDASMKVLREALYMEIIRKKQRQQMQRAQHNQKLLNSIa**
EH	**AANKVSV** **C** **IKN** **C** **AQ** **C** **KIMYHDHFKGGL** **C** **ADL** **C** **VQSGGKFIPD** **C** **GRPQTLIPFFLQRLE**
	**ATFTSM** **C** **IRN** **C** **GQ** **C** **KEMYGDYFHGQA** **C** **AES** **C** **IMTQGISIPD** **C** **NNPATFNRFLKRFI**
FLRFamide	**FSHDRNFLRFa**
	**APSKNFLRFa**
	**NRNFLRFa**
	*GYSDRNYLRFa*
	*DQNRNFLRFa*
	*GAHKNYLRFa*
	*SGRNFLRFa*
	*GNRNFLRFa*
	*GDRNFLRFa*
	APQRNFLRFa
	GGRNFLRFa
	SDRNYLRFa
	SDRNFLRFa
	TNRNFLRFa
	NFLRFa
GSEFLamide	**AMGSEFLa**
	**AVGSEFLa**
ILP	**LCGWRLANKLNLVCKGVYNNPGSTGNYLFYRS**
	**GLSAE** **C** **CRKV** **C** **TVSELVGYCY**
Intocin	**C** **FITN** **C** **PPGa**
Leucokinin	**pQAFHPWGa**
	**ASFNPWGa**
	**NTFAPWGa**
	**pESFSAWGa**
	**TRFSAWAa**
	**TRFSPWAa**
	**PSFSAWAa**
	**pQGFSAWAa**
	**VPFSTWGa**
	**AFSAWAa**
	**TFSAWAa**
	**TFRAWAa**
	**PSFNAW+**
Myosuppressin	*pQDLDHVFLRFa*
	QDLDHVFLRFa
Neuroparsin	**APR** **C** **NQGGNRLPANN** **C** **KYGTVVDW** **C** **GGSV** **C** **AKGPGEA** **C** **GGEWSENGE** **C** **GAGTY** **C** **S** **C** **GY** **C** **NG** **C** **SANLE** **C** **WFGSY** **C**
NPF	**ARPDNSAADTLQAIHEAAMAGILGSAEVQYPNRPSMFKSPVELRQYLDALNAYYAIAGRPRFa**
Orcokinin	**+GFN**
	*NFDEIDRSGFGFN*
	*NFDEIDRSGFGFH*
	*NFDEIDRSGFGFV*
	NFDEIDRSSFGFN
	NFDEIDRSGFGFA
	NFDEIDRSGFGF
	NFDEIDRSGFA
	NFDEIDRSGFG
	NFDEIDRSGFa
	NFDEIDRSGF
Orcomyotropin	FDAFTTGFGHS
	FDAFTTGFGHN
PDH	*NSELINSILGLPKVMNDAa*
Proctolin	*RYLPT*
Pyrokinin	**GDDITNEELAY**_**(SO3H)**_ **DDNLATSEYLRDDNNDYLPEELTEDVTEMSSPEMLSESAAALVGKNSVSFIPRLa**
	**DSEDSSVESRNTKTQASIPRPa**
	**GDGFAFSPRLa**
	**GADFAFSPRLa**
	**SDFAFSPRLa**
	**SLFSPRLa**
	**AYFSPRLa**
	FSPRLa
RPCH	pQLNFSPGWa
sNPF	DTSTPALRLRFa
	GPPSLRLRFa
	SMPSLRLRFa
	FEPSLRLRFa
	PSLRLRFa
SIFamide	**+KPPFNGSIFa**
	VYRKPPFNGSIFa
	RKPPFNGSIFa
	PPFNGSIFa
Sulfakinin	*GGGEY*_*(SO3H)*_ *DDY*_*(SO3H)*_ *GHLRFa*
	*pEFDEY*_*(SO3H)*_ *GHMRFa*
TRP	*APSGFLGMRa*
	APSGFLGMRG
	TPSGFLGMRa
	PSGFLGMRa
	SGFLGMRa

^**1**^ **DECSLTPVIHILSYPGCVSKPIPSFACQGRCTSYVQVSGSKLWQTERSCMCCQESGEREASVVLNCPKVRKGEPTRRKILTRAPIDCMCRPCTDV-EEGTVLAQEIANFIHDSPMGNVPFLK**

^**2**^ *RRYDLECETLPSTIHVAKEEFDEAGRVERTCEEDLAVNKCEGACVSKVQPSVNTPSGFLKDCRCCRETHLRAREVTLTHCYDADGNRLTGDRGTL-VIKLREPADCQCFKCGDSTR*

*The peptides listed include members of both the Type I (true CHH) and Type II (molt-inhibiting hormone/gonad-inhibiting hormone) subfamilies See Christie et al. [10] for the defining characters of these subgroups.

Peptides shown in bold font are novel discoveries for H. americanus, while those shown in italic font are known H. americanus peptides that were rediscovered in this study; peptides shown in normal font are those known from H. americanus but not rediscovered here [11–14,16–20,22,23,26–28,38,59,60,63,87,88]. Peptides have been grouped based on discovery date (novel, rediscovered, known but not rediscovered) and ordered from largest to smallest within each group.

Abbreviations: ACP, adipokinetic-corazonin-like peptide; AST-A, allatostatin A; AST-B, allatostatin B; AST-C, allatostatin C; CCAP, crustacean cardioactive peptide; CHH, crustacean hyperglycemic hormone; CPRP, crustacean hyperglycemic hormone precursor-related peptide; DH31, diuretic hormone 31; DH44, diuretic hormone 44; EH, eclosion hormone; ILP, insulin-like peptide; NPF, neuropeptide F; PDH, pigment dispersing hormone; RPCH, red pigment concentrating hormone; sNPF, short neuropeptide F; TRP, tachykinin-related peptide.

In the peptide structures shown, “pQ/pE” represents an amino (N)-terminal pyroglutamic acid, “a” represents a carboxyl (C)-terminal amide group, “Y(SO3H)” represents a sulfated tyrosine residue, and “C” represents a cysteine residue involved in a disulfide bond. A “+” at the N- and/or C-terminus of a sequence indicates that it is a partial peptide.

The sulfation state of tyrosine residues and disulfide bridging between cysteine residues was predicted only for putative full-length peptides.

Disulfide bonding patterns in peptides with more than one disulfide bridge: the first and ninth, second and seventh, third and fifth, fourth and tenth, and eighth and eleventh cysteines are predicted to be bonded in bursicon α; the first and third, fourth and tenth, fifth and eleventh, sixth and eighth, and seventh and ninth cysteines are predicted to be bonded in bursicon β; the first and fifth, second and fourth, and third and sixth cysteines are predicted to be bonded in each of the full-length CHHs; the first and second, third and fourth, and fifth and sixth cysteines are predicted to be bonded in each of the EH isoforms; in ILP, the first and third cysteines are predicted to be bridged in the A-chain peptide (GLSAECCRKVCTVSELVGYCY) with bonds also predicted between it and the B-chain ILP (LCGWRLANKLNLVCKGVYNNPGSTGNYLFYRS); specifically, the first cysteine in the B-chain is predicted to be bridged to the second cysteine in the A-chain, and the second cysteine in the B-chain bridged to the fourth cysteine in the A-chain (the interpeptide bridges are not shown above); the first and seventh, second and third, fourth and tenth, fifth and eleventh, sixth and eighth, and ninth and twelfth cysteines are predicted to be bonded in the isoform of neuroparsin.

### Expansion of the neuropeptidome of *Homarus americanus* and identification of new peptide families in the American lobster

The 194 peptides identified here include both known *H*. *americanus* neuropeptides and ones described here for the first time (Table 2 and S1 Table). For example, four of the 23 predicted isoforms of AST-A (*i*.*e*., VGPYAFGLamide, AGPYAFGLamide, SGPYAFGLamide and SGPYSFGLamide) are peptides previously discovered using mass spectrometry [23]. Similarly, six of the nine FLRFamides predicted here (GYSDRNYLRFamide, SGRNFLRFamide, DQNRNFLRFamide, GAHKNYLRFamide, GNRNFLRFamide, GDRNFLRFamide) were identified in earlier mass spectral analyses [23]. Among the new discoveries for *H*. *americanus* are peptides previously described from other species, but unknown from the lobster, for example AMGSEFLamide and AVGSEFLamide, which were previously identified from several other decapods [30,36], and novel isoforms from known lobster peptide families [23], for example, the suite of pyrokinins described here. Of particular note were our identifications of isoforms of ACP, bursicon α, CCHamide, DH44, EH, GSEFLamide, ILP, intocin, leucokinin, neuroparsin and neuropeptide F, all peptides from families previously unknown in *H*. *americanus*, and in the case of DH44, from any decapod species.

While the peptidome predicted here for *H*. *americanus* is by far the largest collection of neuropeptides described for this species in any single study, some peptides previously identified from the lobster were not rediscovered in our investigation. For example, prior mass spectral analyses identified isoforms of AST-B, RPCH and sNPF in *H*. *americanus* [23], but no transcripts encoding members of these families were detected within the transcriptome mined here, even though it included RNA from tissues in which these peptides have been detected via mass spectrometry. Moreover, even for families for which transcripts were identified, they did not, in all cases, contain all of the known isoforms of the family in question, *e*.*g*., a number of the AST-As identified via mass spectrometry [23], *e*.*g*., EPYAFGLamide, TPSYAFGLamide and SQYTFGLamide, were not present within the preprohormone we discovered. Why a subset of the known *H*. *americanus* peptides was not re-identified remains an open question. As stated earlier, the transcriptome we used clearly does not have 100% coverage, and thus it is likely that for at least AST-B, RPCH and sNPF, transcripts encoding members of these families exist, but are simply not included in the assembly mined here. Moreover, not all portions of the nervous system were included in the set used for mRNA collection (*e*.*g*., the eyestalk ganglia), and thus it is possible that some peptide groups were missed for this reason. Similarly, it is possible that in the lobster some “neuropeptides” are produced primarily by non-neuronal tissues, and thus not discovered here. For peptide families in which transcripts encoding full-length precursors were identified, but previously identified peptides were not found, two possibilities exist. First, it is possible that multiple transcripts encoding members of the peptide family are present in the lobster, but those containing the peptides in question are simply not in the transcriptome we mined. Alternatively, the missing isoforms may be individual- or population-specific variants [86], and lobsters possessing these alleles were not among those used for RNA isolation. Regardless, by combining the peptides identified in our study (both reidentified and novel) with those previously known, but not rediscovered here [11–14,16–20,22,23,26–28,38,59,60,63,87,88], a peptidome of over 250 sequences can be produced for American lobster (Table 2 and S1 Table). This peptidome consists of members of 32 distinct peptide families (Table 2), as well as linker/precursor-related and other peptides whose structures do not place them into any of the generally recognized groups (S1 Table). To the best of our knowledge, this composite *H*. *americanus* peptidome is the largest thus far generated for any crustacean, and is one of, if not the largest currently known for any member of the Arthropoda.

### Identification of neuropeptide-receptor encoding transcripts

While a number of neuropeptides had been identified from *H*. *americanus* prior to our study [23], nothing was known about the identity of any lobster peptide receptors. In fact, with the exception of those from *C*. *finmarchicus* [40], a copepod, little information was available concerning the identity and diversity of neuropeptide receptors in any crustacean. Using known arthropod receptors, primarily those from the fruit fly *D*. *melanogaster*, as tblastn input queries, the *H*. *americanus* neural transcriptome was screened for transcripts encoding putative homologous proteins (Table 3). Via these searches, 41 putative receptor-encoding transcripts were identified (S3 Fig), including ones showing homology to known ACP, AST-A, AST-C, bursicon, CCHamide, corazonin, CCAP, DH31, DH44, ETH, FLRFamide, ILP, leucokinin, myosuppressin, NPF, PDH, proctolin, pyrokinin, RPCH, sNPF, SIFamide, sulfakinin and TRP receptors. It should be noted that searches for DENamide, DXXRLamide, EH, FXGGXamide and GSEFLamide receptors were not conducted (Table 3), as, to the best of our knowledge, no receptor proteins for these peptide families are known.

**Table 3:** *Homarus americanus* peptide receptor-encoding transcripts and their deduced protein.

Receptor family	Transcript				Deduced protein		
	Identification No.	Length*	BLAST Score	E-value	Name	Length†	Type
ACP	DS01-Homarus1_Transcript_58353	814	224.56	2.6e-58	ACPR	271	I
AST-A	DS01-Homarus1_Transcript_30123	4465	292.74	6.4e-79	AST-AR	467	F
AST-B							
AST-C	DS01-Homarus1_Transcript_10681	3462	324.71	2.0e-88	AST-CR I	420	F
	DS01-Homarus1_Transcript_10638	2367	320.47	3.7e-87	AST-CR II	434	F
	DS01-Homarus1_Transcript_26036	927	202.99	8.6e-52	AST-CR III	265	C
Allatotropin							
Bursicon	DS01-Homarus1_Transcript_10555	4022	600.51	4.9e-171	BursiconR I	845	C
	DS01-Homarus1_Transcript_16714	4815	589.73	8.6e-168	BursiconR II	714	C
CCHamide	DS01-Homarus1_Transcript_24860	2976	350.52	3.5e-96	CCHamideR I	461	F
	DS01-Homarus1_Transcript_37689	4036	313.92	3.6e-85	CCHamideR II	442	F
Corazonin	DS01-Homarus1_Transcript_48447	1640	242.28	1.6e-63	CorazoninR	423	C
CCAP	DS01-Homarus1_Transcript_31037	2050	270.01	5.9e-72	CCAPR	447	N
CHH							
DH31	DS01-Homarus1_Transcript_22256	5371	219.16	1.0e-56	DH31R I	870	F
	DS01-Homarus1_Transcript_5552	3520	349.75	5.1e-96	DH31R II	412	F
	DS01-Homarus1_Transcript_26723	1731	299.29	7.9e-81	DH31R III	377	C
DH44	DS01-Homarus1_Transcript_11147	4328	331.26	2.2e-90	DH44R I	533	F
	DS01-Homarus1_Transcript_14267	2496	163.70	6.1e-40	DH44R II	588	N
ETH	DS01-Homarus1_Transcript_30219	3217	249.60	7.8e-66	ETHR I	475	F
	DS01-Homarus1_Transcript_6537	3346	226.48	7.1e-59	ETHR II	479	F
	DS01-Homarus1_Transcript_54675	777	152.52	1.3e-36	ETHR III	258	I
FLRFamide	DS01-Homarus1_Transcript_7458	4789	277.33	4.2e-74	FLRFamideR	461	F
ILP	DS01-Homarus1_Transcript_11179	5948	196.82	3.3e-49	ILPR I	1521	F
	DS01-Homarus1_Transcript_3743	4812	169.09	7.3e-41	ILPR II	1233	C
Intocin							
Leucokinin	DS01-Homarus1_Transcript_14169	4167	350.90	3.0e-96	LeucokininR	479	F
Myosuppressin	DS01-Homarus1_Transcript_42439	3549	170.24	6.1e-42	MyosuppressinR	419	F
NPF	DS01-Homarus1_Transcript_40414	2096	317.39	3.2e-86	NPFR I	452	F
	DS01-Homarus1_Transcript_17633	4722	311.61	1.7e-84	NPFR II	461	F
	DS01-Homarus1_Transcript_9534	3338	175.25	1.9e-43	NPFR III	458	F
	DS01-Homarus1_Transcript_16169	3828	110.15	7.7e-24	NPFR IV	466	F
PDH	DS01-Homarus1_Transcript_14445	4180	410.99	3.1e-114	PDHR I	454	F
	DS01-Homarus1_Transcript_11293	4055	380.56	4.5e-105	PDHR II	448	F
Proctolin	DS01-Homarus1_Transcript_26916	2961	145.98	1.5e-34	ProctolinR I	664	F
	DS01-Homarus1_Transcript_45976	3186	142.51	1.6e-33	ProctolinR II	414	C
Pyrokinin	DS01-Homarus1_Transcript_40714	2384	134.42	3.3e-31	PyrokininR	658	C
RPCH	DS01-Homarus1_Transcript_57704	935	285.42	1.2e-76	RPCHR I	311	I
	DS01-Homarus1_Transcript_51875	642	150.98	3.5e-36	RPCHR II	213	I
RYamide							
sNPF	DS01-Homarus1_Transcript_35046	3750	191.82	2.6e-48	sNPFR	463	F
SIFamide	DS01-Homarus1_Transcript_25154	2712	401.36	2.8e-111	SIFamideR	568	F
Sulfakinin	DS01-Homarus1_Transcript_21140	3993	203.37	8.4e-52	SulfakininR	1225	F
TRP	DS01-Homarus1_Transcript_51762	2498	334.34	2.6e-91	TRPR I	502	F
	DS01-Homarus1_Transcript_ 23908	1536	409.45	6.4e-114	TRPR II	491	N
	DS01-Homarus1_Transcript_ 26508	2363	333.18	5.8e-91	TRPR III	408	N

*Length in nucleotides.

†Length in amino acids.

Protein type: F, full-length; N, amino-terminal partial; C, carboxyl-terminal partial; I, internal fragment.

Abbreviations: ACP, adipokinetic hormone-corazonin-like peptide; AST-A, allatostatin A; AST-B, allatostatin B; AST-C, allatostatin C; CCAP, crustacean cardioactive peptide; CHH, crustacean hyperglycemic hormone; DH31, diuretic hormone 31; DH44, diuretic hormone 44; ETH, ecdysis-triggering hormone; ILP, insulin-like peptide; NPF, neuropeptide F; PDH, pigment dispersing hormone; RPCH, red pigment concentrating hormone; sNPF, short neuropeptide F; TRP, tachykinin-related peptide; R, receptor.

Query proteins used for tblastn searches: ACPR, *Tribolium castaneum* ACP receptor (**ABX52400** [74]); AST-AR, *Drosophila melanogaster* allatostatin A receptor 1, isoform B (**AAF45884** [72]); AST-BR, *Daphnia pulex* allatostatin B receptor (**EFX87704**; [65]); AST-CR, *D*. *melanogaster* allatostatin C receptor 1 (**AAF49259** [72]); allatotropinR, *Manduca sexta* allatotropin receptor (**ADX66344** [75]); bursiconR, *T*. *castaneum* bursicon receptor (**ABA40401**; unpublished direct GenBank submission); CCHamideR, *D*. *melanogaster* CCHamide-1 receptor (**AAF57819** [72]); corazoninR, *D*. *melanogaster* corazonin receptor, isoform A (**AAF49928** [72]); CCAPR, *D*. *melanogaster* crustacean cardioactive peptide receptor (**AAF56536** [72]); CHHR, *Bombyx mori* neuropeptide receptor A2 (**BAG68400** [76,77]); DH31R, *D*. *melanogaster* diuretic hormone 31 receptor, isoform A (**AAN16138** [72]); DH44R, *D*. *melanogaster* diuretic hormone 44 receptor 1 (**AAF58250** [72]); ETHR, *D*. *melanogaster* ETHR, isoform A (**AAF55872** [72]); FLRFamideR, *D*. *melanogaster* FMRFamide receptor, isoform A (**AAF47700** [72]); ILPR, *D*. *melanogaster* insulin-like receptor, isoform A (**AAF55903** [72]); intocinR, *T*. *castaneum* arginine vasopressin receptor (**ABN79656** [73]); leucokininR, *D*. *melanogaster* leucokinin receptor (**AAF50775** [72]); myosuppressinR, *D*. *melanogaster* myosuppressin receptor 1, isoform A (**AAF47635** [72]); NPFR, *D*. *melanogaster* neuropeptide F receptor, isoform A (**AAF51909** [72]); PDHR, *D*. *melanogaster* pigment-dispersing factor receptor, isoform A (**AAF45788** [72]); proctolinR, *D*. *melanogaster* proctolin receptor, isoform A (**AAF45980** [72]); pyrokininR, *D*. *melanogaster* pyrokinin 1 receptor, isoform D (**AAX52950** [72]); RPCHR, *D*. *melanogaster* adipokinetic hormone receptor, isoform A (**AAF52426** [72]); RYamideR, *D*. *melanogaster* RYamide receptor, isoform A (**AAF56655** [72]); sNPFR, *D*. *melanogaster* short neuropeptide F receptor, isoform A (**AAF49074** [72]); SIFamideR, *D*. *melanogaster* SIFamide receptor, isoform A (**AAN13859** [72]); sulfakininR, *D*. *melanogaster* cholecystokinin-like receptor at 17D3 (**AAF48879** [72]); TRPR, *D*. *melanogaster* tachykinin-like receptor at 86C, isoform A (**AAF54544** [72]).

**Fig 2:**
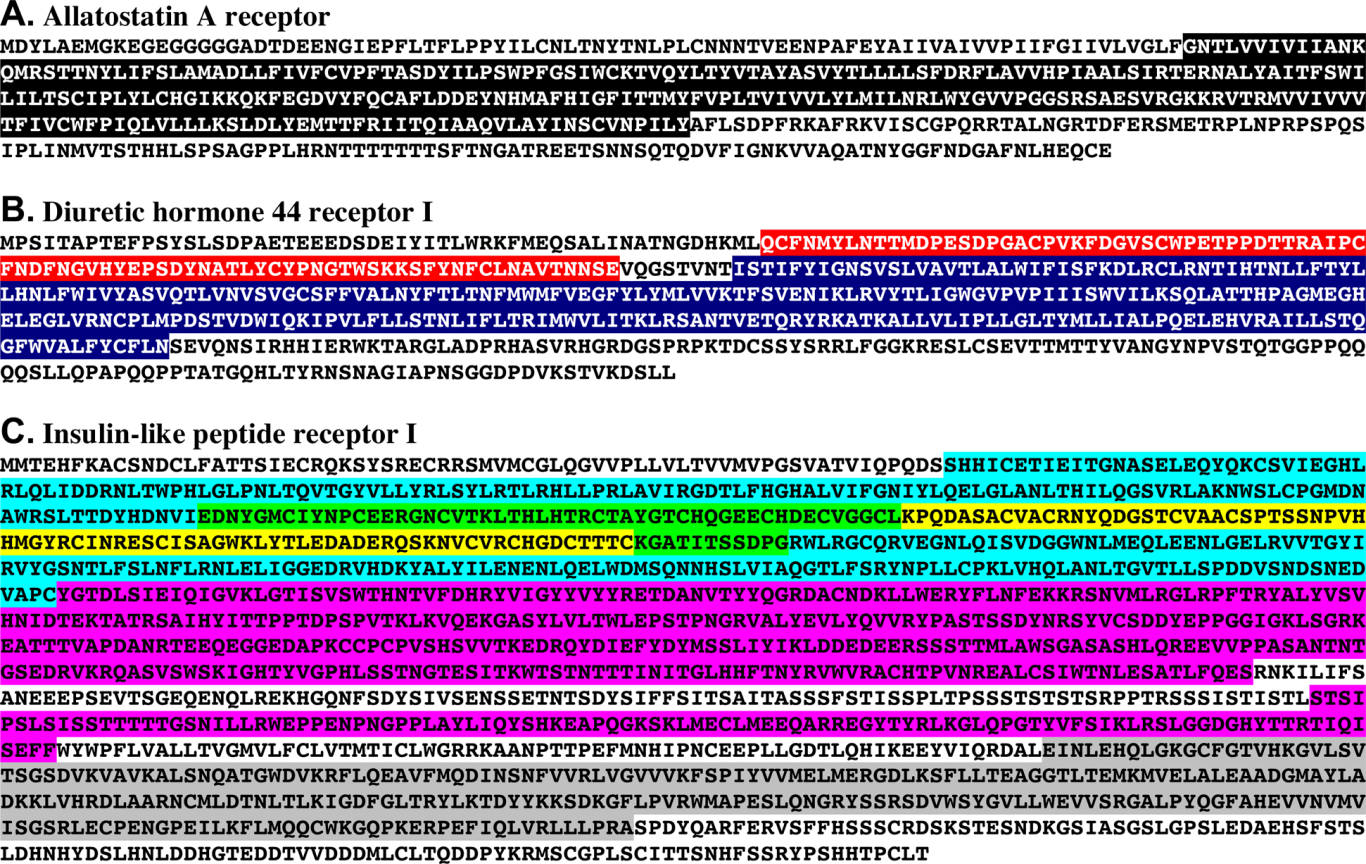
Three examples of putative full-length *Homarus americanus* neuropeptide receptor proteins and the functional domains identified within them using the online program InterPro. (A) Allatostatin A receptor. In this schematic, the single rhodopsin-like G protein-coupled receptor (GPCR) seven transmembrane domain identified by InterPro is highlighted in black. (B) Diuretic hormone 44 receptor I. In this schematic, the GPCR family 2 extracellular hormone receptor domain identified by InterPro is highlighted in red while the GPCR family 2-like seven transmembrane region identified by InterPro is highlighted in dark blue. (C) Insulin-like peptide receptor I. In this schematic, the two receptor L-domains are highlighted in light blue and green, the one furin-like cysteine-rich domain is highlighted in green and yellow, the two immunoglobulin-like fold domains are highlighted in pink and one protein kinase-like domain is highlighted in gray.

Translation of the identified transcripts allowed for the prediction of one ACP, one AST-A, three AST-C, two bursicon, two CCHamide, one corazonin, one CCAP, three DH31, two DH44, three ETH, one FLRFamide, two ILP, one leucokinin, one myosuppressin, four NPF, two PDH, two proctolin, one pyrokinin, two RPCH, one sNPF, one SIFamide, one sulfakinin and three TRP receptors (Table 3, Fig 2 and S4 Fig). These predicted proteins included both full-length and partial proteins (Table 3 and S4 Fig). Ligand attributions are based on reciprocal protein BLAST searches against the annotated *D*. *melanogaster* proteins in FlyBase (when a homolog was known to be present in the dataset) and/or the non-redundant arthropod proteins curated in GenBank. For example, when the putative *H*. *americanus* AST-A receptor (Fig 2A) was used to search FlyBase for the most similar protein, allatostatin A receptor 1, isoform D (**FlyBase No.** **FBpp0305932**; **Accession No.** **AAG22404** [72]) was returned as the top hit, and when this lobster protein was used to search the arthropod proteins curated in GenBank, an allatostatin receptor from the cockroach *Periplaneta americana* (**Accession No.** **AAK52473** [89]) was identified as the most similar sequence. Similarly, the top FlyBase hit for *H*. *americanus* DH44 receptor I (Fig 2B) was diuretic hormone 44 receptor 1 (**FlyBase No.** **FBpp0086614**; **Accession No.** **AAF58250** [72]), with the body louse *Pediculus humanus corporis* Class B secretin-like G-protein coupled receptor GPRdih1 (**Accession No.** **XP_002424517**; Kirkness et al., unpublished direct GenBank submission) returned as the most similar arthropod protein in GenBank. For *H*. *americanus* ILP receptor I (Fig 2C), insulin-like receptor, isoform D (**FlyBase No.** **FBpp0288671**; **Accession No.** **ACL83551** [72]) was found to be the most similar protein in FlyBase and the insulin-like receptor of the shrimp *Macrobrachium rosenbergii* (**Accession No.** **AKF17681**; Sharabi et al., unpublished direct GenBank submission) was identified as the most similar arthropod protein in GenBank. The results of all of the FlyBase and GenBank non-redundant arthropod protein blastp searches are summarized in S2 and S3 Tables, respectively.

### Structural analyses of deduced receptor proteins

For all full-length putative receptor proteins for which no uncalled amino acids were present (*i*.*e*., one AST-A, two AST-C, two CCHamide, one DH31, one DH44, two ETH, one FLRFamide, one ILP, one leucokinin, one myosuppressin, four NPF, two PDH, one proctolin, one sNPF, one SIFamide, one sulfakinin, and one TRP receptor), amino acid sequence analysis and protein family classification were conducted using the online program InterPro [79–81]. Here, the assumption was that each protein would possess a structure typical of those of known peptide receptors, *e*.*g*., seven membrane-spanning regions, and, in some cases, hormone receptor and/or other functional domains.

**Fig 3:**
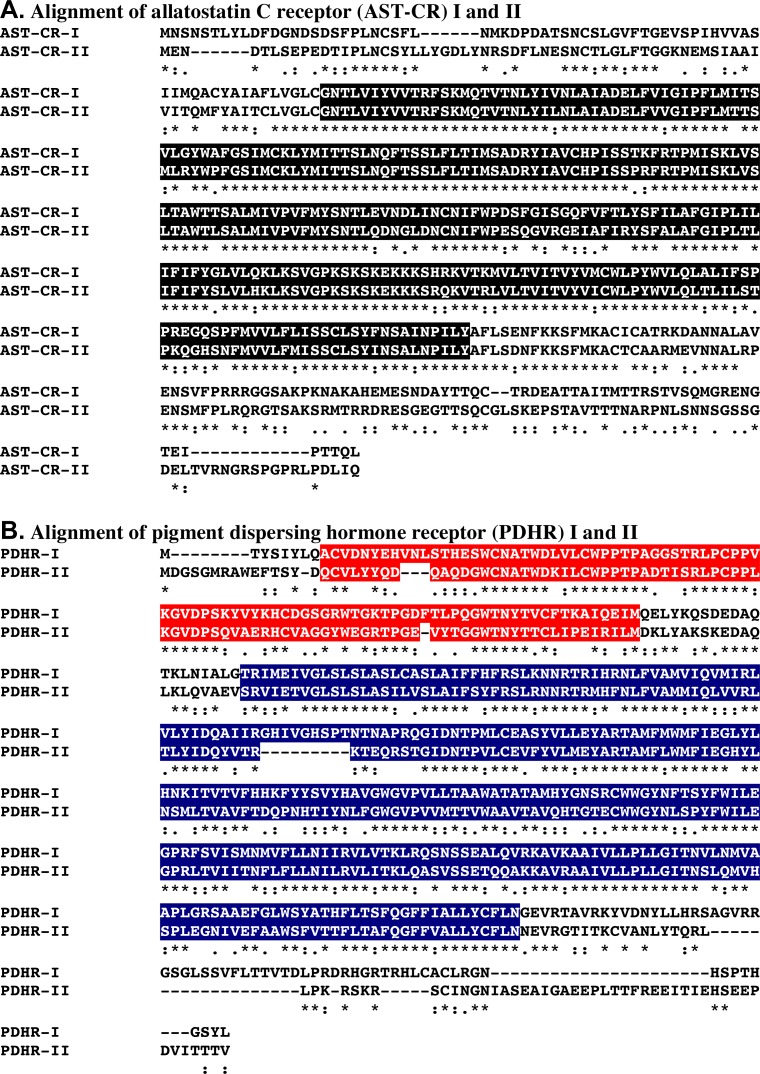
Amino acid sequence alignment of selected *Homarus americanus* receptors for peptide families in which multiple full-length receptors were identified. (A) Alignment of allatostatin-C receptor (AST-CR) I and II. (B) Alignment of pigment dispersing hormone receptor (PDHR) I and II. In each panel, “*” located beneath each line of the alignment indicates residues that are identical in the two sequences, while “:” and “.” indicate highly conservative and conservative substituted (similar) amino acids, respectively, shared between the two proteins. In this figure, the rhodopsin-like G protein-coupled receptor (GPCR) seven transmembrane domains identified by InterPro in each of the two AST-CRs are highlighted in black, while the GPCR family 2 extracellular hormone receptor domain and the GPCR family 2-like seven transmembrane region identified by InterPro in the each PDHR are highlighted in red and dark blue, respectively.

InterPro analysis of the AST-A (Fig 2A), AST-C I (Fig 3A), AST-C II (Fig 3A), CCHamide I, CCHamide II, ETH I, ETH II, FLRFamide, leucokinin, myosuppressin, NPF I, NPF II, NPF III, NPF IV, proctolin I, sNPF, SIFamide, sulfakinin and TRP I receptors placed each of these proteins into the rhodopsin-like G protein-coupled receptor (GPCR) superfamily (**InterPro ID No.** **IPR000276**), with each protein predicted to possess a single rhodopsin-like GPCR seven transmembrane domain (**InterPro ID No.** **IPR017452**) (highlighted in black in Figs 2A and 3A and in S4 Fig). Analyses of the DH31 I, DH44 I (Fig 2B), PDH I (Fig 3B) and PDH II (Fig 3B) receptors using InterPro identified these proteins as members of the secretin-like GPCR family 2 superfamily (**InterPro ID No.** **IPR000832**). A single GPCR family 2 extracellular hormone receptor domain (**InterPro ID No. IPR001879**) (highlighted in red in Figs 2B and 3B, and in S4 Fig) and a single GPCR family 2-like seven transmembrane region (**InterPro ID No.** **IPR017981**) (highlighted in dark blue in Figs 2B and 3B, and in S4 Fig) was identified in each of these receptors. No protein family membership was identified by InterPro for the ILP I receptor (Fig 2C and S4 Fig). However, analysis using this program did predict a number of functional regions within this receptor’s sequence, including two receptor L-domains (**InterPro ID No.** **IPR000494**) (highlighted in light blue and green in Fig 2C and S4 Fig), one furin-like cysteine-rich domain (**InterPro ID No.** **IPR006211**) (highlighted in green and yellow in Fig 2C and S4 Fig), two immunoglobulin-like fold (fibronectin type III subtype [**InterPro ID No.** **IPR003961**]) domains (**InterPro ID No.** **IPR013783**) (highlighted in pink in Fig 2C and S4 Fig), and one protein kinase-like (tyrosine-protein kinase, catalytic domain subtype [**InterPro ID No.** **IPR020635**]) domain (**InterPro ID No.** **IPR011009**) (highlighted in gray in Fig 2C and S4 Fig). Interestingly, in the type-1 insulin-like growth-factor receptor, the first three domains of this protein’s extracellular portion consist of two L-domains and a single cysteine rich region, which are hypothesized to form a binding pocket for insulin [90]; a similar situation may be at play in the lobster ILP I receptor identified here.

### Comparisons of sequences in putative receptors for a common ligand

Multiple receptors appear to exist in *H*. *americanus* for at least 12 peptide families (Table 3); for five of these peptide groups, *i*.*e*., the AST-C, CCHamide, ETH, NPF, and PDH, multiple full-length receptor sequences were deduced. To determine the degree of conservation present among the full-length receptors for a given ligand, the proteins of each of the relevant families were aligned and identity/similarity scores calculated. Fig 3 shows the alignments of the two full-length AST-C receptors, *i*.*e*., AST-CR I and AST-CR II (Fig 3A), and the two full-length PDH receptors, *i*.*e*., PDHR I and PDHR II (Fig 3B), using the online program MAFFT [91]. As can be seen from panel A of Fig 3, the two AST-C receptors are quite similar in their amino acid sequences, particularly in their seven transmembrane domain regions. Specifically, the two proteins are 65.4% identical/83.6% similar overall, with 83.9% identity/95.3% similarity over their membrane spanning regions. Similarly, the two PDH receptors exhibit considerable sequence conservation (Fig 3B), though less than seen between the AST-CRs, being 52.4% identical/76.4% similar over their full lengths, and 51.1%/77.2% and 60.8%/85.4% identical/similar in their hormone receptor and membrane spanning domains, respectively. Alignments of the full-length members of the CCHamide, ETH and NPF families are shown in S5 Fig; the levels of sequence conservation seen in these parings are similar to those reported for the AST-C and PDH receptors.

### Integration of molecular and physiological data

In our study, numerous *H*. *americanus* peptide precursor- and receptor-encoding transcripts were identified. The discovery of these sequences allowed the prediction of a large and diverse neuropeptidome, as well as a large collection of receptor proteins. With respect to the peptides discovered here, many possess structures that place them into well known families, including some for which multiple isoforms are present, *e*.*g*., the 23 AST-As, 13 leucokinins, nine FLRFamides and seven pyrokinins that were predicted. Similarly, for most peptide families, multiple receptors were discovered, *e*.*g*., the four distinct proteins that putatively have NPF as their ligand. At present, the functional consequences of the diversity seen here in neurochemical signaling systems of *H*. *americanus* remain unknown. However, prior physiological investigations do allow for speculation and, in a few cases, at least partial support for existing hypotheses.

First, it is well known that the *H*. *americanus* stomatogastric and cardiac neuromuscular systems, while numerically simple (just nine neurons in the case of the lobster cardiac ganglion [2]), are able to produce a large number of distinct behavioral outputs. Much of this functional flexibility has been attributed to the actions of neuromodulators, including peptides, on the neurons and muscles that make up these central pattern generator-effector systems [1–10]. Given the enormous complexity that can be derived from the complements of neuropeptides and receptors discovered in our study, which is likely incomplete, it is not at all surprising that these systems are capable of producing diverse outputs.

The actions of neuromodulators on the lobster nervous system are well known to be highly state-dependent, with different individuals often responding differently to the same neuroactive compound. For example, the modulatory actions of the peptide pQIRYHQCYFNPISCF (disulfide bridging between the two cysteine residues), a member of the AST-C family (and a peptide rediscovered here), on the cardiac neuromuscular system vary considerably among individuals [92]. Specifically, perfusion of this peptide through the semi-intact heart consistently decreased the frequency of ongoing heart contractions, but showed varied effects on contraction amplitude, decreasing it in some lobsters and increasing it in others. This differential response was found to be due to actions of the peptide on the cardiac neural circuit itself; peptide applied to the cardiac ganglion of hearts that responded with increased contraction force showed marked increases in both motor neuron burst duration and the number of spikes per burst, whereas peptide application to cardiac ganglia from hearts that showed a decrease in contraction amplitude resulted in only marginal increases in these parameters, suggesting that decreased contraction amplitude of the heart was the result of the non-linear neuromuscular transform [93]. At the mechanistic level, it is possible that differences in the response to AST-C are due to differences in the complement of AST-C receptors present in the cardiac ganglion in preparations that respond to AST-C with increases vs. decreases in contraction amplitude; this would require at least two distinct AST-C receptors. Consistent with this hypothesis, our data support the existence of at least three distinct AST-C receptors in the lobster, as transcripts encoding two full-length and one partial protein with sequence homology to known AST-C receptors were discovered.

In *H*. *americanus*, immunohistochemical mapping suggests that pyrokinins are present in the two major neuroendocrine systems (the X-organ-sinus gland complex and the pericardial organ) and in the neuropil of both the cardiac and stomatogastric ganglia [94,95]; this distribution suggests that they could serve as both hormonal and locally-released neuromodulators in the cardiac and stomatogastric neuromuscular systems. Prior to our study, the sole member of the pyrokinin family known from the lobster was FSPRLamide [23], an atypical peptide in that all other members of the pyrokinin family are N-terminally extended relative to the F*X*PRLamide consensus motif (where *X* represents a variable residue). Moreover, in nearly all crustaceans thus far investigated, multiple pyrokinin isoforms have been detected [23,30,34,36,40,42,43,96–98]; *H*. *americanus* and the crab *Callinectes sapidus* are the sole exceptions [23,96], and in the latter species, the known isoform is N-terminally extended [96]. Since FSPRLamide (and a number of other N-terminally extended native pyrokinins and synthetic analogs possessing–FSPRLamide C-termini) had no effect on the lobster cardiac neuromuscular system, while ADFAFNPRLamide (a pyrokinin from the shrimp, *Litopenaeus vannamei* [98]), and to a lesser extent the synthetic analog SDFAFNPRLamide, increased both the frequency and amplitude of heart contractions, it was hypothesized that: 1) FSPRLamide is a truncation of an N-terminally extended peptide or peptides present in the lobster; and 2) that multiple isoforms of pyrokinin exist in the lobster, with at least one ending in–FSPRLamide and another ending in–FNPRLamide. The peptides predicted from the two pyrokinin precursors deduced here support the first hypothesis as all of the full-length isoforms are N-terminally extended relative to the F*X*PRLamide consensus motif (or a close approximation thereof). Similarly, seven full-length pyrokinins were predicted, at least partially supporting the second hypothesis. Interestingly, none of the predicted lobster peptides possessed a–FNPRLamide C-terminus. Thus, the prediction of an N-terminally extended pyrokinin possessing this ending was not confirmed. This said, both of the pyrokinin precursors predicted here are partial proteins and it is possible that the “missing”–FNPRLamide peptide (or peptides) is present in the portions of these preprohormones that were not identified here.

Comparison of the effects of pyrokinins on the *H*. *americanus* cardiac system (where only one native crustacean isoform was found to be bioactive [94]) with those seen in the lobster stomatogastric ganglion (where all of the native crustacean pyrokinins tested produced essentially identical physiological responses [95]) has resulted in the hypothesis that there are at least two pyrokinin receptors in this species, one highly isoform-specific and the other promiscuous in its pyrokinin specificity. Moreover, it is predicted that the promiscuous receptor is absent from the cardiac neuromuscular system. However, only a single pyrokinin receptor was discovered here. At this point, it is not clear whether this protein is a promiscuous or isoform-specific receptor. Given the identification of just one pyrokinin receptor, at least two possible explanations exist for the distinct physiological effects of pyrokinins seen in the stomatogastric and cardiac systems. First, there may be an additional pyrokinin receptor that was not identified here. Second, the same receptor may utilize different second messenger pathways when activated by different pyrokinin isoforms. Such a situation exists in the responses to tachykinins in the stable fly, *Stomoxys calcitrans*, were four different tachykinins were able to activate the same receptor (Stomoxys calcitrans tachykinin-related peptide receptor (STKR), but elicited different effects via distinct second messenger systems [99]. Clearly, additional experimentation will be required to determine the pathways that underlie the differential responses to pyrokinins in these two lobster ganglia.

## Materials and Methods

### Transcriptome sequencing and assembly

#### Tissue collection and RNA preparation

Adult lobsters, *H*. *americanus*, (N = 2) were obtained from The Fresh Lobster Company (Gloucester, Massachusetts, USA) and maintained in artificial seawater at 12°C until used. Lobsters were anesthetized by packing them in ice for 30 minutes before dissection. Following anesthetization, the brain (supraoesophageal ganglion), ventral nerve cord, cardiac ganglion and complete stomatogastric nervous system (which includes the paired commissural ganglia and the single oesophageal and stomatogastric ganglia) were dissected out of each individual and pinned out in a Sylgard (Dow Corning)–coated dish containing chilled (12–13°C) physiological saline. Any adherent connective tissue and muscle was removed to the extent possible, and the tissues were rinsed several times in physiological saline (composition in mM/l: 479.12 NaCl, 12.74 KCl, 13.67 CaCl_2_, 20.00 MgSO_4_, 3.91 Na_2_SO_4_, 11.45 Trizma base, and 4.82 maleic acid [pH = 7.45]) made with ultrapure, RNAse free water. After dissection, tissues were combined and homogenized in Trizol (Invitrogen). Insoluble tissues were pelleted by centrifugation and the supernatant removed and stored at -80°C until RNA extraction. Total RNA was isolated as per the protocol provided by the manufacturer (Invitrogen), and subsequently treated with DNAse prior to library construction.

All animal work has been conducted according to relevant national and international guidelines. No IACUC review was needed, as this study used an invertebrate species.

#### Library production, sequencing and *de novo* transcriptome assembly

Library construction and RNA-sequencing were performed for a fee by GENEWIZ, Inc. (South Plainfield, New Jersey, USA). In brief, RNA samples were quantified using Qubit 2.0 Fluorometer (Life Technologies, Carlsbad, California, USA) and the RNA integrity was checked with RNA6000 Nano Assay using an Agilent 2100 Bioanalyzer (Agilent Technologies, Palo Alto, California, USA). cDNA library preparation and sequencing reactions were conducted by Genewiz, Inc. Illumina TruSeq RNA library prep, clustering, and sequencing reagents were used throughout the process following the manufacturer’s recommendations (Illumina, San Diego, California, USA). Specifically, mRNAs were purified using poly-T oligo-attached magnetic beads and then fragmented. The first and the second strand cDNAs were synthesized and end repaired. Adaptors were ligated after adenylation at the 3’ends. Then cDNA templates were enriched by PCR. cDNA libraries were validated using a High Sensitivity Chip on the Agilent 2100 Bioanalyzer. The cDNA library was quantified using Qubit 2.0 Fluorometer (Life Technologies, Carlsbad, California, USA) and by qPCR. The samples were clustered on a flow cell using the cBOT. After clustering, the samples were loaded on the Illumina HiSeq 2000 instrument for sequencing with a 2x100 paired-end configuration.

Raw sequence data generated from Illumina HiSeq 2000 was converted into fastq files and de-multiplexed using Illumina CASSAVA 1.8.2 program. Fastq files from the sample were imported into CLC Genomics Workbench Server 5.0.1. Sequence reads were trimmed to remove bases with low quality at ends. *De novo* assembly was conducted with the trimmed reads utilizing the CLC Genomics Server; 60,273 unique transcripts were obtained. The average length of the transcripts was 1,657 bp and the N50 was 2,357 bp. The total length of the assembled transcripts was 99,847,148 bp. The assembled sequences were blasted against NCBI nucleotide database and annotated using the top BLAST hit. In addition, open reading frames were predicted for the assembled transcript sequences.

### *In silico* transcriptome mining, peptide prediction and protein structural analyses

#### Transcriptome mining

Searches of the *H*. *americanus* transcriptome described above were conducted on a TimeLogic DeCypher server using a protocol modified from several recent publications [40,82–84]. Specifically, the lobster assembly was selected as the database to be searched using the DeCypher Tera-BLASTP algorithm, and a known neuropeptide precursor or receptor was input to the program as the protein query. The complete list of pre/preprohormones searched for, as well as the specific queries used, is provided in Table 1; the full list of receptors searched for (and the query proteins used) is provided in Table 3. All hits returned by a given Tera-BLASTP search were fully translated using the “Translate” tool of ExPASy (http://web.expasy.org/translate/) and then checked manually for homology to the query sequence. The BLAST-generated maximum score and E-value for each of the transcripts identified as encoding a putative neuropeptide precursor or receptor are also provided in Tables 1 and 3.

#### Neuropeptide prediction

The structures of mature neuropeptides were predicted using a well-established workflow [29–43,47–55]. Specifically, each of the deduced precursor proteins was assessed for the presence of a signal peptide using the online program SignalP 4.1 (http://www.cbs.dtu.dk/services/SignalP/ [100]), with the D-cutoff values of the program set to “Sensitive.” Prohormone cleavage sites were identified based on the information presented in Veenstra [101] and/or homology to known pre/preprohormone processing schemes. When tyrosine residues were present, prediction of their sulfation state was done using the online program Sulfinator (http://www.expasy.org/tools/sulfinator/ [102]). Disulfide bonding between cysteine residues was predicted by homology to known peptide isoforms and/or using the online program DiANNA (http://clavius.bc.edu/~clotelab/DiANNA/ [103]). Other post-translational modifications, *e*.*g*. cyclization of N-terminal glutamine/glutamic acid residues and C-terminal amidation at glycine residues, were predicted by homology to known arthropod peptide isoforms.

#### Analysis of receptor conservation and structure

To determine the proteins most similar to the neuropeptide receptors identified in this study, each protein was used to query the annotated *D*. *melanogaster* proteins dataset present in FlyBase (version FB2015_04 [78]), as well as those present in the non-redundant arthropod protein dataset (taxid:6656) curated in GenBank, using the blastp algorithm [104].

To determine amino acid identity/similarity between proteins (and structural motifs [see below]), the sequences in question were aligned using MAFFT version 7 (http://align.bmr.kyushu-u.ak.jp/mafft/online/server/ [91]), and amino acid identity/similarity was subsequently determined using the alignment output. Specifically, percent identity was calculated as the number of identical amino acids (denoted by “*” in the MAFFT output) divided by the total number of residues in the longest sequence (x100). Amino acid similarity was calculated as the number of identical and similar amino acids (the latter denoted by the “:” and “.” symbols in the protein alignment) divided by the total number of residues in the longest sequence (x100).

Protein structural motifs were analyzed using the online program InterPro (http://www.ebi.ac.uk/interpro/ [79–81]).

## Supporting Information

S1 Fig Nucleotide sequences, transcript identification numbers and translation frames of *Homarus americanus* peptide precursor-encoding transcripts. (DOC)

S2 Fig Amino acid sequences of deduced *Homarus americanus* peptide pre/preprohormones. In this figure, signal peptides are shown in gray, while all mono/dibasic cleavage loci are shown in black. For each sequence, the isoform(s) of the peptide for which the precursor is named is/are shown in red, with all linker/precursor related peptides shown in blue. The “+” symbol indicate the presence of additional, unknown, amino acid residues at the amino- and/or carboxyl-terminus. Uncalled amino acids have been colored green.
(DOC)

S3 Fig Nucleotide sequences, transcript identification numbers and translation frames of *Homarus americanus* peptide receptor-encoding transcripts. (DOC)

S4 Fig Amino acid sequences of deduced *Homarus americanus* peptide receptors. In the full-length proteins shown in this figure that do not contain any uncalled amino acids (colored green), rhodopsin-like G protein-coupled receptor (GPCR) seven transmembrane domains are highlighted in black, GPCR family 2-like seven transmembrane regions are highlighted in dark blue, GPCR family 2 extracellular hormone receptor domains are highlighted in red, receptor L-domains are highlighted in light blue and green, furin-like cysteine-rich domains are highlighted in green and yellow, immunoglobulin-like fold domains are highlighted in pink and protein kinase-like domains are highlighted in gray. The “+” symbol indicates the presence of additional, unknown, amino acid residues at the amino- and/or carboxyl-terminus.
(DOC)

S5 Fig Alignments of the full-length members of the CCHamide, ETH and NPF receptor families. (DOC)

S1 Table *Homarus americanus* precursor-related and other peptides whose structures do not place them into any recognized family. (DOC)

S2 Table Most similar *Drosophila melanogaster* protein to each Homarus americanus peptide receptor sequence. (DOC)

S3 Table Most similar arthropod protein to each *Homarus americanus* peptide receptor sequence. (DOC)
