# Supplementary material for: Neuropeptidergic Signaling in the American Lobster Homarus americanus: New Insights from High-Throughput Nucleotide Sequencing
Source: PLoS One. 2015 Dec 30;10(12):e0145964. doi: 10.1371/journal.pone.0145964 (PMC4696782; doi:10.1371/journal.pone.0145964)
Supplement: S1 Fig — (DOC) [file pone.0145964.s001.doc]

**A.** Adipokinetic hormone-corazonin-like peptide precursor protein (DS01-Homarus1_Transcript_14565; translation frame 2)

**caatcatatgtatgaagttatgtacgcggagataagtacgcatgtacgtgtaccaccagtatgtgggtgtgtgtgtcagcgcacattgctcggagggaaagatgacatcggaggcgggaggagcgaccaatggaacaacagtgggcgtaactggctacgggttcatgccctatataaaccccgacacgactgaaaatcttcagtcgagttccagccgtccagtcagagtcagctgcttgagtcactctcgccaccatcagcagcagctacctcatcgtcgccgcctcctccagcagctaacgacactcgctcttgtctttttccccacttcgaggtctcttgagcagtgatggtcggatggcaggtcatgctggcggtgatgtgcctggcgctcgcccctaccctggcgcagatcaccttctcccgttcatgggtgccccagggcaagaggtcagggggcattactggacccctcgtcacccctgggggcggctctgaccgaggagccgacccctgcaaggacgtgagactggccaccctcacccaggtcgcctcacatctggccgacttgatggatgacaccttcgatctcccccaagacgacgctgctctggctcttcgactcaaacacggacttgtggcaaggcgacgaagaatgtcttaagcagtacagcagcagtgaatgacgagatctggtaaagcctacccacccaccctcgaccatctcccgccaaaacttcaagttcaacatataaacatgaattttcccgccaaaccttaaaagctcaacatataaacatgaattttccc**

**B.** Allatostatin A precursor protein (DS01-Homarus1_Transcript_6078; translation frame 3)

**cgccgtgctctctctcttccatacggggcggtctgtgtcgacgctcactcccagtcgccgcaacactcgcttaaaaaaaatagaaaaagagacctcatctctgccactcccttttgaccactacccgctccatcattcactcttgttaactggactatttagagcattaaattacccaaccaccagctctgttgtggctttgggaagtcagaatcacttcgacgcctcctgccccagatcttcaaagcaagaatcagtggtgtaggatggtgggagagcatggcgggctgggcacgtgtgtcttggtggtggttgtcctcctgctgaccaccaccactaccacagcccacgactacctggaagacctcgatgacccagacacttcacgcctcctagatgtgctgcagtactatgacacagagcccagttacctctacgattatgggaaacgacatagcaactacggatttggcctcggaaaacgaacccccggctatgccttcggtctgggcaaacgcgagggcttgtactctttgggcctggacaaaagatcagacctctattctttcgggctaggcaagaaatcaggtagttataacttcggtttgggcaagagaagcgtcggcgatctcccggaggtctcaaaggtagaggacggggcgtctccaaggacaaagcgagacgtaagcatcaccgaggataccctagaggacaagagagccaaatactccttcgggatcggtaaaagagaaagcagcaagaacaagaggtcaaagttgtacgggtttggtctgggaaaacgagactctggtgaagaaaggagggaagacgacgacatggaaaacagaacccaacagtattccttcggccttggcaaacaggaccccgacatggaaatagagaaaaggccacgaaattatgccttcggtctgggaaagcgagaatctgatgaagattctgataaaagatctcagatgtattccttcggtcttggcaaacgggatcccgacatggacatggacaagaggccccgggattatgcttttggccttgggaaacgtgcctccagtgacgaagatgacgaggagcgttactacgcatatgaacagggaaagagacccactgcctacagtttcggtcttgggaaacgcgcttttagtgagacggatgactatgacaatgtgaacgacaacgacgatggggatgatgagctggaactcagcgacttggaacagtacagcgatgacctcaagagggctacctcgtatggttttggacttggtaagaggagcgatgcacccgacagtggttttggcaggaggtcatacgatttcggtcttgggaagagagctggtcggtatgcctttggtctggggaagaggactggtccgtatgcctttggtcttggaaagaggactggtccgtacgcctttggtctaggaaagaggactggtccgtatgcctttggtttgggaaaaagagttggtccctatgcgtttggtcttggaaagaaggctggtcattatgccttcggtcttggaaagagagctggtccatatgcctttggtcttggaaaaagaagtggcccctatgctttcggtcttggtaagagagctgatccatacgcattcggccttggtaaaaaggctgggcagtattcattcggccttggcaaaaggtcaggcccatattctttcggtcttggcaaacgtagcgattccgactccgaccaatacaccctgggaaggcgttctggagtgtattcattcggcctgggcaaaagagctggtccttatagctttggtcttggaaaacgtgaggtgagcgacgatgaccacgacgaagatgaacaagacataggagtagaggaagagatgtcatcctaagtctccttcaaggtgtctgacactacctaggcgcataattattcccatcttaacttcctcgatgagaccgaattctaaaacaaatcttagagcaagaaattactcatataattataaccccctggttttgtgaggcttagtgcggctgtattcactcctccaagagtatgtgaatgcctcattagttctgattctactaatcggagcttaattggctggctggtagtaactttcaaggatgcttttgaattcggtcttgtatcttattctctaacatatacaagacaggttccagcttcatctttcttattttctacatttctccaaatttactttacaattttaggtcaataatcataaatgtaattcctaatggtaacaccacggaggctaacctatgttgtgcctacacaactctggcctatcctataccataaactacatattgtggcaggtttcagtatgtaaaaacaaaaaaacaaaatcagtattgccaagtgtatcagttaaggtgatggtcgggcaatcaagatgctaaaaacagaggaaaataattgtttttttctttggatatgagagaatgtgttgcaaatacctccagtagccttagtctcaccagaggctaggtctgtatcaattatgttatctttagtattatatgagaaataaagtaatatatattaaagataatattatgaatttgcataacttgaaaaaataaaatcattccacctatagttttcatactattaatgtttgtgtagtgggtcattttactcaagatacggagacctgacgattgtgttattcttgccttgagactttatagaaaacttgaagcattttgtcaggtgacttggggagtactgtacacagaggatatgttctaagctttaatattatataatattttctatacgtattatattgatatttgtccaagattatttataaaaataaattaaaaggctgaagactgacattatgaagacggttgactaggctcttattgaaccttttaataatgacaatgtgatttaatgcatattgatttttatcctatagcttgtggtgaaaccattcaaaatttgtatagcagtgactaatgttttcttcaatatgcattaatgagatatattcgcatgaagagtaacggaattattctccagggatcgatttgcctaagggaagccaacctcaggacgagagaaacttgtataacaaaacctaacataaagtatagtctcttgtttattactggttatcccggtccactttgagccaatacagaagaagtgcttccggaaaggattatagtttaaacctcaagaatattgtatgtatagtgagccacactggagttctatggagtatattgattgtgtttatgccctgattccgttcattgtgtggccatgtgaccctcctacacgggagaacatgtatgctctgtaactatcgaatgtatatcaaaataagatattgaagctatttattttctatatttttcctctgttttataatatatgtttgattaaagagttggttatcattacccagtcgtgaattcatctctcaaaagaagattaataacgatattaaggttttttcccagatttatttccgtgacgttttgaagaagttgaaagttaacctaaatgaaaaatttaatatagaaaacaaataaagtaaaggactaataatatagacagtgctgaaaaggtacctcaaatcaatccagattcgtgttttgtgtacaggatgttgctgacaagtaaacctctccagtaattggggttggttttaatttacatttcatcaaagtggtggccgcttatgtggctgtgtgtaacacgtgggtcaacaatagtgagaaaaatcaccgaaaaggctacctcaggcagcaatgttggtgatccagtagataacttattagtgaatgagaagcttaatgaaattcaggaatttgaacaagatcctaacagtatgtgtcgattggtatattcagtggatctgaatgggaatctttcaactgtcatgttttactgtacaacattaaataaatcaacatcacttgcgaatctgatgtccatggcgaaaaaacttgcccgataacttaacaaataacccctcccaacaaattttcttatcgatttatatcaatcttatggatcacattcccagagggaaaaaaaaatcgcgaattaacagattttttacacgcctataaatatcacatatcatgaaatttaagacacagtaacctttgtttttgttttttaagtgattattgtagagttggaggccttaaacacctgaacactttccaggtcatgttgtatataaaaaccttctgaggtgtgaagattttgtaaatttcattttttgttgttgaaaaacgttttcaaattatttacatttatattttccacccctacccccgggacagtatctattctaaactcttcaatcagacaacggatcgtagaagacaataaaatgttgtccataaacataactctttcacgcagttatatatctcatcatttttaatttattgtggaacagtgcaattagtaaccttgataaatataaccttaaaagtccttggttgctgaagcgctgtcatgggttgcgttaggctt**

**C1.** Allatostatin C I precursor protein (DS01-Homarus1_Transcript_3087; translation frame 3)

**gactgcaggcgatgatgaaccatttgcatatgaacaaacaacaacagcagcagcaacaacaacaacaacaacaacagcagcagcagcagcagcaacaacaacaacaacaacaacaacagcagcagggagaagaagaggttaagaggaagaggatgttcgtgccactctctggccttcccggtgaactgcccaccatcaagaggcagatccgttaccaccaatgctacttcaaccccatctcctgcttcaggcggaagtgagcaagctgactccctgatgacgtcatggccgggtagtgacgtcacatccaacttggtgacgtcacagcctgcctaatacgtcacaatacccgtctggttacgtcaaggtcaacctggtaacgtcactgtctgttattgacgtcacagacagctggtgacgtcagtagcagcgtggtgacgtgtggcctagctggtgacaccacgagggaacaaaagacagtagctttggactacgtgccaaacaaacaaatgcttaactagtttgtgcttcagacgaaacaataatatagcaattaaaaattatattaagtagatgttgatttattccttaatctccaggcagtgatggttgttgggcgtgacacttgtgttcttaaggagaagtgatggcgcgtacgtctcagtggacctgtaaaatgactgttgtgtattcggattttcaaaataaataaataaatcctgtgacatattgctcgcccaaactcaggagagagagagaacgcagtcaagtattgctatagtctcagtacaatttacactccaatttgaccaccatacttgtgccatagcttatctgagccagttggatgcgttctcacatctccagttaagggaagaattttacggaatgaacctttgagtaacagcgttggtcaacaaggggggaagaggtccccagctgatgtgagggaggggcgtacgaggacgcttgctgagggagggtggaggggttgccagcctgctcccccactctcctccttgcacctccccacaagcacctccgcctcccgtacccacgaagtcattttct**

**C2.** Allatostatin C II precursor protein (DS01-Homarus1_Transcript_39; translation frame -1)

**cccgatatgtgtaaaaattgcattagctttaataacattgagatatttaaagttatagatacaatttaccacattatgtattacatattctggaacttttaaatacgtaaaaaagtttccttatgaatcattttaattcttatgatgggcccttatgttgccgtgtgtcctgctgtctgtccgtccaagagatattaatttccatagttgtgttggtgtaccacaagaggtcagatgagttcagtcgacaagattttctatcatatattttttttcagattccttcgatatattgtcatgctgtaatgattaatgtactcacctttagctgcaaccagcgatcatcggcaactggttcacgccctaggcaagacccatccttccagctgtaatggttccctaacttacgactgggaaggtgaagcatgagtatagtgccgccattttgctttcttgggtaccgaagagttagtgtacagtgtgttgtatgcacactgctgctaactggtcgccaggcctatgagattaattccctacttgtgttgctccattagccttggttggtgttaagttaggtacaagtaatacaagtgtgttaatcagccaattttatttcaaaatatgtgacataaataaagtaagtaaccgtcatgaataaagcgaagtgcaattcataaatgatttatcaatgaggaaagtcttctgacctctggctacaggaagcctccgtcagtgaggccgtgaggcgccgccctagtgactgctgacacaacagactgccattgtgactgctgacacaagacaaccgtcatggcttgctgacacaacagcctatcaatgtggtttgctgacacaaaagacagcgccatctacagcctcgtgcagtcaacaagcagataaacgaataatagagaaactcactgtaataaaagttaaaatactgaataatgacacaactaacagtgtagtgtgatgctcctctgcacgctaagatgaagtgtgagatttagggctttttattatttatggttacaacctacgctacataatatgttaacatccacgggttcatctgcagttcctgaaagagttgcacgctgggctggactgggctggacactggctgtgtggaccataaacgagtatttcccgccatgatgcagcagcacaccacaagcctatcatggtgatccacgggtcgtccacactaagcttttatatatgcaattacagacagacaatattttatatatatatatctagtactgatacatctctatgtgcagtatagtaaatattaattaaatataaagcagacgacgggaacagacctcaacaggctctgtggtgagaagcgtcatcctcagttccctcaagactcagtgacgtaacaaatgcaggatatactttgaactgtttaagcttattactacacggagaggcccactttattgccttctatcagtcagtcgtaattttatcacataatttatttctctccgacgaatatttgaatgaggaggaccagaccaaagggtgaggatnnnnnnnnnnnnnnnnnnnnnnnnnnnnnnnnnnnnccaacaccgcttgaccaccgttccctgcctctccgctcctcccgccgctgactctgcttgctgccttagctggcgtgttcacttcctcttgccgaagcaactgacggcgttgaaggcgcactgcttccagtaggagcgtttcctctgcaggtccttgatgtcggcgttgtttctcagacgctccaccatctgcttggcgaagaggtagttgatgagcacagcgtccagggagccatcgtcgtcaatcagatggttcccggctgggtccaacatgtggggcatctggccatatacctgggggtcttggtcaggtagcgccttggctgacaccctgctgactcccaacagggccaccacagccactaccgctaccacgctcacaaaccgcgtcagcatattgtcgttcgttgggtggaggtgaactaaggaagagtgcaggcgagggactgactgactgggggccgctcgctgggttcttagctggaggtgcgcgcgttatgaactcctccttagttcacctccacc**

**D.** Bursicon α precursor protein (DS01-Homarus1_Transcript_10307; translation frame -3)

**tagttagtagacaccagagactattgtttggtaggtgttacttaattttgtttacaacccaaaatacctttttcctccattccttgagatcaatttttaaatattgtgtaaataaggagttgtgaagaggttagttaagtaacaaagaatagagtacttggtgttcatgtcactgccatttattgttatgacgaggaaatgaaactaaactgttgaaaaggttcttgtgatgaacttatgcttgtccccagtttgtcaaatggagtgctgttgcagtattgttgtcactgtagttgtattttttagtactcaatttaggtcacgattatttttgttgttggtgctgctgccactgcaagatcaagatcttttctgctgctgctgctgctgctgctgctgctgctgctgctggtgctgctgcctcattttattttattactgctactgtctcacactttttaacagctgtgtcattccagtggtcataaaatactgagttgtcagtctgaagggcggaatgaaggctggactagtgataaggatggaggtggagtatgtgtgtgtgtgtgggggggggtggttggatggcatttgagaagaagtgcaaaggactaggtaataagcgagggatgtggcagaatggggttgatggataaaaaggataatgaggggaagtgttggggagtaaattgtagaacagggatggtggtgaagtacaagacaaggagttagggtaagtgtgggggagttaggatcaggatagggagtaggttagggaataaggggtggcgagtgagactaaggataaattgtgaacaattggattgggaagtcctaagggataaaatgagcaggggagaggggtgttggtgtgtgtgtgtgtgtgtgtgtgtgtggatggagtaagagattaaggttctatttcaggaagggaacattgcccatgggtgaatcatggatgaagttagcgatctcttgagccaggacagtgccctcctccacgtctgtgcagggacgacacatgcagtcaatgggtgcccgtgtcaagatcttcctccgcgtcggttcacccttgcgaactttgggacagtttaatacaacagaagcttcccgttcccccgactcttggcaacacatgcatgacctctccgtctgccacagcttactgccagatacctggacgtaggaggtacagcgtccctggcaggcgaaggaaggaatgggcttggagacgcagcctgggtaagacaggatgtggataacgggagtgagggaacactcgtccgaccacaccacggtagccacacccaggaccatcagcacccacgacaacccacccatcctgaaggagggaggtgacgctgattaatgggtgggagggcgaagtgtggttgtgtcaactccctgcacctaagttgctctcgttggtcaatactttggttggttcttggttgaggtagttttatggtgagtgtgtgtgtgtgtgtgtgtcaggggtggtgaggtaggagtggagtgtgtgagaggtgtgtaggggaggagtgtgagtgtaagggaagtgagaggcgcgactgcgagtgagaccgtgtagcacccagctttatactgctctcacctactggccacgcccctca**

**E.** Bursicon β precursor protein (DS01-Homarus1_Transcript_28875; translation frame 2)

**cacgatcgcctccaagctggacagtacgatgtggacgacagttgtgttagtggtggtgtgtgtggtagcggctccctgtacacatgccaggcgatatgacttggaatgtgagactcttccctcaaccatacacgtggctaaagaggagtttgacgaagctggacgtgtagagcggacttgtgaggaggacttggccgtcaacaagtgtgagggagcctgtgtgtccaaagtccaaccttcagtcaatactccttctggtttcctcaaggactgtcgctgttgccgggagacacacctcagggctagggaggtaacactaactcactgctatgatgctgacggcaacagactcaccggggaccgaggcacactagtcatcaaactacgagaacctgctgactgtcagtgcttcaagtgtggcgattctacacgatagttagctacatttttacctaattacctacctatatacccccctcccttactcactccctgaccagctaactgaccgtatagttcaccatgatctttttaaattcctgacgctaataaatatcccgaaaattcctgatccctaccatatggaatcaatctacaattaacacccacacctactaccatcaccatcaacacttagtactttacacggttgcacttctatacaagactctactgtgaatagctgagagtgttgcaccgtctactatatctatatatattttcaatgttatattccacacagctgaattttccacctacacgtcgaaaagcaagatttattatggagtgtcaggttgatatttgactgattaactaacagattgaatgattgagaactggatcaattagttcactgattattagaattattaattatatcattgactgagaataccgtatctgttggttgagttgtgactatgctaactgcccccacccccacgacactactccaccaattcagtcaagtatagttgttactgaagttactgaactactgaacgctgctgccgttactacttgacggagtgtgcatatcaaaatatatccacatattaattagctatatattaaaggacaggttatatatatacactgtttagatattattgaatagttttacgcttacctgtattgtagtggataaaggacttagcttaatggaaatgtaaaaaatatataatgtttaatgtttaagaataagtatacacctccaatgttgagaaatgtattgagaaaaatgcctccatgactaacaattaaagaaacaatactgctactgcctcacacctattaacaactgctgctgaccacccattgacctgagaattaatgttaatatagtggtgagtgttgtcaaagcattaaaaaaggaggaacaagaatattttgatgttgcttgtagctattttaatgtacacacgttcataatgtatcgatataatataaagtttaatataaaa**

**F1.** CCHamide I precursor protein (DS01-Homarus1_Transcript_13319; translation frame -2)

**ttttgttcacattatcgtagttcgagtttttgtggtggaattttgtgttacgaaactggcgggattttgtttacagtagcgaacacacgtcaccatcccttaacatacagggtaaaaatcaggacaaatcagactatcttaatgaccacaaatttccgatggatttattaatacagcatcaggtttacattgaaattacagccatagtgaaatacagtaggtattgtccagcccctatgatatgtttacaatactgtataattatagtctcgatcattatagtattattgtagcctagtgtaacgaagcgtattcccttataatggatgggggggtatttttaggaataacgagtacattaacaatgatataaccaaaaaatacaattagaaaaataattatgcgtgagagataacaataaactaaaagtacaattaagaaaaataattaattacctttgagaagcttcattgatatttcccagagaagttagctttttggtatatcaatcactatataaacaaattaaataatttcactataactccctctcccatcttcagtcacaccttaaatatcctctttagatataccaaataatttatataaatacataaagtaataatattgaggtgcaaattatcttcagggcaagatgaaattacttaattttattgaggtaactgaagaataacctccattacaattgatggagatttaaaaccacaatacaatgcagagaaagacaataatcgtcataaatatagtagaatcaatatatagagagcgatggatgataaactgtaataagagtttagtataaggacacttctaaacaataacagacgacctaggataagtggaatggttgtgttgttgtggtggttgtaagtcggcttctcggtggtggtgagggagagaaggagaagggaatgttgtgggataaatagaaggagaaagaggaaaaggggtgttaaggggagtgtgtgagggggagatgatatgtgcaatcgtagggctgtccgtgtgtcgaacaattgtcaaacctccagcctcgattattttgaatgcgagggattatattataaggagggagaaggaaaggaatccatagagctgagaaatgattgaacgaataataataataattgggctatggtggagtacgtagaatatgttaaaagattatgctaagtgagtaaattagttgaaagttgaggaaacagccggtaaggagtaacacgttatttggcgggtacaaacgatgagacgttcaggttattgtttacggacaggaacattgaaatagtcatgttaagacaaggtcattcacacggacgttgaaacagagacactggggnnnnnnnnnnnnnnnnnnnnataggaacgttgaaacggtctctggggcaggaacgttatgacgatcactaagggatagacatcaaacacagatgttgacttatatagacacccattgtttgatacttcagcttgccagccaacactaccaatctaaacaatcacacgtcgatatcttaccacctacacccatccacacaatacacacaatacgctctgctgacgaggagtgacgtacatcttttctctcaagctatcaatgtcagcggcatgtttcactcctgatccatggacgagattgttaacttcacacctttaataatgctggttgtcttcaatattgataatttttttagtattctaatatactgtacattatctattgtttccaaatgcacttaatttagcagtatttataaaagattttcaacaaattatttgcatggctgctgatgaaacaatgggaggagggagagaggggtggagggagaaagggacattagtgctgaagatggaagagggatcattctggttcagcctcaatcagaaaaggtcaatagcatgagacaaaagataatagaaggctcaggtgaaggtagcaggcctgggaataacacacgactaagataaacaagaggagaaaaaacagaataaagggggaaataaaatctgatttcaaaacttgagcggtgataactgtgaggcgagttgtgaaggcataccagaatctacttatggagaatgctacaatatttatccatatgatcgtcggctgcaatgtcgcgagtttcccttaaaatttaactaagataaacctcctctggttttagtgttactgtcccagtttgtagactttaatcatcagtttttagaaaaatcttttataaatagaggaactgagacgcctggttcagggttcaacgcaacaatgtcaacatttttatgtctgtttttccctctgatgtgaaaacaagacgtcacaatcccatgatgttactggaactagaagtcgtttcttctgtcgtcgagggtagctgatgggacgtgtatatttggagaaggcagctggtacgacatcgacgacgaagacgtagtgtttactggaagtagccgagggactgcgcggagggggatgactgtggtgacgtcctctggcggagacggtgactgaggacagccagccagtttcgggcgttggcgacgatttctcggtcggtgactgcctcgtccactgagaggcgatcctcttgcctctgttcgaattctggtagctggttggcctcagggtaaagaggtgagggttcctggcgcgcatattggtcaccgtctcttttgccgtgcgcgccaaaacacgagtgtccgaactgcgaacagctgccccaggcctgggaggagagggcaacgacgccgagcacgaccaggaacacgttgatcatcccgcgagacatgactgctggaggagtcgcggatgggacgactgtgacggagagcgacggtgggtaggacggctgtggtgcgggccggtggtgggaccaagtttcagaccaacgctaaaagctccgtcgtttgatcctgttgttgcaggtctttgaaacgtcaagattttttgtttgtttttcccgtaacgttggccactgtttgatgttgtgttgtgggtgacgggaccagggctggagatagtttcggggagaatttctttttgcagtagactgtgacggtggggagcgggaagagtgtgtttttagcgggagtgttccacgcactacaccttggaccaaatatgattagtccgggattctctgttgccttataaacggttgtactcac**

**F2.** CCHamide II precursor protein (DS01-Homarus1_Transcript_12464; translation frame -2)

**tggtcgaagaaatggctgctaaggttcaatgtcacaaagtgtaagaggatgcacctgggacgtaccaacgtaaggagacgtagcgtatgtctcaatggcatgaagaaactgcattaattgagagtgaatatgaaaaggacctaggtgtgtatgttgacaatgaactgaagttctcgggccattgcgagaaggtgaatcaagcaactacattaaattattcggtcttattagaaggtcgttcagatatctagannnnnnnnnnnnnnnnnnnnnnnnnnnnnaaatgtccaaaggaggtccaccaggctggtgccagcacttcggcacctcagctaccctgagagattagaacacttgcaattgcctagcttatactacagatgggcaaagggggaccctaattgaggtgtataaacatctcaaagggcactactcagccgagtgtccttacctggagttggaggacgctcgccccacaaggggtcacagcttcaggttgaagaaaccacaagctcagaaaattatctgggcaaaatttttggagtgagagaggtaaactcttaaacaatctgccagagtgtgtgtgatagcactgagtgtgagctcattcacagccagacttgatcaacactggcacaggtagaggtactacattctggagccaatacatgacctgtacttgttaacaatgtgtaacagagacatgactgacaacccaagcggctaatgagctgatcagtggcgaagaggattaagtaaagtaaatatatcttaacactgtttataagccaatgatatagttcgaatccagttccacatttcagcagaactcgtcagcaatgtgttttcttgtatgttctcagtgattttcccttttaaagatgtcaggaaagctatcaacaaatgaaatgaacagcagtaatacatctcttttgctgttatagtggaggtgagaaggtgcattgaggctggcgaaaaggcgacaaccattggtagttttaagggtacccggcatcttaggtaggctaagttaactgtagtactgtaactattcaacaattttttttatttattaaattgttgcttatgtacacagtactacattatcaaattttacttatgcaaatattcaagttgtattgtaaagtgtgttctgattcaaagctgaccgaccttattgaaagtcaaggtataccttaattgcactgtatcatcaaacgatgtataaccttacgtatattttaacttgtgagaatgaaaatagatttctgtcgtaacctctgatagctcactacaagttaatgctttattctcacgtctcattatcttttcttgagaaattcacagactatcttaaaaaaaaacgtggtatactggtatactacccaaaagagaattatgttcgtatcaccatttctcttgtgaattagagctcatatgtgttgagaagtaaacaaaaggaagattcagaatatgaaacttaactgttgtgtgtggtaattgtatggcagtgttgtacaggatttctataaattatgtgacccatttttaaacttaatatcttcaaaaatatgataaccagaacatgccagtcttacttacgaccagagaaaataactcgagtcctgtgcatctctataattacttaagtcataaattttaaaatgggtcatataaaatatgggccacctacataatggatgtactgtatcttttagatgacaatctcatcatcttgaggtaaatgaaaacaaaaacatatgtctttagatcgcaaatttaatatgttgtcgacacaggatattacagtatatgacattattatatacgtttttgtctaaggataactcctttatgcaagtacaacgtgatattaattacaacgaaaaattataaaatacagtaaatcattcaacaaaggatatctgtgcccaaccttttcttttctttgtttttctgcagcaaattgacctgtgcactctgagttgtcgagtcatcatcataaaatcagcaacataaaataataagctaagctatttttttccctgggagaaacggggcgtataattatagtaatggacattatccgtcttctgtagtctaattacactaattgttgctggggtgatacagtaggcctacatttatctaattaattgcgaggctaacgtcactaattttcaacgcttccacctctgtgttgacggcagagcactagattagttttcaatactgatatatatgtatatatatatccttgaatttttgaggaaatatacagtaactttttgaggcaaaaattagaaaatctttgagttactgcactttattttactgcacatattgtatatgttaaattacatactatatttttttatgacaaaacctttgcaatagcacacatctccccctcccccccaagtctttactagtgggagattaattgcacactagcaataaaaactggtagttatgtattttattaaattaaggatatatatttatttttatcaaggctttataagaatcatgattaatgaaaatagcaaggagagaatatattggcaaagaggtgatacagctacagtatatgaagacttgaaccgtcaacctcagctgatatgcagacgacaaactcaccgcctcaaccagttaggaaacgaagcaagagcgaggtacttgggatccatctcgtctttcccggtgtgttcctctcggagtatgtgtggcctatcttgtggaacggcgttgttagccacgcccaggggaggggaggctccaaggcgcccccgggagggtagtgagacggcactcctcttatagcgggcgtcactgtagtcatcattgagaacaccatagtagagcacattgtcttgggccaggtctctcgtatcatccaggctgcccctcacaccgccgatcgcgcccacactctcgaggtcgtcgttggtgccactcgcatagtcttctcccctcaaatctagacccatatcagacagttgatccgaggttggtggtgacttaacgcggccctcaggatagctggctcttggtcccattacagagttggctgcacgggcgtgagagtaatgactgctacgggtgggcgtgttcagggcgtccagtaagacgtctaggagtgggcgtggggccacaggtgggtgaacgggtacgtaggccctcttgccgtgtgctccaaggcaggaatggccgtagttgaggcaaccacctttcaacactctgtgagctgaggcaggaggggagcagagcagaacgagggggaagacgagcaggagcacagtggaacttctggggcgggtcatagcgcgactctgcaggggggtcctcgaacctgaaacctttctgaggtttagcgaaccaaagatctgcgacattgtctgagtgaacctttgggcaaaaggaaaccttcgagttgacaggatctacgagtgttctagagccttaaaaacaacagaagatttgaaactctcaggggctttggaaaaagcgaagatctttcgaagatatgtgactctagggtcctggagaggatgaagcccggaagagagtgtgaaaaactgagatctctggagatgaggttgttaggtctatcgaagtcttgcgtcttctggtgcgaatggtaaaacctcgaagtctaaaaggttcagaatattcggccctgtcgaactgcaaagctctttaccaaacctctagtgagacgattcttcaccgactctcacttcacctacttcttgtttcctaacctcaagaaagtcttcaggagatctctggtggccgtggcaggttctgacgcgtgagaagcgggactgagacacgtgcgagacccctgcaggtgctgggagtatcgacgacggc**

**G.** Corazonin precursor protein (DS01-Homarus1_Transcript_40929; translation frame 3)

**tgctggtgtagtacacacacacactcgcgccacacgaccaacaacacctgcaaccccacagcatggtgaagatgtacagtcaaacgctggtgatggtagtggtggtggtggtgttcgctgtcaccctagctgctgcccagaccttccagtacagccgaggctggacgaacggaaggaagcgatcagaccctaacgtgggcgtgacggaactgttagctgacccaccccgccgcctgtccgctcactcacaccctcacccacccacacacaccctccccaagaacatcgaagagcgactgagggccctggaggcgggactcaacgccgtcctcaaagccaacagcattaacttttcccccggtggcgacgaagaatactatgccgagaactaattctcactgagtctgggatcccaaataattttaaaatttcaatggaacaatgtgatattgccaatgtctaataattattgtcagtattattattgtagttattactttaacgtacctggtattcattttgtagccgtgaaccacaacccaagaattcatttaagaaactgaagccgtatagtgtatgcagaaccactgagctgaaataagtcaattcttaattgaaatccttaaatatgtctcatttttctcttaatgctcaaaaacataaattattttaagaggaacaggagtgttgtttctgctaggtatctcgacatgattggttgcatggtttcagtcgtcgaattttgtgctggtgatctgagtcttaaaaacaaattataatgtttttatatatcatacagcagcgtctagtggaagcctttactgataactttatgtcttatatgtatgtttattttaacactcctttgggagtttcctaatttaaacagatattatataatttacgtaacgataaatgtgtcttgatcaccttattactacacgttctcttaactaacttactgaaggtatatttcccagaataagacggaaaaagtaggtaacaaatttcagcttaatcgatgaatccgttattgagaagtacacacacacacacacactctctctctcttgtagcatcgcaatcacataaacgatgacaataaccacctctcccaaagctgataataaataatatggcagttataaacaaacaaatattggatgaaaaaa**

**H.** Crustacean cardioactive peptide precursor protein (DS01-Homarus1_Transcript_1513; translation frame 2)

**tgctggtggttgatcaggttcacctcttttgtgttcagttcaagatggaatgtgagaaaaacgatccacaatgactaacatgagctggtgcggacgtgttgggattctgggcgtgacaactgtcctactgttggtactgctggcagcccacgcccaagctggtcctgtcgctaaaagggatatcggtgacttgctggagggtaaagacaagaggcccttctgtaacgccttcacaggttgtggcaagaagaggtcagaccccagcatggaaggcttggcctccagctctgagctcgacgccctggccaaacacgtcctggccgaagccaaactgtgggagcaactccagagcaagatggagatgatgcgcagctatgcgtcccgtatggagaaccacccagtgtaccgaaggaagaggtccacccctcacacccagccccgccagcacctcacctccacacctcaacaaaaggtggaaaccgagaagcagtgaggtgagagaactgtccagtgaggccgcctctacgcacactcgcctcatcactggttgatgtacgcgattcctcactggctgctccactcccccattccccaaacctaattttaattctcaccatcgacgaagagtgtacaaagagacataagcagaattcctgttttaacatgggaattagagaaaaacatatacaggtatttctggtaaagttccgtggttgaacttcgactgactcgttgctaacataattgttattaccgatcaacgtcattccaataaaattgtccagatttcattgaattttatttatctgtctatattctgtctaatataaaggttttgctgttgcattgtatttgtaagtgttttcgaatgcacgtttccgtggtaaagacattgatgtttggtctaagaatgtcatcaggtttgactggtactggaacataatgatgatgccgagggtttagttctcttgtactgtactccatagaaagtttagctttcaactgtgtatctgtgagtcatgaaacagtgctgtatggcttggtgttaaaacgaattagaatcctctgttcctccaaaaaaaaa**

**I1.** Crustacean hyperglycemic hormone I precursor protein (DS01-Homarus1_Transcript_5405; translation frame 1)

**tgcaccaaccccatcatttgtgttacgaggatgtacaaggatatggacactcgtaccttcaaatacgaaaaaaaaaacatgagcttgaacaagagtagtgtaattagatgggaacttggcgtaagaacacctcattgactgagaaggagtcctcaatgacggtgttatatccccggggaaatcctaacgtgagcggcgtgtgacggggcacgagagtcctgctgattataccgacagattgaagtgtgttctactttttacccaggatgaataagatccaatgactcatttgggggatataatttcttttacttaagatgaagtagaactcaagtgagatatttaattaaaacagaacgaaaaagaaaaaataaattgcttttacataaaccctcactacttactacctaacacatcatacgtcagttactcaacggtaatgatgaatggccagcttccggtgatagtagatccccagacttggcgccagatctaccgtgggaatgaacctattaaccttcgaagttaccagaacccgaactcaggaaccaaacccatgcagtttcatcagattgttctaatttccccccaaactgaaggtgtctgaacacggtcaaaactgtatcgaactttcgcccattaccttaactactcaataccctcttatattatcatctgacaagtctcaagttcttgactaatctctcgtcacgttaattcgtagcagaaactatatatagacttctaagacggcctgagtcacggcctgaggcacacatgatgaaaatgtcttcgaaatgtgttgttattttatcatggaatcttaatttttagacatacgagtttctggaaagatctttaaagaataactagctgtctaatgaggaagacaagtggtaaacggggataaggaagaggatgaaagaagccagagaagacacgaataaaaagtagaaagagaggaataaatgaaggaattacaggaaatggaaagtagatcggattcagtatactgtacgtgttgtgagtgagtgagttagtgagtcagtcagccagtcagtaaatcattcagtcattaagtcagtcagtcaatgagaggataagtcagtggtcgagtgagtcaccgagtagagtacatctatgtggacttgactcagcagaattatgaaggctgtggtcgtaagtgcctgtcagttagtcatcgggaggctaagataacttgttttagggagacattgtgttggttgaaggcactagagttcatctgtatgtctgtgtctgtttggttagaaaattatcttttgtaggaatgttttttcgtattatgagatagtagtggtacctctctctctctctctctctttcctacaccaacgtaacttcggtgtctccccctcacagctgtgcttggtggtggtgatggtggcgtccttggggacctctggagtcggtggacggtctgtagaaggagtatcaaggatggagaagcttctctcgtccatctcgccgtcgtctacgcctctaggtttcctgtcgcaggatcacagcgtcaacaaacgtcaggtgttcgaccaggcgtgtaagggcgtgtatgaccgcaacctcttcaagaagctgaaccgtgtgtgtgaggactgttacaacctctaccgcaaacctttcatcgtcaccacctgcaggcagaactgttttgagggcgacacattcccgaggtgtgtgatggaccttggacttgacctggaactgttccttgagttcagggatatgatcaaggggtagactcacactcaagtctctaccttaagtgatcggaggggaggaggaaggtgtacaagatgggagaactgctacagtaaccgaggggtgtgtgatggaccttggacttgacctggaactgttccttgagttcagggatatgatc**

**I2.** Crustacean hyperglycemic hormone II precursor protein (DS01-Homarus1_Transcript_49975; translation frame -2)

**ctgaattctcagtaaaccggtcatttagactaactaggtcttgttaagtccttttagatggtcctccgaggatgaaaaagatgttaccaatagatcctggcaacaaacaccgacctgtcatgttggcgtcgctcccagtggtgaggaaggtggggatattattcttcaggaaattcgattcttgtgtcacttttttccnnnnnnnnnnnnnnntttttttatattatcgtagatttatcaaccgcgcaccaactcagcaagttcgttgtattcttccacatttagcaacaaggcctttacacattgagtgaagtccgtagtgacgaaacagccttcgctgcacttatcctggtagccaggttgcctaaacaggttctcacagtcccagcaaactctgctcaacttgccccagaattctcggtcatagtaacccttacaagctgagtcgaacactgctctcttatttaggctgtggtactgtgacaattggaggtcttcatcaccgtcgattagccaagaacgaccactacacgacgacgtgaagacaagtataaccataaccgttaccaacatttttgtgtacgacagtgtagttgtttgaatggagaacagattgttcatatcttccctctgc**

**I3.** Crustacean hyperglycemic hormone III precursor protein (DS01-Homarus1_Transcript_1478; translation frame -2)

**cctgcaggtggtggcgacgaaaggtttgcggtagaggttgtaacagtcctcacacacacggtccagcttcttgaagaggttgcggtcatacacgcccttacacgcctggtcgaacacctgacgtttgttgacgctgtgatcctgcgacaggaaacctagaggcgtagacgacggcgaattggacgaggagagaagcttctccatccttgatactccttctacagaccgtccaccgactccagaggtccccaaggacgccaccatcaccaccaccaagcacagagttctgcaggcgaacatgattcaggaacgtagagatgagtgtggctttaccggagtctcccgccctttatataccactcnnnnnnnnnnnnnnnnnnnnnnnnnnnnngactcaataattactccaacatccatttaaagataaagggataactcctacaactgagagctcatatcgaaagtgaatcactgtaaatgtaatagaggcgttattgttatcaagaagcgtaataaataacaaggattccgatactagagaacaatggaaaacaaaaatacaccgtggagttaagagttaactggttctaataagagttaagtggctaccagctaggcacttcggttatgcccgttatggactgtacctcgtcagttgcacttattcgtcaggaaccgagtcttcagaacaacgtgtggtcaggtctttacacagaaaccacgttcaggtaatataatcattgttcactatgagattatttcctccactgtgaatgagtcaactctcaccatttttatattatcgcacagctcactaacataaatcacacacgatctcctggtaatgagggatgggtggcactggcatctatcgtcctgcacaaagaggatcagaacgcccgtctcagataaggtgctgcagataggataaagattcgaaaactcttgttatattgtattggtcttcggtaccacaaaacatcctgaaaatcacctcaaaacatttattttggccatgttgaggacgatccatcaggcaatgtttactctccaatcgatattggaggttcagagcgccaacattttgagtaacaacatctggttcccgcaataaactgttgaagatgataaaacaatacgtcatcggtaacaaaagctctgatacagtagatgttttatagtttaaacaaatgtcataatctgctttactttcatatatatttttattatttatcatcaagattacagtaaatgatttcgtttcatatacaagtctaactatgctgtactgtttaactcctctatacaccatacagtacatctctgtgtgtatttgtttcacctaaataaagaacaaactcagagtggataacaaaaaaatacaacgaaataaaaagtacaatgtaatgttacttatgtaatgtacattagtaaataagtaactcgagtaataaacctgacagacttcaccataagtattcatggtatcgtattacccctcagctttatttacagtggttcatcatactggcccagccatatggttcactgttcacttatttacaatggttctgtcctattttacccaactgattatttaaagaccagcttatattatttacaatagtctctgaactattttgtctgcttaaatctaataatatgactatttcaccatttacaatagttcactgcaccatattacttctgtacggtgtcttgcgcactttgtgacatgtggcaactctgtgtttgcttatattgtgtaacactcccaaaatggaccctaaggaaccacgcaataaagaatacttttcattatgtactacaaatatttacatattttaaagctgtgaaaaactgactacagtatgagtaacaatacatgatacaggtacaaaaattatgtactgtataccacccaggtgcctttgtatcttgtactgtaatatgtaactttgttttggatttaatatactgtacttctatatctatataaactaatgtagtcacccacaaaagtcttttggctcggagagagaacttctgtgggcgactgtacattcctgtataagtaatgtaaaatgaataatcaaaatacaaggcaacagacaaggcactgttcattatagacagtacttccctctgtataatgattaaaagtagttcaccttgcattgtaccaaaagacatgcattataacttgtcctaaatctcaaccgctttgaagacgatgtcatcctctgaatacaatgaatcccaatgagtgtgatatcttaactagtgaatacgatattttattcttttgagcatggaaacctaacctcaaatattttaatcatcactgaaaaccctgaaaaaaaatggttgtattttctctaggtcacataaacaatacctcaatagtaagaccttaaggcctaaaacacaacaacagaatctgcaagacaaacaaatcattatgcctctcaaacaacaatcatgacgaggttttgtacacttggtaactccacccaagacctggaagtcactattgtaggtatgttcagtagtacacaggagtagggtagggtattgcttcagtggcggtgtggatcttggtaattgtagctagtgttcagtaaatggccttcggtaaatagtttcacctgagagagaaccttcggctagtgatgttcatcctagtgacgccaatctctcgaagcacgggagaaaatgcctctctaagatcaggaggcagttcacacagatctgaggaggactcgtaagtcttggcctcctctgcttcattagtcatctcgtccaccagcgcctgatggttattacatgaccggagcagccttactctctgaagctctatccgtaactgcatcagctgtcgcgcgaggtgctggtcttgttgcctcatctccatcagctccttcctaagccacgccatagcactctggagattgtccttgcgtttctctggagtgagttcctcttcctcctgctgcccctcctccaccacccttggtgtatgggtctccagcgcctcccccatcttctcccccctctgcttgatcacccctatccagtctaaatatgaaggtcgtcgtgtggcaagcttcaatctagtagcaagatcgcgcactctggacatgtcatcggtgctttcctcaatctctcgctgcttctgcttgaacacgttgtcaatgtcgatgactggacttctcggagtactgggagtgcggggtgatgatcttagttcaacctcctcctcccccccactagtaccactctccccctcctctgagatgtgacgtagcagccgtgacctgtatacctggttaactagtttgttttggggtttggcgaggttcagtttcaagtctggcctattttgcgctttctggttgtcatctgtttccgaggtcagcgagaaatccaagttctcgacgctgtcaactgtcaggtcatggtccctgtcgttggtcatgtcgaggtctctctcagccaacttctccagtttgtcctccaggcgcctctcaaagcagctactgtcgtcagcgtgagccataaactcactcaagtcacactcgctgtcttcctcctcaatgatgtcggtgaccacgtcgaagttggtggtgcttacgccgaaggtagcgtcgttgaggccaaatatcgcccgagagaggccaaagtttgtgttgtttaggtcaatgattgtcttgttttcgtcaaagtttgtgctattaacgccaaagtttgtgctgtgtacgtcaatattcgtgaaattatcgtcgaagttagcaccactcacgccaaagtttatgttattgtctccagagttagctttatttcctaaactcgatgtgatcactggtgagtttctcgtgtcgtctactcgtccactggcgttacacacaccttcaccgctggtaattccgtcactggtcacttcactcactgttacattcggacccgtcactcccaggttcgtattcgagttgattaccccccaaaagcgatcgtttattctcaagtcttcgctgttcacgtcaaacgcacccagctcgctgttgataaagtctgtgagtgagaaggtgtgcgcctggtagtgcggggtgctggtggtggcagccaaggacgtgtgtgtgaagcaagtccgtggcttactactatcaggggggcgcttcccagcatccgttgacatctgatagttatctttgggttctccctccatcggggtttttcctgtcggcgtcgtctctgtcgtgaccttcttggcgaccttcgtgtcatgcttgcctaggaacttgtagttttgcatgaatttgaacgtcggtttggatttcatggtgagtttctgttccattctcggtactacattctccttttcttcctctgacttgagagtcgtcagttccgggtcctctggtagtgagccgttcctcttcaccagctccagctccttcaccaactgtttcctcgacttcctgtgaacatcgggagttttcttgtgcacgtatagaattttgggtgacgtgcaactattcccttcttcatcgtaaaagagtgaaggtacggacgaggattcactctctgtgtgtgtgagaggcgtaaagagatcgctggaggttctgggaaggtggggggcgtgacctgagcgtgtcctggtgggaaggtaaggcggggtgtcctccacggtgctggaagagtcgtagtggctctcacgcactgagccttccaggatactcgtgatggaactctttccgcttgatcccgtgctcctgatgcttggactcgacgatgacgacgacgatgacgacgatgatgatgacga**

**I4.** Crustacean hyperglycemic hormone IV precursor protein (DS01-Homarus1_Transcript_4035; translation frame 3)

**gggagaactgctacagtaaccgagtgttccgtcaatgtctcgacgacctactcatgattgatgtgatcgacgagtacgtctccaacgtccagatggtcggcaagtaaacaaacacctcagccaaccccagacagttcctgctgcgtctaccggtccccatgggcgtccacgaacgtctttttggacaccgacggatgccttcggacttccttggaacgtttctgaaatcctccgcaggtatcaaaatacgcctggggatatccacgggtgctactgaatgtctaaagatttgtgtgggtacctgatgaattgcacaggtatcccaattcttctataagcacctttggacacctatggagctccaggggcgcccatgaagttctcaacgcttgccatgagtagcaggacaccgtctcgtcttcatgggtgggtcatatgaagaccgggaaatgttcatctaccttcataaatctgcagaacaaccgggatataagccaatatttacagcgagacgagcagtgccccgagtgtaatcatcagctcttatcgtcactgtactatcctcctcttcctcgatgccatggcagcttttactggtaatgtttttttcattaatgcgtatataacttatgtaccttaaaatgtaatgtaagtatttatttgagggagataatgtagtggttggtgcagctgggacaagggtttgaagcctcgagtctcccacagtccgacatttcattgctagtcataactattagtggagatatttttatacgatacgagggatgtgtggcgttagttaggcactcctgagagtcgtcagtacctcacacccaacacacatccttccttgccaacacaaacacttaactaacacatcacatcacatcattaacacaagataaagtagaataatcctcttacttcagttccttcttcagtagtgacgtttaaacaagtctttaactatcacgtctacagggagtaaatatcacggtagctaatgaactgtagagaatccctgaactgattgtacatacatagacaagttccctccacttacacacacgcgcacgcactcacaaacacacatacacttacaaatacacacacacacacacacacacacacacacaccaaacacacaatacagttctcggagtgaaatattttgttatataagaatgagtcagttgtgagttaaatcaaatctattttttgtgacgccatgtgaaagtagcgacataaagaacaaatgttagataaggtagaggagagtgaaggagttacctgtaacccccatcaccctcctctaccttatctaacatttgttctttatgtcgctactttcacatggcgtcacaaaaaatagatttgat**

**J.** Diuretic hormone 31 precursor protein (DS01-Homarus1_Transcript_2312; translation frame 1)

**gccacttgtgactcgctaccacctgggactcctcctacacgggcgtcgcgtcagtcaggtgaagactctcagctacagaccacagtcccccgccacttgtgactcgctaccactactacccgcactacccgccccccgcacggatacccgccagtgattccacctggtgctgctcccgcctctttctttatccagacaacgacggtcaaccgtttgagcctgatgaacagcacgggggcagtgttcgtttctttggtggtcgccttcatcttcgtgtcttcagtcaactccgccgccttcaatagagaggcgcgcgcggtggtgcagatagaggacccggactacgtgttggagttgctgacgagattggggcactccatcataagggccaatgagctagaaaannnnnnnnnngctaagcgaggattggatcttggtctgggccgtggcttcagcgggtcacaggcagccaagcacctcatgggtctggccgccgccaacttcgcgggcggccccggcaggaggaggagaagctctgacgatggccttgacctccaccacgacgacaacctctacgcccaagatcaggctgctgacctggccgagtcatcacgataaatttttgtggatgactaccatgtgacaacttacctcctctgtcctctctcacaatgactccctctcctctcggttgcttttcgtctgctcactgccgttcttctctcaatagttctctcatatgctctcacctcgtgtcattctcttgatttttctcgacataagagaataaccccatcttcacatcttgtggtggacccaacatcagtgacgacaataaaatccgagcattgtcaaccggctccctgaggacggtgggccccgcctggtggccgccgcagcaccgacacctcacacgtaactcctgctgtagtccaccaccaaggtgtttataaccttgttttcctcagtctgtaactgtagtaagattgcatatatgatacagtagacccttcatctattagaaatgatgtaagttaagcttcatcacacagcctctgtcagaagggaggaagatgttgcagcttgtaattaaatagcatgagatacaatgttctctggaagggagttgtgtagtggtttcattgtcatattccagaactttttttccaggtgcttttacattgaccctccggcttcaaatacatagcttgacttgtatagcacaacctgcttccattattttcctcagtggttgaagactataaagatgtttaatgttggattaatacctaggtgttcaggtactatttaccagaggcttgactaccccccacactgattttattcatagacaagtgtcaggaaaatgttcacgaatgtgtacttaatccctggccaatgatattagaagaagccattcacaaatcgagcctacgagcaggtctgtggataagcatttctgatgtcattaaccagagatgaaaccttgggattggtgaaaaacgtttccaaccttcccgtcctgggccgagtagcatcccagcgagcagacgactggttatgttcacttgtgacgttgattttacagtgtatgaaactcatccattcaaggcaatacataatattttgtgctgcatgctttaatgaattcagactgatggatcatattcatcagactttctcactgaaacataatgtgctgtagctgctccgagtatggacagtacaatattcaggagaccatcttgttaactgtctctccatacttaccttctctcttcagcttcattttcattacaagtttataagtttcctgttaagtgacatggtggtagatctcctgtgctgggggtctcttttatcactggtgagcggaggcgagcaacaagcatgagagtgtgacagcaggatcctcccaccccacctactcttctatgggctggaacttgtctcaataagttaagacacagacaaatatttgtgcgtatataacaatacctttggttcaccttaatgtagataaaataaagcgtgtcagtccctgcatttatttctgtatacaaaacctttagaatggatgagtatgtaaataggttgtggccatgaggccgtggggcataatgtgaccacaatctgaagcatactaccagactcaacgtgacccgtgacgatcaccacacaggcttcgatctttcatgtgtcgatcaccaagcagacacaaaccatacactatcacaaaggtagacttgccacaaaccagaaataattggacttctattttcttctgtactactcaactcattattaaacacaattgatgaaaataaatatgtccggtaaattactttaagatacctgtagtagctgtgtctttagttttcaggcataatcatcaatgttggagataataaaataccactcttacccaaatctaaaataaacagtttatacaaattggtgtacaaataataatacttgtacagtactgatgtaaatgttagaaaatgtataaaaatcaaagctggaaatgacatgttgtctcaagaatgaaaacacagctacaaactgacacgtgttaggtactgggatttgacaattctacaagattcctgcaacaaaaatcaactgaaagaaaactcaacccaggttctcaaagatttgtataaagaatattcctcatagctgaatatgtaagaatgctgctaactgattttacttataccgaaacataaaatcagcatgcaaaaatatcaaaatcataccaataatctttaccaaaagaacctttaccttgactgtctaaagtgaatgtttctcatagaactctcaatataatttgatgattacatcttcagtcacaagtaatattcatcaagtcaacagcaggtgaactacag**

**K.** Diuretic hormone 44 precursor protein (DS01-Homarus1_Transcript_7828; translation frame 1)

**gtgatggtgttattccgggcggctatgttgggtgtgatgggcttgttccctcttgcctggtgtttgtccctgggtggcggacgggcggatacctcctccctcctctctctgcctcacccccaggagctgtcccaggatgacctacaacccttcctctcacgccaggggaacaccgactctgctggtgctccttcgtctgttgctgattatactgggtacgacaagagcgaagttctacgcgggctagaggacccaacctcctcttctgcctatcgtctgcaggaggcgttgtctgaagcagtagcagcagcagcggcagcagcagaaggagcagaaggtgttcgtgacggtgcagctgccttgtctcctactgctaacgaaggagtgactctggaggacttagttccttatgatcctgggtactacctctaccctgcctttctcaaccgtggtgatgaggccatgacggggggcagcagcggcatcaacagcctcaggaaggtcaggaacagcaacaggagcaacaggagcaacagcagcagcggcatcagcggcagcaacaccagcagcaacagcaacacgaacaacaacagccccgacactatcagtatggccaagaggacctggccgaatggtttctcccgacgaagggcatccgggctctccttgtcgatcgatgcgtccatgaaggtgctgcgggaggcgctctatatggagatcatccgcaagaagcaacgacagcagatgcagagagcccagcacaaccaaaagttattaaacagcattggcaagcgagacgtgacgaggcagctccaacaggaaggaatccagggtgtttaccagcgaggccagaggaagtaaagatgaggatcagcagtagtggacagtggaggtgtgtgagtgtgtgtgtgtgtgtgctcggcctcatgaagagcaccaaccagctaacaggggacacaggcagcgataatgatgctttacgatggtctgctgcgatgcccccttcccctacggccctgctgcgatgtcctcctcacggccctgctg**

**L1.** Eclosion hormone I precursor protein (DS01-Homarus1_Transcript_36484; translation frame 3)

**tcgctaacacgctctacagaatggttggctccagaaaggtcgttgtgtcggtcctgctggtgctgagcgtgatgctgatggcgttgctgctgcttccgtctgccgccgccgctgccaacaaggtctccgtctgtatcaagaactgtgcccagtgtaagatcatgtaccacgaccacttcaagggcggcctctgcgccgacttgtgtgtccagtccggaggcaaattcatcccggattgcggtcgacctcagaccctcatccccttcttcctgcagaggctggagtgaacatgaaggtgctccatatgaagacttggaaatgctcagttcatcccctcctccctcctgagtatcatgactcacagttcttcagtgtcgtggctcaagccaaaatttggcagtatttaatgctcaagacaatatctggaagtctcctgtgcactgtgcatatatttagaatttctcattgttgttgtctaagatgagacttgacttagcatttgtgcttaaatattatcctgttgaagcactaggtatacataaaaacatatttcttcggtcaagatatggaggtaattagtacttcatgtcgagatccggaggtctcaatgttttcaattaaggcctggaagctttcagtatcttgagtaaagcctagaaggccctaagtacctttagtcgaggagtggaggatcttggtccctagattcaagacctggagtttccgaatcaagatccgggagcctcaggattctgaatgaagatctaaaggttctcaatatcttgaataaaaagttagggatcctcggctttccatacaaggaccaggaagtcttcaatacttgagttagtacctgaaaagtcgtcagttttccgagaagcgaaagtgacaaattcattatcagtgttattgttgttgtttctctgggatttgatatgaaatggccgctaggccaagcagttggcgaccagtaccgg**

**L2.** Eclosion hormone II precursor protein (DS01-Homarus1_Transcript_14893; translation frame 1)

**attcttgcttcaccccgggtcaacaacacacggagttccatctgcccacctggcatcgcagcccctctcgtcaaattcttgttgacaatacaatatttctccatgtctttcaagcgtgaggtggtggtggtggtgatgactgtggtggtcttgatgactctggcgacgctatccgatgcggcaaccttcaccagtatgtgcatcaggaactgtgggcagtgtaaggagatgtatggcgactacttccatggccaggcgtgtgctgagtcgtgcatcatgacgcagggcatcagcatcccggactgtaacaaccctgccaccttcaaccgcttcctcaagaggttcatctaaagctaagccaccccaagggtcccccccaaccccccaggagcctccagggttctcaataatcacagacaagttatttatattttaccttgtgaagtaagggtcaaagacttgctgcgtgtgaataatcacttcaataggatatattttagttttctttgagggggtatgtttccttgtctgctcatggcttgtccagtcgtctcatcaagtactgggccgagctgagcagaatgtagaacggtgtcctctttctgttgtcctttttattgttcttcgtcacgtcaagggttctgttggttccttttgatcttctaacaatgtcatggttctgttgacatcttgcatttatttctgattacttttttcttgttccgacttgagcattccgtttgtactcctcagcagtaaactcatatttgctgccgatattcacctgccatgtaattaatctcagtaatgattcattaatttgttgttagtctctgagtcgtgtttattatgtgccacaatatttattgtattattgtaagtcagtgtaagaatcaaatcaaaacatctatttatcttgcacagttttttcacctgttctctacacaaagaatttccctggactcatctctctcttaagtttcacaagttacatttttatatacctgtattttaaaaacgtatctagtgctctgtttaaagatctcacaaatctacttcttatcatcttacaggtgaaactttccgtccccgccagaggacataagtattcttacctgtgtactcacaaaacagggaagtcacactgatacactgttacactggctcacacgacacacgttgtaggcttctgtctcaaacagaacttgatataatctagcacgaaggaaacaaatattataagaatgaaccctttctgaaatgttaacatctagattgaacttcatttctagtcggtcagtagttttcattgatgtgtgtgtatgtgtgtgggtccagacgttgttggtgactagtcacgtcatcagcaggactactgacgtgtagattctcataatccttaattagcttatataaaaatatttccacttgccataaacacactaacagctgatctctgagaactatcgtgagggcgacagtaacattgtactcattatttatttaccaaagtgatgcatattaagctttcctcagtattatttttataagatatgtgacagattatatattaacacactgttaagatagttatatttatttttatactgacgctacctttagttaagttgttcacccctcaagctactgagttcacttagccttagtattacctaccaagacacaattgtttcgacactgatgaacatcttgaacagtgctaaaaaaaaggaatgttcctccaatgtctgtctgtcttaccaattttgtcataagccacaagatgataataaccccacatccctacgtcactgtagaccgtcagtgttcacctctgtgtggcagcgtcatcagctccctctatatccctcctgcaattaataccacttgaatgtattttccccgcctttcatatttttcataaccttcccttaggtcaatgttacttctgaatgagctgcataatttttgactctatagtgaaggctggtcactacgtttagtataaagagaactgatgaggagattgtttacataatgaaccactgatggtgttaactgtaagtgtcgtgaggggtagaccagatgacacactctgacacatgtatattacgagtttatatgcttcttctcttaatggctgcactctgagagcaaatttagctgaagaaaaaatattattatgggaaaaaacagtgttgagagaacaaggggctcctgtacactttaaaatataataaataatat**

**M.** FLRFamide precursor protein (DS01-Homarus1_Transcript_3291; translation frame -2)

**tttttttgagaaataaacatttatttcaacaaactgcagggttgactctatatgtcgcgccaagaatggtgttaactgagtccccgcttctgttttacttcaatagtatcagtcatctctctaatgaaactccaacaactattaccacataaaatatataaaaaaacggggtcattgcttcattgttttctcctagtttcaagttatataaatactcgtacctaggaaaattaatgttactgcgtcttcaaattttcatctcagctttaaaaatatcagaaaacgggtaactgcttaattttcacctcannnnnnnnnnnnnnttacggtttaatatgtctcagccagtgtgtagtattgtgacactgacaagagggagccacaactgctctctcacgccctcacacacggtacgggtctcctgctaacacgggtataacgctcactcatgacattcgagttggcgggcactcgatccctcctcggtgtctcaaaggacaaatttccaacaaaatagtttatgattatcgcatttttgagcgtgtcggaatgtaatgttttgatcagatcggctttttttttatcaaggatttccactcatcaatttgcaggcttgtatcgtcgaccaacagctagtcttcttgcaaccgtggactattgtgattggcaaatgaaatacagaacctgatcaaaaattccttcttccgaacacacccaactgcctaatgatatataacaccagccatgtacacttaataataacaataatgatgtattcactacttgacgagtatttgaagtaattgccaacttgcaatacggatatgtagtaatgatatactgtactgtacccggtagaggcgtaacagggattaaggcaatatgattaaaaacaattcaaacagtagcaacaggtcatttattgactagatttaaatatcactcggttgaagtatctagaagtaataatggaacagaggtaaaaaaataaactatatgatgatcttcacaaagatatccagcctttgtcagaactaaagcacctccaaggtgtaacactatactgtactagggtcaagtaaataaccttagaactcttagtgataacacaacgttcgtatgtctttagaacttagttaacacaagtaaaaaaatagttatttacatatgtaacttaaatataagtaaaatatacatctatacatacaactaaagtagttttataagtaaaaatatatatttgtacatataactaaagtaatttcataagtagaaatatatattcctacatataggttaaataatgttctaagtaaaaatatatttcttataggtagcaacaccccacactcgagtgcctgctaacttgccctgtccgaggctcacaggagtttatgtacaatgttcacacagacacactacttgtacactcagagacacagtaatccatttatctctatatgttatcagcgctgttactgcatgtagggttgatgggtatgtagttatcatctgcatatatatatagatcaccaactttaatgctttatttcctggtctgactaacacagtttactgattaatatagatacagtatataaattggctgaacttccaaatcactgagcttttgacgttgatctcccataacttgtgtacattattatagtgtaaatggagaggatggggaggaatcattcgtcattatcgaaaaagataataatggtagcaaacattgaggcaggttacactagggtatacatgggtattgtcgtaaggtccagaggatcactagggggtaggagaggtctgaggtaagggtggaagtcatcatcatcttctccagttcaaccgaacctgaggaagtttttggaaggcgccctcacatagcgggggtactcgacgggcctcaccactaccactggtgcgggagactcagcagaggaggaggaagaggggtagtcatcagagccatctcgcttgccgaatctgaggaagttacgatcatggctgaagcgtttggctacttgtggttcctcgaaggatgtgacgtcgatgccgtcctcctcgggctgtagttcactggcccaggccatagggatgtagaagttttgggcggcggctctcttgatgcggtgagcattaatttgcctcaacacagaggactcgatgctgtcgtcgctgaggactgcagacacgtcacgcttggtgcgggccagaggctggatggtggtgggagagggagtggaggtgaattcgcgggctttctgttcctcgtcacaatcttcacaggagagggagctcagctgacggtcgacggagcggccaaatctaaggaagttgcggtcaccacggccaaacctcaagaagttcctgtttcccctgccgaagcgaaggtagttcttatgggcgcctctcttttcttctacaggtagttctacatcctcctgaaggtcggtggcgaattccatgggtgatcctgaacgtccaaacctgaggaagttgcggttctggtctctgccgaagcggaggaagttgcggtttcgcttttcaggagactcgggcatctcttctccttcgtagtcgttggtgtctgatcgaccgaatctgaggaagttacggccgctcctcttgctgttatcatcactacgaccgaacctgagatagttcctgtcactatagccacgcttggtgccttcctggccgatggggtaagcgtcaccaccccatgcttggctggcggggaggaagtacttcaataatcttttttctgggagggcgaacaggtcatcttcttggctctgggcgggtaagagggcatcagtgggtgggtcgagggcggcaaccacgggcggcacgggggctgcgtgggcttggcaacaccaagtgagggtggtaaggagaacccacgccgctacaatcatgtctgaggatcccagtttggagatggaggggaggttaagatccgtggtggtgtatgtgtgtctgggagcgagcaagtggtaggtgctcgctggagtgatggtagcaagggagtgcgactctcttcga**

**N.** GSEFLamide precursor protein (DS01-Homarus1_Transcript_30037; translation frame -2)

**aatcccagcacccagaggtgaaaattgtttacattgatttacttgtgtgaattataggttctgatataatgacaagaccgttgagctggaattttgattaaatgctctctgggacctgtgtgtgggcagcccatcagaaccacaatattatgtgggcatgggaccttgagctacaggtgagtagctaaggatgggtagctttttgcccattgtggtcctgtggagagatgccttactgcagacattggtggttcaggtatgaatcccgttacctagaagtgattttttttactatgttcagtgtatatagatgataaatggtgagtgcgtctcataaaatcagttcgttgacaaacaaagaagttttgtaacatttctgtatttttgttgtaagtggaaggacgctaaaataattttggaaaggttgtggcgggaggaaattagattttaatttaaagagagcagaaaactgccaccactcgccttagtgctcaacaagagccaggagctgctcagccccttctaccatcctcctcattaacaactgatggccacttagaatcaaaacagttgacctctctgggcatccctatccagccagtcacaaaccaacatgagggagtgtggctgagactcactgtattttacatttccaccatcaattggctgttattcagagtgttacatttttaacatgaagctgctggtctagtttccaacgtgattgtgaatgtgaaacttccctcttacgattttgttatctagggactttctttcgcttcattaatagttaatattttgaaattgatgtttttcattttataaagaaaagtgtttgaaatgtttaagaataaattcaaaatattatggcagtatttcgaagatgaagatgaagaacaagggacagtagtatgggagatctggcggtaaataatccatttaaaaaatgaaaattaatatattttaaacatttacattctaatactattacaggaagcttaagattttaataaaaaatatacattggtttattataattttggttagggataagtgctacagatttatattttttcccagtgttaggaaggtatcactcgccatccctcctccgtatgccgagtgagctacgtaacacagggatcatttgtataagggaactactgtagtaataagctaccctagccgtgacgataccttgacataagatagcccaataatttacagtgaacagaggtcagaattgttcccctacagatggaccggaaccgcttcctaggtcttcaaaagacacctcgtatcacttgaaattaaaatccttcaaccaaggaactccgaacctaccgctcgttttgtgtcgtagtctagcgtatgggcaaattcaggttcatattgtcgctttcccaaaaattccgatcccatcgctctc**

**O.** Insulin-like peptide precursor protein (DS01-Homarus1_Transcript_51972; translation frame 1)

**gggcacggataagtataacctcctcgtgtttcgggtaagacattagacgccagacaattagagaggaggacttgtctatctaaccgaccgtctataagccagccagaaacccgcccagtgatgagagcttttgtagtggtgatagcggtggtggtggtggtggtgctggagttggggtcatcaagggcgtctcgaaggacctacccgacgagtgaggaggagccccgtcggcgcctatgcgggtggaggctggccaacaagctcaatctggtgtgtaaaggcgtgtataacaacccagggtctaccggtaactacctcttctacaggagcagacgagacggggagagtgagccggggctgccgccagagaagtacctggacctgttggcagacccagaggaggagagagggctgaggcaccattacttaacctcctcacaacaagcctctgaggacacccccagtgaggaaaatgaagcacctgggagcttctttgggtctctctctcctcaggacttgcctcaccaatctgccgtacaagaagatgaagcctcgagtgtccacttcccatttctgacagaggaggaggcgtctcagatggtcagggtgaggccgcggtccaaaagaggattgtcggctgagtgttgccggaaggtgtgcacagtgtcggagctggtgggctactgttactgaccttcaccctcacagtgctcagttcatgacgggtatcatggtgaatctagtcatagaattaatagcctaaactcttttgtgtcctagtaaaatatttatgacatttatgaagcggaaaacttgatttaacacttggcgacttgtgtagaaaagagcaacggtatggtgaagtcatcaacgaatagaagtgaatcccataaaatagttgtctgacatacacaacagacaatattataatgtgtaacttatttcttagtgttgtggaatatttaatggtacatgaaacactacaggagtacttataac**

**P.** Intocin precursor protein (DS01-Homarus1_Transcript_45955; translation frame 1)

**ccttctctcagccaccacaccaaagatccctcctctcagtgactgcccacccgtcaaccaacagatatgcagttgggtgtggttgtcgtggttatgaccgttgtagtgggcagtaccacagcctgcttcatcaccaactgtccaccaggaggaaagaggtcgggtcccacagcacagcttggccgcacccgcacatgcacggcttgcgggcctggactgcagggaaggtgcctggggccagagatatgctgcgttttaggaattggttgcttcctgggaacgagggaagccaggatgtgccacgctgagaacctagtgcctgttacctgcgccaaccgggatctcaagtcctgtggcaggatgcaggagggccgctgtgccgccgccggactctgctgcaccgaaatgaagtgtgaattcgacagcagctgcacggtggagggtcgggaggagagagttgggaagcagcgggcggagagacaacaccttaccttcctgtccagtttgcccgaagaccagtggaacctgtgagcatgtcctccctctccctggctgctacatcgtcctcggcctggtgatggcagcaccaacacatacctcacatgagtgatatgttgtatgtggtgacctcctccgattcatattaccaaatttataattgaaagttaactttgttccactaatggaattatgaacatctactgcatgtacgtaacccgatctttagtacttatgtaaactaacaatctacgacatcaaacacttagacatggtggctgtgtcaaagagttggatctctgtttaaaaaggaaaaggttaaccttgtcataaatgctccctccccttgagggtgtcattttatcactgaataaagtgtaccct**

**Q1.** Leucokinin I precursor protein (DS01-Homarus1_Transcript_36199; translation frame 2)

**agtaaggtggtcgggtatatatagggatgttggtgggggtcgaggttcttgtcctgacccatagtactcgtgtaaggtcgctcgtgacaacatcgtccctccaagcgacctctcactaccacataccaacatcacggtagtgatggtaacagttgggagatgggtaagtctgtgggtacgtctggcagtggcgttgagcagcggggccgcctcagtatccttcgtgacgtcggaggtgatggacgtctcgcccctcgctctccctcatggtcgacaccctaatttatgtacccctgatcacgtaccctcacatcctatcatacggtgtgaggtgggcaagagacaagccttccatccctggggcggaaagaggtcctcattcaaggctgcacctggcctgcctctcagcctacgtgaagtgtatcttgccttgtttcagaatgccaggcctcgtccaccaccaccctcagaaggggagctaaagcgagcgtcgttcaacccctggggaggcaagcgctcagaccccctccttcctgccagccaacacgaacccaacaccaagaggaacactttcgctccctggggtggtaaacgtgcagcgggatacttcacgcacgataccaacccacttatcatcgaggaggatctgattccctacatcggcgtgctgagtgatgacggggaagccgaggatgtcgtcaagagggaatccttttccgcttggggaggaaaacgcggatccttcccggcagatgattgggaagaggaggaaccaacggacttgtttgtgttggatggtagtttgccttatcctccagtggaccgtctcagatacaagagagaggccgagacatacactaacaccctggtaaacagtgatgactcaggcgtgaagaccgaggcggaaaacattaaaccagataccaaatacgaccagacggctgaagcatccagcactacagtagcgaagaggacaagattcagcgcgtgggccggtaagaggccggacctccaagttatcgaggacgcggtgagaaagatggccgagcacgaacccaagacgacggagagaagagctttcagcgcctgggccgggaagagatcgtcagatgttaacctccaagacggtgaggatgatgagccagtcagtgcctggattggacgaagactgcaagatgccaacacagacgacaagagaacattcagcgcttgggcagggaagaggtcaccaagtatggacctttcaggtaaccaagacaaaagaacattcagagcctgggcagggaagaggtcgtcaggtgatgagctggatgatcacttcctggataagaagacaagatttagtccttgggcgggaaaacgggcggaaggcacattatctcgcctgtctgagtccactctgaaagccgcccttgatgagaattccccagaagacaacgttgataaccacaagagaccttccttcagtgcctgggccggtaagcgctctgagagtaatgaaaaacgaccatcgttcaatgcctgg**

**Q2.** Leucokinin II precursor protein (DS01-Homarus1_Transcript_31470; translation frame 2)

**cgatgagaagcgtccatctttcagtgcatgggcgggcaagagaagcagcgaaacagacaagcgacaaggtttcagcgcatgggcgggcaagaggaacaacggtggcagtgacgaccccacacattcaaacaatccccaacagatctcctccatcctccaacaactccaacaccaaggtctagaattcctccacaaaagacttccaaataacgactggggtaataagcgagtccccttcagcacatgggggggcaagagagcaagtccaatctccgaggatagtcaactaagcgatctatatacctcacaactttagtatctgtgcgagactcttaaccacctgttacacgttggcgttactgtattaatcctaacataatccctctaataatcaactttgttttgatattattcagaaactgtggatgttacataaacactgcaatcacggcttaggctgcgagaaaactccacggttgcagtaactcgatgcaccataatacactataaatgttggaaaaataatactcacggcttagggtcgaagaaaatgccctgtttgcggtaactcgtcggactataataatcttcggtgcaaataatctctcaaaatggaattaccgcaaacacgggacttttttcctaggcctcgtatcaacggctctcaggcaccgctggataacgttactaaatcctacgcaatactcaaacacggtcgcccttacaattattccttgataaaaaatatatataaaataacctccatcaagataacaatataaaataacttatatttctataaaaacttgaaggcaggaagtaatattatcattctctcttatacagttggccttcatcactctgtacggcatctagtaaaacaatattctgttatacattatacactgtgtccaataataaaacactcaacattatctataaatatacaatcttattcattttaaactactgtatatgtaagaataaacacacaacttacacaaagacagacattgaannnnnnnnnnnnnnnnnnncttatttcacctgatgattatacaccttgatgctaacttaaccttatctaacacaagataacatataaataaccaaacctaatcaaacctaacttaaacctaatctaccctaatgtaaccagacctaacataacctaatcccaggtagtgttagtgtatctaattatcaggtgaaataagtttgggggagcgaatcttcaggtaatccacttaacctatcatcattcatacagggttaagacacagatacgtatgtacagtatggaaacaacgttgttacatacccttgttaactgcacactaagcgggtctattgaaacactgcagtacacataatcagctgacatcataacacacatggggcgaaacatccactatcgcaacacaaagtactccagaaaactcgcatagtgtattgacagcatattacacagagtgaaacaaccattatcgcaccatgtaatacaacagaaagctctcgcatagtgggaaccaagtgtagaattatatgtctacgattcattcatgttaacacagggagcgaaacaccacacattacaacagaaccctccatagtgtgaaccaagtgtaaaattaaccttccgtcacccatcctaacacaaacacacttccctatttaccccgaattattttttacgaccatttccctcctctaagtctatgctnnnnnnnnnnnnnnnnnnnnnnnnnnnnnnnngactaaatctcgtgactgccggagagttcacacaaacatgccccaataaacaagaggagaaagacgacgttgaatctcgttaattattcacgcggtagaagttattcattccttaaagcaccaatccattcactgtataatgaaggtcgcgatagagaacagcaacagaccaaccaacgatctctgcagtgaataatgaaagtcgtaacacaataacaaaaacagatcgaccgcctgattggttagtaatatgacacacggcgagaatgataaacaaaacactcaaccaatatacagtgagaggaagtggtatgccatcaactttcataagacaaaaataatacatattcttatttattacttatttctccttatttctactcatattgtattagtattccttattgtttctccatttcgttcgggtttctgtatcacctgatcgaccccacacagcgatggacgagttaaggagattgtcgttagtttcatgtcattttaccatctccaacgcacagcaatggtcaaatcaagatggctgtcagatgtgaagttttattttatttcatggctgcagcactcaacaatgcacgagtaaacacgactgtcatttgtttatttaccggctgcttcgttattcctcctgtaaggctggatgggaggaatgacgtcagcatcctccagtacattagatcatctttgaaaatgcccttaaattatgcaatttgattctaatacgtcagattgtattctaatatagtctcaaatatgttctactgcatcctaatatagcttactgtattttaatatgacctcaaatatgtccgaatgtattataatatgtccaattgtatttcaatatggtttcaaaaatgtccgattatcataatatggcctcaaataagaccaactgtattataaaatggtctcaaatatgtccgactgtatcttaaac**

**R.** Myosuppressin precursor protein (DS01-Homarus1_Transcript_4887; translation frame -3)

**ccgatctttttttttgtatatgtgttaatgaaactaattttcctttttggcactgagtacaatagttgttgacaatattacctaacataacctgaagcacataataagtggctgcccgcacacaggcaaactaccgtcacagctatggatcctctgtgacgacgggcatatgtacacaaaccgtctcataccagacaacatttataaaatataagcgtataagttacagaattgctcttaggactgtcgaagccatagtcgtcacttcagtgtcgacacatcagccaggaggtgttgacatatgcctgacgaaggaaagctcgtcgagtctccgaaaaaaaaaagtattctccatctcaggtggacgattctgtgacaggtgaaggacgtgagaagtgcctgtggcaaagctgggtacccggcacagcttcatgccgtctcttatccccaaagagaaaaatccacctgctcagagaagacctatatttctccttaccacagcctaagacgccgaggtaacgcaatgtaagaaccaaggtatctgagtaatgtctgacacataggggacaggcgagattgatcaacataccattcggttcatttaatggttaaaggaaggcgtggccagcagtaagaccacaggaagaggccaggtcatgtttattgttctctaacaggtgaatcatatgagcctcaccgggctcttctgggactgggaatcccaacatcaacacgatggagcgtggcacattacactctcttgttacatagtgaggattaggggagattcagttttatttgggatattttaagataagattcgcgcatatatttctacattagataaaaaacataatttatgtatataatgtactgtatatagatttttatttaacaacagaaaatatattaattatttcgtaccatatttaataaaactgaacacaaagaaggtaactcttatgcaacaatgccagccatttttggtctataaaagaaatgggcatcttaaaccactagaaacacatgataacataagccgtatagaagcgcgtcaccgaaggctaaaaacgattggcaaagtaacatgtttttttctttttcattctcggcgaaaattatgccagattttcttccttccatgatccctgctgacttattgctgggatcgtccgaagcggaggaagacatggtccaagtcttgtcgcttgacctcgggctcgttcacgggcatactgcgctcgatagcttgagcacctagatactcctccattgctcgggagaactcggagatgttgatgagggcggagcacagtttcttagcgaaggggcttaggggcacctgctggctgaggcagatgggcgggggcatggtctcacccacgcccacacacacgcccatcaccaccaccacgccgaccaccagaaggcaagaccacgaacagctgcggaacaccattgtttgtgtgtagaggtagggctggctcggctagactggcgggagaagagcctgttgcgtcagctgtgagggacgagggcgcggggtgtcgcctgttgcttgtgcttgggcagagcctcagtaactcacgttcgtctacaagggatggaggtaaccaagtcggttgcgtcagctgtgagggacgagggcgcggg**

**S.** Neuroparsin precursor protein (DS01-Homarus1_Transcript_14196; translation frame -2)

**cgatctccgcattaaaaattagaaattgtcgctatttcgcacttatccctgtttagtgtgacaaagtgggagagagagacagaggacagggtgacccagggtaccatgcaaggtgtgtgccagcgatgggtgccaccgaaaagaaggggtttaagttgaaagaatacacaacacttatgagcagcgtgtgatgatccagctaacagtagctgccgaaccaacattctagattcgcggagcagccgttacagtagccgcaggagcagtaagtgccggccccacactcaccgttctccgaccactcacccccacacgcctcacccgggcccttggcacacacggagccaccgcaccagtccaccacagtgccgtacttgcagttgttggctggtaatctgttgcctccttggttgcatcttggtgctgcaccagtctcattgacgatgacgatgaccacaataacggcgattgaggtgacgaagcccagtgatctcatgatggtggtggtggtggtgacggagtctgtgatactctgaagtggagtggtagtggtgaccttagtggttgtggtggttgtgatggttatttatgatgacgtagatggtgagtgagaggtgagaaagagggatttgaaaggtgagagatagattgactcagccacagcctcgtcgtacttgttcagtgaccagaaaggactcggccaagtacgacgaggctgtggctga**

**T.** Neuropeptide F precursor protein (DS01-Homarus1_Transcript_7995; translation frame 1)

**agcactctcgccgcgtctctggcccgagcagacctgcatttacacacacaaacacaggctatgcgaggtgctgttatggtaggggcagtggcggcggtcatggtggcagctctggtggctgggatggcctcagctgctagacccgacaactcggcggctgacacactgcaggccatccacgaggcggccatggcgggtatcctaggatcagcagaagtgcagtaccctaacaggcccagcatgttcaagtcacccgtggagctgcgacaatacctggatgctcttaacgcttactacgccatcgccgggagaccaaggtttgggaagcgagggaaccacggagctcaacgtacggaggagctctatgactactgacccactagtcttacactcatcccttccacccacacccccgtcatacttacaacctctgataaccaacaacctacgacccttgataggcaatctatctgacaacctacaaccctcgccgaccaaatcaccatacagcccacatcaccagttaatcaatctcttgtacaacccacaactcttaacttgtctctccaataacctcacaacctctgacatccatcatttcacaacacaacaatcccttcctgctgcgacccacgacctctgattcccggctcttcatgcactcaacacctgaagctcttcagcaaccaccagccagtaagatgtctcccacacccctctctactgctgcttacaaaaacacatccacttcaatgaacaagtggagctctttcccacttgcaacatgagagaaacactcatgagagttggacatagaatttgagtgtgatattgatgttaatgatgatgaagattttaattcgtgtccgccaaagatggtaacaagtctgcacacaagcgcgttggtctcgtccgccggcctcaacaaggaatattaagtctcgtcaagtgatatcgctagaagtacagtccaacctgtcccctgctggtctccctgtgtatgtgtactaacaaggtcttgccacaaaacagcaataggcacttcacactcacccctggtctccgtctatcattcaacaagtaagcagtttcacgactaattgaaaagaagtaatttcttcgaccaagatcatacgtgttaccgaagacatctcaggcaccaaaagaggcttctacctagggagttccatcgtcacaagttccctcaagaaatcattttaatgagctcttcttggttcgtccagaatatctcagaggctgaaatcatacgatttatctacattagttacccccccccccccccaagctatgtactaggagagtgagcaaataactaatattttattttgcagtctatttacacactgtatgttgccaaatattatgtactgagagaaaaaaatatatgagaaaaatagtcatttgaaatttcttatcattctttctataccctgacgaacattacac**

**U.** Orcokinin precursor protein (DS01-Homarus1_Transcript_1885; translation frame -3)

**tttttaacagttaacttgtataactttattattttaacttgtatgacaaactgtgcagtaagtcatttctctacaacatagtctcacaaaaagtttttaaagaattcatatatagagtgatattgcattcagtatacctctgggaccgtcaacattaatatatattctgaaacagccctgaaaacacaacattcactttgctacaacttgttttgttgatattgtgtgatccaacgggaacttctacttcagcactgggccaatacagtccttgtctgtgtggtagggaacacttaatactgtgtcgtcacttaactaaacgatcagctgtagacagtaagtggttatatgaagcggctggctgcctttcagacctcagagattcattccaaaaaaacccgtttagaaatacttaagcttatgagtctaagtaaccagaggaactcacaaagacaaatgtaaacaataaacaacccaaatgtaaaagaaagaccacttaataaaaatattctttattccttgcttgcttgacattcccagccactcacgagtaacatcagacagagggagggtctccgaggtcgtcttgggccctccggagaacaccgagtttctcgttgctagtcgagcagtccataatagtgaggaggaaagagatgggcaatgttgatctttctcggggataaaatagtaagcttacgatatgttatattgtcagtaactattcttaaagtaatctataagacgtattaagcagtctcactgaagaggacgcaaaatggagctgtcgagggtacattgtggctcagaacggatggtacctcaagggtctgcttcactcggcgctgcgtcggacgaacccgaatccggaacggtcaatttcgtcgaagttacgtttgtacaggttagcgatgtccctgggaccatacactctcttcacgaagccgaagccggagcgatcaatctcgtcgaagttcctcttctcagggtagacgtcataatctcctcgcttgtggaagccgaagccagaacgatcgatctcgtcaaagttgcgcttgttgaatccaaatccagatcggtcgatctcatcgaagttgcgcttgttgaatccaa**

**V.** Pigment dispersing hormone precursor protein (DS01-Homarus1_Transcript_4536; translation frame -1)

**agctcgctcgtcattttcagacactttctcttcagtacttaccgcaaacaaaatactaatacataaacaagcttcatctgcttgtccggaaaatatcgtattaagattccttcttccgttacacagattattcatatattcactataatagttaaacagtaaaatatacgaattacgttcagatagacgttctagcatagagatctattaatcgtatatgataatggtactcgaagggtaagactaattttatgtctgtccagtttactcaatggtgggtgttggtgttttaatatacgtataaatatactgtatatatacataatcaaacaaaagcgttcctactctatatggtctttaatataagcacaagtgttacaggagtgaggtgctggagtaataagacacacgcttggtgctgttacattgtctcaaaagttagatattgatggtccgcccttagggtaagatggtgaaaggctctacacatatacacccgctggtctcgtccttagagttagcagaggaagggggaagaggaggaagtcgcttatctcctaccggcgtcgttcatcaccttgggcagtcccaggatggagttgatgagctcagagttgcgcttgtggggtcctgcggccatgggtccccatggtccctgaatcacacgcaggatctgcgctgccagctctgccacgacctcacgctcaggatacttgagttcctgcgcctgggtgaggacggcggtcatcaccaccaacatcaacatcgctacggccacggtattacgcattttgcttgagggtgatgtttgagatagacggcagaagaggcctgatggtggacgagggtgccagtccgtcgccttttatactgcttctcagctcttctgcgtcggcccccc**

**W.** Proctolin precursor protein (DS01-Homarus1_Transcript_4399; translation frame 3)

**agctggaggagtataagaggcgcggtggagcctcaacatggaagacgtaacaccgtaacagccaggtcccgccgtcagtagagtctggtgccacaagaacgtctgaaccgctcgctataccccctgcctgcctgtctgtctgccaaccccggccatctcacccgatcccgcaaacgctcgccaacctccacaccgcttggtcggtccatcctcgcgctccctgtgccctcgtctccacgcgtcgagccagcctccctcaacaacctacctctctctccacacaaccaacttcttcaaagatgacgaggactgggttggtcgtagtggtggcgctggtagtgctggcggccgcgctcacccaagctcgctacctccccaccagggcagatgacacccgccttgacgaaatcagagaactgctcagagagatgctggagcgcacagctgaaggagccaacagcaggatcagcggctccggttacgacaagcggttcatgtacaagcgatcagttcccgaagaaggcgcagccgagatggtgcagcctgccctcaacctcccacagtaactctcctcctcactcgtcaaccccgctcacctctcaacgagatacaccaatccacaacggcctcattaacaaaaccttaaagcttttatttgttctctcagccaccaataccaaatgaaacttgtgttcatagacaaagaaatatgtaactagataaattggagtttttgtaacacattaatttattcagaatcacaagtatgtgtgtagacaacctggcaggtataccgaattctgtgttcagctctttaactttattactcact**

**X1.** Pyrokinin I precursor protein (DS01-Homarus1_Transcript_33295; translation frame 2)

**tatttttgctagatgtacgaccgagacgttaggcttggaggacgagtgggccgggctccctcaggcgtccttcgcccagtatccacctgccctagacgatacctctgaggcccaacccctcagtctgctctacaacatgtatccctccgtcacctccgcagacacggtccccccaaagtcacaggagcttcaatataacagtcaagacacacccaagagactctactactcccagcgacctggcaagagaagcgtagatctctacgatgacgaggatccggagaggcgaatgaagcgacaaactcctcaacacgacaacgaaccaactgacgacaacgacgacagcactcacaggtggtggtggcccttcgttgcagttcgtcggtcactattctcacctcggttgggtaaacgtggagacgacatcaccaatgaagaactcgcttacgacgacaacctggccacctcagaatacctccgggacgacaataatgattacctgccggaggagttgactgaggacgttacagagatgtcatcacccgaaatgttgagcgagtcagcagcggcgttagtagggaagaacagtgtatcgttcatcccgcgcctcggcaagagaggtgatggtttcgcattctctccaagacttggcaagcgaggtgccgactttgccttttccccaaggctcggaaggcggagtgaatttgttttttcatcaaggccggggaaaaaatctgactttgcttttagtccaaggctaggcaagaag**

**X2.** Pyrokinin II precursor protein (DS01-Homarus1_Transcript_33296; translation frame 1)

**ttgggcaagagggattcggaagactccagtgtagagtcacggaacaccaagacccaagcctctatccctcgtcccggtcgggcctacttctccccaaggcttggctaaaccgttcgactgcattactcataatctcccatactttgttcttcccttacctgtccattacccaaagttatttatcttcctatgaaaagagagagcagactattaccatattctatgtaactctgaaacagagacataaaggctatactgtatgttgagcacctgactatgttggtaatgaatgaccacaagcaactgcactgggatcttagagaaagaacaatacccgtactcttgtcagtaaccttttatctgttgtatgtttgagtcaggactgtcatttgtgtttcatggaacgcaaagaatcagcatattgctaaaacaaatgagccaataaatattatctttatatatcgttgattatctggatcacgaaaccaacttaaaaagacactagagaagaggttgttgagctgcacgacgctctgactacccaaaattatctgttgttaatactaatacgtaatgccaacactaatcagaggttatccttggataccagttattcttacaaagctacacacttacacactgtcttgcttacaaattgtatgctgtaattaaattaattatattaactctcactctttaccaactaacctcacaacccaatttctatcctacaatttaataa**

**Y.** SIFamide precursor protein (DS01-Homarus1_Transcript_635; translation frame -3)

**ggatcttggtaattagtatttagcgtcagtctgagaatgttatcgaagcttatgagaaacaagagcatatttcagcgttcgtttggaagggattacacctgtaaaacaattgactatagaatatacttctcgtaattaactggggtgaaaataacacagcagcgatatacctcttcatatgtttattatcttacgctaatatggaggttcgcccctacgagaagaacgaatccgtcttggcttatattactacatatgataataaaaaataaataaataaaaagtaaaatgtaccgctcattttacgacacatacataaactctccgatactgatgtttcgataagattggagataaggaaggcaaaaataaaagataaaatattagtttatattaattatggtaacagaatcttgctacagagtgaatattatcctcactcaagggccataaaacttttcaaaagtaaacaatgccccgaagtgagcataacgcagtcacagatcagggatattcttgnnnnnnnnnnnnnnnnnnnnnnnnnnnnnnnnnnggacttaaatgtgattataattgtgttttgttttcattatttcacgttttctgatgaactggaagaggaaaaaagagatccatcgttttggtcttgaatactgtggttcagatccgccgttcacgtcagggagtcagagagaaatggagatatgttggttgtgacgtcggcaactacgcacgtcttgggacctcaagagaatcatttcttctcttggacggggaaccaagcggcacacgcctccaccgccacctgacacactgacgccagtcccttgccaggctcgaacactgtgtactcacgggggtcggctcctgctcgcttgccgaagatggaaccgttgaaaggtggcttcc**

**Z.** Sulfakinin precursor protein (DS01-Homarus1_Transcript_16600; translation frame -1)

**taagatacaggataaggttttatttgccccagccacttcacacaagacactacactacacttaaggacttgtgacacgtcttgacactttatgacacatcttaacaaattcataagtcttgacaggccttgacgaaacatgaattacctttgacctgtgttaacacgtgttgatgtgtctgtacgtgtgttgagattattagggacccacaaatcttatatataacaacaggaagaacaacatagtcgacaaaacacgtcatcatcatcatcataatacaacgtgaaacaaacgcaccaacacactctaattgtatctttcaaaacacaccttagttagcaatagagtgcaaatatagatctaatatatcaggcttttaatatggtgatattgttgtagttttgctcatataagaggaaaaagttcaccatgctttgaacacttgtcgaagggggggggggggtcagtaaaagccatgtgtctgtttttcttgttttttaattattatttttttttaattggaggtgatgtagtaaatacctgttcagtggaaatataataatgtgagttttcttagtatatattatgctgtaaagaacaacaaatatagtattgtattttttggaatataaatatttttggggggatttgtgattttgacttttattaagttaacctcccggtgtgttatggcacattgttatagtttgtgggatgggaaattggggaaaagtaattcaatttatcatcatcaattgtccagttgctgcacgacataaaaccattattaaactaaacctcggtgtgttatgcatggtgatccaaacactgatgacttcaccttagatatatcannnnnnnnnnnnnnnnnnnnnnnnnnnnnnntcaccatcctcatgtgaagcagcccgtcgtgtttgatgtttacactttctaatgtagtgtagatcatattgagaacctgtgactatatactttatcaaaaatacattaattcctcccacggtttgtttcgttggcgccacacacacacgaattaattaacttgtcgtgtaaaggtgtgtgtttagttaacggttgtgtcgtggtggtggtgttggtcgctgtgagtcagacttctgccaaacctcaagtgaccgtagtcgtcgtactccccgccgcctcgcttaccaaacctcatgtgaccgtactcgtcaaactccctcttgcccgccgcgtcgtggaagtccagtagttcagggtcttcaaagtcctgcaccagttcctccaccagcgccggcggcaggtggctctcctctaacctttgtctcaccacgggggccagaactcgtgctagggaggaaggtctggctggagccgacactcccccagacaacatgaaggccgccatcaccaccagcaccgccgcggtccagcttgtccacctcatggtttgtaaacactcgcctcaccactgtacacgatgttgttgttgtggtggtggtgtgtg**

**AA.** Tachykinin-related peptide precursor protein (DS01-Homarus1_Transcript_997; translation frame 1)

**gagttgagcgccacccaccgtagctgtcacacatagctacacccgctctcccagaatctccccccgtgcatctcagttcttctccccttcactctcacacacacatacactcactgctgctgcactccaccacaactccaacaatctccatcacatttcttcgtcctcaacagccaaactcaaccaataagaatggtgcgggcgtgtacgtgggctgtattactgggcgtggtggtggtcatgggcgtggtatctgctgcgggtgagggccaggacacccctcaggacagggagaggcgggcgccctctggcttcctaggcatgagaggcaagaaggacgcatccacagccctcgatgacaacaccgccgccagcgaatactcatccctgccagacccatacccactctacggcctcagggacaacaatctccctatgctcttcgctgtcccctggaaaaccaagaaggctccctctggcttcctgggaatgcgcgggaagaagagcgatgaagaggtgttcagtgacgccactgccgacaacgacctcgagatcttgctgaagcgcgccccatctggcttccttggcatgcgcggcaagaaggctccctcaggtttccttggaatgagaggcaagaaggctccctctggtttcctgggtatgaggggcaagaagtactatgannnnnnnnnnnnnnnnnnnnnnnnnnnnnnnngcagcagcagaagcgagctccctctggatttttgggtatgcgtggcaagaaggcttattacagtgagaaccccgacgaggagataagcatgacaggagtggacaagagaacaccctcaggcttccttggtatgaggggctgaggagtgagatgctccctacactctgctctaccgtgtctcaagatcatccctagagccgtcatccctgcataactcagaagcaccatttccagtgaccttcccagccaagtcagacaccttagtgcaggactcttttgtcatcactagtagttacctctgaccagatcttggtagttcctccacagataatattatgtagctaagcaatactgatataatagaaataaaagagaaaatgttaaacccccaaatatttataacaaatattagtcttatatacactcagcaggggggtgatgacagcagcaagtagtgcaagttctgtgcagcttctaccattgacgtccacatttgcccaaaagtattcttatatccttgttctctactttctctaacctacagctttctccatcactgtagtctatctgtacagttgagtccaacacactgtcaaatcttgtgtagtgctcatttcagcctaagtgataagtagtgtgatagctgtacccctgttagaggtgttggaggctgggggacgtagtgcagaggtccagataccacagcaccatgtaaaacccttctcatttacccttctcttattccgtttcccttggctggttttctctctcaatagaatatgaaccttactgtctgtacatcattataatcttgtaaattatagagaaattaatttgtaaattaatatatggatgaaggtaaaatttgccatcatataacatatcatttctggtgtaattgaccaggtccttgcatcaagtggtcatgacactcgtaatagtgttactgcttatgatgcaacttgtgacattatttggtcactgctgagtgattttgactctcccagttgccttgcttgacattacagttttgttaattaaattttgattgtttaaaagtgtcagtgattagcaataattacagtgaaatgccattaatggcatggaattttttgacagccatattttattaatttgtagtagtttgtatcatttaggcaatgaaagtaggaccttctaaaatatatagatcccatttattttcaaaataagatggagttagcactggttcacaaccatagaggcaactgtggaaggtcaagacgaggtgtctcctccactaaagtcgaaagataccaaaatattctgtaattgcattactctaattgcaataaaagacaatcctcgtaaaaaaaa**
