# Supplementary material for: Neuropeptidergic Signaling in the American Lobster Homarus americanus: New Insights from High-Throughput Nucleotide Sequencing
Source: PLoS One. 2015 Dec 30;10(12):e0145964. doi: 10.1371/journal.pone.0145964 (PMC4696782; doi:10.1371/journal.pone.0145964)
Supplement: S2 Fig — In this figure, signal peptides are shown in gray, while all mono/dibasic cleavage loci are shown in black. For each sequence, the isoform(s) of the peptide for which the precursor is named is/are shown in red, with all linker/precursor related peptides shown in blue. The “+” symbol indicate the presence of additional, unknown, amino acid residues at the amino- and/or carboxyl-terminus. Uncalled amino acids have been colored green. (DOC) [file pone.0145964.s002.doc]

**A. Prepro-adipokinetic hormone-corazonin-like peptide (from DS01-Homarus1_Transcript_14565)**

**MVGWQVMLAVMCLALAPTLAQITFSRSWVPQGKRSGGITGPLVTPGGGSDRGADPCKDVRLATLTQVASHLADLMDDTFDLPQDDAALALRLKHGLVARRRRMS**

**B. Prepro-allatostatin A (from DS01-Homarus1_Transcript_6078)**

**MVGEHGGLGTCVLVVVVLLLTTTTTTAHDYLEDLDDPDTSRLLDVLQYYDTEPSYLYDYGKRHSNYGFGLGKRTPGYAFGLGKREGLYSLGLDKRSDLYSFGLGKKSGSYNFGLGKRSVGDLPEVSKVEDGASPRTKRDVSITEDTLEDKRAKYSFGIGKRESSKNKRSKLYGFGLGKRDSGEERREDDDMENRTQQYSFGLGKQDPDMEIEKRPRNYAFGLGKRESDEDSDKRSQMYSFGLGKRDPDMDMDKRPRDYAFGLGKRASSDEDDEERYYAYEQGKRPTAYSFGLGKRAFSETDDYDNVNDNDDGDDELELSDLEQYSDDLKRATSYGFGLGKRSDAPDSGFGRRSYDFGLGKRAGRYAFGLGKRTGPYAFGLGKRTGPYAFGLGKRTGPYAFGLGKRVGPYAFGLGKKAGHYAFGLGKRAGPYAFGLGKRSGPYAFGLGKRADPYAFGLGKKAGQYSFGLGKRSGPYSFGLGKRSDSDSDQYTLGRRSGVYSFGLGKRAGPYSFGLGKREVSDDDHDEDEQDIGVEEEMSS**

**C1. Prepro-allatostatin C I (C-terminal partial protein from DS01-Homarus1_Transcript_3087)**

**+LQAMMNHLHMNKQQQQQQQQQQQQQQQQQQQQQQQQQQQQQGEEEVKRKRMFVPLSGLPGELPTIKRQIRYHQCYFNPISCFRRK**

**C2. Prepro-allatostatin C II (from DS01-Homarus1_Transcript_39)**

**MLTRFVSVVAVVAVVALLGVSRVSAKALPDQDPQVYGQMPHMLDPAGNHLIDDDGSLDAVLINYLFAKQMVERLRNNADIKDLQRKRSYWKQCAFNAVSCFGKRK**

**D. Pre-bursicon α (from DS01-Homarus1_Transcript_10307)**

**MGGLSWVLMVLGVATVVWSDECSLTPVIHILSYPGCVSKPIPSFACQGRCTSYVQVSGSKLWQTERSCMCCQESGEREASVVLNCPKVRKGEPTRRKILTRAPIDCMCRPCTDVEEGTVLAQEIANFIHDSPMGNVPFLK**

**E. Pre-bursicon β (C-terminal partial protein from DS01-Homarus1_Transcript_ 28875)**

**+TIASKLDSTMWTTVVLVVVCVVAAPCTHARRYDLECETLPSTIHVAKEEFDEAGRVERTCEEDLAVNKCEGACVSKVQPSVNTPSGFLKDCRCCRETHLRAREVTLTHCYDADGNRLTGDRGTLVIKLREPADCQCFKCGDSTR**

**F1. Prepro-CCHamide I (from DS01-Homarus1_Transcript_ 13319)**

**MSRGMINVFLVVLGVVALSSQAWGSCSQFGHSCFGAHGKRDGDQYARQEPSPLYPEANQLPEFEQRQEDRLSVDEAVTDREIVANARNWLAVLSHRLRQRTSPQSSPSAQSLGYFQ**

**F2. Prepro-CCHamide II (from DS01-Homarus1_Transcript_12464)**

**MSQIFGSLNLRKVSGSRTPLQSRAMTRPRSSTVLLLVFPLVLLCSPPASAHRVLKGGCLNYGHSCLGAHGKRAYVPVHPPVAPRPLLDVLLDALNTPTRSSHYSHARAANSVMGPRASYPEGRVKSPPTSDQLSDMGLDLRGEDYASGTNDDLESVGAIGGVRGSLDDTRDLAQDNVLYYGVLNDDYSDARYKRSAVSLPSRGRLGASPPLGVANNAVPQDRPHILREEHTGKDEMDPKYLALASFPNWLRR**

**G. Prepro-corazonin (from DS01-Homarus1_Transcript_40929)**

**MVKMYSQTLVMVVVVVVFAVTLAAAQTFQYSRGWTNGRKRSDPNVGVTELLADPPRRLSAHSHPHPPTHTLPKNIEERLRALEAGLNAVLKANSINFSPGGDEEYYAEN**

**H. Prepro-crustacean cardioactive peptide (from DS01-Homarus1_Transcript_1513)**

**MVKMYSQTLVMVVVVVVFAVTLAAAQTFQYSRGWTNGRKRSDPNVGVTELLADPPRRLSAHSHPHPPTHTLPKNIEERLRALEAGLNAVLKANSINFSPGGDEEYYAEN**

**I1. Prepro-crustacean hyperglycemic hormone I (from DS01-Homarus1_Transcript_5405)**

**MVASLGTSGVGGRSVEGVSRMEKLLSSISPSSTPLGFLSQDHSVNKRQVFDQACKGVYDRNLFKKLNRVCEDCYNLYRKPFIVTTCRQNCFEGDTFPRCVMDLGLDLELFLEFRDMIKG**

**I2. Prepro-crustacean hyperglycemic hormone II (from DS01-Homarus1_Transcript_49975)**

**MNNLFSIQTTTLSYTKMLVTVMVILVFTSSCSGRSWLIDGDEDLQLSQYHSLNKRAVFDSACKGYYDREFWGKLSRVCWDCENLFRQPGYQDKCSEGCFVTTDFTQCVKALLLNVEEYNELAELVRG**

**I3. Prepro-crustacean hyperglycemic hormone III (N-terminal partial protein from DS01-Homarus1_Transcript_1478)**

**MFACRTLCLVVVMVASLGTSGVGGRSVEGVSRMEKLLSSSNSPSSTPLGFLSQDHSVNKRQVFDQACKGVYDRNLFKKLDRVCEDCYNLYRKPFVATTCR+**

**I4. Prepro-crustacean hyperglycemic hormone IV (C-terminal partial protein from DS01-Homarus1_Transcript_4035)**

**+ENCYSNRVFRQCLDDLLMIDVIDEYVSNVQMVGK**

**J. Prepro-diuretic hormone 31 (from DS01-Homarus1_Transcript_2312)**

**MNSTGAVFVSLVVAFIFVSSVNSAAFNREARAVVQIEDPDYVLELLTRLGHSIIRANELEXXXXAKRGLDLGLGRGFSGSQAAKHLMGLAAANFAGGPGRRRRSSDDGLDLHHDDNLYAQDQAADLAESSR**

**K. Prepro-diuretic hormone 44 (from DS01-Homarus1_Transcript_7828)**

**MVLFRAAMLGVMGLFPLAWCLSLGGGRADTSSLLSLPHPQELSQDDLQPFLSRQGNTDSAGAPSSVADYTGYDKSEVLRGLEDPTSSSAYRLQEALSEAVAAAAAAAEGAEGVRDGAAALSPTANEGVTLEDLVPYDPGYYLYPAFLNRGDEAMTGGSSGINSLRKVRNSNRSNRSNSSSGISGSNTSSNSNTNNNSPDTISMAKRTWPNGFSRRRASGLSLSIDASMKVLREALYMEIIRKKQRQQMQRAQHNQKLLNSIGKRDVTRQLQQEGIQGVYQRGQRK**

**L1. Pre-eclosion hormone I (from DS01-Homarus1_Transcript_36484)**

**MVGSRKVVVSVLLVLSVMLMALLLLPSAAAAANKVSVCIKNCAQCKIMYHDHFKGGLCADLCVQSGGKFIPDCGRPQTLIPFFLQRLE**

**L2. Pre-eclosion hormone II (from DS01-Homarus1_Transcript_14893)**

**MSFKREVVVVVMTVVVLMTLATLSDAATFTSMCIRNCGQCKEMYGDYFHGQACAESCIMTQGISIPDCNNPATFNRFLKRFI**

**M. Prepro-FLRFamide (from DS01-Homarus1_Transcript_3291)**

**MIVAAWVLLTTLTWCCQAHAAPVPPVVAALDPPTDALLPAQSQEDDLFALPEKRLLKYFLPASQAWGGDAYPIGQEGTKRGYSDRNYLRFGRSDDNSKRSGRNFLRFGRSDTNDYEGEEMPESPEKRNRNFLRFGRDQNRNFLRFGRSGSPMEFATDLQEDVELPVEEKRGAHKNYLRFGRGNRNFLRFGRGDRNFLRFGRSVDRQLSSLSCEDCDEEQKAREFTSTPSPTTIQPLARTKRDVSAVLSDDSIESSVLRQINAHRIKRAAAQNFYIPMAWASELQPEEDGIDVTSFEEPQVAKRFSHDRNFLRFGKRDGSDDYPSSSSSAESPAPVVVVRPVEYPRYVRAPSKNFLRFG**

**N. Prepro-GSEFLamide (C-terminal partial protein from DS01-Homarus1_Transcript_30037)**

**+RAMGSEFLGKRQYEPEFAHTLDYDTKRAVGSEFLG**

**O. Prepro-insulin-like peptide (from DS01-Homarus1_Transcript_51972)**

**MRAFVVVIAVVVVVVLELGSSRASRRTYPTSEEEPRRRLCGWRLANKLNLVCKGVYNNPGSTGNYLFYRSRRDGESEPGLPPEKYLDLLADPEEERGLRHHYLTSSQQASEDTPSEENEAPGSFFGSLSPQDLPHQSAVQEDEASSVHFPFLTEEEASQMVRVRPRSKRGLSAECCRKVCTVSELVGYCY**

**P. Prepro-intocin (from DS01-Homarus1_Transcript_45955)**

**MQLGVVVVVMTVVVGSTTACFITNCPPGGKRSGPTAQLGRTRTCTACGPGLQGRCLGPEICCVLGIGCFLGTREARMCHAENLVPVTCANRDLKSCGRMQEGRCAAAGLCCTEMKCEFDSSCTVEGREERVGKQRAERQHLTFLSSLPEDQWNL**

**Q1. Prepro-leucokinin I (N-terminal partial protein from DS01-Homarus1_Transcript_36199)**

**MVTVGRWVSLWVRLAVALSSGAASVSFVTSEVMDVSPLALPHGRHPNLCTPDHVPSHPIIRCEVGKRQAFHPWGGKRSSFKAAPGLPLSLREVYLALFQNARPRPPPPSEGELKRASFNPWGGKRSDPLLPASQHEPNTKRNTFAPWGGKRAAGYFTHDTNPLIIEEDLIPYIGVLSDDGEAEDVVKRESFSAWGGKRGSFPADDWEEEEPTDLFVLDGSLPYPPVDRLRYKREAETYTNTLVNSDDSGVKTEAENIKPDTKYDQTAEASSTTVAKRTRFSAWAGKRPDLQVIEDAVRKMAEHEPKTTERRAFSAWAGKRSSDVNLQDGEDDEPVSAWIGRRLQDANTDDKRTFSAWAGKRSPSMDLSGNQDKRTFRAWAGKRSSGDELDDHFLDKKTRFSPWAGKRAEGTLSRLSESTLKAALDENSPEDNVDNHKRPSFSAWAGKRSESNEKRPSFNAW+**

**Q2. Prepro-leucokinin II (C-terminal partial protein from DS01-Homarus1_Transcript_31470)**

**+DEKRPSFSAWAGKRSSETDKRQGFSAWAGKRNNGGSDDPTHSNNPQQISSILQQLQHQGLEFLHKRLPNNDWGNKRVPFSTWGGKRASPISEDSQLSDLYTSQL**

**R. Prepro-myosuppressin (from DS01-Homarus1_Transcript_4887)**

**MVFRSCSWSCLLVVGVVVVMGVCVGVGETMPPPICLSQQVPLSPFAKKLCSALINISEFSRAMEEYLGAQAIERSMPVNEPEVKRQDLDHVFLRFGRSQQ**

**S. Pre-neuroparsin (from DS01-Homarus1_Transcript_14196)**

**MRSLGFVTSIAVIVVIVIVNETGAAPRCNQGGNRLPANNCKYGTVVDWCGGSVCAKGPGEACGGEWSENGECGAGTYCSCGYCNGCSANLECWFGSYC**

**T. Prepro-neuropeptide F (from DS01-Homarus1_Transcript_7995)**

**MRGAVMVGAVAAVMVAALVAGMASAARPDNSAADTLQAIHEAAMAGILGSAEVQYPNRPSMFKSPVELRQYLDALNAYYAIAGRPRFGKRGNHGAQRTEELYDY**

**U. Prepro-orcokinin (C-terminal partial protein from DS01-Homarus1_Transcript_1885)**

**+GFNKRNFDEIDRSGFGFNKRNFDEIDRSGFGFHKRGDYDVYPEKRNFDEIDRSGFGFVKRVYGPRDIANLYKRNFDEIDRSGFGFVRRSAE**

**V. Prepro-pigment dispersing hormone (from DS01-Homarus1_Transcript_4536)**

**MRNTVAVAMLMLVVMTAVLTQAQELKYPEREVVAELAAQILRVIQGPWGPMAAGPHKRNSELINSILGLPKVMNDAGRR**

**W. Prepro-proctolin (from DS01-Homarus1_Transcript_4399)**

**MTRTGLVVVVALVVLAAALTQARYLPTRADDTRLDEIRELLREMLERTAEGANSRISGSGYDKRFMYKRSVPEEGAAEMVQPALNLPQ**

**X1. Prepro-pyrokinin I (Internal protein fragment from DS01-Homarus1_Transcript_33295)**

**+IFARCTTETLGLEDEWAGLPQASFAQYPPALDDTSEAQPLSLLYNMYPSVTSADTVPPKSQELQYNSQDTPKRLYYSQRPGKRSVDLYDDEDPERRMKRQTPQHDNEPTDDNDDSTHRWWWPFVAVRRSLFSPRLGKRGDDITNEELAYDDNLATSEYLRDDNNDYLPEELTEDVTEMSSPEMLSESAAALVGKNSVSFIPRLGKRGDGFAFSPRLGKRGADFAFSPRLGRRSEFVFSSRPGKKSDFAFSPRLGKK+**

**X2. Prepro-pyrokinin II (C-terminal partial protein from DS01-Homarus1_Transcript_33296)**

**MTRTGLVVVVALVVLAAALTQARYLPTRADDTRLDEIRELLREMLERTAEGANSRISGSGYDKRFMYKRSVPEEGAAEMVQPALNLPQ**

**Y. Prepro-SIFamide (C-terminal partial protein from DS01-Homarus1_Transcript_635)**

**+KPPFNGSIFGKRAGADPREYTVFEPGKGLASVCQVAVEACAAWFPVQEKK**

**Z. Prepro-sulfakinin (from DS01-Homarus1_Transcript_16600)**

**MRWTSWTAAVLVVMAAFMLSGGVSAPARPSSLARVLAPVVRQRLEESHLPPALVEELVQDFEDPELLDFHDAAGKREFDEYGHMRFGKRGGGEYDDYGHLRFGRSLTHSDQHHHHDTTVN**

**AA. Prepro-tachykinin-related peptide (from DS01-Homarus1_Transcript_997)**

**MVRACTWAVLLGVVVVMGVVSAAGEGQDTPQDRERRAPSGFLGMRGKKDASTALDDNTAASEYSSLPDPYPLYGLRDNNLPMLFAVPWKTKKAPSGFLGMRGKKSDEEVFSDATADNDLEILLKRAPSGFLGMRGKKAPSGFLGMRGKKAPSGFLGMRGKKYYXXXXXXXXXXXXSSRSELPLDFWVCVARRLITVRTPTRR**
