# Supplementary material for: Neuropeptidergic Signaling in the American Lobster Homarus americanus: New Insights from High-Throughput Nucleotide Sequencing
Source: PLoS One. 2015 Dec 30;10(12):e0145964. doi: 10.1371/journal.pone.0145964 (PMC4696782; doi:10.1371/journal.pone.0145964)
Supplement: S3 Fig — (DOC) [file pone.0145964.s003.doc]

**A.** Adipokinetic hormone-corazonin-like peptide receptor (DS01-Homarus1_Transcript_58353; translation frame 2)

**agacaggtacttcgccgtcctacatcctctcaaggtcaatgatgcacagaggcggggcaagataatgctcttcttcgcctggctcatctcagcggtcatctccctccctcagagcgtcatcttcaacgtgcagccacaccccgactacccagaattctaccaatgtgtcaccttcggattctttaatgccaacaacagcggccagatgatgtatacgatattctgcatctcttttctctacttcatccccttatccatcatcatcatcgcctacacacggattatcttagagatcagccgcaagagtaaagatacacaacacgactacacgagggaggagcgctaccacggtcgactacagctccgacgcagcaacatgtccaacatcgagagagccaggaccaggacgctaagaatgacgtttatcatcgtcatggccttcatctggtgctggacgccctacgctctagctacactctggaacttcatcgaccccaagaccttctacagtatgaacaaacacctccaagatatcctgttcattataggcgtgagtaactccgtcgtcaaccctatcatctacggccgctactccatcagttgttgtcgagacgtatggaccaaatctggtgaattctgctgttactgctgctggtgttggtgttgttcctgtgcttcccaccagcgcctcggctctgtaccccctactaacattaactcctgtaagagtaatggccactgtcacgtgaggtccctactgatagagtggaaccgtagctcacaaaagtccctggcggcgccggagggtggcgag**

**B.** Allatostatin A receptor (DS01-Homarus1_Transcript_30123; translation frame -2)

**tgtgtgtgcgtgtttaattgtgaagacagcaagcataatttgaaaaaaaaataagatacaaatattatggatgtagattgtttttgcagttataacgaatgagatgtgataatgaataaaccacgtagaataatttcaatggaaacttatagtctatatgaatgagtcaacaggagatgtgtataaatttgagatactaaatatggcattcattaatataatcatcggtgaaaatagctatacctcttatggtctacatattatattcaactaaattattgatataattaataaggtagagagtaccttacatactaaagcgtattctttaagctcaaactgttagacattagggtatctaccacgagccaaaatgttgaaaaatcttcagtgtgaagatccttttccagtgacgtgacgaaagccattatttgatagttgaaatacgcgggggtggaatattgaagtgctagtatggggtgggggttgttaaccccatcacgggtagcacctttttaatatggccaatggtgaactccacactcttaacacttcctccagagtgttgcctccatggcctgtttgcatcattatttcactttatctgtggcccaaaacaagacagaaaaaaatcattaaggagaatggtccttaagcatcccctccactgccacgttttactgacaggtagggagaatggtgacctagaatgatactgtccaatataacgattaacagtttaaatacaatgataatcaccactgatatatcacaatgtcactgtaagtaaattatggaagaaaaacaacaatgttcacaataacaatgaggaatacagataatacccacaatattttcagacaattatctaaacacatagcaaattgaccagcattaaataaaaggaatgcttcaataaagctgaaggtcatatcaacaagaaagaccatgtcaggttcattaaagctgtgtttggtggattagtgacgcaggtcagtttaccatgcaatgcgaagttgggggtaaaagttgggttaacatgcggggctcattagcatatcagcccaacgcacctccacaaaatctttcaagaacacaaaattatttatctcttgagcatattccatttgaatacaaacgagcaaatatccatatcaaccattagtatttggctcagtaacctacagcaatggcaatttgcatatttgttctcagtagtgccacttgttgaaggcagtgagcaagctgtaccatgctcaggtttttagggtcacgtcgatgtatgtaaacactgcaggattcatatatgtacaaatacttcacccacgcaactattcgtacacacttaccagactcacgtacatttttacacacatatgtgacttggtattactatccactctgcaaagcatctatcgctgtggatgactcctttaattttacaaagtctatctgatctctatatttaagtgtatttaataaatttgtctatttacaaaaatataggtatatgtatatgtttatacgacatccatttctaattcattattgtctccaatcccaaaatactctaccaaaaagattcttcaatatgataaatatgatattgattttcatcaatgtgcctcatttgttttcttctaaaatcgtcttttgattacaaaaaaattaccaagaatgttagctggttgaggctgaaccccctcgacgttattcacattgctcatgcaagttaaaggcgccatcgttaaagccgccgtaattggtagcttgagcgactaccttattgccaatgaagacatcttgtgtctgcgagttgttggaggtttcttctctggtggccccgttggtaaagctcgtggttgtagttgttgtggtgttgcgatgcaagggggggccggccgaaggacttaagtgatgagtgctggttaccatgttgatcaagggaatggattgtggactaggcctagggttaagtggcctcgtctccatcgacctttcaaagtccgtccggccattaagcgctgtacgtcgctgggggccacacgatatcaccttacggaatgcctttctgaaaggatcggagaggaaggcatagaggattgggttgacgcaggagttaatgtaagcaagaacctgcgctgctatctgcgtaatgatccggaaagttgtcatctcgtacaaatccagagacttgagcaacaaaacaagttgaatcgggaaccagcagacgatgaaggtgacgacgacaatgacgaccatcctggtcactcgtttcttaccacggacactctcggcgctgcggctcccccctggcaccactccgtaccacaaccgattgagaatcatcaggtacagcaccacgatcacagtcagtggcacgaagtacatggtggtgatgaagccgatatggaaggccatgtgattgtattcgtcgtccaggaaggcgcattggaaatagacatcaccctcgaatttctgtttcttgatgccgtgacagagatacagcggtatgcatgacgtcaatatcagaatccagctgaaggtgatagcatagagggcattcctctcagtgcggatgcttagggcagcgataggatgcaccaccgccaggaacctgtcaaaggagaggagtaggagggtgtagacggaggcgtaggcggtgacgtaggtgagatattgtaccgtcttgcaccagatgctcccgaatggccatgagggtaatatgtagtcggatgcggtgaaggggacgcagaaaacgatgaatagcaggtctgccatcgccaggctgaagataagataattcgtggtcgacctcatctgcttgttggcaatgatgacaatgacgaccaaagtgttgccgaagaggcccaccaagacaatgatgccgaagataatgggcacgacgatggcaacgatgatggcatactcgaaggccgggttttcctctaccgtgttgttgttacagagcggcaggttggtgtagttggttaagttacagaggatatacggaggaaggaatgtgaggaagggttcgatgccattctcctcgtccgtgtctgctcctcctcctcctccttccccttccttccccatctctgctaagtagtccatcgttcacgtttaactgcaagagctgatctatttatgtgtccactcactttacggctgccatcaactaaagttcatttattgcacgaccttacgatatttatttcacaaagtttgtttcagttgcagtgaactttatttttgtcacctgtatgccagacaaacctacaatgttcaagtcctgcatacacattaattatctaatggcggttccaacatgtagatatgtgtcattttaaatataaaattccaaattaactttgggtcgttgtttgggtgcacagcacatatccgggtccagccactttaattgtttactctaaactcaactaggcttaattgactaaataacgagaatttgtttccaatacacttgataagtatacaccattaggcatattatgacacttcatactggagtgtattacacacatttcagttctcaacacaagtattttgtttactgtataaaacttgacgtcttaaaaataagcactgtaacaacgcaacagggaaatatttctgtctgccagttaaggacttcagatgtgttatttctcacatgataggcattcgtaaaagaggattccagtaatattttacaccttcacattgtttgtttttcccataaatcgttccttcaatgggaattatttttcaagctcatcgatcatactacgctcaaaaagtatttgacagttcttgcttttcaacgtttattaaaacatgtgtgctagtgtctctactgtcggctgacatcaggcgatattctgtgatgtgttggccaggtgctcgttggatcttccctgatgtacagctgaccacaggtgctggtacccagtacaagagatgtattcttctaaatttgaaatggataacctctgcaatacgtacagcacagttttatgcacttttccctgtctacacggatttgacactggccactacacttaaatttttcaacccaaaacacacgaagtatatggtttcggataaaaacttttatgtgtcaaaagtgttacacgttaataaaaaaaaggttattatataaatattatctccacaaagcatatggcgtagcttcaagcaacgagactcctacccgaacggtttgtatgacaccactttgggctcgacactatacagcgtaccagccaggcctgataacacaccaaggcccacggacatttcccgccaggtgagaagtttatgtgagaatgaccagcctgacagctcctcacgtatgcctggtcagttaccaaagaacgacgacactaaggaagtgagggagtcgaccggcgcatcacagagccgagcttgacgagagagacagagtgccgggggctgccagctc**

**C.** Allatostatin B receptor (DS01-Homarus1_Transcript_24860; translation frame -1)

**gacactatctctcagcctgggactttcataccacctgtgtcacttgtattattaaaactggacgacctcatagatagccatgccgttgttgataccttgggtgttgtctgacagcgtgtgggaggagccgagtctggtgcatgtgattgtgtcgtccaggctcgggtgtcgtggactgacgttcgtgtgtcatgaggagacggcacaggtcctctcatgaaggagggtagtgagggtgatagtctctgaggggcggagagttgagcacctgtatctagagccactggaacgtacggaacggagcgaattgatgttcctgtgtccacttcttccccaacaacaacagaacaagtagcggttgtactgtttcctgaaggttcccgagatgcagtaaagagcgatgggattgatgcaagagttgatgaagccgagacaaaacccgacgattctaagagcgtgccagaagtcattgtaattattggaaccttccggatcaaagtaaaaccacaagagaaagacgtgtgtgggtaggaaacatacggcaaatatcatgacaaaacacagcacgactttagcaaccttcctacgggtacgaatctgtttgtggaagacgtgtgactccccgggaacatcagaggctaagaggtgacgagccatcaggagatagaatgtccctatgacaatcagaggcaggagatagtagataagggccttggtgaggatactagccggcatataccagtcgatgaagttctcagggacgggataacacacactaatggacttgacattagagactttgaagactcgcacgctggagaacactgcagaaggtgtggccagcagaacagccaccacccagatgctgacagctgtacgtactgttactcctctcgctgctcccactgccttcttaacaggagagacaatagccatatagcgatcagccgagagcgccgtcagagtaaacacagtcacccccaccgagatgtccctcacgaactcgctaaacttgcactcaaagccgccataaggccacgactctatggtgtagatggtggagacgaaggggacggtgaagaagagcaccagcaggtcaccaagcgccagggagatgatgtaggtgttgggtacgttgcgtagtgtcttgttacgcgcgaagatcacaatcagggcaccattaccaataaccccgacgatgaagatgatggcaaagagcattgggacgatgtaggtctcgggtcgttgagggtaggggacgtagccggtggtgacgttagtgtcgttctcgtagatgccgtcgtttgttacgttgacggaggtgaagtattcctccgtcaggttgagagagagagagtagtcgtcagaaggaccccaccagctttcgttcttgatggtagtagagagccacagataggccctggtggtgatgctggggtctagaaggaacgtttggccccctgtaagaggccccacgtccactacaccagcagatagttcctgatctttttctgtggtgttgctaatgctccccgcctccatattggaattctggtgactgctcagatcttccatattggaactacaggaacgtaagatgtctgctgcagtaatcaataattttctatttggttttgtgtttatgtcactcttgagatatctcttttgttagggtatctctcgtgtatgataactcttgactcttttatttaagacttttaaagacttatgctcgacacttcaccattttccttgactatttcttattttaagtttactcataaaatcccaatataactccacacaataccagttacccccttgactactctacactccacacactcactagtctccacactaacttgatcaggacatacaaacagacatctttcttatatcacaaattccagatctttaaaaaataaacccaaataaaacttaacacaaagtttctagttaatttatttgttatcgtcgaacgttttctcactattcactattatgttactgttctacgtaaccaacttgatatttggtaacaagtcctcagtgataattatgatcctcgtgttggtctccatggtatagattccttaagggcgtctcacctgtagtagataattacgtttagaagtaatgtcaataggtgtatctgcaacaacattatttacacccgatacatataaaagatattaatattttctcagtatgatttttagtttttttttattgttttttttttccacaggtgataaatgctgtgtagtagctaggtagaagcttctcatctataccaatcaatccctcgctgacctgtggttcctacctggctccatctaccattaagataactgtagcagaggagaagatctcgttggtacagatgataagttatctcccacaagactggcgttcacttaagtggagtggaggagagagacaggccactggtgaactgaggagacttgagttaaggaagaaggtcttgttagctacctcactcatctcaactcatctcgactcatctcttacttggttattgtaggaggcatcatcagcactcaggtagattagacagatggcacacagtgagagggactatccttgagtgtcattggttcattggttcactaatgtcccactgcttcatttatctcatagttatatttcaccttttctacagtatattcgtctcaaagaacatatttgattctcactttatatctacacaacaaacacttaaacacttattgacactcaagatatcgtaaaagtttgtctatttcacccgatgattcagtaattgtttcgaatcagtgttatttgattcgaattgcagtccgctttgagggagaattgtcacaggtgaattcaatccaacaagaaaatacaacttggttacttagatcgccttcctgttaattacagatcagagaggtggagcgcacagacgatggtccaccgcgcaccgta**

**D1.** Allatostatin C receptor I (DS01-Homarus1_Transcript_10681; translation frame 2)

**cgggcgacacacgtgggggctgtccgggttctccccgagtcccagccaccttacccagccagttggtcgtcagccacgttctcgcccacacacgctcccatacgctgcctccccgtgtgatctgggccacccaaaccttcgtgataacctcccctgaaaactttatcatctcgctcgagggaaaacaagatagagaaaacttttatttgtattttccaacgagacgatcttgtcttaatttttatcttaatattatttaagactgtgaggaacgtttgtagagttagtttagtaagtggagacaagagacgatggaattgtcataatctctcgtgttttgttcacctacagacaagactcatgtatgtttccgtaaaaatacaagacatgtgacgcctcatcacacatgataatacaatgttaggtatttcnnnnnnnnnnnnnnnnnnnnnnnnnnnnnnnnactaaaatgatactcatacaggattagtattttcccccataaactcgttgcatatatagctggccaaagtgtatagcaactctctcttacgaacacactttcctcacaagacagacatactgtttggccaggtacagtgctgtaatctggtgtatcttgaggtgaagtgctgtaccctatactgtacaatatcttacggtgcacagctttatggtactgtgaaccctgccgtgagaaacaccagcacaaaagtctggtgtacagaaaaacacacaccgtggaagccagtgtgtgagtnnnnnnnnnnnnnnnnnnnnnnnnnnnnnnnnnnnnnnnnnnngtgtgtgtgtgtgtgtcaccttgcagctgacatatacataccatcaagtccctcttcctccaccttcttactacgaacgttcccttggccagacgttctcaagttggtggatgaaaacgtcctcggcaagacgttctccatctggcagaaacacacagaactgacggaggaaactggttggcaagatcctgtgagaggttctggatccacgagagctctgtgagaagtattgatccacgannnnnnnnnnnnnnnnnnnnnnnnnnnnnnnnnnnnnnnnnnnnnnnnnnnnnnnnnnnnnnnnnnnnnnnnnnnnnnnnnnnngagagttctgtgagaagtattgatacaggtcgttcctttgatcctttatgaatatgtcctctgagagctgcacaagaaggtaataagtaaacatgaaaagtaattcctctctcgtgtcaactcgacagtgagagaatcgactctggaggagaacacactcttacttttgagtccgactttaaagaatcacatcaactaccaccaccaccaccaccaccatgaactccaactcgacgctgtatttggacttcgatggcaacgactccgactccttccctcttaattgttccttcctcaacatgaaagatccagacgcaacgagtaactgtagtctcggggtgttcacaggagaggtgtcccctatacacgtggtggccagtatcattatgcaggcttgctacgccatcgctttcctggtgggtctgtgcggcaacaccctcgtcatctacgtcgtcaccaggttctccaagatgcagacggtcaccaacttgtacatcgtgaacctggctatagcagacgagctctttgtcatcggcattccgttcctcatgatcacctcagttctgggctactgggccttcgggtctatcatgtgtaagttgtacatgataacgacgtcactcaaccagttcaccagttcactgttcctgaccatcatgagcgctgaccgatacatcgccgtctgtcaccccatcagttcgaccaagttccggacgcccatgatctccaagctggtctccctgacggcctggaccacctccgccctcatgatcgtgcccgtcttcatgtactccaacacgctggaagttaatgacctcatcaactgcaacatcttctggccagacagcttcggcatcagcggtcagttcgtcttcacactctactcgttcatactcgccttcggcatccctcttatcctcatcttcatcttctacgggctggtgctacaaaaactgaagtccgtgggacccaagagtaagtcgaaagaaaagaagaagagtcacaggaaggtgacgaagatggtgctgacagtgatcactgtgtacgtcatgtgttggctgccctactgggtgctgcagctggcgctcatcttcagcccgccccgggagggccagagtcccttcatggtggtgctgttcctcatctcctcctgcctctcctacttcaactcggccatcaaccccatcctgtacgccttcctcagcgagaacttcaagaaaagcttcatgaaggcgtgcatctgcgccaccaggaaggacgccaacaacgccctggccgtggagaactcggtcttcccgcgacgacggggcggctctgcgaaacccaagaacgccaaggctcacgaaatggagtccaacgacgcctacacgactcagtgcaccagggacgaagccacgacggccatcaccatgaccacccgctctaccgtgagtcagatgggcagggagaacggcaccgagatccccaccacccagctctgaagacgcggcttctactgctactaggcacctcctcccaccaggtacaggtgtgtcatcgttttggggggctcagagtctgtagcaagctgagactctacaccaagtgacgtcatcatccactcaagctgacatcttcgtcgtctgattggttgacgtcatcgttggaggcagttttttgtaggtagtcgctgaaattatttgtgaggagtttgtgtccattgtggctgaagctataaacggttttttcctgttgtcgctgaaactgaaaacgtgtcatcttgtactcgctgaaaccgaaagctgttcctttcttacagtcgttgaaactaagaacatgtcacccttgtgctccttgaaactgaaagcgggttttagcttaccaatacagaaactaaacaccaatttcttctcgccggagaaacaaaacaaatatcatttagttctcgtgataagtggaaacccctttacctttcgtcatagataaatgaattgcagttctacaagacaccaaaaggccgcagcggctaccaaatgacatctggaggttctcacatgatgccacaacttgtaggaggagagtcactgaagtttctggcaatacttggtgtccccagaatgacttgtgacggaatcctcagcgttttactcacatctcgcaacagagacctcgaggtttccgggtgtcaaagacctcagttggcttagtggataaaaggtgctagaggagagttccagatgtaaaatatactccaggtactattaacagagggagctccagatataacgcatacgccacacattagctacaacagtgagacattataagactctaacaacataacaacattacaaga**

**D2.** Allatostatin C receptor I (DS01-Homarus1_Transcript_10638; translation frame 1)

**cagcggctaatacgtctgtctgggtgtcatgcaatgggcgctttcgtgtggtgaacttgagagtgcagcttctacattgaacaagtcataaagacattaaaacaatgatcttctcgttctgatagactccctgttgtggtgtggtggtactgtctttactgcataatataaacaaaggcaaccaaattgagaacgatggagaacgacacacttagtgaaccagaggacaccatccctctcaactgctcttatctgttgtatggtgacctctacaacaggtcagatttcctcaacgaatccaactgcactctggggctgttcacgggaggcaaaaatgagatgagcatcgcggccatcgttattacacagatgttctacgccatcacgtgtctggtgggtctgtgcggcaacaccctcgtcatctacgtagtcaccaggttctccaagatgcagacggtcaccaatctgtacattctcaatcttgccatcgctgacgagctgtttgtagtcgggatccccttcttgatgaccacctccatgttgcgttactggcccttcggctcaatcatgtgtaagttgtacatgatcacgacgtccctcaaccagttcaccagttcactcttcctgaccatcatgagcgctgaccggtacatcgccgtctgtcaccccatcagctcaccgaggttccggacacctatgatctccaagctggtgtccctgacggcctggaccttatctgccctcatgatcgtgccagtcttcatgtactccaacacactccaggacaacggcttagataactgcaatatcttttggccagagagtcaaggagtgaggggcgagatcgcctttatccgatattccttcgctcttgctttcggaatccctcttactctcatctttatcttctacagtttagttctccacaagttgaagtccgtcggacccaagtcaaagtctaaagagaagaagaagtcacggcagaaggtgacccggctggtgctgacggtgatcacagtttacgtcatctgctggctcccctactgggtgttgcagctcaccctcatcctcagcacgccaaaacaggggcacagtaacttcatggtggtgctgttcatgatctcctcctgcctctcctacattaactcggcgctcaaccccatcctgtacgccttcctcagtgacaacttcaaaaagagcttcatgaaagcgtgcacctgcgccgccaggatggaggtcaacaacgccctaagacctgagaactctatgtttccccttcgtcagcgggggacatcggcgaaatcccgcatgacccgacgagacagagagtccggggagggaactacgtcccagtgtggacttagcaaggagccctctacagccgtcaccactaccaacgccaggcccaacctgagcaacaacagcggtagcagtggcgatgagctcactgtcaggaacggaaggtctccaggaccccggctcccggaccttatccagtaggctgcctcgacggccatgctgctgggctgtcagcaacacacacctgctcggccatcctcgacacaacacattgtgcttaaggtttcctgaacgtcttaatcattcctgagataaatgggaaccaaaaccacatcacaactattggtcacttaatgagtaacatgttataataggctgagttctttcaccttatatcacaagtgatgcaaaaaaataacattaacctgtgagttatattttgctgtcaacctccacaatgtttagttaacaacagacctggactgttaatgtgggtactgatgtggcgtcaataatcttatatgaaggtcaagtcattatgactgtaacgtcctttacacgttacatcactgacttgtatgtaacgttcttctgaagctcccgttacgtcacctacctgcacgttacataacttaccctggtagtgatgttggtcaaattcacgtaacatcacacacctgtgtcttgcatcactcatttgcatgtgatgtcatttactaaatgttgtgtcacttgaatgtaaaataattcaccaacatctgtactcttacctgaacctgactcacttaccttcagtacttggatatggtattccttacttgtagttgacgtcacaggtgttgttaaagcgtccccccccttcctcccccaccaccttggcgacacttttttgctttacttttgttttgtttttatttttcatgtacataacaactgttgtgagatgtggtagctatacggaggcagctcaccaaagtgtacctctggcagtgtcgacgctaccgaacgacgtcatctttgtctctcacaacagctggtcaatgttaacaacactactttttagtcata**

**D3.** Allatostatin C receptor I (DS01-Homarus1_Transcript_26036; translation frame -1)

**ctcccttcagtgttgtgagctaataacatcaccataacatcgtcaggaagggactgtgagttgtcagtaggtcacactatggtattatctgcctcagtcccacaggatgacgaagccttcacctctggtctagaggtgtgaggtgaggggtctgccgttgaggaaggcgtcgtcgcggggcaccaatggtcgccgtgtagcagacagaggcactatcacctcctccctcacacacccgtcgtgggacgcaacctccttcggttcctttgccctgggaggcttgcaggagcctcctcgtctcctggggaacatggagttctcagccgccagggcgttgttgatgtccttcctggcggcgcaggtacacgcctttaagaaactcttcttgaagttctcgctgaggaaggcgtacaggatggggttgatggcggagttgatgtaggagagcgaggaggagatgaggaagaagatgatcatgacgcgactttggacctgtttaggcatactgaaggtgaggatgagctggagcacccagtagggcagccaacacaacacatacgccgtgatcaccgtcagcaccagcttggtcaccttcctgtggctcttcctcttctttgtcttggtcttcgcccccaccgtcttcagcttgaggatgaccagcgagtagaagataaagatgagggcgaaggggcagccaaacgccaagacgaaggagtagatggtgaaggcggtgtggccattgacacccactgagtctggccagtagatgttgcagctcgcttgttcgttactgtcattcaggatgttggagtacatgaacaccgggatgatcatgagggcggagatcatccaggccgtcagggagaccagcttggagatcatgggcgtccggaacttgggggcgctgatggggtgacagacggcgat**

**E1.** Bursicon receptor I (DS01-Homarus1_Transcript_10555; translation frame 3)

**caggattgcgcacaaactttgcagccatttgggctaacctggctgctgacttccctccagccaataccgacctggttgacaccacctcgctcgacctgggagaagccaccgccgccacccacaccctgcccctcctgcctcgccatcaggtcctctgcaaccccgaaccaggtccgtttatgccgtgtgaggatctcttcgactggtggacactgcggtgtggggtatggatagtattcctgctggcactgctggggaacggcgcggtagtggtggtgctcgtgtttgccagagccaagatggacgtgcccaggttcctcgtcactaacctcgccttcgccgatttcttcatgggactctacctagggttcctggcggtggtagatgcctcgacgttgggagagttccgtatgtatgctatcccttggcagacgtcagttgggtgccaagtggcaggcttcttgggtgtgctgagctgcgaactctctgtttacacgctcaccgtcatcaccatggagcgtaactacgccatcactcatgcaatgcacctcaacaagcgtctctccctgcggcacgccgcctacataatggtgttgggctggctcttcgcctgcaccatggcccttctacctctcatcggagtatcagactatcgcaagtttgccgtgtgtctacccatcgagacgaaaggcgctggtctgggttatgtagtatttctaatgttcataaatggcgttgcctttcttatcttgatggggtgttacctcaagatatactgtgccatccgcggttctcaagcatggaacagcaatgactcgcggattgccaagaggatggctctcctggtgttcaccgactttatttgttgggcacccatagcgttcttttctctgactgctgcctttggattacagttaatatcgttgaaagaagccaaagtgtttactgttttcatcctaccctttaattcatgttgtaatccatttttatatgccctgttaaccaaacagtttaagaaagactgtgtgatgttgtgcaagacaattgaagaatctcgtgtaacgagaggaattggacgctgccgtcactcatcaaattttagtaaccgccagactccggccaacaccaacagtgcactggagaactcctcacgtcaggataaccagttgtgtcgttgccagaacaagacacaagaatcacagaaactccaccaccgcctacgtatatcagcactaaaataccttttctgccacaaagatactgagggactcaattccactagcgactttagttaccagcccaccaaaagtgctgtcaagtctaagcgtcacacttctgtttcgagcgagacttacagttcttcctggtcggacacttggcgtcgaggccatgcagccatgtccctacggatacttgaccgtcgtcaccacaattcctggtacctgtctcgcaagccttcgcaggagagcaatctttcctcttctcgtaatgactcctcagccaccacagcctcaacatctacgtggcggatatctcggtcttctgtgtcatctgatatatctagcagtggttcaagaggtgtgggtaaatctgacgtggccccaactttacggttggggtctctgagagaacgtcgaggggaatgtcacatacagatcccaactcgacagattactcatcatcaccaagcactgctggtgaggcagcagtctggcgcatccggccaacgatcagccccaatcacatcggcagtccgcatcaagcctcggttacaaagacagagtgccattgagcgtgaaacatatatacccaataaggcagcagggggacaaaatgaaataacatgcccgctgcaccaacgatctgataacctctcttgtgtatatgaacaagaaagctatgaagaggaagaccatgaggcctcaaaagattatttaaacccacgctgtcctatggcaggacttactgtgacattcattccacgtaaactgagcaccatctcctcccattcagttagtgttgtcagagacgcagagggcgatgaacctgcagttggaccctgtgtggatgtacacagctctagcgatccatttccaatgtccaattgtgacttttcacggggagggaaatgtgtgagtttgacgcttcttccgcagtcttcttcacaaacgagtccttcacgctttccctcggacggtcacttaccccgctctcctcgatgcacagaacttctatacttcaccaacttagcagccccggcccttatagtcccaagcactgaacaaaacgctgagtctcctcccaaagatctcgatgcaacaccaaaaaatcattatggtcaagcaatcctcattcacagtcagccacggtctccacaaagtcttgagcacgatgagtgtatggagtccaccgcactgatggatgatgattgctatggtgacgatgaagtgtttgaagaagaaaataagagtcgtgaaagacctttagaaacacactttccacttgacgatcctcctggggaaacacgccctcttatatgataactttttactgtttttacaaatgtggaacagtaacgaggaaaaaatactagctctcgaaaccggtgacaaagatctgaacctttccaccttttatcctcgactgtccaccaacataaaacgtctaaaactttacttcataattactgttataatcagcatgctgtgaaaacaatacctacatttttataattgttctataatgccatgcatcttcacaatagcaaacttttcctccaagtgctcagagttagccctcagctctccgttgtagctctttttaggcatagttacaaagttaccaacgatgcatttgtgcctctgtacctgttctgaacattctgaggaaaatttccatgtgatgtagtgatgcctgggcatagtgtggaggaagatggagcttgatggtgggtggtgaggtgtgtgaaggaaggtgaaaagtgatgttgggtggtgaggtgtgtggaggagggtgaaacatgatcgtgggtggaggagggtggaatgtgatggtgggtgttgaggtgtgtggaggagggtggaacgtgatgtggggacacagaggatggacgttggctcggctctgggaccagattcaactttagtcctccttctttagagttaattatttatggtgctctctggtgacttttaaactgtgtacgtatgatattaacaaagacgttgtgtcgaatttgttatcttctgttacgtgtatgctaacagtatgcaagttatgcagcatggggtacctgttgcacccctgatacaccatataactatgtacaagtttttatgtggttccaagaatgaaacaaagttattacagcgtttgaggaaatacagtggtccagtggaagaaaactgattatatttatattaaaactgtaaatggtgttgacgatttattagataaaggtttcttaagaaattagtagacatatgaaagtgaactgtgtgaaagtgaacccaagtattatgaagtctttttaaatatggcagtctttagtgccattataaatactactcttgctggtgaagttcaaaatcgatctctctgtcgtgctgaggagagtgaggttcattgtgtgttttcagcacatcatggtaaattgcgtctcacctcattttgaatgtgattcatttaggaatcttaatgtcttttatgtgtgtcggacattgattgtaagttctccagtgctgtgagctgatgaaacaccaaattatatgatcatttgacaaaagatgtcagtcccttgtgttttaaacagtcactacagtgtggtgtatagaagtaaggaaaggttgtgttattaataatgtcgaaactaccatgccactattacacttcactggatgacactgaaaatatgtgtgtgtgtgtgtgtgtgttttgtgtgtgttttgtgtgtgtgtgtgtgtgtgtgtgtgtgtgtgtgtgtgtgtgcgtgtataaagtcatatttgcattacttgtcacct**

**E2.** Bursicon receptor II (DS01-Homarus1_Transcript_16714; translation frame 3)

**caccaccttcgtcttcgtcatcaccaccaccaccaccacgtcaacaccatctggctcttcccgtcacgccatccattcgaatccggcctcagtgcatccctttgccaggaccgttcatgccgtgtcgtgacctgttcgactggtggacactgcggtgcggggtgtggattgtgttcctgctggcgctaatggggaacggtgtggtggtggtggtgctggtggccgcttacgccaagatggacgtacctcgcttcctcgtcacaaacctcgccctcgctgacttcttcatgggcgtgtacctcgggttcttggccgtggcagacgcctcaacgcttggggagttccgtatgtatgccatcccgtggcagatgtccccggcgtgccaagtggccggctttctgggagtgttgagctcagagctctctgtgtacacgctggctgtcatcacactggagaggaattacgccatcacacacgctatgcacctgcagaagcgattatccctccggcaggctgcttacatcatggccgtgggctgggtctttgcaatcaccatggcggttctgccactcgctggggtctctgactatcgcaagtttgctgtctgtctgcccttcgagactgacggcgctggtctgggttacgttgtgttcctgatgttcatcaatggcgtggccttcctcattctaatggggtgctacttcaagatctactgtgccatccgcggctcacaggcatggaatagcaatgactcgcgcattgccaaacgcatggcactccttgtcttcacagacttcatctgttgggcgcccattgctttcttttccctaactgctgcctttgacattcatcttatctcccttgaggaagcgaaagtgtttactgtgttcgtattacctctcaactcctgctgcaatcctttcctctacgctctcttgactaagcagttcaagaaagattgtgtcatgttgtgtaagaccattgaagagtcgcgcgtgacaagaggcatcggccgctgtcgccactcgtccaacttcagcaaccgtcagacgcccgccaacacaaacagtgcagttgatcacacctcgcagggagacaaacagtcgtgcagctgcaaaggtaaacaagaaaagaataaacgtcctcgatggactttcatctcactaaaatacctcttatgcactaagggggcggaggaaatcacctcatccagcgacaccagttaccagacggaccccgcccagcaccgagggccccgacacacttctctctccagcgacacgtacagcggctcttggtcagacacttggcgccgaggccgcggctccaccacactgaggatgatggaccgtcgtcgccacaattcttgggcagcatccaacaagccctcgcaggagagtagtttgtcgtcgtctcggccagactcctcggccacctcagcatcgacagccacctggcgcatctctcgctcatctgtgtcgtcggatacgtctaacagcagcggtagggtgaagaccagtgagcccggtggtccatctaggttaggatcattgcggagaggcgatggacgcgggtttggaccaacacgtcaactcagccagagtcaattcgtgaaagagacgccatgtactcggccgccgcctgtccgccccaagcctcgcctacagcgtcagggggccgtcgagagagaggcatacacaccaggtaaggaaaacagtggcctggagagccctccctgccctctgcacctgcggccagatcacctgtcttgtgtgtatgaacagagccatgaagatgacaacatggcggctggaaccagtcctggtgctggcctgccaacacaaccaacacttctcctcagtccgtgtagtctcgacactcccatggatgacgacgtcttcactgaccctcccgacgaggaaattcaagttccagttgggtcttctccctcccacaggcaggagattactcttgtgatgcctactgaagaggccaatgaaccggcagccactgagaacacagcactcatggcagaaggcgacactgacgctgaaaaggaaacttcgaaggaacctcagtcgaacaaagtgttggagacgcacttcccactggaagccactttaccggaggtgcggccattgatctaacgttatccacgctagaccttccctcctcttggctctactgagactactagatctaaggttctggctgcctggaacatcccccctcctccctacagtaacttggctgtctggaacacaccatttccagcagtaaacttccatccctgacaccatgacctcctagacctgccctcctattccttccgaaatctgcaacgcttccaggcccaacagaggatctggctttcccgagccttgatgcatcttgagtagcatctccagggacgagactacagctgcatggaatgaactgcctctgtagcacctccaggtctgaggacttagactcctggcgagcctctactctcagctccagaggtcaaagcctcacaccaaccagtagcgtcatctcgagcaagtacagcattgccgtcacccaagccaaagaactgtagtgtaagtcgtgtattcagtagatgtttgtatactctgtgtctttaacattttatgattatttaatgagcttttaaaaaagagagctaataaattatttacatttatctttcattcccttaaccttgtgggtaagttcagagggagttacctgatgttattgcacaaaagaaaaattacatacaccagtggttaataataataatccatgtaaatattacaaattttcacacttttataagcaatcaagtttcaggtgtaaacaacagcaactattattgtgtgaatgaaacgtgtgttttaatcagtaggcgtgaattaatgtaatgaggaatcttatcttatcttaatagggaagagtaatacccacattgtgattaacattaatattttatttgtgtatgacgaacagatatacataaccaaatgcctttatattacacacagaattgtataaattccataaacaaatgtatacacaactaacgaaaaattaagttcaacacagcgtgattgagatgacaaagtacgataattactgctactttttttttagggaaaaattattgaaataattctgtaaacagtacaaagaagcacacgatattgaactaaatgaaaaataaataaatgtctctgaaaatgtataaatgaagcaatataacaaaacacttcgaaattagtaccgctgccagacgtaagataatgacgcgatcaattaacaaatgagtgttgccaaacgtacgataattatcgtacatgtgcgataaattcatctttgacacgatgtacgataccattttctattaccgtacggtttgtacgataattttgggtttgaaagtagtgtgacagtaaatttggttatggtggccgttacgaagcagaatggtacgtaaggagtgtgcaaacctccacacttgaaaaattttagactaaattaccgcaaacaaagaaatgggctaaaaactaaaactgtaaatggaactcttctctcatctgagtgcctcaaaagttcagggtcatgtgtcaaattttcgcccccagaagatatgcttgcacaattgtttcacaacttgacagatgaggatcttgaagtagagggctaatggtgatcactgaaaatatatgttttagtgtggtcttactgagttattaatctgcaagagtaagcacatttaagaatgatttgagttttatgtaaatgactgcttactcttattcatattgattctctagaatggcgtatatttattttccttctaataatttttgtaataaaggatatgatattactgtaaacttatatgattttcattgcttgtcctagttttgatactacctgattataagtcagtaacaaaggtatcattctgatgaagaaaattataatgggtaagttttcaattaataatcccaggtacacatgataatgaagtttgggaatgcaataattaaaaaatgtatttaataataacacgataattctaccgaaaatacgataattttgaagatccagtacgataattcccatctgaaaatgtggcaacactgttgacagttgcctgggcgaggaaggtcggcagacagtggtgggttttaccgagaggaatttacacttgttcttgaaaatactgtacttaaaactacgtaaggtggttcccatttaatatacaagtacattgaagtatacaacagaggatctcggttacccagctatgattctacgttacttaggagagttactaaggtgagttcatttctgaaatgttaagaatgactccttcacagtatacagtagttaagttcccaccaaacctaaatgcttcattatgtcctgtatgaagcagcttaaagaactgaataactaaagaaatcggagaatttacaagaactctcaagctgaggcactaacttaaaatatcctcaaaagccaataaaaaaatagctatctgagcatgcgtcgtgtaacaagcaggtagttagatatttagtatcctgaccgaaaatgggacaaatatttgcgttctgttttagaactttaaataagtctgaggccagtcaaagaatatttatgatacaatgttgggtgaattttcgatcttgcaccggtatttctctcgctgttattaatatgtttttcgtaatgtttagacaattctaagacctttttcttcctgtgatatttccgccggaaattaagggtaagtgagatattaatttttcaatattatta**

**F1.** CCHamide receptor I (DS01-Homarus1_Transcript_24860; translation frame -1)

**gacactatctctcagcctgggactttcataccacctgtgtcacttgtattattaaaactggacgacctcatagatagccatgccgttgttgataccttgggtgttgtctgacagcgtgtgggaggagccgagtctggtgcatgtgattgtgtcgtccaggctcgggtgtcgtggactgacgttcgtgtgtcatgaggagacggcacaggtcctctcatgaaggagggtagtgagggtgatagtctctgaggggcggagagttgagcacctgtatctagagccactggaacgtacggaacggagcgaattgatgttcctgtgtccacttcttccccaacaacaacagaacaagtagcggttgtactgtttcctgaaggttcccgagatgcagtaaagagcgatgggattgatgcaagagttgatgaagccgagacaaaacccgacgattctaagagcgtgccagaagtcattgtaattattggaaccttccggatcaaagtaaaaccacaagagaaagacgtgtgtgggtaggaaacatacggcaaatatcatgacaaaacacagcacgactttagcaaccttcctacgggtacgaatctgtttgtggaagacgtgtgactccccgggaacatcagaggctaagaggtgacgagccatcaggagatagaatgtccctatgacaatcagaggcaggagatagtagataagggccttggtgaggatactagccggcatataccagtcgatgaagttctcagggacgggataacacacactaatggacttgacattagagactttgaagactcgcacgctggagaacactgcagaaggtgtggccagcagaacagccaccacccagatgctgacagctgtacgtactgttactcctctcgctgctcccactgccttcttaacaggagagacaatagccatatagcgatcagccgagagcgccgtcagagtaaacacagtcacccccaccgagatgtccctcacgaactcgctaaacttgcactcaaagccgccataaggccacgactctatggtgtagatggtggagacgaaggggacggtgaagaagagcaccagcaggtcaccaagcgccagggagatgatgtaggtgttgggtacgttgcgtagtgtcttgttacgcgcgaagatcacaatcagggcaccattaccaataaccccgacgatgaagatgatggcaaagagcattgggacgatgtaggtctcgggtcgttgagggtaggggacgtagccggtggtgacgttagtgtcgttctcgtagatgccgtcgtttgttacgttgacggaggtgaagtattcctccgtcaggttgagagagagagagtagtcgtcagaaggaccccaccagctttcgttcttgatggtagtagagagccacagataggccctggtggtgatgctggggtctagaaggaacgtttggccccctgtaagaggccccacgtccactacaccagcagatagttcctgatctttttctgtggtgttgctaatgctccccgcctccatattggaattctggtgactgctcagatcttccatattggaactacaggaacgtaagatgtctgctgcagtaatcaataattttctatttggttttgtgtttatgtcactcttgagatatctcttttgttagggtatctctcgtgtatgataactcttgactcttttatttaagacttttaaagacttatgctcgacacttcaccattttccttgactatttcttattttaagtttactcataaaatcccaatataactccacacaataccagttacccccttgactactctacactccacacactcactagtctccacactaacttgatcaggacatacaaacagacatctttcttatatcacaaattccagatctttaaaaaataaacccaaataaaacttaacacaaagtttctagttaatttatttgttatcgtcgaacgttttctcactattcactattatgttactgttctacgtaaccaacttgatatttggtaacaagtcctcagtgataattatgatcctcgtgttggtctccatggtatagattccttaagggcgtctcacctgtagtagataattacgtttagaagtaatgtcaataggtgtatctgcaacaacattatttacacccgatacatataaaagatattaatattttctcagtatgatttttagtttttttttattgttttttttttccacaggtgataaatgctgtgtagtagctaggtagaagcttctcatctataccaatcaatccctcgctgacctgtggttcctacctggctccatctaccattaagataactgtagcagaggagaagatctcgttggtacagatgataagttatctcccacaagactggcgttcacttaagtggagtggaggagagagacaggccactggtgaactgaggagacttgagttaaggaagaaggtcttgttagctacctcactcatctcaactcatctcgactcatctcttacttggttattgtaggaggcatcatcagcactcaggtagattagacagatggcacacagtgagagggactatccttgagtgtcattggttcattggttcactaatgtcccactgcttcatttatctcatagttatatttcaccttttctacagtatattcgtctcaaagaacatatttgattctcactttatatctacacaacaaacacttaaacacttattgacactcaagatatcgtaaaagtttgtctatttcacccgatgattcagtaattgtttcgaatcagtgttatttgattcgaattgcagtccgctttgagggagaattgtcacaggtgaattcaatccaacaagaaaatacaacttggttacttagatcgccttcctgttaattacagatcagagaggtggagcgcacagacgatggtccaccgcgcaccgta**

**F2.** CCHamide receptor II (DS01-Homarus1_Transcript_37689; translation frame 1)

**tctctctctctctctctctctcactccacaagaagaaatcaagaatgaccagttaggggaaaactgatcattcttgctggtgactcagataactgtaaataactgactgtggtagactgtaaacactccagaacttacaagtggtgtagtgagagctgactgaggtgagctggtagatgtgagaaaagaaagtaaaaaaagctactattttcaccagcgtatgttaccagctggacacacatacacacactcatcatagacacaaaagattctgatggagttttttaacctctattcctcataaaagaacctaaacaaaaactcattatcgttctcaagaaaagacaaaaagttcctcaggagacgatggaagacacggccatgttatcaccgacgctgctgtttactaatcagacgttggacttggacgttgagaggaacaccacacacaccaacaccctcctcgacgacaccatcctagacaacacctcatggtccttcaactacaccaatacttcaggaggacaagacgttggcctcagtagtaacgaaacgtacctgccctaccgagaacgcccagagacctacctggtacctatagtcttcgccctcatcttcatcacgggcgtggtgggcaacggggccctaatcttcatgttcctcaaacacccgaaactcaggagtgcgcccaatactcatctggtgtcgctggcggcgggggacctcctgatggtgctgctgaccgtgcccttcacctccatcgtctacaccgtatcatcctacccgtttggagaggccgtgtgtcgagcctccgagttcgccaaagacctgagtcttggcatcaccgtgttcacactcacggctctcagcgcagaccgctacatggctatcgtgcgccccgtcacacaccacgtgtcggactccaccgggcacgtcgccatcgctgtagccattggaatatgggtggtggctgctcttctggccacacccgcggccatcttctccaacacaccagaactggtcaaccccaagggagaaaagttccatatctgtacaccatacccggagtacttaggggcagtgtacaaacaagtccacgccctggtgaaggcgatcatgtattaccttctgcctctagctctcatcgcttcattttatgtcctaatggcgcgccatctgttcatttcagcccagttcctgccaggtgaggcggcgggccagcagaggcaggcacaagctcgccggaaagtggccaagatggtgctggctttcgtcaccatcttcgccatctgtttcttgcctttgaatgtgtttaatctgtggtggcactttgcaccaaactcccgggaaacttatgatatttactggcacacttttcgtatagttgggttctgtctctcgttcatcaactcctgcatcaaccccatcgctctctactgcgtcagcggaacctttagaaagtactacaataaacacgtgttctgctggtgcactagacactcaggcaggagagactgggaaggagccgagtccacaggaacacggataaccacggcggtgaggacggaacaaatccctctcaagatggtgggaactgacaatgggacgcagcagcctcctgcacacagactgacgctgaccaacaccaccgtcctcaccactaagcctcaccacaaccccgcctccctcgtgtgacgctcccagtcatcctgaagctgctgcattactccctccctcccatctgcacctcctgtaatcatttttctcagctgttcatcaccgtgtcttcaccaccctctcaccacctcctcacttatctacactacgatgctttcctcgttcatgaatcaccaactcttatgataggcagtctctccccttcacgtcctgttgacagtggtattgaagcttctcgccatctgacatttcaacagcacaaggcagcttacctcacaacccctctctatgtttgtaccttgatccagtgagacgctttaaagtgacgtcatctcacacacacttaatattccaccaatcagaagttcttgatcaaacagtagtacacgtcacccaccagctagtgtacacaagcaccaccacacgacacacaaacactcataccataataaaacaagacccgacgtcacagtgtagtttttaaaatcacacgcaaacacaagcacacaaacacacatctagggaaagacaattttgccctacaatcagcttttctttctatcttcaaatcttgttcttaaaaattccatcgttttttcttgagataattagaaaaaaaacttttagatatagacgatgtttgagtgtttgtgtagcacttgatgtaagataatagagtttcagtgttgatgtttgtccattttcttggtcgtaattatctgtctgaccttaaattaggtcgtaaggtattgcttattctatgtatgagcagacaaaggcggtgagaaataacctaaagtgtttctgtgcaagatataacaccctcacaccgaaaaccatccatcagtattctacaacacttagcaccgcccacaatactcacaagctatccagtgattggctgggaagtactgatgagcgaggccaggggagacgctgaagcccttggccccgcccacatactccagaatccaattacaatcgacggacttgtgaccaatcagatcttgtcctctcatgatgggtggtgcccgctgtttggaatattaatgaatgacttggtaactgacatacaacttctttaacccggaaatactactgtagataccaacatactgtagaggtactgtatcttaactgtacatacgacaaaaatatcactagagaatacagcttttaatgttgctatcacttctctgtgcagttgtcaataccatcaatatttttttttccttataatcgggggatagacgtaagttgagacaatatatgtgtgttaaaaaaaatgtgtgttattacttgtgaattaatattattttgtatcgctacgtaaagactacaggccatctaggaggtaggttcaccatttaccctcaacaagacaccaaacaatgacttcataagccttataacgagaccaacaagtcatttctcttacagtaaacatcttagtgaaaacatctaacgaatatcttcaaacattaacccattcagtggtagaaatcacttaacgttaaccaggataagacaccaaaatccgacacgactcctgaatgggttaatgagatgaaagtatcccccccaaaagttcccaaagaagtgaaaaaaaaagtccaagagagagaaaaagaagtccagtgccctaataatcaccggactttttttagggggtgacttttttatcatcagttttattgacagccataacttcataatcctcactattgaatctaggtcacacgcatgttataaccagacccattattaattcacaaaggtcataggtcatttgtttggtgtttcatgggggattagtttacctccactcttttagtcccgttttctacaggggaaacaacgagagaggagaatctgtccattacttcccaaatcccataatccaaaccacatttttcattattgttctatttatctaattgctcttaatagaaaaaaaatatttactgtaatgtttaagtttatttctaatttatttgtctatttattaatttcgtttataatctgataatctctttgtttacaacatcattatttaatcggtttctttttactgaagattaacaaattcacaacaggtaaactctcactaattagcttttaccttgataacacctgatattatttactttacatgacttataaacaaattttttgaaggcagtatacagtaggtgatgatgatggaaaattgtacgctggggaacaaggcttctcattggatgtgccctgtgaaaacaagttcggtgattggctgattgtacgctcaggaaaggaagacacaagaaatttgattggttgtttgtgagatccggaaacgatataatgattggataatgat**

**G.** Corazonin receptor (DS01-Homarus1_Transcript_48447; translation frame -3)

**tttggctcaatgatttcttacatgtaaatatataaataggatttttttttggtggtcgcaggttgttatgttcccattcactactcactaggtcgagtgaaccataacaccacccacttataacatttacaacagttgtgtgtctgagtgtcgctagagatccacatgaagtatttcaacactgataggttgtgacttgtgtgttaactatgtgggtcaattaccacaaaaaaaaattacatttccagaagcagtaacttgatggaacacaaggtctttagtggcaccagctggttgtaactctttctgatgtgagagagtcagctgatggtacaagaggccttagtcaccagtcagctgcgcctcttacagctgtgtgtctgagtgccgctgcaggtccatcttcataagggggtagttgttacagggacactgctggaggagtttggaggacgagataacaccttcgttctctgagtctgagggcagggggtcggcggggatcttggcggcacgcgaccaagagtcactagagctcctcctgccgttgctgctggccaccccgctgtctgcacgactcagctgctgtttccactccagtccctcctgttcctcctcctcctcctccaggccctgactcagagggctaaagccatcgtgacgaccgtagttttgggatgtcttaggactcagtcgggggtgatgatccaggagtcccttcgatcgtcggccatagcggacagagttcctcaatggtccgtctcctattgatgtctccaaggggcctgaccgaggtgatatcagggccctttgacgactcgactgacgccggaaggaatagcggaccccgtcctcgaagacgttgacgacggaggtgtccatctcggcagcggaggacctacaggtggtggagcggctgctgttccttccacttgcctggtaccgaaggtttgatccacttctgtttataatgatgaggttgaatgaggccttccgtttatggcagcgacacagatggaaagcgccgtagattagaggattgaccaggctattagacatcccaaagaagaatatacctgactgaagctcggctgctacattttcctcaacttgggtaaacataaatataatcatcatcatatagtatggtgtccaacagatgacgaacgccatcactataaccactgagatcctaagcgccctcatcttggccttcctcagtagtcgccttcggttaaactccgggcaggtgttccccaaggtggtacgctcgttacggaacaccttttcactcttatgcagagtgacgaaagtggagacgtaggtaacaagaagaattaacagcggcaggacgaacatacacaccagactgaacaccccgtagagttgctccagccaggcaggggagtagagaccgtaggtaacacactggtaaaactcctcgtagaagggaccacgcactacatggaacaccagcgcctgaggtatgctcaagatgccgctcagcacccacacgaagatgatgccatagctacagtgtcgcagcgtgtcggaccgtctcatggggtaccgcaccgcagtgaaccggtccacaccgatgagaaccaagatgaaggtggacaggtagaggctaaacatct**

**H.** Crustacean cardioactive peptide receptor (DS01-Homarus1_Transcript_31037; translation frame 3)

**cctcattaaaagataccaccacccttgtgtgtacaatagggctcgaaccaaggtacatgagaagtgttgtacgggggtgactgccctctcctcggctccacggctcatcagtcacgagacccacggaacatacacctcacggacacagttgagaagatcacaacgcgtcctacaaggtggtgttcaggtggtccaggtcacccaacatcccagtaaggccagagggagggagcagcaacccctaatacaccctctaagccgtcctggattcgcctcttgaaaaattggtgatttgaagataacgtatcatgccagcgtcagtgttgagactcccttgtaataggaagcagtgacctattacgacccttactgtgttctctcgtgaaataggaaaaaaaccctctctgatccgcccaattttgatacggaataggactatagtgacctattgtagcctacacagaaaataggaacaccttctcatttgggtcacgaaattgtgatattaacccattctgacctaccatgacnnnnnnnnnnnnnnnnnnnnnnnnnnnnnnnnnnnnnccctctaattttgagcctattaccatgaaatagaataaacctttggactattttcgccccaattttccatgaaataattgagggttaagggtatatttattccggcccaccatgaactaggtaaatcctcaggacttatcatgaaatcaaccatcatggcgggcaaggtcgagaagtcgccatataaccagaccgtcaccgtcgacagccaatcacagctctcggagggggtcgactcgaagacgaaatccaatcataactcactctacgtcctcgaggatgcgtttattgaggtcaactgctcggacttcatggacttagccgcggccaatggaacttgcggcaactatactatcacgtccaatgttaccaatactaccactacagcgggggactcttactacttctacgagacggagcagttcactgtgttatggatcctgttcatctccatcgtggtggggaacgtggcggtgatagcggctctcatgctctccaagaccaggaaatcccgtactaacttcttcatcatgcatctggctctagcagatctgtccgtggggttgatcagtgtattaactgacataatatggaagacaacattgtcgtggaatgctggcaacattggctgtaaggcggtcaggtatgcacaggtgttagttacttattcctccacctacgtgttagtggcgctgagcatcgaccgctatgacgccattacccatccaatgaacttctctggcagctggcggcgggccaggaggctggtggtggtggcttggttactgtcagccgtgttcgcctcaccaagccttggattcttcagggagacttctattgatggcgtcctccagtgctggattgacttctcagaggcgtggcagtggaagctctatatgactctcgtggctctcaccgtcttcgtcttccccaccatcatcatcaccgcctgctacgccatcatcgtctacaccatctggtccaaaagcaagatcatgacagtgaacagcaaagccctcggtagcaaaaacggggagaagcgggtcacgtccggggaggatgactcgaggcgcgccagctccaggggccttatacccaaggccaagatcaagactgtgaagatgaccctcgtgattgtcttcgtgtttatcctctgttggtcaccgtacatcgtgttcgacctcctgcaagtatacggctacgtgccccagacgcccaccaacgttgccgtggctactctgatacagtctctggcccccctcaactcagcagccaaccccatcatatactgcctcttctccactcatatatgccgtaacctcagaagaatcccagtggtggactgggtggtcaggcaagtgttcccctgcctggagtgcgctcagcagccggtcgagaaccgcggagccttccaccgctacggtaccgagtacacgaccgtgtctgacgcctccagccggcgacacactctcacttccgtcag**

**I1.** Diuretic hormone 31 receptor I (DS01-Homarus1_Transcript_ 5552; translation frame -2)

**taacgtcgaggtggtgtattatagtatctggtgaacggtaaggaactgaaaattaattcagtagttagattaacccacatgtaccatcctaaagtacgttcattaatatctatttttgtgtcattcatgtatttttaaagctctatcttgccatatcacctcggtgaattctttgttttttctatcatttactttgaaaatttaccatcgcatcctcagccaaagaataatcgtctctctcccccttcattcttattattacttattattttatcgatgaaaaaaaacattaacatcaaaattttgctttgttctccacgttctacggaacattgttgtagacgagttacattcgccccgcgggaaataacgaggctgggaatgtgtgtgcgtgtcgaggctcttgtcccctatcaccctctatctcgtctccttgtgattagttggctctcgtatctgctgctgtagcgcaggttttacgcatatgtatactgttattggtatctccatttctactctaaatgatctgtctatttatgtttctatctctctatgtgcatatagataagtaattaacagagtgggtaaatagagagatgattagggtagagaactagagttgcaggtgtataaacaggtgaagagagattactagatcagcagatatgtaaagacatataacgatgaacagagaaacgacagaaagagaaatagacacagagagacagataaatggagagatacaattaaataaacttgcaggtgttgatgacaggataggaaatatatatacaaagtgaaactaatttcccactataagtgtggatgatggcagttcggtggattctgacttccacagacaacacggaaatggtagagaggacacgtggttttatacacaaaagcttacagtgagacctccccgacacactgcaggaatttaacgtaattacattaacggtattctagttttcagtaattttcagtttcttatgcaagtttaccgactttgggattgaaaatatcattgagtggtcaacattcccccgtggatataatcccaattagaatgataaacgttgaaatatttcggagtttgttgatattaaagattaaaattccctttgaatcaataaacagtgatactagttagggtgtatatactgtatgtcagagggaaaaatctttttgtattaataaacaatcagatatctcaacttaggttaatggcagagcctttggttcgatgaaatttgaaaagtctttgcactgatttatatctaaggaaaatactcccattttggcctgataaacattgaggcatttttgtgcgaaattatgctaaagggaaaaataataattaagattttggtgatttcacttcattgagaaaatcccttttggattaaaatgcgcattacgatatttcaatcctaatttttgttcatcgatatatcccttctggcaacacgtgtttggtgtcttgactgtcttgactcgtcgtgatctggtccacgtatacctgaggtccccgtctacacgtcgtccccttcaatggttgtgccgtagttggtgactgtcgtgttcccatcagccatactcgatttcatgtannnnnnnnnnnnnnnnnnnnnnctctccattgaagaagcagaagagcagcgaaacacccagaccctggagggaggcggcgatggcggagacgacgaggtagaagccctcggctggtgaacccttggggggcctgaaggggatgaggatgtagtgaaggcccagcagcggaatgaggataagtgtcgcgcggaccgccttgcgggtggagtgcgtatcaggggcggtgttgacggctcgcagtttggtgacgagaaccctgacgatgttgacgaggaagaagagattgaggagcatagacaacaccacaggtacgttcaggatgtagttgtagttcccgtcttccatccaacagtaaacactctgttcatcgtcggtgcctctacatgctccgtagataatggtgaagacggccggggcaccccagcccagcatgtagaaccacttcataatcctgtcctcggcgacgaaggcaacgacgagcagggtgtgcaggtagaggccctcgcagaacatccagaaatagttggagactaggaaatagtggaggaaaacgtgcagcacctgacacccaaccgtgttgctgaagacgacgtcaaccctcccaaccacacactcgaaccacactaaccacatggcgttgttgataatgaagcttacaaacaggttcttatgaatggtcacacgagtgcacttcagggatttgaagaagaagaagatgaagagggagatacaaagagcaacgagggacatggaatacccggcgatgtagatagtgttgactccctgatgaagctcgaggttggagacgtcaatgcaggttgtgtagttggaccagaagttgttggtgtcgggatgagtgaaccacgtgccattctcgtcacagtccttgtggccgctacgttggggatcaaacccatgaatgaaagacgggcactggacgtgggctcgggtgccggctggggtgtcgttccaacaggaccacccatcgaacgtccgggggcaatacggaccgaaagacggttcaggaataggcatagtggcatggagagcttcacactcagcttgtcgacgggcgatttcctgcttctgtttaaaagtcccttcccaccctttgctagacccagcctccgtcgacgatgagttgtcttgcttggttgccgacatttcttcccacgcccaatcatccatgactgagtctgtctcgttgccctccatctcagagcttcggttaaagaaaatattatccacttgggactaaactgagaccaaaggataaatattcccttgtttttatattcacagaagcgtcctggtcaaccggtcctttaattcttccagactttaaactgtgcttgcaagacgtcctcgagcgctgatgattttcttgcgacgcgtcctcgagtttttataatgttcatgcaacacgtcctcgagcgtgtatggtgttcttccagctaagacttccaatacgtatgatatttctaggaactaagtccaagagaatttttaactattccggcaactaggacctcgaatcaaggctggaccgtcgctcacacatgttctgagacacggcgcgagatgtgtgtgtgtgtgtgtgagtcgagttgtaaggtacaagataactccttgattgacgggtttattttggctctgtcagattgtaataatatttgaaggatatgtctctttctgagaacgttctgctacgtctttgttactggtccaatttgaatttgttttccatgtatcttgttttaagtatgatttacgtacgagtgtcttaagtgggtgaacacatttgcagctgcgtctttcagtgtttaaatctagtattcggcaccaacacagccacccgttcacttgttgctggcgaagattcgtttaaatctagtattcggcacca**

**I2.** Diuretic hormone 31 receptor II (DS01-Homarus1_Transcript_ 26723; translation frame 1)

**ttaccctgtactactggtgacatatttaaacagcctgaagacatgtcctcactcaaccggacactggacatggagggacacaccactaccctcctggagacagtcatcgatgcccgctaccagttgtgtgtccagctgatgaagagtgtcccacagcctcctgacggtgtagtgttctgtcccaggacgtttgacgggtggtcctgttggaacgataccctggctggcaacacggcttactccccctgtccttacttcattaccggcttcgaccacacgcgtatggcccataaggtgtgtaatgaggacggctcctggttccggcaccctctcaccaacaatagctggtctaactacaccacctgtattgacctggatgacctaatgatgcggcagctcattaacaccatctacatcgcgggttactccgtctccctcatcgctctcgccatatctctcgttatcttcttccacttcaggacgctgcagtgtacacgtatccggctacataagaacctgttcctgagtttcatcctgaacaacatactgtggatagcctggtacctggaggtcgccggtaaacccgagactgtctttgagaataagacaggctgccaggtcctccacatcttcctacattacttcatggtggcgaactactgctggatgttctctgagggcctctacctccacacactcctggtggtggccttcgtgtctgaggagcggctcatgaagtggttctacctgctggggtggggcgctcctggcctcattgtcaccgtctacgccgctatcaggggctcctccagctccgacaccaagcattgctggatagacgagagccactacaccctcatcctatccactccagtgtgtatatctatcctggccaaccttgtgttcctgatcaacatagtgagggtgttggtgacgaagctcagagccaaccatgttcctgctgacaccaatggcaccaggaaagctgtgagggcnnnnnnnnnnnnnnnnnnnnnnnnnnnnnnnnnnngcgccggggtccaacggggagctggtatatcaggtggtgacagccctcgtctcctccttccaggggttctgcgttgcccttctgttttgtttctgcaacactgaggtaaccacggcgatgaagaagaagtggcagcagtaccaattcaaccacggctccaccaggttcagtctcaccaccgcctgcgtgctctctgttaatggcggtaacagcaggggctctacagctccccacggttcctcagtcctcaggaggacgttcagccgagggaaccatcaccaccgtcaggacatgttggagatacctggggaagggtctccatgggccaacacccgtagcatcgtgatggagaaccccagcttactcatggagaccagaagtgtcgtggaggaggattacttcggctccagagaggattgtgttggaggtgaaagccaacactcagccctggtggaaatgcaggctgtaggagacgtcagaaacaacgtagtggtggagacgcataactacgccaaggtggagaaggtggtgttagttgaccctccaaacatcgtagttgtggagcctgattactgttccttgctggagaacggggcgatggaggacactgaaggtggagacacaacagcaggaggaggacgtaggggtgaaggaggggtgctaaatcctgtaggagatgtgatggagggtggtgaggccct**

**I3.** Diuretic hormone 31 receptor III (DS01-Homarus1_Transcript_22256; translation frame -1)

**tcacgacatcattttattcatatgtaggacacttaacattcatggtgctcctcaaaagggcctctaaaaaataaagaacatcaggaaatatatattattacaactttatatctttggtatggacatttaatgtaccttacttctttttggctttacgtaagtatttctacaagaataattaatcccttgtctctgtacagctaaagtcagtctacttagtgtgtgtcgtctgtggactgcacgacacgacaatcatgtctcagtgatgtgtggagaccatcttggacaccgagtaaaattacaatattatatttttaatgctacacatcttggaccaaatctagtgtcagttcacatcaacaatgttgccaagctcggtggcaataggtataaataatcattgatactatatccggtgacaacacacatgagcactgttgccaaaactcgatggcaacatacatgagcatagctggcaaaaaggcctcaccccaaagcctcttaaccagaaatgctccggacgggagaattgggaagcagagaagacccagagagagatctggagatatacttgtgagattaagatagaaaaaatcaagacaacttggaagtttaagtggtctctgaaaaactcgagtcaccaaggaagatgacgtgtttaccttggcgactcgtgtgttatatattgttcaggaacgtttgacactttaaaaatggcaagaggaagttagagaaaaaacagcttaatgttggagatggtaagaaagatattgtgttcttaagaataaagaagacacacagtacatgattcatgacaactggtgagatggagactaatgaagcattctgatcaacaccgaaacatacaggatgaaactcatgtttagcgctcagctgatgatgtaaacaaacaaagcctgcacgttttaatgtgactcctgctacgaaacgaagcctttaaaagacggaatattgaattggaggaaaattatcaattaagaaaagctaagacaatttattttatgaagatactcatttgtttaagagttgtcggtgtccagtaaagacatggcgactattaccagtgaccagaggaaggtttggttcttcaagagtatccgcattataatgaagaaaaaaaagggtaaaacatgattcaagaaccaagtgataagcaggaagcagtagactgaaaacctaacattttgttgcaatacataagtagcaataggggagatcagacacgtgtaggttgtcaaacttgttcagttaggtgtgatgcttcgcaactactgagaacggtagtcaaaactggcgtcttaattttttatattttcagaagagataatgaatttcttatggcgagagatgaatgattactgagaaacgtctcacaatatctgagaaatatggagatacctcagcatgcaggaagacatttatggtaagttacagcacaactcttttggatgaatatctcaggcaacaagtaagatcagatgaacagcgtagcggttcagagaactaagattatatagtactgtacaatgtggggaagttacattcttatgtacaagaggaggacgagggaaagaagtcgactgaatgtctatgtaacacacaagtgggaatccttgtcttcagtgtattatttgcaatacagagaagtgtgcacagggtaagacgtcacttgccagttcttatcatgtccttaaaaaagaaaaaaaagaaagaaaaagggactatccaaatcttttacattactcacattgaggagttttcgcatttagtccttcgttgaagatatattatcatgcgataaattaacctgctattcatctaaaaccgcgatggtttgggaagactcgtagccttggtcaacactggtcctgaggccgcagaactgaccgtttacattaatgttcacacacggctccgcctcatctttcctgaacaacactacagggtgacgctccactctctcgccccccagtctggccgccgatggctgtggactcttctctctcttttccgaccttttcacctcgtcctctacttcttccagtagacttggggacctggagactgggtttgagtctaggttatggactggactctttgacgagacaaatctctctgagcctccaggtcccaatttacttgcaatttgttttggtgacctgcgggagggtgcggttatgggtgcggtcccactatcaacagtagatgcctgggactgttgcgtgatactggtcctggaacctcgaagactcagcagctggctggcattggctccgctgttgctacgccggaagggttggttgttggcgcagatggacacgatggatgagttccggcgcgcagggctggggtcgagcagggtcgtctggacggtggagaggctgcgactgcggagcttctcgggcccccgcactccacgtggggcgcgtcgctcctgcagccgtacccacgatctcttcaatagacttctaacctctccgttggtgtagcaaaagatgatggccacgacagcgcccgtgctaccctctatcacattgttgaagatttggtagatgtcgtgccagtcacagcctagctcctggtggcggtagatggtcacgatgtactgcagcccgaacagcggcaccaccatcatggtagcccgaacagccttcctgtactggctgggttcgttgccattggttgctctgactttgctcaccaggatacggatgatgttcaccacgaagatgatattgatcaggatagccaggagcggcggcaggttaatgatccaatcgaggctttcgttcggcattatccagcagtcagtgttgtcgtcacgcaacccccgcaccacactgtacaccgtcagaggcaccaacgggaaaccccagccaatgaggtagaagatgacgaggctgttctgctcggcgaaggcggaggctagcaacttgtggaggtagaagccttcacagaggtaccacatatagttgctcaagctggtgtacttggtcaggaggttcagtgtcacgcaccacgtcaggttctggttgatgaggtctttcttgtacagctgcaggaacctgaaggtgataacgcccaccgcttccagcagcagactcagaaacaggtgtttgtgcagcgtgatgcggtgaacacgcagctgtttataggacaagaagatacagagggcggggagaagggcggcgaccgagacgctgtaggcggcgatgtggacgtacaggcgacggtggatgttgtgctccacactgcatgacgagtagttgctccactcctgacctgatcccgtccgacggaaccacatgccatccgtctcgcatttcttcgtcgcctgtttggcacatgaaggttccctgccgtagtagatgtaggaggggcagggtctcatagcctcggtgtctctgggtgtggctgaccaacactgccagccgtcccacgtgttcggacagtgaccagtaggccaggaggcggtgagcatttggtcgcagcactggacggcggcctcacagcagctgcgccatttggaaggaccaacttcacccttgaaggacgccaacaccgcctccgaggtcacaacagcttcctccaactgagagaagttcatcgggttgaggaggacggagacattgtaggtgacgtcttcctcctccaccgccttctgcacctccagtgtaatccatttggtgttgaaaagttccctgttgacgatgtggaaatagcagagggcgcaggcgtcagcgttgtacatgtccggcgggaggtacttctcctttgatctacacagaggacgacctgcgctcaagatcattctttcgtgtaggtcaccatcaacgactttaccaccacctgcctccttctcctctagtggaccagctgttttactctcttctccaacagctgagtttttgtcgtcgagcggatcagctggtgcctcctcttcctcctgcaccaccttctcctgcaactgtgtgttgccggacaccactgacgtattttgtttgtcgtgtgtggtgtgtttggtgtagtctcccccctcacgctcctcccgggttcgaggcacaagctcggccgaccttcggtatgacgccctctttaaatattctttatacaacgtctgctgcgactcccggagcctcaagtcgtcacgctggtagtcgctgaggatgtagcgctctcttgtgtccttcccgaggtcatggagatcgtcttcaggatactccaagtccaggtcgtatttcccggggagccccaagcgatggtcgtcgccgctagaggcaccagggtcgttgatggtgtcgtcgagcaggaagcctctatgaagagactcctgtgtggtggtggaacgactgctgccgtcggtcgtccacagcagcaacatcaagtagaggaggcgacacacactcccagcaccacacttcatggtggtgtcgctgtgatgctcaagtccagtgggaaagtcctaaatgcagagttttatttgtcagtggagcgtgacgtaagtgtggtggtgttcactggctgccccgatcatgtccttcacacaaacactctggttcattactgttcttcgagctcgtagttcacaccgagggtagagtttaatgttttacacacgccagcgtacaagtgaggcctcagtaatgcccgctggtagtgagtagaaatattgtcactgcagatagtttgtctttatcttttgttatgggtcagacatgtaacgagagaagattttcaacaatgatgttaagaagaagtcaagcaggatggccgctgtggtgagatacagacgctgtggtggtgggtgtgcggcggacaccaggcgaccactgccgacctccaggacctggacacacagtcatactttatcatcccatggcagcttcctggagacgagcgaatagtgtgaactccgcttgtgatgccatacatagttagtctgcatgttgcaacgagtttttttttacttctttagccttgaaatttatgccaaaaaatgaaaattaaatcacaatatgtcccaatggcggtgtcactggcggtgtcattatcaacagattacagtgctgacgtatgggaaaagtaccagacaaactttgtgaactttttgaaaagtttttctgagatgagagttcagtaaactgccacagtgtgagggtgtggagttaatgtggtaggggtggtgtgccatggtagttcataaggagggcgtcacatggtgtgttggagagagcaggacatagagcgcggcgcgagacggtgagtcagtcagctggtgttacttgcctcgctggtgggagcggccctacctacactctcgcgtggcaccaccactactgacctgcgctcc**

**J1.** Diuretic hormone 44 receptor I (DS01-Homarus1_Transcript_11147; translation frame 1)

**attattcacgaaatggggaagaatcggtcggctcacggcaacggatgagtctacgcggtgcctcgtctcgccccattgagcaacatccaaccaattcttccccatttcgtgaataatccgccaattcagacccaaatttcgcgcacttctcttcaacttgcagccgatttctttacgtgtgtgtgtttgtaattcatctagtgccccatttatcgttccatcgccaaaaacgatcgtatatagccagcgatttttgaggtaaatctctcttttattttccgttttttgtgttgggttgtaattaccttccctccttccccctccctgtgtctctcacttactgggactatctctcccacctatcacaccctttaaataaattacacatcaatatattgttagataaaaagtgataaaatctcaagtgaaatgaagacaaaagaactgtgaacatgtaactgttgtaaagtgactgactggttggtgagcagattgtcaaggggaagaatcagactggataaaaactgagtgctgaagacaatctggttttcacgtttagccactgcagccatggtgagggagtgcggcgtgggcggcagactgcgagacaaggtcagccacccaccataccacccacaccaccacccaggactcaacacaccatccaccaccactacccgattcaacacctgttttcattagccgttgcctaaacacctgagatcttacacacacactgactgaggtactcaattttgtgcgtctgatagtaactcactggtcatctgagtgcgcctgcgcattaactgcgtctgaacggctcctgaaacacctctcctgaagtctgagtgcgtctgaagtcttagatatccagcgtgttattgaatgatcctggatgtgtcatgtttcttgttcacttcctcgagaacattagttatcgcctcccatactgactgaactcacttcaacgccaagtgtagccaaaccagtactagctaagtgcgtttatagtactcgaggagtgttcttgggagtgtttacgagtacagtacgtagtttagcatcacaattgaactttcaaaagagctgtcatgtattaggtgttggtacacatgtaagggactgtacctgcactcacccgatcaacaatatagtgaaattacagtagagtgatgttgagtgtctcgtgactactctgacaacaacaccaccatgccctcaataacagcacccacggagttcccgagctactctctgtccgatccggccgagacagaggaggaagattcggacgaaatttatataactctgtggagaaagttcatggagcagtccgccttgatcaacgccaccaacggcgaccacaagatgttgcagtgcttcaacatgtacctcaacaccaccatggaccctgagagtgacccgggggcgtgtccagtcaagtttgatggggtgtcttgctggcccgagactcccccagacaccacgagggccattccgtgtttcaacgatttcaacggcgtccattacgaaccttcagactacaacgcgacgctgtactgttacccgaacggcacctggtctaagaagtcgttttataacttctgcctcaacgccgtgaccaacaacagcgaggtacagggctccaccgtcaacaccatcagcacaatattttacatagggaactctgttagcctcgtcgctgtcaccttggccctttggattttcatctctttcaaggacttgagatgtcttcgtaacaccatccacacaaaccttctgtttacatatctgctccacaacctcttctggattgtctacgcttctgtgcagacactggttaacgtgagtgtgggctgttcattcttcgtggctctcaactacttcacactcaccaacttcatgtggatgttcgtggaaggtttctacctttacatgctggtggtgaagacgttctctgtggagaacataaagctacgagtctacacactgatcggctggggtgttccagttcctataatcatcagttgggttatccttaaatcacaacttgccacaacccatcctgcgggtatggaaggacatgaattagagggactggtaaggaactgtcccctgatgcccgacagtactgtagactggatacagaaaatacccgtcctctttctactgtccaccaacctcatattcctcacacgtataatgtgggtactcatcaccaagttacgatcagccaatacggtagagacacagcggtacagaaaggcgacgaaggccctgttggtactgattcctctgctggggttaacatacatgctgctcatagctctgccgcaggagctggagcacgtgagagccatcctactctccacccagggattctgggtggcgctgttctactgcttcctcaacagcgaggtgcagaacagcatccgtcatcatatagagcggtggaagacagcgcggggattggctgaccccagacacgcctctgtcagacatggacgagatggttctcctcgacctaaaactgattgttccagttatagccggcggctgttcggcggcaagagggagtcactgtgttctgaagtgaccaccatgaccacatatgtcgccaacggttacaaccccgtcagtacccaaaccggtggaccacctcaacaacaacaatcactactgcaaccagcaccacaacaaccaccaacagctactggtcaacatctcacctatcgcaattccaacgccggtatagcccctaatagtggtggggaccctgacgtcaagtccaccgtcaaagattctttattgtaagagcccacgtgagatacagtacttggcggatgaatacactcggactctatgggaacactgagtacatagtctctgattccttcgctgtaagatgcaactcggctgtagtcaattatcaagatgtacaggaaaatgaggtaactgtgtcttataccatcttgggcgggatcaacctgtcagtttaatgaagagacgcgagattatgaggttgaaggtcgttgcagcaaggtcagtgagtcgagctgtaatattggagacgggacaatacagtaagtctcgctgagtttgctgcaggatgagaaaaatattggtacagcggtgaaaatattattaagtgagagatattttgttggaatacagaagaaaagttaacacaatctgaaaggtattaattttaatccgggaagatacaagtaaagaattatgtaatgttgaaaaaaaaattaattttacgtgaaagtacaaaacagaatgaatatagaccatgatgatgaaagaaccttctatgagaacaaactcaaagactaccgctgaatggaggttacatgatgatacagtcaatatattacacgatatgaaataaaagttgaagtaacagtgtgttttacgactatcggtgagactggtgactgtgccaatggactgtacttataactgtattttatcaagtgttggttttactgattagctcttatcatcaaagatacaatagtgaataatacagatatattttatacaactcatctattaatgtgctcaaacacaaccatataaactgctactataaaaagcctggaatctcaagtatatcggtgggataataaggcaaaaaattataaagaaataaacaaaattaatacccgattacgtttgaaggtaatgaaggaagggtttgtgttgtgtagtgaaagcttctgtgaactctgcatcgaaccataacgcactgaacgatattgaagcagggagccacacacacacacacacacacatacattcctggaccattcaattcttcttcctcacatgcaacgtcatacatttaaacaacatctacgacgaagaatcataacaataatgaaagcgagatagaaaatgtataaccaaacaaggagaaagttgtagataataaacctgacgtaagttgtggattataactctcaatgtgacacaattttacttaagacagtctcttaataagtgttgttgttgtaggaatgttaaagatgttattgaagcacagaaatacctgctgataaaataactctcggtagcacttaattaaacaaaggttattaacttagagcaacacgggagaaagagagggcggtgaatttaaacatagtgtatcatcttgaagaaacataacaagatggtggcagtagatcgtcaggttgtcattgagagactgatgatgttgtcagtgagagactttgagaagttcttgttacattgcggtcacggagaagtactgatagtcactggatgaatcagcagtggtggtgtcagtcatcagc**

**J2.** Diuretic hormone 44 receptor II (DS01-Homarus1_Transcript_14267; translation frame -3)

**cctttgactgttgcctagacacatacgaaggggtgagaatgggtgtgggggaaggggagggagtacgaggccgagaactctgtccatcgccgccatttgacctaagcatctgaggagagggtgtctgtcgatcacgccgctcagcttgggagtgaatggagtgtagtgacgtcctgccacggctggcatatgtcttggtgtgggagacgagtgtagagtgtgaagttaccaccagaggatcacctttgtaccacctattgtacagttttcttagctcttgacgtacttctccattcatcaaacagtacagtacggctacaaagaaaccctggaaggaggcgaagacctggtcaaggaagagccagacgatctccacataagcattggtggaggtggagaggctccagagcacaaaatagtgcacaccaaacagtggcaccagtactagtgtagacttacccagcttcctgtacttcatggactcggcggatatagatgatctgagcttgagaacgaggacgcgcaccacattcaggaagaagaagaagttgatgagattagagatggcgacaggggcccggatgcacacccagaagatccactgaacgttgttgacagtccagcagtgggcatcatccagggtggctctcagcgtggcccagatgcccacacaccccagtggcagtccccaaccaaggaggatgtagagggtgatggctgaggagtcagtgaagagtgccatgaagatgaggctgtggaggtaaagaccctccatgagcagccaagagtagttggccaggatgaagtactgccacacacagatcatcagcttacatgcccacgtctggctgccgtcattgtagtaagtctcgccgtcctgaaggtggaagttgtgagggagggcgataccgtccatcaggagtgaggacttgagcagcaccaccagcgcccggagcataaacgacccgaacaggtggagatgtaacaagttcctgggacacctcagcttcctaagagaggcgaggatgatgaaggagatgacgagcgtggcgagggagacactataccccaccactgacatcttcttgatcgtcggcagccattcccctatgatggtgaacctgatggtgatggaggtagggtgggcgacgtgacccacacagggggtgtagttggtccagcccagcccctgacctcctgcccacgacccattatcaccacaccgcctcgatgcccgtgcctcggctatgagacccggtacgtagtcagggcagggctgggtgatggtggtgttaggtggtgtgggcgcccaacacagcattttgtcccacactcgagggcaccacgatccttcctggtgtaggtcggaggaggtggtgttggcctcgaggagctggtagagatgacactctccctcacggcgtaggagcatctgcttgagccatagcttctgtttgtgctccggccacgactcgttaaactccccactgctgccattgattgagtcgaacaaactcaaatcgatggccaatcctgcacctccaccttcagcgccttcatcatctaaggacgaagtgtcttgtagatccaccccttcaggcgcccagctggtatcctgggtctccttctccgacacagaccaaggggacccactgtttacctcagtccccctgtctgggatgttcccgttttctgctgtggtcaccatgaaggccaccagacagatgagggcagcgaggtgaccttggctcaacatggcgaacatagccccaggtcagaggtcacggcaggcgggtcattgactggaaatggtcctccgtgtcggccactcctcgtcctgcctctgaacgtcacacttcacttggactttcagatacaagtttcagtcgatttttgtttccaggtcttgagtttagtcatttgagacgctgaagtggctaagtctctagcatgtcttgtttgttttctcttcgaatgatttcaacttttgacacacataaagtaaatagttcttcccgggcgaagcaataaagttaacgaagaagaaaaacttaacctaaaaaaaaagtttagtgtgaaactttttatgctgttccatggtgtcagttgtttagttcgcctcacactaacgcagaaataaaagtggaaagcataacagcgaatcaaatcttgctctaaggaaaattaagagattagaagtatcacgtccacatgctttttcgcgcgaggactgttgtctaattagccaaaagaaagggtaagtttcctggccacacttgcgcctggatgagtgtggtgagagggaggggggagggaggagatggtcacccctctcagcgacaacaacactgccgggcgctgggaagaacggccaaagaaacaccgaccgcgacggagtaggtgcggggactgactggtggtccggggtgcgggagaccctcgtgtggtggtggtggtgtcgcctgttgagctccggccagacccaccaccacacga**

**K1.** Ecdysis-triggering hormone receptor I (DS01-Homarus1_Transcript_30219; translation frame -1)

**gtactgtacaccctcatctattgttacagatccttaagaagacacggacacatacacactcctcaatggaagataatatacgaaccttagaaacttctaatcttaaaactacttaaatacatacacacacatacacacacnnnnnnnnnnnnnnnnnnnnnnnnnnnnnnnnnnnnnnnnnnnnnnnnnnnnnnnnnnnnnnnnnnnnnnnnnnnacacatacacatacacacatatatgcaatggttgcccctcgttatcggggatggtagcctctcagatcacttagatgtaatcttcttaaaggtgtagcagctgccgtcaacacaacaacaagagcatccataattagcgaggtcttagtgtcaagggctaacctagcaagatcgctaatactctgcagcacaagtcataaacagaataatatccaggccccctcctcactgctggcgagacagtggccagggatgagtccataagtaatttctcaatgccatttgtcgctgtctaaccagcagttaagtattaattcctgggtaattatcagccagtgcagctgatgcgttgtcctgtcctcctggctactccttgacacaagaccataatcctcgccacctcaggatttgttagttgtatcagtggcagcatctacgtgttaaatttcggcctgggtggcagactaacttcaaatacatgtataaggagcaaaaaaaatatatactgtatacgttatgttttaaggcaaacaccaagtcgcaacaagtataacatcgacgtacgtttgaaagataaatcggttgaggaaatattgatgacagtatagaggttgaggtaataacagtaaagacgtcatgatactcatcgtcttcacacctggcatctgtctcactcttgtataacaacaagaaatctttacatttctcaagaaagacaattaaagtttataaactcagctcttttaagaaacatcatttgttcactggtaacacttggtcaggattgtatcaacatggacaatacagtaataaatgcttggtataatagattataaattgtacagaggttgacttagaggattcagttttttttcagggaatgtattcagtgctttgtgtcacattaacaacatcttgcgtaaagtttgtctcgaaaattgttctggtggagcgtgtgtgagcgtgagctagacgaaggtgtccttggtattgacatgagacctgttggtggacacgaggctggtctgacgtgtgacctgggaggagttggagcccgtggacgcgtagctgtactggttactgaccacagtcttggagaacagcgtcttgtacaccatagaacagttggtgccgtagttgaggcggagattattactgacggtggggttgttgaaggaggtgttgctgtaggtatgttgccgccgcagacgacctcgcttgccacggaacacccgacgaaatgccgtgcggaatttggtagacgtcatgttgtagaggataggattgatagaggagttaatgtagtacatgatgcggcagaagtagaggaggttgtagtatccttctatccccaaggaggtgactgtctcgttagggacagtcatgatccagaagtagaggactctcatgggcaggatacagaggaagaagaagaggaccaccgtggccagcatcaccaccacctgcctcctcgctcgcatctgtgggttctccttcttgtgggtcagctcgtaagtgtccacgagcagctggcgggctatgatggtgtagaggaccaccagcaggaacagcgggaggaagaagaacaggatggaggtggcgaagaaatatgatttgatccagttgccgtgaatgggcatgatacactgtggcttgtaggtaccatcgtagaacctcgctatgttgtactgcacgatcatcagcagcggcccagcagagatgaaagaaatggcccagatgatagtgatagcaacaacagccttcgccttggtgcagcggtacccggcctgcaggggctgacagatgacgtggtagcgctccagcgagatgaccaccaacgagagggcggaggcgtggacgacactcatctccacgtacggcacaagcttacacatggtataccccaacacccagaggtcttggactgagtgtagctcgatgagtacaggcggtaggcagatgagaagtaccaggaggtcagccacactcaggttcatgaggaagtagttggtggagttcctcatgtctcggtccttgaggataacgacggggaccaggacattacccaccacacccaccactagcaccacactacagaacaccgtcgataccacccggatatactcggggaagacgggcatgttggtggcggtggtggtggtgttggtctcgctcccaaagtccgtcccgttgtagagtaagtcggtgatgttatccgctctggttgtgaggagaggcgggtatgatgacacaaggaaggacttcgggtacgagactgggtatgggttctgggaggtggcgtctagcagggcgaagctgggtcctcctggggttataccgagggactcctgaggacgatactcagcgaacgggtcctcgtagtccactcgctccatcctcgttaagattttcaacaaattcacttatttatctctttttattttctttctcccggacacttgcacttaagtatctacttatttagcacttagcgtaactttattagtgacgtcatgattatataaagtcagtaaagaacacattcccgaaataaacacacttagatgttattattcttccttttactatactgtcacttgaacacttgaacacttgaacacacccaagtctttaaagtgccaattcagatactttacaaataacacatgtttgttacagataaattacaagtatgtcttttccacaataccgtccagaatattagagagactcacacacacacacagacacaataaacaagtcgttgaggtacactacttgacacaacacagaaacaggagaccttacctaactagtccaggctccctcgctacactaaccggcgcttccctcacacctgttcaatcacgagcatcacacctggccatatccatcaccttacattaacatctagcagcagtaagaggtgatccttccatagttaaggagtctggtggtatcataatgtactgcttgcgtaccagtaacagtgagagatacca**

**K2.** Ecdysis-triggering hormone receptor II (DS01-Homarus1_Transcript_6537; translation frame 2)

**acacacaccagtaaaatattatacacagacacacacctccaaccaactctcttcactcactcgcacgcacgccccttcgccatccaggcacccaaaccaaccaacctcctgctgctgccccgtgtgtgtgtgtgtgtgtgtatgtgttaactagtattggtggacactggctttgatctgcaccgagacttaacgagtctaaaacgacgtcccaagaaaagactgacaacaaaggcttgataaactctaaagcgaaaaccacataaaagttattcattttctccgtgtttgtcaaggctcctggaaagttgaacgtgtggtattaacgaactgaacatatctgagaaaataaaaaaaactaagacatcaattaaaatttttaccaagagtgttttgagagaaagtttaccatatataaatgattataagagacaagatcataacataaagagagaacggaacgcaagggtaacagtagctcgtcccacccatacacacacacacctgagataatcttaatgaggagacaggtgtaaacagtatagactagatggtgtgtccttccctcaatgtacacacctgacagtttggtgccgctaaaaccataacaataatacatcaccataacactatcatcgtcgtcttctttacatggtgactttatggtgatttttgatgataaagttcccctacatcatcctgctctggtgatcttcgttatacctacccttaattggttttggacacctatacccctctatctctgtccctatacctgggtgtcctgatctgtcgttatactcacctgggatataccaattacaggacctgaaccctaactgaagctccaccacgttctgaaccccttggcgtgtctcacttgaacctcgtctgagtactgtacctgcaggaggaaaggcaagcacgtcagaacaccttcactactatgtcaggaaggtgacctcagtgacgatgaatagtgaagatccagaccaaagaaaacgtgagattgaaaatatgaccactggagtcacctcatgaactaacggtacacaaaaaaatggagtttggtttagacctggtggtatcgacgccatacctggagtgtggaggaggagagtgtggtggggtagtacccttgccttccttggtacctcctgcccacaacaccacctcagcccttctgccctgggcccccaccaccactaccttctcacctgcccctctaaatggttctacagctcaaggcgccctgccaaatatctccttcccggcgtacatgcagggggtgtacacagcctggtgcctggtgctcctgctggtggggctgctggggaacgtgctggtgcccctactggtggtgagggacagggacctgcggggcgcctccaccagcgtcttcatcgtcaacctggtggcggctgacctgctggtgctagtggtgtgtttacctgccctgctgtctgagctgtacgcccctcctgctgtctggatcctgccgccttccatgtgcaaggttgtgccctacgtggagttcactgtggctcatgcgtccatgttaaccatcctggccatctccgtggagcgttaccgggccatctgtcaccccctgacggccgccgccacctgcagtcgggcccgtgctgccgtcgcctgcttcctcgtgtgggtcctcgctacctccgtcaccagcccggtgatcgccctgacggagtacacccatgtacgatacatcgatcagtcgctggtgcccgtctgttacacccgtgttgacatactgtgggccaaggtgttcgtggtgtcatcgatggtggtgctattcttcctgcctctgttggtgttggtggtgctgtactggaggatcgccagacagctgttgctcgaggacaaacagctgtgtaaggacaagcccaatcccaacctccaggctcgtaaacaggtggtggtgatgctgggaacggtggtggtggtgttcttcgtgtgtctgttgcctcaccgagtcttcagtctctggttcatcttcactaccaaggagtcagaacagtccctcggtcaggaggtgtactacaacttgctgtacgccttcaggatcctggtgtacttcaactcagccatcaaccccgtcctctacaacgtcacctcttcgaagttccgtggcgccttcttcaggttggtgggtgtgcgtcgcggggagcaggtgaggtggtcggtgcagcagacggccaccaacaacagcaccgtcagcaacactacactcaccagcagcctcaacaagtctctccctccctggaagagccagagactcttagtccgttgtagttacacctgcctgcggggcgagggagagacatgccccctagtggatcgccgccccgttagtcccgctgtttggggcaacactaaccacgcccaccaacctcctagtcctgctaccagcccctttctggccagatcttcctgcacccgcgacgccgtcaggcagattgaaagtttcgtttaacctcaactctcccacgtccacccactggcagatatttttatcctgagcaagattaaccactcaccagacagcaggccacctcgagttgtataagtgatttatctcacatcctctctctcataaccttcactatctttacgtcattcacctccctaacttaaactaacctaaactaagcgacttatttaatgtaactaaaattaacctgctgtaagctaacccagagacgtcaacgcatgaatctgataccaaattgctggcctcgtgggtatgtactgtaccacaacctcctgaacaccttaacagtccagtcgttcagagacggattccacatgttcagatttatacgttaatattattctgattatattatatgtcctcgaattccgagttagggaaaacttgatatataaacatttgagagtcgtagttgtgactgaccaagacttacgatggttaatgtaccttgagacaccttcatgagtgttgtatcgtgtcagcgtcctgctcatggaggttgtaataatggatgttgtcttgctcgtatatcacctttaatactgagtcttatcctgcccacttcctgctcatgaggaacttgtaacactgtacatgggtcgtattgtaccaagtgtgagacctattaaaaatgtattgtcttactggtgacgtgatgtgagggatttgtattatgcgggtcatgtcctgcccgcgacttgctcacagtggggagatggtcctcaaatataatgtgttatgcttctgtattttgatggtatcgcaaaaactttacagaataaacatatctcttgatggtgaacaattacaataacattaaagaataat**

**K3.** Ecdysis-triggering hormone receptor III (DS01-Homarus1_Transcript_54675; translation frame 3)

**ggatgatgggactcgacaactcttcactagtagttgaggaagatggtggaaatttttccatcttggacaacctcgccacccaccctaacaagtccctctggccaactggaccagtcattcacttgggtgttgatgtggagctctttcctgagtgggctgtgggcgtgtggacaggcgtgctggtgaccttaatgacggtgggcgtgggcgggaacgtcttggttcccgtggtggtgatgaggacccgtgatctgcgatcctccaccaatctattgctggtgaacttggcagcggctgacctgctgctgctgctggtgtcactacccacggcactcatagagctgcactcgcgacctgagacttgggtgctgggagagcctatgtgtaaactggtgcccttcgtggagtactgtgtgtgtcatgcctcagtgctcaccatcttggtcatcagcttcgaacgctactacgccatctgtcgacctttgcgtgcctcctacacctgcactaagatgagggcctgcacctgtatcctcaccatctgggccgcagctgtcatgctgtcttgccccatgctggtgatgagtcaacacaagagagtgccgtatgtagatgggagtaaggtgaccgtgtgcttcactaatctgacctccttctgggctaccctctacatcaacctcgtcaccgccatcttcttcttcgtccccctcgtcctccttatactcctctacctggtcatcggcaggagcctcatgcaggactcagcctcagcagctctcc**

**L.** FLRFamide receptor (DS01-Homarus1_Transcript_7458; translation frame -3)

**agaagaataaaaaccgtaattatgaaatacaaaatttttacgtcataattttcggtttgcggcaaatgaagacataacgataaccaaaaatttcgacccaattgagggaaagctaacaagtggaggaacttgaccatatatgacacatcaaaatataagaagagtgtcagtcagtagtgttgggtagggagttggggaccgaggctgttcttgatacacacacacacactcggtcggtcccattgccacaactcacttcaagtccctaataaacactgctaaatgtggacattagtgtataatatatggttatacttgtcacagtatagtggtgagagtttaatgctagatgagacttccaaagagtttcaatatcaagaattaaaacatgtaatagtttgtatattacccctccccttatcccaatataatttacaatagaaaaatgtagattaattcataattacactagaaatactgaatatggaatcgctaaggaagtagaagctgcaaacacttttgtcaacctatgtcacaaactgtgttggtcaggcagtgtatatatactgtatgctgtcactgtgaggaagtgattttgatattaagttgaatcggctgaagaattttcgttagtgaataatattgtgttcattataaatgggaagtctgcaatgcatgggtattagtataatgcgttacactatggcaatatatcgggcatagtaaaatagagacttttattacaaagcttatggaaggtgtgtggacagtaaggtaaccacatcagagtggaaggttagaaagtagggggaagtgatattaggaaagtgacttaaaacaataaaggaagttagtactttcctttcttcatagttaacaggtacttaggtccttcctgaggacaagtgtacctcctgaggacacatgtacctcctgagaagaggtgttcctcttcatgagaacaaatcgtacctctcaaggaaaggtttaactgaagctgtagttgatattattttcatggtttacaggtatatatctcttaaggagagtgacccatggtaaagtttttatagagagagagagagagagagagagagagagagagagaccattttatacattaacatggatcaccttccagcaacaataaacacagacttcatcacatacacatggtaagtcttgctctacctgccccaccacacttacaacttttcaatcttatatctatacaatatatatatactttttttttttaatgctcacttacaactatgatgggatccaagtggtcaacatggccccacatacagtacatctttaagttaacagtgattgtcaataacctcaagtgtgagcacaggtaaggatattggctatcaaatacagtatattatgtagggcataagcacatgcacgcacacacacacnnnnnnnnnnnnnnnnnnnnnnnnnnnnnnnnnnnnnnngtcatccttcacttctctcaaaactactttgtatgtaatggtgtggagcctcagtcctctccacagtattacagctcaaagtattttcctcaggttactttactttgacagtgtttttatttattttgtttaataatttccacttaatattgtcttagttctttacaatctcattcaactttgactagattaatagacatattctataatttcacttcgtttctctctgggattatcacattatacaaataagacaaacatgataggtttaggaatggagataattatactgtacatgttgtgtgttaattatgcttacattaacattagtacgcagccgtcaccaggagtgtaggcgccataaacatacgaaggataatattctcctatttcttcagtcttatagtttctccacatacattattaggctgtgtttgtcgttctccattacagctgttggtagtgttgcttctggcttgcagggtacaggttatctcatcaccaattacagaaatgcattcgaaacatgctgataatatttctggttgtatgatcttaggaaagcttgataatttttttttttgggggggggggcctttctaaaacaaatgctaaaattctagaaaaaccttggaatgactgaattgttgtctatgtaaatatgaaataagtctttacatttcctgtttggttcactgcagacacacatggtagcgtcttttactgaaacttctgagtatctgtgtaattgctttctatgtacagtatgtcacactcatagcaacacacgatcatgagtcactaagcaactaggaatgttttcactgtcaacaaatccgggtctaaggtgaagaaccactccactggtttagggtggacgagaggtataggttgaaagtacctcatatttgtcctcagccttggaagttgtgattcatttctttgatggttctcttagctagacacatacagctcttattttttatcctcttcactgtaatttgttttgtttcaagttatatcatggaggcttcatgcttcacttctttcaggtttttccaggcttcatattcatatcctccccaagttgttatcagttgtttcccctcccttagatgcccctgtttccaccacctatattctggttcagttttctggggatcctcacgctggggaactatttgtagagcctctgagaaatcatgttacctatctactgaggaaacatttcataatgaggtgatgtacccagagtactgccgaggtgtgctactgagatcgacacttcagaaggaggaaatcttgaagaactgacaacctaacctgagaggtcaagagcatcacccacatcagcccgactaatcatggaggttgtttcacagagcaccacagaatgtcacacttatatgtctacagagttcattctttaaaaaaatagggaaattaatggattaaggagactcttttctgtaagttcacagctctctatagaggaatacagttcatgctacatgagaaagttgtcttctctaaacttttaaaccggcatccaaaagtgtacagcgtaaagtgtttgtgccaccacaactgatatgtaaactttgcgacacgttaagatgctgtgtcctttattcagaggacgtggacattctctgaaggggtgcgctggggcactaatgatgggcttctggagggggtgaaggaagtggaggctgtcctaggcgtggaggagtctcctaagggtatgctggagtagcgagaggcacgccaggaagtcccgctgcaggagtggccgtggtgaccgtggatgtcatgcgggtgtaatgatcgacctgactggtgtgatccgttccaagaagacagtcgaatagtctgagtcatcttgccgttggtcaaggttcgagattctccgtaaatactgttgttgccgtaggctgccgagtctagggatgcgtcccttcccctggcacaaaacaggaaacccgaacaaaacatcctcaaaaacaacttcctgaacttctgtccgaagatacagtagatgatgaagttaacggacgagttaatggtgacgaggaggttactggacatcgtcagttcagcaatggtgatgtccagaagctccaagacgtttatgatgaaggcaagaacattgcaaacgaagaacaccgtgacgacgaccagcaacatcactgccagaccaatctccttccgctgtagtcgactcagctgctggcgctcgtgattggctgcccggacctctttatagatgaagaagttgaagatcatgaggctaaggaaggggaccaggtacatgactatcaggtaaacccacatgatgtagatctctatgtagtaaggattcagacgcagcgctgacggtaccacgatcacaaaggtctcgccgtctacattacactccttattagacacttcccagaacctcggtatgttgtacaggatggagaacagagctacgctgatcacgtacacctttgcccgtccatatgtacagagataccttgcacgtagcggccgacacactgctatgtaacgctctactgtcaccgtcactgtcaaatataccgatcccgtctgtgccactagcgccagagggaagacgaagggcgtgactctctggtagacgccctcagagtaccataacattgtgtgtgtgtactcgcagatctccggcagaccgaacataagcacggatgtggtggtgacgatcatgtcgaaggaggtgaggccgattagacagcagttgatagacgatcgcatcttgggcctagacaatattgtaatggatatgagattgcctgccacccccagcaagccaacagtggttaacaagaaaccatacacgatgaaccgcaagagcctgggtgatggcgcggtggcattgtaagtctccaaacacattaaatcacccacttccatcaacgtctcattatccattatggaaccctcggtactgaattcttcctccatggtctccaagagggtagtgttgagagtcatcttggtggtggctgtagagtagagagtcgtcaaaacaaaagagactccataggtagactgccaataggatctaagactctctctagctcatcactctggtgggttaaggtggcggtacagtgggtcaggttacctcacggcctatcacgctccggcctggcctcacgtccctccccagcttctgtgaagtcctataggcgagacaatccttcactaaagaccgtttcttccctgtttgactccacgaatctgcgtgggggtgatgacggccactgacagcagaggtggtaggtggcgaatct**

**M1.** Insulin-like peptide receptor I (DS01-Homarus1_Transcript_11179; translation frame -2)

**tttaatctacatcaagtgaatatttgcaacattgaaaataaattgtcttataatgaccatcaagagaagtcaaaatgcacatctgctgtcagtcgcttcaggagtcttgtatattaagtttgtaccccgaatgatgctcagtggacctatctacttaagtaaacaaatctactaaagtggacatgtgttctaaaagttttattgtaattctgacgccattttggacataattttctgatatatcttaagtaagcctgttagccacctctccatgttctcatttacgataatatcttaacatacaatcctttgagaatacaagcactggccacaagtcacagcagcaatgaggagagtacagattacgacaagcttatgctcaccagggtaccaatttacccttacacctcaggtacaaatgtgggacacagcctagcgagagtgtagtatcggggaacaaatctgatttaaccattaacctaacctaacctagcccaagttaactcccacctaacctaacctagcccaagttaactcccacctaacctaactcaacctaatccaacctaccaagcctaatctaaagattttctaatgttctttagttatattaagttaaggtaagctaagatgggccaagttagtttagtttagatttagttgggttaggttaggtctagattatgttaagtaaggtaaggtaaggtaaggtaaggtaaggtaaggtaaggtaaggttgtgtactgtatattaggtaaggttaagtaagaataggttaggtttggttaggataggttggttaggtacggtttcaaatctgagatgagcaaagccttaaatctacgtctttggaagatttccagcatcacaaatgatctagatgacacacaggtacatgaagcagtagtccttggagtactacaataattctggcaaatgttcaaaactcccagatatgatattgaagtatatataataataccgattagaaagtagcatgactgtcattctggcctaaagctgctgtttgatgcacttcttagtcggggatcacctcatgggatccaagcgtggaggcccgccacacttaatttttcccacctctcgagttttcaaaacttcgaaattgatagaatcgtcgacagctactgtagtgttgaggcagggagggtggtggtggtcgtcgtccgcgttaagtgaggcagggggtgtggtgggaagggtagcgggaagagaagtggttgctggtcgtgatgcaggagagtggcccacacgacatcctcttgtaagggtcatcttgcgtgagacacagcatgtcatcatccaccacggtgtcatcctctgtgccatggtcgtcgaggttatggagagagtcatagtggttgtggtccagagaggtactgaaggagtgctcggcgtcctccaggctagggccaagggaaccactagctatggaacctttgtcgttgctctcggtgctcttagagtcacggcagctcgagctgtggaaaaatgacacccgctcgaatcgcgcctgatagtctggggaagccctgggcaacaagagacgcaccagctgaataaactccggtcgttccttgggttgacctttccagcactgctgcatgaggaattttagaatctctgggccattctcaggacattccagtcgtgatccagatataaccatgacgttcactacttcgtgagcgaatccctggtagggtagggcgcccctggagactacctcccacagcaggactccgtagctccacacatctgaacgagaggagtagcgtccattctgcagcgactctggagccatccatcgcactggcaggaatcccttgtcagacttcttgtagtagtcagtcttcaggtacctggtgaggccgaagtcaccgatcttaagtgtgaggttagtgtccagcatacagttcctcgcagccaggtcccgatgcaccagcttcttgtccgctaagtacgccatcccgtctgcagcttccaacgcgagctccaccattttcatctcggtaagtgtgccgcccgcctcagtgagaagaaaggacttgaggtcgcctcgctccatcagctccattaccacatagatgggagaaaacttgactaccacacccaccaggcgaaccacgaagttggagttgatgtcctgcatgaaaaccgcctcttgcagaaaccttttaacatcccagccggtggcttggttactgagagccttgacagccactttgacatctgagccagaagtcactgacaggacacccttgtggaccgtgccgaagcatcccttccccagctggtgttccaggttgatctctagagcatcacgctgaatgacatattcctcctttatgtgttgcagtgtgtcacccaacagcggctcttcacagttggggatgtgattcatgaactctggggtggtgggattagctgcttttcgacgtccccacaaacagatcgtcatcgtcacaaggcagaacaacaccatccctacagtcagtaaggccaccaagaagggccaataccagaagaattctgagatctgtatggttctggtggtgtaatgaccatcgccaccaagggagcggagtttaatactgaagacataagtgccaggttgaagccccttcagacgataggtatatccttccctccgtgcctgctcctccatcaaacactccatcagtttgctcttgccctgaggagcttctttgtgtgagtactggatgaggtaagcaagaggtgggccattggggttctccggaggttcccatctgaggaggatattactgccagtggtagtggtggtgctactaatggagagagaggggatggaggtggagagggtggagatggtactgatggaagatgagcgggtggggggtctactggtggaggtggaggtggaggaagagggggttaaggggctactaattgtggagaaggaggaggaggcagtaatggcagaggtaatactaaagaagatggagtagtcactggtgttagtctcgctactgttctctgagacgatgctgtagtctgaaaaattctggccatgtttctctctcagttggttctcttgctcacctgaagtgacctcgcttggctcttcctcattggccgaaaagataagaatcttattccttgactcctgaaacagtgtggcagattccaaattcgtccagatgctacacaacgcttccctattgactggtgtgtggcacgcccgcacccacaccctgtagttggtgaagtggtgtagacccgtgatgttgatggtggtggtgttggtggaggtccacttagtgatactttctgtaccattagtagagctgagatggggtcctacatatgtatgaccaattttcgaccaggacacactagcctgcctcttcacccggtcctcagaacccgtattagtgttggctgatgcaggagggacgacttcttcacgctgcaggtgggaggctgaagcaccggaccaggccaacatagtagtactggatgacctttcttcatcttcatcgtccagttttatatatattaagcttgacatatagtcatagaattctatgtcatattgtctgtcctccttggtaactactgagtgcgagacaggacaagggcagcacttaggagcgtcctctcctccttcctgctcctctgtcctgttggcatcaggtgccaccgttgttgttgcttctttcctcccactcaacttgccaattcccccaggaggctcgtagtcatcactacacacataactgcggttataatcagaggaggtggaggcagggtaacgcaccacctggtacaggacttcgtaaagtgcgacccggccgttgggtgtggacggctctagccaggtgagcaccaggtatgaggctcctttctcctgcaccttcagcttagtcactggggacgggtcggtaggaggagtggtgatgtagtggatagcagagcgggtggcggttttctctgtatcgatgttgtgtacggagacgtaaagggcgtagcgggtaaagggtctgaggcccctcagcatcacgttactcctcttcttctcaaagttaaggaagtatcgctcccataacagtttatcattacaggcatcgcggccctggtagtacgtgacattggcgtcggtctccctgtagtaaacgtagtagccgatgacataacggtgatcgaagactgtgttatgggtccacgacacactgatggtacccaatttcactccaatttgaatctcaatagacaggtcagtgccataacatggtgcgacgtcctcattcgagtcattggagacatcatcaggagagaggagagtcacaccggtgaggttggccagttggtggacgagtttggggcagagaagcgggttgtaacgggagaagagtgtcccctgggcgatcacgagggagtggttattctgtgacatgtcccacagctcctgtaggttctcgttctccagaatgtacagcgcgtacttgtcatgtacccggtcctcgccacctattaactccaagttcctgaggaagttgagggagaagagcgtgttggagccatagacgcggatgtatcccgtcaccacccgcagctctccaaggttctcctccagctgctccatcaagttccagccgccgtccacagagatctgcaagttgccctccacccgctggcacccacgtagccaccgtccagggtcactactggtgatggtcgcccctttacacgtagtggtgcagtcaccatgacatcgcacacatacattctttgactgcctttcgtcggcgtcctcaagggtgtacagcttccaaccagcactgatacaggactccctattgatgcaccgataacccatatggtgtacggggttggaggaagtgggggaacaggcagcaacacacgtggagccatcctggtaattcctgcaggcgacgcaggcggaggcatcttggggcttcaagcagcctcccacacattcatcgtggcactcctcaccttggtggcaggtaccgtacgcggtacagcgtgtgtggaggtgtgtgagtttggtgacacagttgcctcgctcctcacaggggttatagatacacattccgtagttgtcctctatgacattgtcgtggtaatcggtggtgagacttcgccaggcattgtccattccgggacagaggctccagttcttcgccaggcgcacagaaccttgcaggatgtgggtcaaattcgccagtcctagctcctgcaggtagatgttgccaaagataacaagggcgtgtccatggaagagcgtatcgcccctgatgacagcgaggcgaggcagtaggtggcgtagagtccttaggtaagacaagcgatagagcaacacgtagcctgtcacctgtgttaggttaggtaggccaaggtgaggccaggtaagattacgatcatcgataagctgcagtcgtagatggccctcaatgacgctacacttctggtactgctctaactcactcgcgttcccagtgatctctatcgtctcacaaatgtgatggctggagtcttgaggctggatgacggtagctactgagcctggcaccatcaccaccgtcagcaccagcagaggtaccacaccctgcaggccacacatcaccatactacggcgacactcccgactgtacgatttttgacgacattcaatgctggtcgtcgcaaacagacaatcattgctgcaagctttaaagtgttccgtcatcattctatggttcttgagagattcgttgtgggggtaagaggagctctgataacagggtaggccactagacctctgtgagtacacttcttttttttcgccactgattggtctgttagtcgtttgggtggtcggtcacgtttctgttacacacacactgttggtaagtactaggcatgtactggctccagaatgt**

**M2.** Insulin-like peptide receptor II (DS01-Homarus1_Transcript_3743; translation frame -2)

**acacacagacagacagaacataaaaggaagacaaaactgtttcacttcaatgacagtaaagtcaagtggagttgtcaatgataatagtgtgctctcagttattgcatcattgttagcttcaaaactaagtcaaattcataagatattccaggaaaacacacagaatctcctctctcattttctttgataaaattgcatactggtttgagataacgccaaaaaatattcacttgtaagagagagagagagannnnnnnnnnnnnnnnnnnnnnnnnnnnnnnnnnnnnataatcagagaatggttaacatacataaattggtcaaattatttgcaacataaaaattattctttttaatgtgaagtatgatgcttcatgatataccagagataaataagttgaagaaagttaccctgttacagtgtcaagaaacaaaatcttgaaaaacatgcaagaaatgatggtgaaatatataaactaaaagctttggcattgtatttagtaataaactaaatgcttaactttcttaagaaatcagcaaaagaatgctctaagaccccaagagctctgtattaataaatcacatcacaaagtcacatgatcaacaatacctatagtgaagaagggggtgagaggaatattgtgggccaggagctgcagaattgcttcaatacacagcaacaactgccgttcacccgcgaggtttcatcttcagcattggcacatgatagctgtcttaaaacaatgattcatattgcatcagagcttgcatacgaatccccttgcataatgagataatactgatagacgccatctgcagtgttgatacccacagcgcaagagtgcgagccattatcagcaagcatcttgtagtgctttataggaatatctacatcatcttgagtaacaaaattttataaataccacttaaataatataaaatagaatgaccagaaaaagattagcatttgagtacatcacacatgctttgctgctaagatctggtctagagttcctcaatagggaacttcaatatatagcaataaatctgcaatctaaaccactagaacactactttggtcacttaggtatgcctaggttagcctttccctatacaggcgtgacattagtccataaatcacgagatatatcagtggtgcagtagtccccctgctgtctgctcactaaactttctttttctttctccttgtgttcctttggtgttaaagacatataccttgaggagacatactgggcagggggcagtaaagggtcctctcccatttcattttcttcaaccatcacatgatttaaattaagagcgtccaaattggaatgcgcagatatcatgccatcttcgcttaaactcgttgtgaggggtggaatattcatcaccatatttccactcattgaccgctttctgctcgaggatgccacgttctgcaggcggttgttaatcgacagagacaattttcttgtcttatctggaattctcatctccagtgacacagtgtcttcaatctgcactgaagactggggcccatccagggacggtgatatcagtcgtgggtaggcgatcaagtgctcaataatttgtaacacttgtggccttcttgatggcacacgatgccagcaagtcttcagcagcacatccagctgaggtttaattcctcttggtatggcaatagtatggccagccttcacatgctccaacacctggtcatttgacattccctggaaaggaaatgctccaaacgtgacgatctcatacagcaaaacaccgtacgaccaaatgtcactcattgttgtaaagacgccttctgtcagggattcaggcgccatccatcgcacaggcaacatacctttacgattaaacttgtagtaattgttttcatacattggccttgtcatgccaaagtcagcgagctttacagttctgtttgcattgaccaggcaattccggcaagcaacatccctgtgaacaaatttcaggtctgccaaatatgcaagacctcgtgcaatatctagtgccattgaagttaacctcttgttgctcacttcatcatcatcatttcgattcttttcatttaccaaatttcttctggcaagaagatatactttgaggtcaccatataacataaactccatcactgtgtagataggttcagtttttgtgcagactcctaagagacgaacaatgttttcatgttcaaagtttttcatcatctcggcttcagagagaaaatccaacttttcagatatggtagaaccaaccttgagcgtcttgactgctacggccacccatccctcagtatcaaactggcactcccctccatacaccgttccaaatgctccttcaccaagcttgcgattgatgacaactttgtctcttggaatctcccaaccatcgaatatctcaaacaatggtcgccgacgccacaattcttgtatttgttcaagtttcttttcatacctcctcttaaatatgcagaagcagatgaaaagcagtagtatgaggccgccaaagcacagcgtacacaggacaattaaggcatatgcacattccagattcacaaactcactgaacgcctgtattgcacatgtgctgccatctactggtttttccccggttggccatagaaaccgatccttatttattttgaagttgaaatcaatctccttgtcttctccagtgtcagatactgaaggaggaggatgaaagcggccgacctcaacatactccttgttctgaaactgccacactatgatgtccgtcatccttgaggcaccggaggaaaagttgatgtgaccagacactccattaaaggatattgctgatatctccttcatgaatgccccaatggtgcttgttgctctaaggtcagcagcgtacgatggatccttcttgaacagattatccaaggccagagcgtaggtccacacggcgtcgtaggtaaagccagcatagtcggactctgatgcattttttgttttaacagtagtgttaattcgctgactgtaaagtttcctccactctgcaactgttattccttcttgcataatgtcattatcttcagcaaaatatttgtatgagagtgacatgtgtccctggagagcgctgtgcatctgagccgtcgtgcacttggtgtaacttggcaccttgtctacatcataccagtcggccgagaaccagtgggggaggaaccacaggtaagcctgatggccagtcatcttcagatggtaggcgtcgcatataacttctcgagcaacatcttggtaaaagtcgccaatgatgatgtaataatttttgttcttcaagtccaggagatactgagtcatgtcaggtgtctttctctcctggggatacttaagggtttccagctcaataccatcttcctgcagtttatcacgaagaagcgtcaagtattctgagtacctctgtccattttctgtgagtgatgcaactctcttccacccttctctcttaaaataggaggagtacacgtatcggaaaatcttattttctggaatagtcctaaaaaagtatggataatggtcttgatctgaaaatatggcgccttcagcaccatacgagattattggcacattgaagtattttgacacaccagcgattggttcaactgtatcagaacatgctggacccacaaacccaatcatattattgtagaatccctgactagaagacatttggagaatctctatgaactgcctcatcaccatggctggctcacacgctccgtttaacacaatgggatgaatttcaaaatcagtaaggatgcttttatttttgttgacggcctcaactgccatgaaatacgcaggaacattgccgggagcgataaacttgcgtctgttttttttttcggtggagattggaaagagaccgaccatcttcagttggtgcagagtactgcccggttgccatgtcaaccatcgttgcttgttgttcttcatccagtgacaggcgacctcatcaagagccaaggatgacttgtttttccacatgttgtaagtgtccaagagatccatgaagtctgtgtggtttacctgcaccttgtggaaggcattatatgcaaattttgcatatctcttcaatttcttccataaaaatttatggaaccgatgcatctcgtatttgcactgatatggagaatcaccatcagctttaatggttctgtctatgcacggcggaaaggaaacagatataaagtcaaacggctggagcagcacagacggccaccagtggaagatgagaattgaacgttcttcttcaccctcaagcccggacagatcactgttgttctttatgaatttgtgattgaggtttggtccaatccaagacacaattacatatgctttcatggcctcaatctgaccacgaaggaactttgtcacattataatctgcatatgaagcaaagaggacagcacatggttgatgagcactgcatctagagggaacatacaggccatcttcacaccccagactctcggggcagtaaaactccccagttgttgggtctctcattttaaacgacagagtgttcaattcatcttgagtcctagaaaacagtttgagagttggggaatttggactggtgaaggacctccagtgctcaacgatgaaatcaggtcgcagtaacctcttaggcatgaaccacccaaaccgtccgcctgatccaaggtagctgctggtctcaaagtctgtggtgagggactccacgttgtagcctggcggtatccacacctcgaggctcaccatggctttaggtatccttacctccatgctgctt**

**N.** Leucokinin receptor (DS01-Homarus1_Transcript_14169; translation frame -2)

**cagacctacatcatagcttcttcggcgggtccttccattcgatttttcctatttcctatgagagtggatcctacagcaaggaattcactccattccttctatatcatatccgcgtgtcttgtcgcgtactttaggattccataagagtagtgaatatgaaaatgtatcctacacagtctttttttttcaaatttatttcacttcagtgtctctatttacggcaggattcccagtgggtggttagaatgcgagtgacgtaatgccttctccatcctcgaggtccctgacactgaaggactcaggtcactcttcgagaatacaaatgagagtcaatcctacacaagactacaccatcccttcattgtcctgtcgtcccttctcatcctataatccgaataggactctgtatagagggttgggatgaaattgaagcttacgttaggactcccacttatagtatcctatataaagactttcactccatttctcttcatatatttacaactcctctcactttaggattcttcatttcttctctcactcaaggacgacacttggagattgagacgtgaatagatcctacggagggaccctactcaattcctttaataatctcgtgtccccacaccttccaggattcagcatagaggattagaatgtgggtgaatcctacacaagattcccattccaatcctaattactttatatcctgctgttacctcccactataggactctaacaggactcaacatggatactggagacgaaagcatatcctacatactgccatcccctggtgcccatcactcatactctggtgcggcctgccaatgcaaaaactctacatggaggcttaggacgggagagaatcctacacgagactccacaccccatcccgtttatatcctggtgtctcctaccactctgggtcgatacacagaggggtgaaggtgagagtgggacttgagagggacgcagacgtgtacttcctgatggtcaccgagggactccccgttgatggtccggactcctccagaggtggtggagggtcgtccttgaacggtgagtgttgacttgtgcgaggaggaacgtgaggagccgttcagcatggtagcgtcagtgctgcgagatatccgctggtcggaggctgtcgaaacttgccgttcaatcggatacctgaacctgaaggacatctgatacctggaccgatcaaactcgctcgtctccccagtattttcttccttaaaaagccatcggaacttctgccggaactctcgcttgaacttctcgttataaatagcatatataaacgggttgcagcagctgttactcatagccaaccaatgacagcagaaccagatgatattgatgtaccggtaatcgttaatgcctgggtagatttcctgcaggatgtggtatgtctgtagaggcgcccagcagaaggcgaagagagccaccacaataaacagcatcttgatcacctttttcttgttcctgaggacgtgggcgtccctggcatcctcagcgttacctggcgtcctggcgccccacagctcccaacccatcctgatgtaggcgaaggagatgatccccagcggcacgaagaactgcagccccaccattacatgagagtaggtccacatcactacggggtcgatgccccccacgccacagaagggtatgtcctctttcgtccggggatccacaatgtacttcaccctcagggctatggcattagggatggccagggtagcactgaagagccagatggacgcgatcaccaccttactcctaaacttggagggtctagcgttgagggggaaaacgatggctctgtatcgatcgacggcaatggctgtgagggtgaagatggacacgttgacgctcacagtttggaagaagggacagaaggcgcacataaactccggcaggttccatcgctgtagaagtgccgcctggaactgaaagggtatagcgaatagacctataatgatgtcagccagggcgaggttagctatgaagtagttggtgacggagtgcatcttgcgggaggtggccacgatccacatcaccagggcattccccaccacggctatgagggagatggacccgtagaagatggagagaagcaccacaatccccgtcggcacctcgtacagcgccgtctccgtcgagttggtaatggtctctgagttgctggtgactctctccgtcgtgtcaaccaggaaggtgaagtcggtgaggttggtaaagttgatgtaactccagttgatgtttgggtagctgagggtgaggttctgtaggaggtaaccgtaggggtcgtcgtaggagagggacatggtggtnnnnnnnnnnnnnnnnnnnngtggtggctatggcaggtgctgaggagtaggctgggagaggtgaggaggtcttcaggatgctaggtagtagaggggcaggatcagggttcatctctcactattgtctcactgtgtcaagtgccttattcttcttcctcttctccgtgaaattgaccaaatataaaacactttgcatcatattaattcagattgtgctttcagttcaaggtaatctattacaaatgcacaacacttgtaataccactaatacgacacttatttcattcataaacacgaggagaatattagtgtttacagaataaatagtacttatcttttcataagcgtctattcactaaacactgtcaccagttctctcgtgtatgtctctccctgatcccataccttattaagagaccatagaatccaatagtccttttatttactattttctgattcttcttactccaaattttcagcctcgcgggtttgagtaagcgattaattgcctttatcttaaggtttttttttcacatcattaacaaacacaaatacacaaaatccttcaccaaacaaacagaaataaaaacaaaatgattacgagtcagtatttcagcacaaaccaccaggggaactatgagacaaaattcgacgtctactaaatattttccaaacactggtcttgatcatcgtgttgtcttacggtccttcagtagttgagacccagctggcagtactgtacactacctcctgtctctctctaacatcatgcactgagtaacttatgaatgaatttccataccataacttatatgtcctttgggttaatacacgagaatatatataaatgtctagttatcattttatcgttgtatatcgaactgtcaattactatgagatgaaaattattgtttagtcactcacaggtcgtgatgcactcaatgtctgtcgtcacaagtgtctcattgaggatatactgacacttgtagatttaatgttcattgcattaaatatatctgacgtgtacgagaacccttcatccttggtgagtcttagatattccaggaagtagtcttgaggattatgttacgtgttctgaggagaatcttattggtgggtcttaaaaactcgtaccggaaggtcttgatagagaggctcctgtagaaggtgtcatcgggtcttatactgtagaaccatcagattctccttgatctctcccctttttgagtctaagaggctgagtgacggtgagacacgcctttgatcctcagcttctgttgtcgtatgagtcgatccttcaacggcagaccaataggggtgcggtggccgagtggatagagtactctcctccagtcctaagagtcctctggttcgatccctttcacgcagctcaccggacgaaaataaatatatgttgtggtaattatttgtcgcttttcttctttttcttctctttatcttctttcagttatcacgtaattatcatcagtttcctctaatttatcaaactttttcccccagtcaatgaggaatattaatggtccggttactcacacaatataatataaatattctttatttcactttatttcctgaatcacaaaccaacatcatagtcgacgtttttggtcatatcaattttcagtccccaacttttcctggtcttccacgcagtacaagaagagtgtaaaaagttttgaagaattattcgtttttcccgattttcattaagtaggatcactaaggtcaaggtgaggccagtgtagcgcggtagacggaaacggagggacgacgcagcctgacctcccactacaaggagcga**

**O.** Myosuppressin receptor (DS01-Homarus1_Transcript_42439; translation frame -1)

**cgtctttcatctaagtaatgttccgtgttattctacagtagtagggagttcatcattacaccgtctttcatctaagtaatgttccgtgttattctacagtagtagggagttcatcacaggaatatatacaacctcaactcgcaacacagatccagtactgaatcaacatattatcacgctgtggatgaggaataaatgtcccaactctttggtgtaattctaaagtcgttatccttgacagtaaatgtatgttcaagatggtattcagatcatcagtttgttgttcctttgttgacacttttagcgcattaactttgacaaaatgtttttaaggttaaatttatattatgatgtatataatataaagttatgtctcaattgcagggagtttatcgagcgtgaaaataatgaaaactcgacaggatttctacgaagtgttgttgacaactacggctgtattttagacttatattacataatagtttacgagtgaaaggtccattatcttctattttcagccagtgaataaaatgtcaacaaaaatcattcttaaacactgtcagaaaaacatggctcctcaaaatgcaacttgaataaatgaacggtaaaagctcttggcttggtgcctgagactaatttgttcataaatgtttggggatacaaggagcggcagcagcgcgcactgcgaccacacatgtttagttgaagcacctctgtgtctcagacatgtgtgatacatgtgttgttgctctccaggcctggatctgccgagggtacggccttccagcttagaatatatcgaggcgtgcggatagatagcaccgacacacagcacggtttgaacaggttgctgaaggtgtccctgaactgttggctcatggcgcagtatagcaggaagtttatggcgctgttaaagagcaccaacatgtccatgatttccccgagtttttggtagcaagggaagaactcggagcctagaatgaccgtcagaagtccaaggataccttgtggtacttcagaggccagaaacagcacaagaatggctagcaacatttttgtaacccggtcggcttgtcgcccagcgtcagaggcgcggcctgcggagtttcttttcatgagctgtgtgcgtcttcgggcggccaataataattcctgaataagagcaaaagacaagccggtgagggcgccacatggcagcaacttgagtattactgcgtaaatccaaaagttgatgctcttcattaagtcaccgtgcgcctgtgccagttcactgaagcctacaatatataatgtctgaccattgtattctatcgtgctgatggtgaagttgaggtagttgggaatattgcaaattaccgaacagaagaaggagactacgataattctactggtgcgctgcatggagcaccaggtggtgttgttctgcggaaaggcgatagcgatgtagcgccagacggccaatatcagagtgaggcagatggagagggtgtggaagacttgagcaaagtgcgcgtggaacaggacgaagacggcccagccccaggagtactgtgaggtgagggggcggccctgccacacgtactggtgcatggtgtaggggatgtactccaccatcactagcaggtctgtcatagccagccccgtcaggatagcgttggttggtgataacatagagcgcctcgtcagcaccaccatgttgatcacattggcgatagatccgaagacacacactaccagcgacatacagccgtgcaccgcctggtaactctcccggaagtggttccatccctctgtgctgcagtatttctcattggcggcgagatctggctgtgtcgcgttttcagtgtcgttgtaggagtagctgtagtcctcgatgtagtcactgtaggtacttgggtcaatggtagcattaaccagggcggtgatgaaggtcagtgttactgcggcatcagggccccctgctgcctccacctgctccatgacgaggttgtttgagggtcaggaagggctgcagtaagggctggccaaagagaacttactactataggactggcaacaaagtgaaggtacttagaaatgagggagaggtaaaacaggcgtgaatggggctgaaaactgtgtctacgtcatatctaagatttatcaattacacaggtaaatttctcgtgtttattaaagagtaacggcgtcaattcacaaacaggtaatgaatgtatactaaacagtaattcacaggtagacactagttttgaacaagtgacaaggggaaggttaaaggttgggggaagtttaacaacgttgaagcttgatgaatgttagaagaatattcacagacgttttgtgaatatgttagtcttatgttcttgatccttctttatatttgttgatattagttcatctctaaacttttgtgttaaaaatccactttatttttaccagtggaggacttatatatttattatctgatttgcaatagtaagaggaaaataataagttttcataatccattttacattcaacatattagaactggtcgcagagggaagtaagtttatcaccacttcatagatttttccggaagtctatataccatcactaatttcaccattttcactatttcttccttgtatttctaaaattttaaaagatttaatgtttatcgtagctggggacaccatgccttcacctcatattaaatacggctgaacaaagggtttttacatacgcatgaataaaacatacgagtcgttctttcgtcattatttgctttggagttgtgtcctggattcggatttattggaaatatgtaaaaaaaattctaaacgtgaaggtcatgtgaaaactgttaaactatcaaggagagtctttccatattaatcagccccttgaaggtactacttattaatattaagataagataatataaccactttgaattaagctgatttaattaaacttgcgatactcaagagaatttttttcgtctgttggtgatgttgtatcgtaaattcttaactttttttctcgttttatctatgtcggcttcagttaacattatatcataaagtatcacagccgctgcatgataatgtcattaatgtcctcacgccgttagtgttgtgtcagtaccagttcacctcactgttggtcattctcccttatcaatcactggacctataggcaacacctcttaggttctcctcgtcagttacagctcactccctagtctctgtccgcgtcaaccccaaaaattcaaatttcttccttcaaatatctatgttcccggaagacgtccacattaacatagagcaacaggctgccacccgtctctgaaacttcctggtgtcgtgagagaattataaatctctgagtattcgtccgaaagttttctaattcatgaatcactatagccacagagttagattagttaaggtttgttagttatctggagactttcaagttatgggggagtgacggaaggagtatatgtgtgtggtctgtcaacgaagtaggactacacacccagccagcctgcccgctccctactgt**

**P1.** Neuropeptide F receptor I (DS01-Homarus1_Transcript_40414; translation frame 1)

**cgtccgccaccaagagttcccgtgggtgtggctgtgtgaagagcgagaggaggagaaataatcgttcttggagtccagttttgccatcatttacatgtactgtacacaggcacacacacatgtatatatacaaggatggcttcccagacagtgaaaagtatagacaacataaactaatataacctgaagacgttattgagtcccctacgtaaacagcgcgtcgatacgtcaaggttaacgtcaaggtaaacaatgcgtcgatacgtcaaggtaaaataaacaggtctctggtgcatcacacgagaaaagcaagtttcccataaaactgaaaatatcccactctgtcaggaccagaatcccagtacaggggagtattaccaaggattgacatcatagctaatggtaggagtttcgcggagctgaaaagtaagttgtattagctagaatgtgtttagctaaaatgtgtctctcacatgtgatcagttcttcgtagtcaagcaaaacacgtttctcacagcaaacgattacgtaatgggagctagaatatgcttcccacaactactgaatgtgcaacaccaacaaaaactagagtgtaacagctgatgagggagtagtatcacgctcttacgctcccaggacctccggctacagtaaaactaacagtacgaaagcgatcaacagtttgtagcagcagccatggatcagggtccagatatctcagtggcgagccaggagctgccctcggatggctttgggtcgccagagattagtagcctcgacttcctaacgctagcaaacttcgacaataatctcaccaacttggcgcacaacttctcctggctattcaacgtgtcagagggccttaacatcgacctaatcaacaagttccagcgaaataggcgggtgaacgacggggcctactacgccctcatcgtcgtctacagtctactcatcgtcctaggctcgacggggaactccctcgtcgtcgttgccgtcattagaaagcccgctatgaggacggcgcgcaatgtgttcatcatcaacctggccatttcagacctcctcctgtgtctggtcaccatgcccctcaccttggtggagctcctgtcgcagtactggcctctcggtgaccaccctttcctctgcaaactggtgggcactctgcaggccacctccattttcgtctccaccatctccatcaccgccatcgccctcgacagataccaggtgatcgtgtacccgacgaaaaattcactgaagactgtgggggcggtgttgatgttactgctcatctgggtcatctccttcatcctcgctctccccaacttcatctggcgaactctcaaacaccacgtcatcaatcttccaaacctctactccatcaacttctgcttcgaggagtggccgacggaacacggtcgaggctactacagcgtcttcgtcattcttgtgcagtattgcctgcctatcgtcaccgtcagcgttgcctatgctatgatctgccggaagctcaagttcaggatggcaaacagtaccgtcagatcgtccaagaaaggtgagagggacgacaggaggatgaagaagaccaacaagctgctcataaccatcgccctcatcttctgcctgtcatggctgcccctcaacctctacaacctcgtcgtcgacttccacaaccccttcggcgacgacatggagacgatgctcgtcgtgtacgccgtctgccacatgatgggtatgtcgtcggcgtgttccaacccactcatgtacggttggctcaacgacaacttccgtaaggagtttctggagatcttcagtagagtgttgccgtgtagggagccccagccccagctgcagggcagcaggagaggtcaggccttggtgcaggagaaaaccactcccacgccctccaagttgcccgccaccccacagggcaaggacgagcgacctaaggtgctttacatgaaggctaaagaggcggtagaagtgaacggcacctgtgacacccagggtgacgaaacctacatcacccaggtggtcactaacgccacgctgtaggaagattggtagttgtggccgtgacctgccgtgacctgttg**

**P2.** Neuropeptide F receptor II (DS01-Homarus1_Transcript_17633; translation frame 3)

**gaaggcgaagcgtgaggacagacggtccgagtcagccagaccagtcactcgggggagagccaggaactagagaaacttattacgtgtcttactcttcgttcttcataaatgtgtcggtgtttacaatgtggagtttgtacttataacaaacttttaatcgatgtattttcaatgaatcttcttgatgtcaacagagtgtggtctgtaaaacctaacatgctttgggtttgtatatcttaatggttaatattagtataaacaaactgtaaccttcctaaacattaagctgattctgtttactggatatataaaggtgcattaacataaacagcttcacttacctccaagaatatcctggaagacgtaaatgacgaaccaaacagtccatctggctcttcatgttaggtggctgtactgcggaggctaacctgggaataaaacaaaaatccaaagttactgtggcgtttacactacattaatcatcctggaaaaaaaaaagtagcgtctcttgtacaaattaattagacccccaaatatcttagacttggatgagctaaatgtttgggttttatgtggatgataataaactcgtaaacttgtaatcatcctggaaaaaaaaaacttgtgtattcttgttcgaggtaaagaaatgaccccaaaagtaagcttatcttggctggtctgaaccttctgcctttccctgtgtgtgaagaacatctgtatgacggtaatacagtacacagtgtgttagtgaaggaaaacatagtgtttgtgaccataagaacgcctgtctcctttacccgagagcaaacattcacgtgacaatataaaaagaatgttaaatgtgaaaataaaaacgactgactatttttcctccgtgtaaacattcatatggtagtgtgtgtacgtgaacagtagctgatatatactaccttatatgtgtacacagatcccattagtgcacataaaataacatgtacacgatgctacagtcgtttacacgggtgattataacattatgaacagttgtctatgaacgttctttacgtcaattatcaagtttgtacacatcgaaggctgtacatacataaatacacacacacgcacgaccgataacgtacaccgataaagaccagttaacgcacatgagtgatcatcataacaatacatgtacatgatttaggatgtgcacagagaatcctcagttggcgtacacacggaggtacacatagatatacgtgacagagacacagggtaagatgttcacattggagtggcagacggcaggggtggggtggaagaggctggacggccccccaccctccctcaaccccagcatcatggcgctcgactccctcgacttcgactctgaagactttaacctgatgaacctcgctgccctgaacgagaacctcaccagcctggcccacaacctcacccacctctttaacctcaccggcggcatcaacgtcgatctcctcaaaaagttcgagaagaatcgccgggtcggggacggggcgttttacaccctcatcttcgcctacagcgtcctcatcctcctcggggctaccgggaactccctggtggtgatggcggtcatcaggaaacccgccatgagaaccgcccgtaatgtctttatcatcaacctggccatctcagacctcctgctgtgcctggtcacgatgcctctgacactaatggaactcctgtcgcagtactggccgctgggggacacgcccttcacctgcaggctggtgggcacactgcaggccacctccatcttcgtctccaccatctccatcaccgccatcgccctcgacagatatcacgtgattgtgtatccaacgaagaagtcgctgcagaaggttggggcggtgggcagcctgctggtagtgtggctgttgtctttcctgctggcgctacccaacttcatctggcggacgctggagacccaccacgtcaaccttccggggatcgaagtggtgagattctgcttcgaggattggcccttcgaacacgggcgcgcttactacagcgtcttcgtcatcctggtacagtactgtctgcctatactgacggtgagcatcgcctacgcaaggatctgtaacaaactcaagtacaggatgaccaacgcctccagcaggtcagcgcgctccagaaaggaagacctgaggatgaagaagaccaacacactactcgtctccatctccctcatcttctgcttgtcatggctgccgctcaacctctacaacgtcatcgtcgatctccataaccccttcggcgaggacacagagtctatgctgatcgtgtactcagtgtgtcacatggcggggatgtcctcggcgtgctccaacccgctcctctacgggtggctcaatgacaacttcagaaaggagttcctggagatcttcggggtggtgtgtccctgctgccccgtcgtcaccaacgcctccaggatgaacagtctcaagaccagcaggatcggcaaggagggcgggtctctgaagtccctgccgctctgtaatcacccggtggtcttgtacactaaggcgcccgaggaacagacctgtaatggcctcagtgtggacctgcaggaccaggaggtcaccttcatctcacaggtcgtgaccaccaccaccctctagctcaacacagacgatcgacggaaggaggaccaggaggcggcgacggaagagtgtggccatgtcctgaggaagacccacaccttacatcctaccacgaggagtctccaagatcctaaacgggaaagaaagaaaaatccttcaacacaggatttcatttttggaagaagaaatttcatatctactatttctcttcagaaaactcatgtctaggatgtcaatagtttcctttaatctagtgtaggatgtgctttgaatttctcagctgtaggatatcacctaaaaatccttcctaatgatgactataatctgactaaagaaataatacgcctgactcacaagcctgactcacaaatgtatatattaaataacaactttannnnnnnnnnnnnnnnnnnnnnnnnnnnnnnnnnnnnnnnnnnnnnnnnnnnnnnnnnnnnnngaaagcaaattatacaaagtttactcgtgaattagagacaaaaattataatctaaaaactgtgccaaggaagagacgtaataaaataggaaaaatatttgaaagaatcatcccacgtataaataaacaaacaaacaaacacaaacatagaacacgggaagtctatatattgccctaaggaaataaaacaccgtacatgaataaaatagaaaacggactggacagaggagagaaagagggtcaatatacagttatgctaatctaaaaatccacccaaaataacaacaaagtcataaaatccactacaagaagagattacagctataaatgtctaattgtcaagataaaacaccaaagtggggctacttaactaaatacctttgacagaaaacgtaaatattgtagaatcatagaaaacgtacctaacctatcatcaaagaaaacgtatatatcaccagtaactacactaggaagtaacacatgatgaatacagaacataaatcccctaatgatatagataattaccctatatatatcgtcaaggtgaataattagcccaagtttcctcaatatatcaagaatgtgacccaagattaagttccgttgctgttaaaaaatggagtcgtgatttatggctcttttgtaagtcttggcaagtgtagtcgggtggagtagtcgggggaggtggaaaagaggtcgggtggagatagtcgggtgaagaattcgggtggagaaagaataactgggatggagaaataagtcggatggagaaggagacagaaagagaaataactagacagagaaacggacagataagacatgatgaacgaaaacaaaagtagacggacaaacagacttaaggtgaaaggaacgtagagacagacagagatgacaaattaggccaaagaatggagagatagtgaaatataattgataaatacagggaaataaataacgattagataataaataaagatgaaaaaaggggaaaaataaataattaagcaaacagacgacagaaattaggacaataatacccccggttttttttttgtaaatataaaatagtctcccactacctacacccgctgtgcgttttctataaattaacgttttctaaattaatatcttttgaagacttatggtatactacactaagtggtttcgaaccttcacttaatgagcccaagtagaggttcgaatccctcatgatgacaacaacttgagctcggattctgaaactgaccaattatcttagggcattttccctatgctggccaatcactaccctttcctggaaaaaagcagaccaatcatcttcatcccaaggaacaagcttgaccaatcagtgctgttccttgaataaataagccaatcccaatcactcctagattcaaaatgggcaattaccacatttcctataaataaaactgaccaattaccatgcttcctggaacaagatgaccaatcataatgtctcctcaaacaagttggccaattatcctccaccattgaacaaattgaccaatcacaattaatctcgttttttcagaattcaagctcaattatattttgttccaataatatatattttctaaaagtctccagaaatatatctacatgtttgtgtgtgttatgc**

**P3.** Neuropeptide F receptor III (DS01-Homarus1_Transcript_9534; translation frame 3)

**ccactcccagctcgtcactgaaagctgcggtacagagactgatctacggactttccctgagtgacagctaaaaattggacagataattccttgagattatggcagcagcgaggcgtgtgcgactatcacggtatctcgcccgggattaaaaaagatttcactcgtcctgatgatggcggggaaaaaatgcagattgtcggtgatgaattaaatgtgaacttttctgccgcccgacatgcgatagatcgtacctggtaaaaagagaaccgaggtcattagttatttttctttatataatttgaattgaaggttggaggattgattttttgtgagtgaaatgtgacgcagcagtggaagacgagtttaacggattacacttttattttgtctccttaaagtgatattctcaattttggaaagatgacagacacaggaccataaagcggccgctctcctgcaggtgttgtggtcttcagtcactcttctggttgtctggggtaaaacagtttgtcctcaatgaagaattaggttttttnnnnnnnnnnnnnnnnnnnnnnnnnnnnnnnnnnnnnnnnnnnnnnnnnnnnnnnnnnntaatgatggtgttgtcagtcattccatcctcggtcgctgtctggtatgacatgatcacctgagtgcctctgtgttgtccgtgccgcgcccaccccgctcacctatccatgttggaggagtcaagtcacggtggtgagggaggagtggtgggggagaaggatgatggtgagaatgtgtttgaggaggtgagcggtgtagaggtgtcctgcagggaccatggtgcctgcgggggcttctcccctcaggaggatctcaccaatcctttcctgcccctggagaagctgggcaaactcctgctggagccaagcctccccaacaacaccaacacgtcgtcgcatcacccgctcttcaacttctccatccacgaggcgtacgacatcatttccgacacccaggccgggtacctggacggggtgacggaggttgtcttcatcgtctgctacgtcagtctcatcctcttcggcgtcgggggcaacatgatggttggttgggtcatctggcgcaagaggactatgcggacgcccaggaacctgtacataataaacctcacagtgtcggacctcagcatgtgcttggtgtgtatgcccgtcacactcgtcggtctgctctacaagaactgggggatggggagcctcgcctgtaagctggtgccggtgctgcagggagccaacatcatggtgtcaacctccacggtggtagccatagcggtggatcgttatgccaccattgttaaagcgggagggtcgacgaggaataagttccacgtagcggcctccatatgtgccatctgggtgtcgtctgtgctgtttgcgctgccgctctacttctactacatcgtggcgcaggtgaagctgcagcacatcctgctgtactcccgctgtgtcgaccactggccctccaggagcgcgaagaacgtgtggatcatcgcgctcctgctcacacagtatggcatccccatcgtggtgctgagtgtagttcacgcccgcatcaagcgttacctcagccagcacatgatgggccagtatgacgccaggcgagcacagaaggagatagagcgtaaccgtaagacaacaatcttgctctccactatagccgtggctttcgctgtctgttggctaccttggaacatcgtcaacctgttggcagatttcgaatacgaaggtttcaaagacccgactcacttgtacacggtgtttggcgcctgtcacatgatcgccatgagctcggcgtgcatcaaccccgtcctctacggttggctcaacaccaacctgcgccgagagctgctcgagttcctgccacccatcttcgctaagatgggctggatcctgccggagtccttcaggaggagaaccggggactcgccaacgagacagccggagagcgtgacgctgctggtattccagggcaaccagaacaacagcatccagacggtggctcacccgcctcagaccatcaccaccaccatcatcaaggacgacacctagccaggtgtgagctggaggaccaatcaggaggaggtgaccatcatccgccacgacgacggaggccgccacgcagcgttggacggcacccactgcagaagagactcagccaacaactgccggggaggagcggcggtggccaccacgtccgggaataggagggaggtcacgtttgaggctctggtggagatgaagtcagaagtttcgtcagaggtcatgtcggatttgtcgacggaaataacgccagaaggtcaagaggaggtcatggcggaaaagacaaccagaggcattgtagaggtggcaaatgagtcggcattagaagcccccagaaggcaggtcatgcatgacggactatcatacgcaccaaaagaggtcaagtctacaccctcgtcagaggttacgtgacgtaaccaacgatggttaacagacaaagaattttttcgtttatgacagacagcgtcggggtctttggactcgtgaattgtgtcaccagaggggacaagtgccacgcctgggacaagaaaccggcaggacctgatctgccttgagttttgtgcctctggtcttcttcctgtatgacacgggttgtggctaatacttctcacctcatagtagtgcgtaatttgtcattaccgagcggagatgttctcattcagctcaacgacaaaaggaatgacgttcaccttcatccctgcagtgaaaacaaatgcctgttgcggcttttacgtgtggcggcactgaacagggtgaacagagagtgcaggggtcgacctcgtgaatccatgaaatatcacctcctaatttcctccagctaagacataaagaagactgtggatttgtgccacaaaagattaaaaatgttacatgtatatgaacgttttttgaaccttgatgtaaaaactttcaatcgtgctgagattctcccatcactttgtgctgctattaattataaagatttttttttttagcgacaagagtgtcaccgaccaagggtagagacggagcttgtgggtcgtccaacacaccctcatgctcccagataccgacgtaaaaaacgaaagtggtggcctgaatcatgccttttattcctgtaccatcgacatgtacatgtattattaagcgcactcatttccttggtgctatgttcattcttgtatatataaaatgtttgtatacggtttgttttgggagaatattctttgaaactgttgtgtgatagcgggtcagctatatatataggatccagagagagagagagagaga**

**P4.** Neuropeptide F receptor III (DS01-Homarus1_Transcript_16169; translation frame -2)

**tctccttcatcgtaattttattcaaaatataatcattaaaaatctaaatttaataagaaaaaaagtacaatcagggtgcattcttatatctacagttacagggaatatccattaccattctgaaaactgaaaaattccaaaaaccgaaatgcatctggtcccaaggatttcggataagggattgtcaacctgtacttatgtatcaatagaccaggtagctagttgaaattagtaagatttcattattttgtacatgtacttcctttcaggtgtttaacacttgagctgtgaccttatttcttcttataaaatcttcagaatatcaagattttttctttttattcatgtgagttaaaacaacagtgattatagacctagaaatccaactaaaactaaggtatatagttggatgtttaatagaaacacttggaagtactgtactacccctagaactattatatcaactgattttctcgtgtaagaacctcaaaggtatttttctctatcactgaaaacaaagtccatagcaagacatgacaaatctcaagaagataattcttaatatttccgatctagcaaattcatatgcttatgacaatccttgtacgatacttctgtcaagtgaagctatgagcagttacacaacaaatattacaaaacattcacttgatgcagcatcatacatcaactacgcatatgacatacagtatctatttttactcttatagtaaatccatgctaacctgttgctaagttatttcaagctttgttattgcttacagtaagttcattattatctataacaggactgagctttcaatcttgatattaagatacagttcactgtagccttaacagaattgatttcaaacttggttcttagttacagttagctcatcaaaacctgtagaagagtcgtactctcggctgagtttaatatcagttatttccccaagtgcgacctccatggtggtgaagacaacaaggtcacccatgacgtggccacctagcgtgccaccttcttcatcactcaggcagatgtgcaggtgtgtcccacccttgctgattgtcccggacattcccaatacctcaaaatgtttcttgaatgtttcgatcttgttggagccatcagttttggaagcgagacggagggtggcactggtgatggagccacagcatgtcattaccaccactccactaggtgcctcacggtgcacatactcctccaaactttttctcacttcctggccaggttcgagtcgaagaacatgcactttcatattgctcattttcttctgcttgtatcctcatacctgctaggtctcgtcgtggttttgatcggtaacttccccaatcttcgccgactccgtcttaaggaagggcgtcttcgtctgatggtggttctttttggaccctccttcttcaggaacatccttcccggggcaggagtcgtccctgaggggcctatggaccagcagggacacactctccaccgtgggatttgttcctgtcaccccgccgccccctgtgttgcctgaccgtcgcaatggacacgacgtcttcctgagtaatataggtatggcctgcgtcatctccttgcgcaggttggtgttgagccagccgtagaggatagggttaatgcacgccgagctcatggcgacagcgtggcacgacccgaacaccacgtagaagtactccggctcctggaagcccgcgtagttgaagtctgcgaggaggttgacgacgtgccaaggcagccagcagatggcgaacgttacggcgatggaggtgaggaggatagtggtccgcctgttgcgctcgatctcccgctgggctctgcgggcgtcgcgttgagacatcttgtgactgctcaggtagttctggatcctcccgtgtacgaagctcagcaccaggatggggatgatgtactgcatgacgatgagcgtgatgttccatgtgtacttcaccaccggcgacgaccacgactcaacacacttctcgtacagcaagtagtccttgatctgcaccttctctacgaagtagtacgcgaaaagcgggaggggaaacactagggacacgagccatatagccgccactgaccaaggtatgtacgcccgggcgtctctgtttctctggacgcagacgatagttaaatagcgatcggcggcaatggccacaatggtcgccgtcgacactagtatgttggtacactgcaccaccggcactaacttacagataaagttccctaagctccagttcttgtgcagaagtccgactaatgtgaatggcatacacactaggcacatactgacgtcagacaccgccaagttgataatgtacacattacgagctgtcctcagctcagggcgatggcctatgacgaaggcgacgatgaggtttcccagaatcccgacagtaatcagtaaggaatagattgtgatgagggaaaccttagcagctgtgcccaggtagctcttctggttggtttccttaaggatcttcatgatctcctcccaaggaagactctggttggctccagtaggtagaacagagctcttaaagaatgactccagctggttggtgaaggggttgtcctcctccccgatggttgtaccggacatgacgtgaccctcaagagccttgtactcgtcaggctggagaggacacatgacgcccgagcacagttcaggctctgagaagagggagatattttccatggttatttttaaagtgtcgacccgggatagctataatgccgatgattaggaaagtctgatgtcacacatgttaaccatttccaccaggttcatcaatgacaactaatgaaacaagaagacatacatgcggctttggaaatatggaagaaatgtcaagaagttggccgccctatctacaagtgagccccaagaaactatgccctgaggacaggaacctctctaacatcacacaacaacacatgaaacgaagttcctgtcctaaggtgaagggcatcagagggacgtgggcaagtctaccctgctagcggcagtacgtgaccagagcaccatcgttatcaagcaccgagttcatacactggacttgacctcctcagggatcaacaacaccatggctactttgtccagagttcttcagctacgagaaccagtacacacttgtttctttgcctcggctgccctgaaggctcgtgcacctgagttaacttgtctagggtccctttacacaggactaaacccccacaaggctcggccacctgaatgaccttgtgaaatggtttagtctttctcaacctgcatcctccgcagccatccataactagcgtgtgagtttatgtctcatctttgattgtcctctccccgttccgcttctgtcctcaggtgtgtgactagcgtggtcggggaaaaggtgagggagactcaggggcttcgagtagagggacttttcctccgtcagcgcttgcaggtgacgcgcggcccacgacactcagcaacctcaaatgactttgcagatagatccacaatagtcagggcagtgcttgagtgggatggtctagttagatcagagcagcgcttgagtgggattgtcttgtaagatcaggttagtgcttgagtgggttgcgtgggacagtgcttgaaggatagactttacttagtcagatcattgcttgagtggggtagtctagtaaggccaggtcagtgcttgagtgggtaagtgttccttggccgtatctggtgttgtgtgaggcaaggaggtggtcttgccggccactgactcacacacgtcctctctctaccggggctagccgggaactcgcgcccggaccctccctcc**

**Q1.** Pigment dispersing hormone receptor I (DS01-Homarus1_Transcript_14445; translation frame 2)

**tcaagaaagtgtctgtagcggaacactgtttgatgaaggctcaacaactcctgttggggacgatgaacttcccagcgttgagacagagccagagccagtgttcagggtgttgagaaaaggcccgattgaggaaccctgacagaaaagggttacttcctagtgtacgaagagtccaaatgtggatcacagacagaagggggctatataacactactagacaggggctactgcgtataacactaccagaaagggacttatgcctagtgttatacccaagtgttgtccagaggtgacccgctccacgcgccccgctccacgcacgctgaactaaccagtttggcgtgctggtggtggtgtgattgttgtcgttgttgctgttgagcggctgttattaccatcatgctctctgccctactgcacctgctgccgctgctgttcctgccccccgctgccccagcattcgtctgtctagacgccggtgtgtgagcctgaggatcaccccctctcacagtcgccgcccatgacttactccatctacctgcaggcctgtgtcgacaactacgaacacgtcaacctgtccactcatgagagttggtgtaacgctacctgggacctggtgttgtgctggccgcccacgcccgcaggagggagcactcggctgccctgcccacccgtcaagggggtcgatccttcaaagtatgtgtacaagcactgtgatgggtcgggtcggtggacaggcaagaccccgggggacttcacactgccccagggatggaccaactacactgtgtgcttcaccaaggccatccaggagatcatgcaagaactgtacaagcagtctgatgaagacgcccagacgaagctgaacatcgccctgggtactcgcatcatggagatagtgggtctcagtctctccttggcgtccctctgtgcctcgctcgccatcttcttccacttcaggagtttgaagaacaaccggacgaggatccaccgcaacctgttcgtggcgatggtgatccaggtgatgatccgtctggtgctgtatatcgaccaggccatcatccgcggccacatcgtcggtcactcgcctacgaacaccaacgcccccaggcagggaatcgacaacacgcccatgttatgcgaggcatcttatgttctcctggagtacgcacgcaccgccatgttcatgtggatgttcatcgaggggctgtacttacacaacaagataacagttacagtgttccaccacaagttctactacagtgtgtatcacgccgtggggtggggagtgccagtactcctgaccgccgcctgggccaccgccaccgccatgcactacggcaactcaaggtgctggtggggatataacttcacctcgtatttttggatcttggaggggcctcgcttctcagttatttcgatgaacatggtgttccttctgaacatcatcagagtgttggtcaccaagctgaggcagagtaattccagcgaggctctgcaggtcaggaaggcggtgaaggcggccattgtgttactgccgctgcttggcatcactaacgtgctcaacatggtggcggcgcccctgggacgctcggcggcagagttcgggctgtggtcgtacgccactcacttcctcacctcattccagggcttcttcatcgccctcctctactgcttcctcaacggcgaggtacggacggcggttaggaagtacgtggacaactacctactccaccgcagcgccggggtgcgtcgagggtctgggctgtcgtccgtcttcctcaccacagtgactgacctgccaagggaccggcacggccggaccagacacctctgtgcctgcctgaggggcaatcacagtcctacacatggatcgtacttatgacagttgcgacgactccctggctgccccaagaaggtcgtcagggggtcggcggcgcaccatcaccacctacgcaagaaaaggtctcacgaccgctctaccttcagcacggggcgacgtgacggcgtgggcacggctcacaccactccagccactctgcacgccactcgcgacgctccatccttcgcggactccaccaccacgccctccaacacctgtcacgtctttcccgacattgagctagactccaccgacgcctccaatgccatgccgtcgctacatatggtagcgtcccactgtctcaggcaatgtagtcgtttcagcctgccggaggcgcagttagagtgtaccaaggctcgattaaaggacccggaagcagagtgcgatcaggactgctgctgcctccatgagtcctccagtaccgccctggagcagctgagggacagccccagtagaggggcaggtgaggagcaggaagggggcgacatagcgctgaggacacttaagcctcacaggtcctattctttaaggctcactaggaactacaactccaacagctctcgagtgagggaacaagacagcagttttactaatgggccttcaaaaccccaccttaattgtactgactgccagtgtaagagtgctcagcgagtctcggatgtgttggtcgacgcgagacataaaaatcgctcgtcccaagttagttccgtggcggaaatccctgaagtgtgctcgagcctcgtagccccatgtgcctcctgtcaatcccagacgtcacgggactcctgctacgaccaccaacgagacgacgtcgaggacgtcgaggacgtcgcggaggaggtgactgagtcagggtgctctgacgcgcgctcagaattcgaagaagttatcatcactactacctgctagcactgttactgtcgagttaacagaaggaaacaacagatctggtacctcatcctgctagctgtgtagtcctaatattaacagctggagatgagagcagcgctctttaagagccattatcttaacagtacagaaagggtagtagtgaagaggatggccagcctactctcataacaagagcaggagttcattgccttctcatccctctcttccacaattcttgcagtggctcgcatcctcagtacttcaagcatcctcagagacaagacagtgagggctgccctctatgctctgacccgggaaccccacgtctcagaccacatgggccttcacctcacctctaaaattaagctcgtccgacaatttaagtgtttttaaacagtctttagtgacgactcttgacaacagatgtggccagttcgtgctgttcaggactcgggaaagagtcataagatatgtaaaaagcaaaaaaaaaaacattaaggtttatgtcaaaaatttcttaggctagtgtgtgatatttctacacgttcttagtgtcagtgagctgtaagtaatccgccttttgagattgaatccccttgttggatttagaccaatatatctatttcttttttgagatgtcgcttattaaaatatacatgattgtaaatgtcgctatcgtaaaaactttacttctaaactaaattacaaaagacatgagaaggccaagaggatgtccagcagttacataaaatattggtttaaaaagaaaatttaaatattccacaatttttcccattctcagaggaattcaattcataatattctagtttcttcttttcaataataaccaaatttggcgataaatttaagcctatctcgattatagtaaaaaataaataaaaatatatggttttatcgagaagcaaaattttcctgtaaatttgttattttctttggtggtaatagataacatgggtctagatttatagactacctgaagacgataagaaactacctgtgtgggtgaggtgacctactcaccttactccaacactgtccagtaagtcactatgtgtatgtcaaaaaccattacattaacaataataataaaaagcgttatcagtcttcacaatagatgaggtacaggtaattacactaatggacaccaaagcattcgcttatatcgccacaacaaccatcaccaccgcaaccattacccccaacaaccatcaccactacaaccactactactgcaaccatcaccacaacaaccatcaccacaacaaccaccaccacaacaaccactaccacaacaaccatcaccacaacaaccatcaccacaacaaccatcaccacaacaaccatcaccactacaaccatcatcacaacaaccatcaccacaacaac**

**Q2.** Pigment dispersing hormone receptor II (DS01-Homarus1_Transcript_11293; translation frame -3)

**tcttccctctctcttccctctctcctccctcatctcgctctaacaccaggggacgctactataaacatcttcactatcttcactaccttccttccttatcttcactatcttcactatcttcactatctatcttcactatcttccttatcttcactatctaccttccttagcttcactatcttcactatctatcttcactatcttccttaccttcactatctaccttccttagcttcactatctcaccttatcaattacatacaaaatggaccaattatcgagttgattaccataaaggaaattctcaacaggtgtgaagggtacaggtaagtctcctttaggtggacaggttaattacgttggaaaggtactgaggtaacgaggggattaaacctggtgtagaaatgagttggttcagtattttaagtatgataattagactccagacaggtaaaatctcattaactacctgtttcattaataagagtactgataaagtaactcttaatcacctacatatatagataattacattcggcacctgctcaatattaatcacaaattaattaacctttatcaataattacttcatttttctaattacgtaacacaggtaatttattttctggttactctacataatccttcacactgaaataattaaggattatacagtaaaatttttcactcatatggaataatttaagggataaattatagtaattatatacacctcaaaagctgtcgtaatatagtggtcaatatataaacccatatatccaaggtttaatctaattataccatctgacgttaaacctaatcacgtatagaaataaacttcatatataatcaatactcataagcctctgccatcaacatcatacagttttaacccttaatgatagtctaattaaaacaacatcgtcacctatgcagttttaaccctaatggtagcctaattaaaacagtagttaagtatttattaacaataccgtgacttgtccatattactctattatcagcttttgcgtccagtcattgtaaacacgtctagcttataatcccttcatcagtgaggtaaacagacctacatgtaaacaatagaatgtcaacaaacaccctaagcttccatatatatataggtttactaacaaatataacagagttttatagtatatatatataaatttatatatctataccaaccatccaccaatgactatatacagtgtacacagaaccaatggaaaaaataaccatactttttttttcgattatgtataaaagttttggtatttttttcaataaatctgttcattcatcagttatttcctttacatattttacactatagaccaaatctcacagcataaacgccataccaatatatacaatccactctaggttatgtatgtatgtatgtatgtatgtatgtatgtaaattacattcatgaattgacctgaattgacctgaattgacctctgatcctcaacacacggccaacacccccagagggccacgtagggcggccagaggtcaatgataacacgataacaataacgagataaaaaagaatcagtggaaataataacaaggtttgacaggtgtgacaggcgtgacaggcgtgacaggtgtaacaggtgtgtgcgtctctaatcacctgcacacgaatcataggtggctaattactctcacactatacaagacatttaaaacacccatgacggcagataattattcaaaatgtattaattatgtgattatatttagcataatgagttatggcgtaaatctacagtttaagaataacttcctaaatggcttgtttgctagtgggaatagattgaaatagcaaatagcagctaatatagtgataagcgtttatatcatcctgtcaaatgtcctgctagtactaaaagacagacagacagacacacacacacacacacacacacacacacacacacacacacacacaaggctaaagcacatcctaaaaactaaagaaaaagatttagatacaattattatcatcttgaggaacattaagacacacgtactctcatttcttaccaaacactaggacatatttctcataatccacgtataatttcttaattatctatttaccttaatatctacgtctagtaaactcattcttagttaattagttatcacctcattatactattaattagctatcagtggtatatgtgtctcaggtcttatacggaacccggattgtcttagaaatctgttgataatgagagatatgtttccctctcagccaattagaataaagtatattctcattcgtatcttcttattggtcgagtgcgtcatggatgtgtgtttacctttagttttcacacgaaccaatcagattattcgatgatgtaggtggcggtaatgccaatcagagcacgtgaagagactgatgagatgtagggaggaggaaaaaagatgataatgaagttgaaatgacgaaggaagagagggagaagagtgaggagaagagaatggcaaatagagagaagatgggcaagagaagagaacgagggaggggaataagagaggagagatgagagtgaggagaatgaggagggagataagatgggtcatacagtggtagttataacatctggctcttcactgtgttctatcgttatttcttctctgaatgtagttaatggctcctctgctcctatcgcctcacttgcaatattgccattaatgcagcttcttttagacctcttgggcaggagtcgttgagtgtagaggttagccacacacttagtgatcgttcctctcacctcattgttaaggaagcagtataagagagcgacgaagaatccctggaaggcggtgaggaaggtggtgacgaagctccaggcggcaaactcgactatgttcccctctagtggtgagtgaaccatctgaagactgttggtgatgcctaggaggggcagtaagacgatggctgcccgtactgccttcttggcctgttgggtctcagaggacacactggcctgtagcttggtgatgagaactcttagtatgttgagcaggaacaggaagttggtaataatgacggtaagacgaggtccttccagtatccagaaataaggactaaggttgtatccccaccagcactcagtgcctgtgtgttggacggcggtgacagctgcccatactgtcgtcatcaccactggcacgccccacccgaacaagttgtatatcgtgtggttgggctggtcggtaaacactgccacagtcagcatactgttgaggtaatgtccttctataaacatccacaggaacatggccgtcctagcgtactccatcaacacgtagaacacctcacacaacacaggcgtgttatctatgcctgttgacctctgctctgtcttccgggtcacgtactggtcaatgtatagtgtcagcctcaccaccagttgaatcatcatggccacgaacaagttgaagtgcatcctggtcctgttgtttcggagtgacctgaagtaagagaatattgcgagagagacgaggatggacgccagggagagagagagacccactgtctcgatcactcggctcacctcagccacttgtagcttcaactgggcatcctccttagacttggcatagagtttatccataagtatcctgatctcgggtataagacaggtggtgtagttggtccagcctcccgtgtacacctcacctggcgtccggccctcccaataccccccggcgacgcagtgacgctcagcaacttgggaggggtcgacacctttgagaggaggacaagggagacgagagatggtatcagctggtgtaggcggccaacacaggatcttgtcccaggtggcgttacaccagccatcttgtgcctggtcctggtagtataacacacactggtcgtaactggtgaactcccaggctcgcataccggacccatccatcgggcggcgaggtgggtggcgggggcactaggagggtggcaggaatcggttaataatgggtggcggtagacgggaggaataagttagta**

**R1.** Proctolin receptor I (DS01-Homarus1_Transcript_26916; translation frame -1)

**ttggtgacggataaatgtggttgatagtaaaagtagttgaatagttatatgttatttcaaggtaaaaagagataaatgttataaggaaattattattagatatgagatgaaagtttgcatcaaggtgacgctatgtttgagtcatggttgatgtctctctaatgtctcaaacttttcctttattaaaacattaacacgatgtaacaggtcaggattatgtgacggcaacatatcatcaagctgtgattgtcatgtcgcagagaagtgatatattcatactaaaatatgtggataattcacaatatcttctgttaatcattaggcttttgccattagagtacagtatatatatatagttatccttttctatacaatttatctgtttaaccatcaatctggtgaatgtatcgttgattcgtttgtcgtatctgaagacttgggtggtgtcagtggggaggtgttgaccaggtgggagtcatcacccttctggagagtgatggaggggagatgaccgtcagcgtccccgttctcgtcttcccccgctctggtggtcgatgttggtgatgactgactcgtctcacaactctcatcatctatgaacttaaactcaagtagattattctcctctacgctgctcacagacgaggatggcgtcggtgccttctctataattatcactccctcatcttccttgtacttcttcctcacctcaaggtcgaccttctcatcagacacctcacagtggttgccgacatccaccttcttcagctcaggactcggcccggggttaatattgttatcaaaagcttgtgtggcgcgtttgttgcggctgatgggagagacgtgggagtgtttgttgacgcgaacgtgggcgttgttgacgtgtgggtgagcatctgctgggctgagggtcagatgtcgaggactacgcccaaacccgtactggagtgttacccttgcgtctagtgagcccaccccgttttctttaaatgatccagacgccagacggggggagtggtctcttggaggtctgggtgacggggtggatgacttggaaaccctccgacacactgacttgatggaggtagtgtctgaacctcgtcctccaacacacccacctcctccactacctcctacagtgacggaggtgacgaaggagtgggcgagaggttgctgatggtacatacagggtacaaacgtcatgaggaaggtccggcggtaccggtcagagaaagcgcagtacaggatgaagtttgctgcggcgttgactgcgttgagaaggttgaagatattcccaaggcctctcatgattgcgtaggttttcgtcttgggcggcggctcatacacggtgctgtacagcaggagtacggccgtgggcatctggcagacaagagcgaggatgactacagctataagcatgacggtaatcttgtattcttgttggtggctgtagtgatctctctctgataccagcgtcatcttgcggcggtgaagcttactcgttcgcacggcctgaatgaggaagaagttgaagacagccaacagacagagcggcaacaggatgaaagtgacggcagtgaaccagtagtaggtgtgacggtaggtcgagtcctggccaaggctgctgtagtcgagggcgaggtaaggttggtgggtgtcgggtcgagtcttagtgacgaccacccactcgtggggggtggtggcggtgagggcgaagcacaggaagtacaccacagtcaccaccttcttggcccgcgacactgtacacagcacctttcctttgattggatgagagacggctatataacgctcgatggtaaaggtcacggtgatccagatagaggtgctgctggaagcatcagtgagccagagaccgtagcggaagtactgccagtagaaccagtgcctcggctgcttgatatccgggtggtgttggagagacagagagaacacgaacaccagatacaggaggtcggagatagccagcgccgtcaggtagttgttagtggaggacctcatcttacgccttgtgagcactaccatggagacggcattccccaccaggcccacacacatcaccaacggcaccaacaccctctgtacccagtgtcgagactcgtccatgaacctctcgtaggaggcgtagttcgaggaggtggtggaaggtatggaggacttgtcaggaggacctacatgatgctcgacgctgatgtaggaggtgttctggtcctctgggtagtatgggtagttgtcaccgtctaggttgaagagcgaggtgttgtggccgtaaggtagaagaacagatgtcgaattggggtcttctaacaatatggagttgttgatgacgcctgaaggagcccactcaaacatctccagcacctccatgtttcagtctttcagttcagtcttttcaagtttccaagactaaagtacgacacagatcccgagtgttgtcctgtctcacgatacttagctgtcacggttaggttaagtattccctgtccaaagagttgataataacctttgttttgttttcaactcttgtcaatcactggagaaactcgtccactcagagacagacagacacacacacacacatacttactctcactctcttctatataccgcctacgtcaccgattcaacaagtgaccacaaaaacttgagtgttcattttaagtttttataagtttggttaaggagaacagactcctactggcctctaaacactaattgtcgagagaaaaacacacaaacaccccacacacaaacacccaagaactgtgcgtgtgtgaaggagatgtcagcttcactttgggtatgtgtgtgtgtgtgaaggaaggaggatagtctctaccagcgaggagaggtccgaggagagagagaggtctcactgtggacgatgagggaggagggaagagccaagggtcgccaaccactcagtacgagctctccgccccg**

**R1.** Proctolin receptor II (DS01-Homarus1_Transcript_45976; translation frame -1)

**actgactcatccacctcctagtgacactgactcattcactgcttcagtccacttacaggtacagctgcatcaaggcttaccttaaagtttacgtgttaacactcacgtaatactgctcactaatacatggttacaggtacaccataacctacagtgaaggaacctctcacacacatatatatatatgtttatatttacaggtgcatataagacaaaaatatggatattggcaagtatatacctggcaagcgatctatggctgacttataaatcattttgacaatatgccgaaaatgtgtcaaaaatataagcatattatcttcccacgtgagggtgtgaagtactgtcctccgctgctgtacacacactagttagttacttcgccttcatatctttctcagcgttgataaaatccccatcaatcacacttatataactataacaaaacaaaacaagtatattgtctcaatcacgtagcaccttcactcactcgctcggtggctcctggatcccccagttcgatccccggcaagcagaccaagccacagtgaacacataacagcgaccacaactcttacaggagatatattgtagtgtgtggctgtacaggacacaacatggtggcgggagcgtgtgcggatgatgcagtattgctattaaagtcaagttataatctaattccgggcaccgtagtgggtaaccgagagaaggtagttagaacagtgaacgtgtacaactgttggttacaattataaacatgcttgcttaattacctgctaacacatctatttgttaatctaaagctcatgagatgtgaatcattattataatacatttatgaacaacttgtaggaaagacagacatacatgataaaccctcggtgctcccggcgggtcgaaaaataaaagaagactgggccgctcgctcgccaaatgttcgcaatacacattcagggaagctctccaccaggttcactcccacgggttaagagtattatgtacagctctttaacattacagagagtttatgatatacattattaacttatagatccgactaatactttctgaatgtaggaaaataggtctaagatttggcccaaccactgcctctagtagtcctgacaccacacgtgaccagctactagtggccacattaatgatacatattactaagtaagtaatccggtaaaatattaacatttactgtatatatacacactgatgattcctacctctttactgatggctatataaacctaagtatatattttattgtcaccttgtagagacatatatcaagaggaagagtaaacacgtaaataagccacactctaaataattatatgttcttggtttataattcgtaattatagtaaataacgtcttcatttatacagtttacagttagtgtctgtccaccaccctcacaacatgaggacctcaggtcagttagcttattcttagacattttattattagtccactgagaaggttggcggagctgtcggcgtctctgatgattcttgcaacacaacacatcacttcaccttctgtggcgtcccttcctcctaaggttctcaagagctagatattaatatttactgacgtgctgtaacaaacttatgtcaagcttgtctgactgaccacagcagacctttaacacagtctttgctaataagttatgtgtactcccataggtaacttagaggaggacatctcagagagcagtctgctcatccactgaaggttgttgacaggtgatgacgatgtgtctgccagccagttgggtgctggtattgtttctttgaggcaacaacgacgacagacgacgggcaaagcggctggcggctcctggagggggtgctgaaggggcaggagtctgggagttgggcgacccattgaggagcgttgcggcggaggcggtgacggcggggctggtcacacccgcgttcccgttgtcgctgccgttttttcccgaggatgtctgtaggatgagcgacgtgacatacgcatggaggacatcctagagaagcgcggactgccatcctccacgttgctgtaggccgtggccatccaggagtgtaggggcgaaggctgacggtaccaacgtgagcagaaggtgcgtaggaaggtccgccggtacttgtcactcatggcgcagtagagcacgaagttacaggcggcgttgatggcggccagtaggttgaagatattgcccagacttagctcgacgtattgactgttagaatgaggttctgacacataaactgttgtatacagcaggaggacagcaacgggcagctgacacacgagggccagcaacaccacagcgataagcatgatggtgatcttgttctcctgcgactgtgagtggttgtccctctccacgcggtgattggtcatggtgcgtcgctgtgctctgcttagcttcaccacgtggattaagaaggagttgaacacagccaagagcaccagaggcagtaagatgaagattactgccgtgaaccagtagaacaccttcttataggtggcgttgctgccgaggacggagtagttcatcactagcctggcctgtccagtggcgtcggtcacctcgttgatgacccactcgtgcggggtggtcgctgtcagggcgaagcagaggatgaacaccgccacgatcaccctcttggcccgactctccgtacagaacaccttgcccttgatggggtgacacacggcgatgtaccgttcaatggtgaaggtgacagtcagccagatggacgtggaactggaggcgtcggtgagccaaagagcgtagcggaagtagtgccaatagaaccaatgatgtgggcgactcatccccgggtggtggcggatggagagggagaagataaacaccaagtagaggaggtcggagatggctagggcggtgaggtagttgttggtggaggaacgcatctgtcgcctagtgagcaccacgatggtcacggcgttacctaccacacccaccaccaacaccatgggcaccaacacccgctgcaccacataccgggagatgtctaggaacgcctcgtacccgcttatatcaactatctcctgttcagaggcttcggtggcttccagatctgtgctgttgtagtagtaggagaagttaccgtatggatctgtggtgatgggcggctgtagggtgggtggggctag**

**S.** Pyrokinin receptor (DS01-Homarus1_Transcript_40714; translation frame 1)

**caaacatcaacaccaagaaatacttttaaaaaatcactccagacatcagcaccaggtatgtcctccccgagtcaaccagagaggggagtagtggtgaacctgacggaggaggtgttgggcgccgtgatgaccggcgtgaacaacgggagtggaggaactgaagtgaacgacaccttcgatgaacacgagtacatcagccgtgaactgggaccccagagagtctcgtacaacactctactgccgctgactgtcgtgtactgtgtgatcgctgtaggaggcgtggtggggaacgccctcacctgtctggtcgtggccaggaaccactccatgaggacctccaccaactactacctcgtcaacctcgccgtcgccgacctcctcaccctctgtctagcactaccaatagagatgtaccagatgtgggtgcagtacccatggccgtggggtgacgccgcctgcaagataagggccatattacctgagactctggcccacgtgtccgtcttaaatatcctggccgtgacgggagagaggtacgtggccatcacggaccccgtctacgcccgcaccacgcacaccctcgcccgtaccgcccgtgtcctccccgtcatctggatagtggcgctgctggcggctacaccctggggctactaccagcaggtcaacctcctcctgggtccgttcgggtcactgcctcagagtgcgtggtgcgccatcccttaccacgacacctccaccagctggtcttggctcatgtgggtctcctccgtgggcgccttcatcctgcccatgaccatcctcatgaccctctactgcaagataggtgtggtgctgtccatagacccgcccaccaggacgcccgcggcaggtgctggagccatgcacacccgcaaggtgggcataaggatgctcgtagccgtggtggtggcgttcttcgtgtgctgggcgcccttccacgctcagcggctcatgttcgtcatcgtcaccagctacggcaagtggaccgcccatctccgctccgtcaacaccaagctctactactttaccggtatctgctactacctcaactcggccgtcaaccccatcctctacaacctgacgtccaccaagttccgggaggcctttctcaagctgctgagtaacgaccggcgacgacgacacttgagccgccagtccacctttaatacaacggggacctccatgtcgaacgggcgtagcggctcctcacggacaatccccacggacttaggctcactcaaggacgcgacgtatcctgctagggtggctcttgcaaaatgtggccgccatgcttctttcgacgactttccagcgcctcgacctaacatggcgcgctacggtcgtcagagcagcttcgcgggaacctactgtctccccaacagcacgaacagtaacagcaacagcgtcgcctgcagccgccagaacagccgggctggagagaatggggtcctgagtcctttggaggcaagaaggtcagtggagggagagaaaaggcactcaggagagggccgtcgactgctggaaggcgagcgacagtcgtcctcggccttcaacactgacatgatcgaggaggagagaagttccggcggtgtgtccagactcgacagcgtcaggagggatcaacagaacaaaatcgcaacagaaatggagaaactgctgtctgacgaatgcttaaacaccgaggcaggcgactcgaaaaccatggctggagcgccaggcgatgccgtaaacgagatgaacttgaaacaagggacgggtgaatgtaaagtagaagtggagttccccagcaacaatgatggtgtcacctcacaggacccaggagagagggggacgacggggaagcaagatatagcatcttcatccaaagagaacggagataaagatacaatcactaatacggataatgggaaaagtgttcagttttgtgatggggtgagtacagaatttagtgtagtgtgagcgagactcccacaacttttagggcattacctctggtctccaccatcttggtgatcttcctacgggttttaagatctggatgaggacttcacaaaacatcgtacgctaaatatccttgaaagctgaagatgttagaatataactaactggctaacttaagctaaatcacaaaacgaatagataaaacatatgaaaaattacataaaactattaagtgaaactcttcctaaactagataaattaaaggtacagaatctattggaaaatattctgtaccataaaacaaaacaaaaatggtgttaagttaacgtttctagtaaaggtagaaactcactacaattttgagactttctagatgttatttttacgtatctcgttaaggaacagtcttagtgttaagtgaaggt**

**T1.** Red pigment concentrating hormone receptor I (DS01-Homarus1_Transcript_57704; translation frame 1)

**aagatgcccccggagatgttgttcaactcgtcccacgtcatcaccatcagcgcctactcctgcctcatggtcctctccgccctcggcaacatctcagtcctcaggtccatcgctgggcacaggtcgaggacgttggcttcccgagtgactcttatgatcctacacctgaccatcgctgacctcctcgtcacctttctcctcatgcccttagagatcgggtgggcgtggacggtgtcgtgggaggcgggagatatagcgtgtcgcatcctcgctttcttcaggaccttcggggtgttcctcagcgggttcctgctggtggccatcagtgtggacaggtactacgcggtactacggcctctcacagtcatggaggccaagaggagggtgaggctcatgctgtggggagcctggggagcctctggagtctgttctataccccagacgttaatcttccacgtagagagtcacccagatcacccgtggtttgagcagtgtgtcaccttccatagcttcccttccccgacctacgagctcttgtataacgtggccgggttcctagccatgtacgccatccctctcctcaccattatcttttgctacggctcaatcgtcatcgtactctacaggaaagatgtgagttcgcgggaggcgggggaggagaggtacgggccctcgctgggacgcaccaagacccgcacactgcacatgactcttatcatcgtcacagtcttcttcctctgctggaccccttataacatcatgagtctctggtacttcatagacagacagagcgcccagttagttgaccccagggtacaggcatcactcttcatcttcgccgtggccaactctacagtcaatcctctcgtctatggttacttcaatgtccggcgcagccctaagaacgccaatcccaggcaagaatggcgtatgaa**

**T2.** Red pigment concentrating hormone receptor II (DS01-Homarus1_Transcript_51875; translation frame -2)

**gtgtgtgtgtgtgtgtgtgtttgtgtgtgtgtgtgtgtgtgtgtaagacttacctgggggagggaacacacagccgctatggaccaggcgaaggttagcattatcttgccacgtcgttgagcatcattaaccttgagcgggtggacgatggcgaagtatctgtcgaggctgatgcagaccagcaccagggacgagaggtagaggccgaaggccctcaggaactggaagagcttacacgccaggtttccagccacccactgtgtcgtgatcctccagccgacctcgagtgggaagttgatgagggtgaccatgagatcagcggccgccaagtgcatgatcatgaggttgacgcgagacttccggtgacggttacgaaagagggtgatgaagacggtgaggtttccgacggcggccaccacgaacatcaccgagtagatgatgatgtcggtcatggcgttgtcgtcgaaacccagactcggtggcaggacggtggaagtggagttgatgatgggggtggtaaagttgccgagggttaaggtggcgttgatctggcagtcggaattaccgggaactatgacacactcgctgtcattgaaggcgcgttccagccagttgctcactcctccaccgattttcatctcc**

**U.** Short neuropeptide F receptor (DS01-Homarus1_Transcript_35046; translation frame 2)

**gtcagacagctttggggaagcgcgtgtcgcctgtctgtctcgcatagcgagaacgcaagccctgtggcaccagtttattacattaactattgactcatatgataagtggcactgtgggcatgattcaggcattgcaacagtgcctgtcacgcttacatgacagtcaaccctttacactccggagagtaagaacacctgttgcacgggtcagccagtgcgaccgctagtgacgacctacgtgatagctgtgcaacaaacactgtgctgtgaaaactggcatcttatataatttcgtcatttgatcatgtgacattgtagtctaaggtatgtgcttttgactatcatgacgatgaacacttcgtcagcgacaggcgtgagtgtcgtgctggaatcagcctggagcagctcggtggtcaccacagatatcaatgacacgtggcccgtctcttcgtcccctcttgacctggacaacatcacgctggacgacattgctaccactccctgggacaacactacctctcaagtctacaacatcctcaactaccccaccacgcaggctatattctacatagaatacctgaccatattcctactaggggtatttgggaactgcttggtgtgctacgtagtattccgcaataagtccatgcagaatgtgaccaactacttcatcacaaacttggcactggcagacatcttgctatgcgtactggcagtgcccttcactcctctttacaccttcatgcggcaatggatgttcggccgggtgttgtgtcaccttgtcaccatggctcaaggcaccagcgtctacgtgtccacacttacactgatgtccatcgctattgacagattctttgtgataatctacccgtttcgaccacgcctgcgcttgtcaatctgctacttgatcatcgtcagcatctggcttttttccatctcggcgacgcttccgtatgcgctgtatgtccggcaagtggagtatcaggaccgatattactgcgaggagctctggccttcagaatccatcaggcaggtgtttagcggttttacagccattatgcaattcgtcgttccgttcatcatcattctcttttgctacgtcaaaatttctatccgcatgaacgagcgagtcagggccaagccgggcactaagaacaccaggaaggaagaaacggaccgagaacgaaagcgccggacaaaccggatgttgatagcgatggtgacgatcttcggtacttcctggctgcccgtcaacgtggtgcacctcgtcggagactactatgcgccggcgagtgaatggagctactacaacctgtgcttcttcatcactcatgtggtggccatgtcttccacctgctacaacccctttctttatgcctggctcaacgaaaatttccgtaaggaatttcaactggttttaccttgcttccagcagactccctccactgaccgtgtaggtcagtggcgttcagagaggacctgcaacgggaacgacacccaacaagagaccctcctacaggcaggacaatgcggaggcactggtggagattcaataaggtctttcaaccaccagctgtcagtcacacaagagagcccgactaacatggcgtccccagccactgtcgagacccatcccatgaccaccttcatagaggctccgtcacacaccagcggcagcacacctgaccaggccgtccagtccctggtcaatggagaagcatccgagtacgtgtgaacacagnnnnnnnnnnnnnnnnnnnnnnnnnnnnnnnnnnnnnnnnnnnngaagatggagagcgtgtctgcggggctgtgaggatgtgtggctggctgcctctcttcggtttcatccactaatgtcttcaccgctgacatcgctgtgacggctaccaccactactgttgctgctaaaccccccatcattgtgatgattaccaccattgttttaatgagtacgaacatcgttgtgacgaatactactattgatgtgacgaatgactctcgctgtaacgactaccatcatattcgatacaataaaccaaccgggaagcccagtgttaccaaggttgtcaatatttaccactactaagtttaacaacttctgcctactaaacaagggcatctaccaaaattaccccttattatgagttgaattgtcacatttatccccacaaaagaggtaaataaccacaatttctacaaaatggttcagtgagaaattttttgaagctttcttacatgtaataagagagaacttaccaatgactgttatcaacaccgtgtgtgtgtgtgtgtctgtgtgtgtaaacggtcttggtcactatcacgttcattatcaccgtcatcattacctcagtcatgttttttttcttaatttcactttacgctttattcttagtacaagaggaattgctacctctcgacagtgttgctgctattggtcattatattcacttaacagttttttattggttattacctcaaagggtccaccaagccatcgattttttttttatctcattgtgtatcataaaagaatattttctccttcacatacataactttataagcagcaatgtccagaagggtaaactattgtagaataggttttagggtcaagatccaactaagaaacaaagatgggtgttcctctaggagaatgttgtgaaatgtggtgaatctgtttctctggtcctccaacacagtggattatgttctagtggtccttcaacacagtgaatgtatttcaatagcccctcaatacaagtggatatgtttcagtgatcctctaacatacttgatgtgtttcagtggttctccaacacagtgaatgagtttcggtggtccttcaacacagtggatgtgttttattgattcttcaacgcagtgaatgagttttggtggtccttcaatacattggaagtttcaatggccctccaaaagtggattttttcagttgtgtggctcaaggaatggttgtgtatattatcaacaatattagatgcgtttcagtggcacatgtccctcagtgtcgacaacagcagtaacaggggctgtgtcacaggaagctgcaataatggaagcgtctcagaggtattggttaaatggtactccctaataagtattagtaaaggaatatcgcaggtgagggtaaataatccctattaaaactacatataagccagtgaagatgctcaacactgatgtcactaacaatcgtgtatatgcttcaccatcagcagggatttagtgtttgcagtgtctccattcacacaacatgtacccatatactacaacatcgtcactctagagagaggagagtgtcaggacctggggaatttttctactcgtcactgtcagtttacggtatataggcctcacttcccaggaagtaaaagacacacgacacctctctcaacgtctcctctaatggttttctctttgtatcatatatatgtatatacattatttaatctgaagaaaatgagaaatctgtcggagtcatggctccaaaaacaggaaagatcataataatattctccctaatagatgtgtatgttatagttcatgatatgggacctgatgtgtaatacaaaaaaaatatgttttttgacgcaatgggaagctagatcgagttagtcctatgtgttaaagcaatgtttttacttggtaacagtgttaggttttacacccattcattatatgttattaatatgaatatattatataatgttatttatggtatgtcgc**

**V.** SIFamide receptor (DS01-Homarus1_Transcript_25154; translation frame -1)

**ctgatgtactgctcaacacacctgatgtactgctcaacacacctgctgtcccgctcaacacacctgctgtcccgctcaacacacctgctgtcctgctcaacatacctgctgtactgctcaacacacctgctgtactgcatgacttactgtaggtgctgtgcgtgatagttaataacacatatacgtcttacaataacaccaagagttgtaaacagagagtcccgcgaggctggatcacgactagtgagggaacaatgacctgacctccaggaatgaccagacagacaacgtcatagttttctgactgatgacaaaagaaccagagagagacaaagttgtagtaatggtcagtaaaggcgtcccgagtaacaggagattcatcaaagagtcgctttagtcttagtctacggcgtcgagtttgccgttggtgaaggtgtggatgctgtctgcatagctgttgctgtactcatccacttccaccacctcaatgcctcccaatattggttgtggtggacgcatctcctccaagctggcgtccttggtaaagttatctgcgaggtggagagcaggactgaggccgttggccagagtgcgtggccgccctgtgtggtacctggtgcgggtgttagctcgtccctctagtgtgtacacgttggcggagctgttcttgtgatgattgatggggtcgacggtgccagcttcgggtcggatggtggagcggtatgacggatagtaagacctacgcgtggtggagtacgagtaggagtcatagcggagagtagcacagcacgagcgactcttgacgatggccaggaacccattacggtactttttgttgaagaaagcgtagagaatagggttgatgcaggagttggatgaccccagccactgtgccataggggtgacgatggccagaactgctccctcggtctcaccaagctcatcaccaagcttgatcctggtgaagataacgtagagaggcagccaagacaacatgaagataatcaccaccaccaccagcatcttcacgaccttcaccttactgcgttgttgcatctcgtgaacggcggcgtctctgctgtctcctgggatgtgtctactggcaaccctgatccatatcattacgtagaagaagatgataagcagtaggggaagcaggtagcagaagagcaggtgggcgatgaggaagtagaaacgttctgcttgtctgctgggccacatctcaacacaaactatgaggtcaggagcttgcttgtagaacacctgtgtctcgaagtagatggagtaaggtatagccgaggcgacggccattatccagatgatgacaataagggccctggctcgttctgttgtgatctggagcttaagaggaaaccagatggccaagaacctgtcgagggagacggccaccagggagttaacagacgcagatacggacacagcctggacgtacgctaccaacttgcacattatccatcctaacatccacgggtagtatatgctggagagtagtgtggctggtagacagaacactatgaccaacacatcggccattgccaggttcacgatgaaatagttggtgggtgtgcgcatgcgcggggtcctgaagacaacagcgatgacgaagcagttgccgataagtcccaggatgaagacgacaacataactaagacagagcagaagacccgtgttgaaggagtgccggtacagaaggtcgtccagagacaagtcgtaatcgctgtaattggaagccagtgaggcgttaaggttgtcttggtagtcgaggtcctggtctccggttgtggtgatgttggtggtctggttgaggaggaggagggtggtggtagccgcggcgtggtgtggtgtctcagagttaaaggttgacaggtgagttaccgtggcaaccttctcgttgacggacactaacaaggtactgctgttgctgttggatgtgtccctcacgtttctcgtctctccgtgggatggatccgtccagctcattgactcgggtgtcccgtagagggtcgccgagacctcgctgctggacaacacactgactggggtgccgaggctgtgtacagccacacctccaggcgtcgccgtcatcacgttgtcctacaccacctaactcttcccctccgtgtggtgagccagagaaggcacacctctctgtcaccttcctgtaacacccttatctaacactttcctgtggcaccttcctttgtgacactttcctgtaacatccttacgtgacactctactgtgtgacacctttcagcagacgtttgtgactgcgtgttgcaggaagacacttacattgttcagtaaacacaaaacttgtcaacaacagttctggaaatttgtgaatcgatgtaaaataagttagtggcgaggaggaggtggcggggcgactgacttaactttcgtatttaggagaaaattcttaattttttaaaatataaatttcagtgttgtttccgcggaagtttctcagcaatactatttttcctaaagtttactggtaaattgtctgctataatacttactgagtgacaaacttggaacactggaacccgtgtatagtacagtgaggcaaagtgagtcactaaaccacctctgtcgtgttggtcaaggtgatgctgcagttatctcctacagcggccaggttgtggctatgggtgtagggtagtaggtggccctcaacactcagca**

**W.** Sulfakinin receptor (DS01-Homarus1_Transcript_21140; translation frame -2)

**gtcacaccgtggcgtcacaggtcgtcacaccgtggccgtcacaccgtggtctcgcgagtatctttctttatctccatggccaacttgaaggcgttgttactctggctcctgaagctggccgccgtcccgttcagcctccccattctctcgttctcctttctacagccgaaggcgtgcctgaaaccctgcaggaacttcttgttcatgaagcagtaagtgatggggttacagcaggaggaggcataggagagcaggtgcataaaggagatcccgaaggaccctagaactcggtacacctgctcggggatgtagaggcacatgatgttaacgacgaagatgggcgtccagcagaggaagaactccagcaccaccacgaacagcatcttgatcacatgtttcttggcctcgatgctcttctccaggtgggtactgcggaggcgtttgaaccccgtcttcggttctttccggtgtggtgactccaggttcccgttctcccgcaccgactccacgtgccacttgtagttttggcagtctcgtccgtgtaggcagacgtgggtaccgtcctgactggaggagggggtgtgggtgaggtctgtaggggacttatgagagtggacgacgacctctgggatggcgacgctctctggctgacaacagtgttcacacgtcctcgtctgggccgggatcttgcggaaccttatggagatcttggagacacgacgggagtgtttgcgtctcagactctgttgaaccatcgccaagccgttgaggtctgtgagggagattaagggtctggtaccttggccggtctcccgctcaagtctcatgccgtgccatagagagatggtgatggaggagtaggcagcaaccatgatgacgaggggcaccagcagcagccccgcccccaggaacatgctgaacccgcgctccaggtgaagactcggccacatctctctgcacttgtggcgactcgtatcgccgacggggagaagctgtgacagggcagctataggcgccatgaacaccagggacaagagccacactaagctgatgatcttgtaggcgtgtgagagggtttgccatccccgggagcgcagcggttggcagatggcgtaatacctctccagcgagatggccaccagcgtccagaccgacacagacaccgacacagcctgaaagtaagggatgaggcgacacatgactgggccgaagataaagtctctgagcaaggaaccgaccagggtgaaaggcatgcaaaacacacccagcaggaggtcggagaacgccaggttgaggaggaagacgttggtgatggtcctcatcttgcggttctgcaggagtgtgacgatgacgagggtgttgccaatcacccccagcaggaagatgatgacgtagcaggggatcctcaccgatccctccgtgtctagtaacggcacggaggacgcctgactcttcctcctgttggtgcttctgttgacgctggggattgtgcctttactgtgcttgctccccgggtcctcaacagcaccttcgtggggctcctcgtgcaggatcatggggtgatgctcaggtggaatattctgcacgtctgtcgctggctcttgtgactcaccgcgtcttgcattattcatattattgtcatctttctgtgtttttattttcagtaaatcagcagttctctctatcttgttcttcttatatatgtaatgtagtctgttgtgtctcgtcagttgggcgtcgttactcactatttgttccttattgatgtccatatgtgagtcagcctccaggttctcaacaattatctctgtgtgttcgtcgacgggggtagccttctcccatgtcggtattgatcttctttcttcgtcatttataccattgttattctcctcctctactgcctcaccttcgttctttctcacgtcagtagttggtgttggggacctccgccttgtgtgtaattccccgcgggtgtcaaggagtgtggcgtcagcgtcagtcatagactggacgaggggcggtgacacgacttgaccttctagccaattggttatgtcaataccgacctcacctgtgtggactgtgggattagcacctgcctgataacgtcgtaaggggtccacattggcttgttctaggctgtcgaacccctgatcagggttaaatggagagacgtggaaatccctaaacccggaattggggagataagttgaaacccaaatggggagttgtagagttgtactgttctctatggtgcccgcgacgatggcgttcacgaacctcttctcgttttgatgttcattgttctcttctgtttttgtttctcttgtcatcttttttttgcttatttcttcctctctatccatatatgtaataacttcctcgttttcgcctctttctctctctctctcgtaggaattaccagacacagacatcaccttccttgtctcaggcggtccatttattgaacgcctgtcgtgttcctttcctctcacgtgaggaggtttgtgtgtgtcctgactctcaccactggaactgtcctcctccggtaccctggtcaacactgtgttcattaagacctcactgtcgactttatcatcacgagtttccgagaaggagttgtgacttttactttttctcgtcacgtctgagttaacttcaccttttgctttcttatcaatgtgcttgtgtgttctaccgacagaggagataaaatcaccattgtctactgttctctcactctgtttgtcactactctcagctaccctaacattattacgttttagggaattcacaatgccgtgtctaatcgctaccgttatttcactgtcatgtaaattcttttggttaagtaaatcaaaattcacctcttcaccgctccttagttggggtgttaagtgctctctgccatcgctgtccaagccccgtatatttctgttcggttctgagaggtatttttcagaattctctttgttgagctgttcttcgctgcctctgctctttgtcgggtgttcttcaggcgaggcgaggtcactgcccgtcctctccgtcgtcacaactttaccgagtctactattgctgtcggctgatctgtccccccagagaggtgattcagaggcatctcttatactggtgacggacggcttatcaggaccgctcacagacttcagcgagtgggacctgacactcgaggaatccagaacagcgggaacttctgaatattccgaataaacagcaaatctcgagtacctggagagattcagtgaagcttcagggagaaacaactcgccgcggactcttgattttcggtgacgttcttgacgtataattctgacctcacccgctgttcttaagctgctgttggtgtggcggagacgcttgtagggactcgtcagcgtcgtgggcgtgggagccttaggtttgggcgcgtcgggagagtatgttgtgggcgtcagagagagtgggtgttgtggtggctttctggcgaacatatagttgtaggtggccccctggcggacgtcagaatcacccacggacttgtaagggaacccatgtgaccttctgctggggggaatcatgaatctcgaggaatcaatagggtgaccagcgcccaagatccccggcaggagaagcacgcaggagaggaacacgtatgagagaatcgaagatgaggggagaccggatgagggaagcaccagcacgataaagatcatcatcttagtaactcaggcgactgtaaggagaggcagaccgaccagccatggccacttgtgtcctcagtgacgaccaggaagaggaattcgtcgactgttgtacctccacctgacacacctgggagacggcttcactatcgtccaaggcgcgcgtcactaccggagtttattctaaggtatctgctgcaccttgcgggagtttgtttatccaggttgagatagtggaagatgactgttagtggaggcatgttacccgtagtctctctgtacaaaaacacat**

**X1.** Tachykinin-like peptide receptor I (DS01-Homarus1_Transcript_51762; translation frame -1)

**gaaaaaaaagttgtaccgggggattattattattcagctgaacgaataataaaaatgtcttcatgaaaatatatatgtttctacaatgtgtttatcgggttgtgataattgttaataaatgttatgatattctttgataatttagaaatagatcaggtatttttggaattatttattgatatgtgtttaaagtttgtacagaattatccagtggggaaaattcactcgatatttaatctagttatgataaggatttgtaatgtttaattttttggtggcttatatataccgaatatttaaggacatatttataaacatctatttaacttgtatgtatataatgttgtcatggaggtagcgagtgactatgacattctataaacataacttgaatatgaaaaaaaaaaaacatctctacaacgtttgtcacaggttatgagggcggcgcttctgaatacctctaaaaaaaatattgaagactaatgctgatattttataagtttgtgttgcgtgatgaaggagatattgatgaactatggcagtactgggtggtcgtggtgaggagagagcggcagcggggagtgggcggtgttgttgtgtttgaggagaggcggccgcccacccacacacctgcctgggtcaccgataagctgaagatgaagggcgaaggggtcatcgtcagcggagaagtcagaggagctggggtcagatgttttcacacagcttcgaaggttcccctgcatctcggcaaacaactgacggaagaagtacctaaacctggcgttgaggaggtagtagacaatggggttgatcatagcgttggacatagctagccaatagaaggccagatacatgtgctgtacgtagggtctggtgctgagctgtgggtgatggtgtaccaggataaagtacacgtgatacggcagccaacagagagcgaacaccccgaccagtagcgccaacatctgcaccactttccttttgcttttgaaattgtcggcctgtcgtgacgttttggcagagcgtcggtcgtacagcttacagcctatgacggcgtagcagacgaccatagcagtcatgggcaccacgtaggtcacagccaagatcaccacattgtacacatagtccatatgggagactgaagccaaaccatccggccacagaataacgcagattgtccgtccacccaggtcgtactggatggtggtggcgtagggcaggatgggggcagctatggaagccgccaccacccagatgatacacagcagcagtcgacacacacaggctgacatccgaggcttcagaggtcgcattacgttcaggaacctgtcgaaggagatgacggcaagggtgaagacagaggcggcgagggaagcatgagccacgaactgtgatatggtgcagtaaatgctcccgaacgcccaatgacttttcaacatgaagacgaagttgaagatgcagttgaagagggtcatgaagaggtcagccaccgacaggttcaccaagaagtagttggttgttgtcctcatcctctggttacccaacacgatcctgatgacgatagtgttgccgatacaagctccgagaatcatcaccccgaaggcgaggacccagagcacctgttggtatacgggcatctggaagggcagatcactagacgcctcgttctccaagtcatcgttgtgatatggagttgtctcgacttgaagtaagttgttgagaactcttcgaagagtgtgagggtcaggcgcgtccccctccatagacagcaacgtcaggaggtcagtagattggttactgtgtccctccagccagtccttcacaaggctgccgggggtcgcatcctccagcccacctcccaccatcctgctggtcttggtggccaaaatcctcagtgggttcagcgtgtgggtcttcccctcaatactaggggtgaagccagaacttgcttccataatgtgagcgagatgtttcctcagccaccttctggtagcgttactctcgcttgtagggcttacgttatcttcaccaacactccccatgggtctcacaatttgtctcacagtttggatcacaacttggctcacagtactacccactcaactgtacactcacttggttcactacgcgtgtgatgtttccctgaaagttaaaatcacaatcttccaaacgacgggcagaaatgtttcccaggaatatgtctcgttaccgaccaccaacaatggggtcagacaacatgacgtcaaaagttgtgtaccgaggataagtgttcatacaaactgctgcaggtcgctaacagaggcagcctgcagcacgtcattaatagactgttcctgcagtaggacagaacaactcggccgctaaaaaggacttcaacaagagacgaaagtggccgggatggagataagaggggaggttgattcacttataacacctgagccaccttaactccgggttgcgtcgcgtcgcgcctgggagttgctgggcggagtggaggt**

**X2.** Tachykinin-like peptide receptor II (DS01-Homarus1_Transcript_23908; translation frame 2)

**gggaacatttatttaaaagttcttcccacgaagtcccattggtatcacaaacgtgtagataatgatgatgaacactgcagagcgtatctatgatgataaaagttcacagccacaccgggatacctcgacaactaccaccaccatcaaacctacggcaccagacgctgaggtcctcgccaactacacacaaatttacactaacatcgtcggctgtctcgtgtccgagtctgatagtctcaacttgacccttggggaaggattttggctccctatcctctacgagtccttacagcttccctacaatgtcaccaccgctgccttctctcagccctacactaccacgacagtctcctacgaaacgtacagcttcgcaaatggcgacgacgaccaggtgcgcagtacgacctcatactggtcaggggaacaaccaggtttcccacgagacaatgaccagttgccattggaggtaatcagggagatatggcagcgatgcttcttccgcacccccgtcatggccacacaccggccctacttgctggtgtggtggcaacagttcctatggaccttggtattcggagccatgatggccatggccgtaggcggtaacaccctcgtcatgtggatcatatgtgcacaccggcgcatgcgcactgttaccaactacttcctcatgaacctgagcgcagcggatctcttcatgtctgtcctcaactgcatgttcaactttatctacatgttgcacagcgactggccatttggggctgtgtattgcaccatcagcaactttatggccaacgtaaccatctcagccagcgtcttcaccttgatggctatatcattcgacaggtacatcgccatcgtcaagcccctggaacctcggatgtcgaagacgtcggcgcgagtcttcatcttagtgatctggagctcatctatggtgctgtccctcccgtgtctcctgtactccactaccgtctccttcacgtacaaggatgacgaggtgaggagaggatgtatcctaaggtggccagacggtcagacaagcagctcccagcacgagcacatctacaacatcgtgttcttcatcacgacgtacttgctgccaatgctggtgatgctggtgtgttatttccttattggtcgggagctctggggtagtcgcagtattggtgagcttacagatcgccaagcggccagcatcaggtccaagcgcagggtggtgaagatgttcatcatcatcgtgacaatgtttgggctgtgctggctgccacaacaagcgttcttcctctacaccttccacaactcccagatcctcgacacggcccacatccagcacatttacctggccttctactggctggccatggccaacgctatgatcaacccggtcatctactactggatgaatgccagattcaagtcgtgcttcaagaaggtgatgctgaagagagggataactaaacagagagagagagtgagagaagccactacacactcacacgatctctccactggcaggttcaggtcgtacatcaagga**

**X3.** Tachykinin-like peptide receptor I (DS01-Homarus1_Transcript_X; translation frame -1)

**ccgtcgtaggctatccgatggagagtttctggagaccccgagcaggagaaccttgtggtgtggaccctggttacctcgactggctctcgctcgtagtaatgcacgcagggtaaccagccggagaacaccttcttgaagccctggcggaacnnnnnnnnnnnnnnnnnnnnnnnnctgttgttgagccagcagtagatcataggattgtacatagagttggacatggccagccagtatatggccaggtaggtctcctggatgtagtcggtgtgagctatctccggcatcagattggacaggatgaagtataggtgataaggcagccaacagatagcgaagatcgacaccaccacgatcatcatcttcactaccttccttttggacttgatgctctccacctgcctcggagtttgctcaccaatgctgcggctcccccacagagtcatgccaatacgcgcgtaggtgaagcccatacataacagcggcaagatgtacgtcaacaccatcagcactaccgtatgtacatattctgactggctgtagccttggtcaccatcaggccactgggcataacacactaccctggtgtcaccatccttgaggggcagcggggcggtagtgaagaagagcatgttgggcatggatagacaggtggatgatacccatatccacaccacgatgaggatagtggccttccgtcccatgcgaggccgtaacgggtgcatgatggcgatgtatctgtcaaaggagatggccatgagcgtgaagacagaggcgcagatggacagaatggatacgaactggctgatcttgcagtatatcctcccgaagcgccaatgaaggtttagcatgtaggtgaagttgaagacaacgttgagggtggataccatagcgtcagccacggacaggttcaccaggaagatgttggtgactgtacgcatgcgtctgtcagccaacacgatccagatgacaatgaggttaccccctgtagccaccacaaccatcccgccgaagaagaccgaccagatgagttgatgccaccagggtaattggtatttgttctcctcccctagggtcacgttccccgtgctgttggtgctgttcaacatcgtatagaccaaattgatcgtagggtcttcgtattcctgaccggtgccgttctgcgccatgtctctgacctggatagtgctcatattccaggtcgtttcataatctaagagctccataatatagagaagtggcagcttgtagggttcaggcggcaagtgatatatcttcccttgagaccacagtagaggcagattacagtgttttatttctctctagacgatagatagatattctcttaccttacctattacagttaaaccttcctacgttgtctataagtcatgttaaacgttcagtaaacttataagtgaaggatatgtatcacgtatcacaagagtatttacaccggagaattcccgccataaagtaaacacagagatggaggcttaatttcaggtataaaaatttctataagaagtttttatctcgtgtcaccagagatgcttgacacaagtgcctctagcctttaaaaaagtcatctcggggtagtttacatggtagcttcatcatcataaacaggtggtaagtgagacgagtgatgccatgaaaaaattatctaaagcctcttatcactcacgagttaatcggtgttgaagtaccataaaacatctcaaactcccattaactttacttaagaggtttttgacaggttattgtttgttgaggctgagactcggtgtgtggcggagcttacacaggagtttttggagcccaggagcccttgatggagcctatggtgtacattgtgtcacacttcgccatactcttcctcctactcagcttcgagacgttccccctagtcactccggcagtgttggtaatgtgagtttttgtaataaatttttaaatagttattggagggaggcgtgaggtgagagctgttagtgtacctggatcacttatcactccatcgtgtcagcgtctcatcacccacaacacaccgcagtagatctgggtgtgaatgaaacttctttctctcagtctcatatactaaacttcaggcagtgaacttttccatagtttataaacagcaactcgcagttcagctctgttctggggacccgcactgacctatgagagtccaccgccatgacttaactcacaaagacacatcaactcgggaccagaaaaggtcaggcaacagtttgtagtagatggtcgcactgtgagagagatgccgggactacgagcagcgcggcggtgtttccccgtccagccttcaccggcaactctctcacagtgcgaccatctac**
