# Supplementary material for: Neuropeptidergic Signaling in the American Lobster Homarus americanus: New Insights from High-Throughput Nucleotide Sequencing
Source: PLoS One. 2015 Dec 30;10(12):e0145964. doi: 10.1371/journal.pone.0145964 (PMC4696782; doi:10.1371/journal.pone.0145964)
Supplement: S4 Fig — In the full-length proteins shown in this figure that do not contain any uncalled amino acids (colored green), rhodopsin-like G protein-coupled receptor (GPCR) seven transmembrane domains are highlighted in black, GPCR family 2-like seven transmembrane regions are highlighted in dark blue, GPCR family 2 extracellular hormone receptor domains are highlighted in red, receptor L-domains are highlighted in light blue and green, furin-like cysteine-rich domains are highlighted in green and yellow, immunoglobulin-like fold domains are highlighted in pink and protein kinase-like domains are highlighted in gray. The “+” symbol indicates the presence of additional, unknown, amino acid residues at the amino- and/or carboxyl-terminus. (DOC) [file pone.0145964.s004.doc]

**A. Adipokinetic hormone-corazonin-like peptide receptor (internal protein fragment from DS01-Homarus1_Transcript_58353)**

**+DRYFAVLHPLKVNDAQRRGKIMLFFAWLISAVISLPQSVIFNVQPHPDYPEFYQCVTFGFFNANNSGQMMYTIFCISFLYFIPLSIIIIAYTRIILEISRKSKDTQHDYTREERYHGRLQLRRSNMSNIERARTRTLRMTFIIVMAFIWCWTPYALATLWNFIDPKTFYSMNKHLQDILFIIGVSNSVVNPIIYGRYSISCCRDVWTKSGEFCCYCCWCWCCSCASHQRLGSVPPTNINSCKSNGHCHVRSLLIEWNRSSQKSLAAPEGGE+**

**B. Allatostatin A receptor (from DS01-Homarus1_Transcript_30123)**

**MDYLAEMGKEGEGGGGGADTDEENGIEPFLTFLPPYILCNLTNYTNLPLCNNNTVEENPAFEYAIIVAIVVPIIFGIIVLVGLFGNTLVVIVIIANKQMRSTTNYLIFSLAMADLLFIVFCVPFTASDYILPSWPFGSIWCKTVQYLTYVTAYASVYTLLLLSFDRFLAVVHPIAALSIRTERNALYAITFSWILILTSCIPLYLCHGIKKQKFEGDVYFQCAFLDDEYNHMAFHIGFITTMYFVPLTVIVVLYLMILNRLWYGVVPGGSRSAESVRGKKRVTRMVVIVVVTFIVCWFPIQLVLLLKSLDLYEMTTFRIITQIAAQVLAYINSCVNPILYAFLSDPFRKAFRKVISCGPQRRTALNGRTDFERSMETRPLNPRPSPQSIPLINMVTSTHHLSPSAGPPLHRNTTTTTTTSFTNGATREETSNNSQTQDVFIGNKVVAQATNYGGFNDGAFNLHEQCE**

**C1. Allatostatin C receptor I (from DS01-Homarus1_Transcript_10681)**

**MNSNSTLYLDFDGNDSDSFPLNCSFLNMKDPDATSNCSLGVFTGEVSPIHVVASIIMQACYAIAFLVGLCGNTLVIYVVTRFSKMQTVTNLYIVNLAIADELFVIGIPFLMITSVLGYWAFGSIMCKLYMITTSLNQFTSSLFLTIMSADRYIAVCHPISSTKFRTPMISKLVSLTAWTTSALMIVPVFMYSNTLEVNDLINCNIFWPDSFGISGQFVFTLYSFILAFGIPLILIFIFYGLVLQKLKSVGPKSKSKEKKKSHRKVTKMVLTVITVYVMCWLPYWVLQLALIFSPPREGQSPFMVVLFLISSCLSYFNSAINPILYAFLSENFKKSFMKACICATRKDANNALAVENSVFPRRRGGSAKPKNAKAHEMESNDAYTTQCTRDEATTAITMTTRSTVSQMGRENGTEIPTTQL**

**C2. Allatostatin C receptor II (from DS01-Homarus1_Transcript_10638)**

**MENDTLSEPEDTIPLNCSYLLYGDLYNRSDFLNESNCTLGLFTGGKNEMSIAAIVITQMFYAITCLVGLCGNTLVIYVVTRFSKMQTVTNLYILNLAIADELFVVGIPFLMTTSMLRYWPFGSIMCKLYMITTSLNQFTSSLFLTIMSADRYIAVCHPISSPRFRTPMISKLVSLTAWTLSALMIVPVFMYSNTLQDNGLDNCNIFWPESQGVRGEIAFIRYSFALAFGIPLTLIFIFYSLVLHKLKSVGPKSKSKEKKKSRQKVTRLVLTVITVYVICWLPYWVLQLTLILSTPKQGHSNFMVVLFMISSCLSYINSALNPILYAFLSDNFKKSFMKACTCAARMEVNNALRPENSMFPLRQRGTSAKSRMTRRDRESGEGTTSQCGLSKEPSTAVTTTNARPNLSNNSGSSGDELTVRNGRSPGPRLPDLIQ**

**C3. Allatostatin C receptor III (C-terminal partial protein from DS01-Homarus1_Transcript_26036)**

**+IAVCHPISAPKFRTPMISKLVSLTAWMISALMIIPVFMYSNILNDSNEQASCNIYWPDSVGVNGHTAFTIYSFVLAFGCPFALIFIFYSLVILKLKTVGAKTKTKKRKSHRKVTKLVLTVITAYVLCWLPYWVLQLILTFSMPKQVQSRVMIIFFLISSSLSYINSAINPILYAFLSENFKKSFLKACTCAARKDINNALAAENSMFPRRRGGSCKPPRAKEPKEVASHDGCVREEVIVPLSATRRPLVPRDDAFLNGRPLTSHL**

**D1. Bursicon receptor I (C-terminal partial protein from DS01-Homarus1_Transcript_10555)**

**+GLRTNFAAIWANLAADFPPANTDLVDTTSLDLGEATAATHTLPLLPRHQVLCNPEPGPFMPCEDLFDWWTLRCGVWIVFLLALLGNGAVVVVLVFARAKMDVPRFLVTNLAFADFFMGLYLGFLAVVDASTLGEFRMYAIPWQTSVGCQVAGFLGVLSCELSVYTLTVITMERNYAITHAMHLNKRLSLRHAAYIMVLGWLFACTMALLPLIGVSDYRKFAVCLPIETKGAGLGYVVFLMFINGVAFLILMGCYLKIYCAIRGSQAWNSNDSRIAKRMALLVFTDFICWAPIAFFSLTAAFGLQLISLKEAKVFTVFILPFNSCCNPFLYALLTKQFKKDCVMLCKTIEESRVTRGIGRCRHSSNFSNRQTPANTNSALENSSRQDNQLCRCQNKTQESQKLHHRLRISALKYLFCHKDTEGLNSTSDFSYQPTKSAVKSKRHTSVSSETYSSSWSDTWRRGHAAMSLRILDRRHHNSWYLSRKPSQESNLSSSRNDSSATTASTSTWRISRSSVSSDISSSGSRGVGKSDVAPTLRLGSLRERRGECHIQIPTRQITHHHQALLVRQQSGASGQRSAPITSAVRIKPRLQRQSAIERETYIPNKAAGGQNEITCPLHQRSDNLSCVYEQESYEEEDHEASKDYLNPRCPMAGLTVTFIPRKLSTISSHSVSVVRDAEGDEPAVGPCVDVHSSSDPFPMSNCDFSRGGKCVSLTLLPQSSSQTSPSRFPSDGHLPRSPRCTELLYFTNLAAPALIVPSTEQNAESPPKDLDATPKNHYGQAILIHSQPRSPQSLEHDECMESTALMDDDCYGDDEVFEEENKSRERPLETHFPLDDPPGETRPLI**

**D2. Bursicon receptor II (C-terminal partial protein from DS01-Homarus1_Transcript_16714)**

**+PPSSSSSPPPPPRQHHLALPVTPSIRIRPQCIPLPGPFMPCRDLFDWWTLRCGVWIVFLLALMGNGVVVVVLVAAYAKMDVPRFLVTNLALADFFMGVYLGFLAVADASTLGEFRMYAIPWQMSPACQVAGFLGVLSSELSVYTLAVITLERNYAITHAMHLQKRLSLRQAAYIMAVGWVFAITMAVLPLAGVSDYRKFAVCLPFETDGAGLGYVVFLMFINGVAFLILMGCYFKIYCAIRGSQAWNSNDSRIAKRMALLVFTDFICWAPIAFFSLTAAFDIHLISLEEAKVFTVFVLPLNSCCNPFLYALLTKQFKKDCVMLCKTIEESRVTRGIGRCRHSSNFSNRQTPANTNSAVDHTSQGDKQSCSCKGKQEKNKRPRWTFISLKYLLCTKGAEEITSSSDTSYQTDPAQHRGPRHTSLSSDTYSGSWSDTWRRGRGSTTLRMMDRRRHNSWAASNKPSQESSLSSSRPDSSATSASTATWRISRSSVSSDTSNSSGRVKTSEPGGPSRLGSLRRGDGRGFGPTRQLSQSQFVKETPCTRPPPVRPKPRLQRQGAVEREAYTPGKENSGLESPPCPLHLRPDHLSCVYEQSHEDDNMAAGTSPGAGLPTQPTLLLSPCSLDTPMDDDVFTDPPDEEIQVPVGSSPSHRQEITLVMPTEEANEPAATENTALMAEGDTDAEKETSKEPQSNKVLETHFPLEATLPEVRPLI**

**E1. CCHamide receptor I (from DS01-Homarus1_Transcript_24860)**

**MEDLSSHQNSNMEAGSISNTTEKDQELSAGVVDVGPLTGGQTFLLDPSITTRAYLWLSTTIKNESWWGPSDDYSLSLNLTEEYFTSVNVTNDGIYENDTNVTTGYVPYPQRPETYIVPMLFAIIFIVGVIGNGALIVIFARNKTLRNVPNTYIISLALGDLLVLFFTVPFVSTIYTIESWPYGGFECKFSEFVRDISVGVTVFTLTALSADRYMAIVSPVKKAVGAARGVTVRTAVSIWVVAVLLATPSAVFSSVRVFKVSNVKSISVCYPVPENFIDWYMPASILTKALIYYLLPLIVIGTFYLLMARHLLASDVPGESHVFHKQIRTRRKVAKVVLCFVMIFAVCFLPTHVFLLWFYFDPEGSNNYNDFWHALRIVGFCLGFINSCINPIALYCISGTFRKQYNRYLFCCCWGRSGHRNINSLRSVRSSGSRYRCSTLRPSETITLTTLLHERTCAVSS**

**E2. CCHamide receptor II (from DS01-Homarus1_Transcript_37689)**

**MEDTAMLSPTLLFTNQTLDLDVERNTTHTNTLLDDTILDNTSWSFNYTNTSGGQDVGLSSNETYLPYRERPETYLVPIVFALIFITGVVGNGALIFMFLKHPKLRSAPNTHLVSLAAGDLLMVLLTVPFTSIVYTVSSYPFGEAVCRASEFAKDLSLGITVFTLTALSADRYMAIVRPVTHHVSDSTGHVAIAVAIGIWVVAALLATPAAIFSNTPELVNPKGEKFHICTPYPEYLGAVYKQVHALVKAIMYYLLPLALIASFYVLMARHLFISAQFLPGEAAGQQRQAQARRKVAKMVLAFVTIFAICFLPLNVFNLWWHFAPNSRETYDIYWHTFRIVGFCLSFINSCINPIALYCVSGTFRKYYNKHVFCWCTRHSGRRDWEGAESTGTRITTAVRTEQIPLKMVGTDNGTQQPPAHRLTLTNTTVLTTKPHHNPASLV**

**F. Corazonin receptor (C-terminal partial protein from DS01-Homarus1_Transcript_48447)**

**+MFSLYLSTFILVLIGVDRFTAVRYPMRRSDTLRHCSYGIIFVWVLSGILSIPQALVFHVVRGPFYEEFYQCVTYGLYSPAWLEQLYGVFSLVCMFVLPLLILLVTYVSTFVTLHKSEKVFRNERTTLGNTCPEFNRRRLLRKAKMRALRISVVIVMAFVICWTPYYMMMIIFMFTQVEENVAAELQSGIFFFGMSNSLVNPLIYGAFHLCRCHKRKASFNLIIINRSGSNLRYQASGRNSSRSTTCRSSAAEMDTSVVNVFEDGVRYSFRRQSSRQRALISPRSGPLETSIGDGPLRNSVRYGRRSKGLLDHHPRLSPKTSQNYGRHDGFSPLSQGLEEEEEEQEGLEWKQQLSRADSGVASSNGRRSSSDSWSRAAKIPADPLPSDSENEGVISSSKLLQQCPCNNYPLMKMDLQRHSDTQL**

**G. Crustacean cardioactive peptide receptor (N-terminal partial protein from DS01-Homarus1_Transcript_31037)**

**MKSTIMAGKVEKSPYNQTVTVDSQSQLSEGVDSKTKSNHNSLYVLEDAFIEVNCSDFMDLAAANGTCGNYTITSNVTNTTTTAGDSYYFYETEQFTVLWILFISIVVGNVAVIAALMLSKTRKSRTNFFIMHLALADLSVGLISVLTDIIWKTTLSWNAGNIGCKAVRYAQVLVTYSSTYVLVALSIDRYDAITHPMNFSGSWRRARRLVVVAWLLSAVFASPSLGFFRETSIDGVLQCWIDFSEAWQWKLYMTLVALTVFVFPTIIITACYAIIVYTIWSKSKIMTVNSKALGSKNGEKRVTSGEDDSRRASSRGLIPKAKIKTVKMTLVIVFVFILCWSPYIVFDLLQVYGYVPQTPTNVAVATLIQSLAPLNSAANPIIYCLFSTHICRNLRRIPVVDWVVRQVFPCLECAQQPVENRGAFHRYGTEYTTVSDASSRRHTLTSV+**

**H1. Diuretic hormone 31 receptor I (from DS01-Homarus1_Transcript_22256)**

**MKCGAGSVCRLLYLMLLLWTTDGSSRSTTTQESLHRGFLLDDTINDPGASSGDDHRLGLPGKYDLDLEYPEDDLHDLGKDTRERYILSDYQRDDLRLRESQQTLYKEYLKRASYRRSAELVPRTREEREGGDYTKHTTHDKQNTSVVSGNTQLQEKVVQEEEEAPADPLDDKNSAVGEESKTAGPLEEKEAGGGKVVDGDLHERMILSAGRPLCRSKEKYLPPDMYNADACALCYFHIVNRELFNTKWITLEVQKAVEEEDVTYNVSVLLNPMNFSQLEEAVVTSEAVLASFKGEVGPSKWRSCCEAAVQCCDQMLTASWPTGHCPNTWDGWQCWSATPRDTEAMRPCPSYIYYGREPSCAKQATKKCETDGMWFRRTGSGQEWSNYSSCSVEHNIHRRLYVHIAAYSVSVAALLPALCIFLSYKQLRVHRITLHKHLFLSLLLEAVGVITFRFLQLYKKDLINQNLTWCVTLNLLTKYTSLSNYMWYLCEGFYLHKLLASAFAEQNSLVIFYLIGWGFPLVPLTVYSVVRGLRDDNTDCWIMPNESLDWIINLPPLLAILINIIFVVNIIRILVSKVRATNGNEPSQYRKAVRATMMVVPLFGLQYIVTIYRHQELGCDWHDIYQIFNNVIEGSTGAVVAIIFCYTNGEVRSLLKRSWVRLQERRAPRGVRGPEKLRSRSLSTVQTTLLDPSPARRNSSIVSICANNQPFRRSNSGANASQLLSLRGSRTSITQQSQASTVDSGTAPITAPSRRSPKQIASKLGPGGSERFVSSKSPVHNLDSNPVSRSPSLLEEVEDEVKRSEKREKSPQPSAARLGGERVERHPVVLFRKDEAEPCVNINVNGQFCGLRTSVDQGYESSQTIAVLDE**

**H2. Diuretic hormone 31 receptor II (from DS01-Homarus1_Transcript_5552)**

**MEGNETDSVMDDWAWEEMSATKQDNSSSTEAGSSKGWEGTFKQKQEIARRQAECEALHATMPIPEPSFGPYCPRTFDGWSCWNDTPAGTRAHVQCPSFIHGFDPQRSGHKDCDENGTWFTHPDTNNFWSNYTTCIDVSNLELHQGVNTIYIAGYSMSLVALCISLFIFFFFKSLKCTRVTIHKNLFVSFIINNAMWLVWFECVVGRVDVVFSNTVGCQVLHVFLHYFLVSNYFWMFCEGLYLHTLLVVAFVAEDRIMKWFYMLGWGAPAVFTIIYGACRGTDDEQSVYCWMEDGNYNYILNVPVVLSMLLNLFFLVNIVRVLVTKLRAVNTAPDTHSTRKAVRATLILIPLLGLHYILIPFRPPKGSPAEGFYLVVSAIAASLQGLGVSLLFCFFNGEXXXXXXXXHEIEYG**

**H3. Diuretic hormone 31 receptor III (C-terminal partial protein from DS01-Homarus1_Transcript_26723)**

**+LPCTTGDIFKQPEDMSSLNRTLDMEGHTTTLLETVIDARYQLCVQLMKSVPQPPDGVVFCPRTFDGWSCWNDTLAGNTAYSPCPYFITGFDHTRMAHKVCNEDGSWFRHPLTNNSWSNYTTCIDLDDLMMRQLINTIYIAGYSVSLIALAISLVIFFHFRTLQCTRIRLHKNLFLSFILNNILWIAWYLEVAGKPETVFENKTGCQVLHIFLHYFMVANYCWMFSEGLYLHTLLVVAFVSEERLMKWFYLLGWGAPGLIVTVYAAIRGSSSSDTKHCWIDESHYTLILSTPVCISILANLVFLINIVRVLVTKLRANHVPADTNGTRKAVRXXXXXXXXXXXXXAGVQRGAGISGGDSPRLLLPGVLRCPSVLFLQH**

**I1. Diuretic hormone 44 receptor I (from DS01-Homarus1_Transcript_11147)**

**MPSITAPTEFPSYSLSDPAETEEEDSDEIYITLWRKFMEQSALINATNGDHKMLQCFNMYLNTTMDPESDPGACPVKFDGVSCWPETPPDTTRAIPCFNDFNGVHYEPSDYNATLYCYPNGTWSKKSFYNFCLNAVTNNSEVQGSTVNTISTIFYIGNSVSLVAVTLALWIFISFKDLRCLRNTIHTNLLFTYLLHNLFWIVYASVQTLVNVSVGCSFFVALNYFTLTNFMWMFVEGFYLYMLVVKTFSVENIKLRVYTLIGWGVPVPIIISWVILKSQLATTHPAGMEGHELEGLVRNCPLMPDSTVDWIQKIPVLFLLSTNLIFLTRIMWVLITKLRSANTVETQRYRKATKALLVLIPLLGLTYMLLIALPQELEHVRAILLSTQGFWVALFYCFLNSEVQNSIRHHIERWKTARGLADPRHASVRHGRDGSPRPKTDCSSYSRRLFGGKRESLCSEVTTMTTYVANGYNPVSTQTGGPPQQQQSLLQPAPQQPPTATGQHLTYRNSNAGIAPNSGGDPDVKSTVKDSLL**

**I2. Diuretic hormone 44 receptor II (N-terminal partial protein from DS01-Homarus1_Transcript_14267)**

**MFAMLSQGHLAALICLVAFMVTTAENGNIPDRGTEVNSGSPWSVSEKETQDTSWAPEGVDLQDTSSLDDEGAEGGGAGLAIDLSLFDSINGSSGEFNESWPEHKQKLWLKQMLLRREGECHLYQLLEANTTSSDLHQEGSWCPRVWDKMLCWAPTPPNTTITQPCPDYVPGLIAEARASRRCGDNGSWAGGQGLGWTNYTPCVGHVAHPTSITIRFTIIGEWLPTIKKMSVVGYSVSLATLVISFIILASLRKLRCPRNLLHLHLFGSFMLRALVVLLKSSLLMDGIALPHNFHLQDGETYYNDGSQTWACKLMICVWQYFILANYSWLLMEGLYLHSLIFMALFTDSSAITLYILLGWGLPLGCVGIWATLRATLDDAHCWTVNNVQWIFWVCIRAPVAISNLINFFFFLNVVRVLVLKLRSSISAESMKYRKLGKSTLVLVPLFGVHYFVLWSLSTSTNAYVEIVWLFLDQVFASFQGFFVAVLYCLMNGEVRQELRKLYNRWYKGDPLVVTSHSTLVSHTKTYASRGRTSLHSIHSQAERRDRQTPSPQMLRSNGGDGQSSRPRTPSPSPTPILTPSYVSRQQSK+**

**J1. Ecdysis-triggering hormone receptor I (from DS01-Homarus1_Transcript_30219)**

**MERVDYEDPFAEYRPQESLGITPGGPSFALLDATSQNPYPVSYPKSFLVSSYPPLLTTRADNITDLLYNGTDFGSETNTTTTATNMPVFPEYIRVVSTVFCSVVLVVGVVGNVLVPVVILKDRDMRNSTNYFLMNLSVADLLVLLICLPPVLIELHSVQDLWVLGYTMCKLVPYVEMSVVHASALSLVVISLERYHVICQPLQAGYRCTKAKAVVAITIIWAISFISAGPLLMIVQYNIARFYDGTYKPQCIMPIHGNWIKSYFFATSILFFFLPLFLLVVLYTIIARQLLVDTYELTHKKENPQMRARRQVVVMLATVVLFFFLCILPMRVLYFWIMTVPNETVTSLGIEGYYNLLYFCRIMYYINSSINPILYNMTSTKFRTAFRRVFRGKRGRLRRQHTYSNTSFNNPTVSNNLRLNYGTNCSMVYKTLFSKTVVSNQYSYASTGSNSSQVTRQTSLVSTNRSHVNTKDTFV**

**J2. Ecdysis-triggering hormone receptor II (from DS01-Homarus1_Transcript_6537)**

**MEFGLDLVVSTPYLECGGGECGGVVPLPSLVPPAHNTTSALLPWAPTTTTFSPAPLNGSTAQGALPNISFPAYMQGVYTAWCLVLLLVGLLGNVLVPLLVVRDRDLRGASTSVFIVNLVAADLLVLVVCLPALLSELYAPPAVWILPPSMCKVVPYVEFTVAHASMLTILAISVERYRAICHPLTAAATCSRARAAVACFLVWVLATSVTSPVIALTEYTHVRYIDQSLVPVCYTRVDILWAKVFVVSSMVVLFFLPLLVLVVLYWRIARQLLLEDKQLCKDKPNPNLQARKQVVVMLGTVVVVFFVCLLPHRVFSLWFIFTTKESEQSLGQEVYYNLLYAFRILVYFNSAINPVLYNVTSSKFRGAFFRLVGVRRGEQVRWSVQQTATNNSTVSNTTLTSSLNKSLPPWKSQRLLVRCSYTCLRGEGETCPLVDRRPVSPAVWGNTNHAHQPPSPATSPFLARSSCTRDAVRQIESFV**

**J3. Ecdysis-triggering hormone receptor III (internal protein fragment from DS01-Homarus1_Transcript_54675)**

**+MMGLDNSSLVVEEDGGNFSILDNLATHPNKSLWPTGPVIHLGVDVELFPEWAVGVWTGVLVTLMTVGVGGNVLVPVVVMRTRDLRSSTNLLLVNLAAADLLLLLVSLPTALIELHSRPETWVLGEPMCKLVPFVEYCVCHASVLTILVISFERYYAICRPLRASYTCTKMRACTCILTIWAAAVMLSCPMLVMSQHKRVPYVDGSKVTVCFTNLTSFWATLYINLVTAIFFFVPLVLLILLYLVIGRSLMQDSASAAL+**

**K. FLRFamide receptor (from DS01-Homarus1_Transcript_7458)**

**MTLNTTLLETMEEEFSTEGSIMDNETLMEVGDLMCLETYNATAPSPRLLRFIVYGFLLTTVGLLGVAGNLISITILSRPKMRSSINCCLIGLTSFDMIVTTTSVLMFGLPEICEYTHTMLWYSEGVYQRVTPFVFPLALVAQTGSVYLTVTVTVERYIAVCRPLRARYLCTYGRAKVYVISVALFSILYNIPRFWEVSNKECNVDGETFVIVVPSALRLNPYYIEIYIMWVYLIVMYLVPFLSLMIFNFFIYKEVRAANHERQQLSRLQRKEIGLAVMLLVVVTVFFVCNVLAFIINVLELLDITIAELTMSSNLLVTINSSVNFIIYCIFGQKFRKLFLRMFCSGFLFCARGRDASLDSAAYGNNSIYGESRTLTNGKMTQTIRLSSWNGSHQSGRSLHPHDIHGHHGHSCSGTSWRASRYSSIPLGDSSTPRTASTSFTPSRSPSLVPQRTPSENVHVL**

**L1. Insulin-like peptide receptor I (from DS01-Homarus1_Transcript_11179)**

**MMTEHFKACSNDCLFATTSIECRQKSYSRECRRSMVMCGLQGVVPLLVLTVVMVPGSVATVIQPQDSSHHICETIEITGNASELEQYQKCSVIEGHLRLQLIDDRNLTWPHLGLPNLTQVTGYVLLYRLSYLRTLRHLLPRLAVIRGDTLFHGHALVIFGNIYLQELGLANLTHILQGSVRLAKNWSLCPGMDNAWRSLTTDYHDNVIEDNYGMCIYNPCEERGNCVTKLTHLHTRCTAYGTCHQGEECHDECVGGCLKPQDASACVACRNYQDGSTCVAACSPTSSNPVHHMGYRCINRESCISAGWKLYTLEDADERQSKNVCVRCHGDCTTTCKGATITSSDPGRWLRGCQRVEGNLQISVDGGWNLMEQLEENLGELRVVTGYIRVYGSNTLFSLNFLRNLELIGGEDRVHDKYALYILENENLQELWDMSQNNHSLVIAQGTLFSRYNPLLCPKLVHQLANLTGVTLLSPDDVSNDSNEDVAPCYGTDLSIEIQIGVKLGTISVSWTHNTVFDHRYVIGYYVYYRETDANVTYYQGRDACNDKLLWERYFLNFEKKRSNVMLRGLRPFTRYALYVSVHNIDTEKTATRSAIHYITTPPTDPSPVTKLKVQEKGASYLVLTWLEPSTPNGRVALYEVLYQVVRYPASTSSDYNRSYVCSDDYEPPGGIGKLSGRKEATTTVAPDANRTEEQEGGEDAPKCCPCPVSHSVVTKEDRQYDIEFYDYMSSLIYIKLDDEDEERSSSTTMLAWSGASASHLQREEVVPPASANTNTGSEDRVKRQASVSWSKIGHTYVGPHLSSTNGTESITKWTSTNTTTINITGLHHFTNYRVWVRACHTPVNREALCSIWTNLESATLFQESRNKILIFSANEEEPSEVTSGEQENQLREKHGQNFSDYSIVSENSSETNTSDYSIFFSITSAITASSSFSTISSPLTPSSSTSTSTSRPPTRSSSISTISTLSTSIPSLSISSTTTTTGSNILLRWEPPENPNGPPLAYLIQYSHKEAPQGKSKLMECLMEEQARREGYTYRLKGLQPGTYVFSIKLRSLGGDGHYTTRTIQISEFFWYWPFLVALLTVGMVLFCLVTMTICLWGRRKAANPTTPEFMNHIPNCEEPLLGDTLQHIKEEYVIQRDALEINLEHQLGKGCFGTVHKGVLSVTSGSDVKVAVKALSNQATGWDVKRFLQEAVFMQDINSNFVVRLVGVVVKFSPIYVVMELMERGDLKSFLLTEAGGTLTEMKMVELALEAADGMAYLADKKLVHRDLAARNCMLDTNLTLKIGDFGLTRYLKTDYYKKSDKGFLPVRWMAPESLQNGRYSSRSDVWSYGVLLWEVVSRGALPYQGFAHEVVNVMVISGSRLECPENGPEILKFLMQQCWKGQPKERPEFIQLVRLLLPRASPDYQARFERVSFFHSSSCRDSKSTESNDKGSIASGSLGPSLEDAEHSFSTSLDHNHYDSLHNLDDHGTEDDTVVDDDMLCLTQDDPYKRMSCGPLSCITTSNHFSSRYPSHHTPCLT**

**L2. Insulin-like peptide receptor II (C-terminal partial protein from DS01-Homarus1_Transcript_3743)**

**+SSMEVRIPKAMVSLEVWIPPGYNVESLTTDFETSSYLGSGGRFGWFMPKRLLRPDFIVEHWRSFTSPNSPTLKLFSRTQDELNTLSFKMRDPTTGEFYCPESLGCEDGLYVPSRCSAHQPCAVLFASYADYNVTKFLRGQIEAMKAYVIVSWIGPNLNHKFIKNNSDLSGLEGEEERSILIFHWWPSVLLQPFDFISVSFPPCIDRTIKADGDSPYQCKYEMHRFHKFLWKKLKRYAKFAYNAFHKVQVNHTDFMDLLDTYNMWKNKSSLALDEVACHWMKNNKQRWLTWQPGSTLHQLKMVGLFPISTEKKNRRKFIAPGNVPAYFMAVEAVNKNKSILTDFEIHPIVLNGACEPAMVMRQFIEILQMSSSQGFYNNMIGFVGPACSDTVEPIAGVSKYFNVPIISYGAEGAIFSDQDHYPYFFRTIPENKIFRYVYSSYFKREGWKRVASLTENGQRYSEYLTLLRDKLQEDGIELETLKYPQERKTPDMTQYLLDLKNKNYYIIIGDFYQDVAREVICDAYHLKMTGHQAYLWFLPHWFSADWYDVDKVPSYTKCTTAQMHSALQGHMSLSYKYFAEDNDIMQEGITVAEWRKLYSQRINTTVKTKNASESDYAGFTYDAVWTYALALDNLFKKDPSYAADLRATSTIGAFMKEISAISFNGVSGHINFSSGASRMTDIIVWQFQNKEYVEVGRFHPPPSVSDTGEDKEIDFNFKINKDRFLWPTGEKPVDGSTCAIQAFSEFVNLECAYALIVLCTLCFGGLILLLFICFCIFKRRYEKKLEQIQELWRRRPLFEIFDGWEIPRDKVVINRKLGEGAFGTVYGGECQFDTEGWVAVAVKTLKVGSTISEKLDFLSEAEMMKNFEHENIVRLLGVCTKTEPIYTVMEFMLYGDLKVYLLARRNLVNEKNRNDDDEVSNKRLTSMALDIARGLAYLADLKFVHRDVACRNCLVNANRTVKLADFGMTRPMYENNYYKFNRKGMLPVRWMAPESLTEGVFTTMSDIWSYGVLLYEIVTFGAFPFQGMSNDQVLEHVKAGHTIAIPRGIKPQLDVLLKTCWHRVPSRRPQVLQIIEHLIAYPRLISPSLDGPQSSVQIEDTVSLEMRIPDKTRKLSLSINNRLQNVASSSRKRSMSGNMVMNIPPLTTSLSEDGMISAHSNLDALNLNHVMVEENEMGEDPLLPPAQYVSSRYMSLTPKEHKEKEKESLVSRQQGDYCTTDISRDLWTNVTPV**

**M. Leucokinin receptor (from DS01-Homarus1_Transcript_14169)**

**MSLSYDDPYGYLLQNLTLSYPNINWSYINFTNLTDFTFLVDTTERVTSNSETITNSTETALYEVPTGIVVLLSIFYGSISLIAVVGNALVMWIVATSRKMHSVTNYFIANLALADIIIGLFAIPFQFQAALLQRWNLPEFMCAFCPFFQTVSVNVSIFTLTAIAVDRYRAIVFPLNARPSKFRSKVVIASIWLFSATLAIPNAIALRVKYIVDPRTKEDIPFCGVGGIDPVVMWTYSHVMVGLQFFVPLGIISFAYIRMGWELWGARTPGNAEDARDAHVLRNKKKVIKMLFIVVALFAFCWAPLQTYHILQEIYPGINDYRYINIIWFCCHWLAMSNSCCNPFIYAIYNEKFKREFRQKFRWLFKEENTGETSEFDRSRYQMSFRFRYPIERQVSTASDQRISRSTDATMLNGSSRSSSHKSTLTVQGRPSTTSGGVRTINGESLGDHQEVHVCVPLKSHSHLHPSVYRPRVVGDTRI**

**N. Myosuppressin receptor (from DS01-Homarus1_Transcript_42439)**

**MEQVEAAGGPDAAVTLTFITALVNATIDPSTYSDYIEDYSYSYNDTENATQPDLAANEKYCSTEGWNHFRESYQAVHGCMSLVVCVFGSIANVINMVVLTRRSMLSPTNAILTGLAMTDLLVMVEYIPYTMHQYVWQGRPLTSQYSWGWAVFVLFHAHFAQVFHTLSICLTLILAVWRYIAIAFPQNNTTWCSMQRTSRIIVVSFFCSVICNIPNYLNFTISTIEYNGQTLYIVGFSELAQAHGDLMKSINFWIYAVILKLLPCGALTGLSFALIQELLLAARRRTQLMKRNSAGRASDAGRQADRVTKMLLAILVLFLASEVPQGILGLLTVILGSEFFPCYQKLGEIMDMLVLFNSAINFLLYCAMSQQFRDTFSNLFKPCCVSVLSIRTPRYILSWKAVPSADPGLESNNTCITHV**

**O1. Neuropeptide F receptor I (from DS01-Homarus1_Transcript_40414)**

**MDQGPDISVASQELPSDGFGSPEISSLDFLTLANFDNNLTNLAHNFSWLFNVSEGLNIDLINKFQRNRRVNDGAYYALIVVYSLLIVLGSTGNSLVVVAVIRKPAMRTARNVFIINLAISDLLLCLVTMPLTLVELLSQYWPLGDHPFLCKLVGTLQATSIFVSTISITAIALDRYQVIVYPTKNSLKTVGAVLMLLLIWVISFILALPNFIWRTLKHHVINLPNLYSINFCFEEWPTEHGRGYYSVFVILVQYCLPIVTVSVAYAMICRKLKFRMANSTVRSSKKGERDDRRMKKTNKLLITIALIFCLSWLPLNLYNLVVDFHNPFGDDMETMLVVYAVCHMMGMSSACSNPLMYGWLNDNFRKEFLEIFSRVLPCREPQPQLQGSRRGQALVQEKTTPTPSKLPATPQGKDERPKVLYMKAKEAVEVNGTCDTQGDETYITQVVTNATL**

**O2. Neuropeptide F receptor II (from DS01-Homarus1_Transcript_17633)**

**MFTLEWQTAGVGWKRLDGPPPSLNPSIMALDSLDFDSEDFNLMNLAALNENLTSLAHNLTHLFNLTGGINVDLLKKFEKNRRVGDGAFYTLIFAYSVLILLGATGNSLVVMAVIRKPAMRTARNVFIINLAISDLLLCLVTMPLTLMELLSQYWPLGDTPFTCRLVGTLQATSIFVSTISITAIALDRYHVIVYPTKKSLQKVGAVGSLLVVWLLSFLLALPNFIWRTLETHHVNLPGIEVVRFCFEDWPFEHGRAYYSVFVILVQYCLPILTVSIAYARICNKLKYRMTNASSRSARSRKEDLRMKKTNTLLVSISLIFCLSWLPLNLYNVIVDLHNPFGEDTESMLIVYSVCHMAGMSSACSNPLLYGWLNDNFRKEFLEIFGVVCPCCPVVTNASRMNSLKTSRIGKEGGSLKSLPLCNHPVVLYTKAPEEQTCNGLSVDLQDQEVTFISQVVTTTTL**

**O3. Neuropeptide F receptor III (from DS01-Homarus1_Transcript_9534)**

**MLEESSHGGEGGVVGEKDDGENVFEEVSGVEVSCRDHGACGGFSPQEDLTNPFLPLEKLGKLLLEPSLPNNTNTSSHHPLFNFSIHEAYDIISDTQAGYLDGVTEVVFIVCYVSLILFGVGGNMMVGWVIWRKRTMRTPRNLYIINLTVSDLSMCLVCMPVTLVGLLYKNWGMGSLACKLVPVLQGANIMVSTSTVVAIAVDRYATIVKAGGSTRNKFHVAASICAIWVSSVLFALPLYFYYIVAQVKLQHILLYSRCVDHWPSRSAKNVWIIALLLTQYGIPIVVLSVVHARIKRYLSQHMMGQYDARRAQKEIERNRKTTILLSTIAVAFAVCWLPWNIVNLLADFEYEGFKDPTHLYTVFGACHMIAMSSACINPVLYGWLNTNLRRELLEFLPPIFAKMGWILPESFRRRTGDSPTRQPESVTLLVFQGNQNNSIQTVAHPPQTITTTIIKDDT**

**O4. Neuropeptide F receptor IV (from DS01-Homarus1_Transcript_16169)**

**MENISLFSEPELCSGVMCPLQPDEYKALEGHVMSGTTIGEEDNPFTNQLESFFKSSVLPTGANQSLPWEEIMKILKETNQKSYLGTAAKVSLITIYSLLITVGILGNLIVAFVIGHRPELRTARNVYIINLAVSDVSMCLVCMPFTLVGLLHKNWSLGNFICKLVPVVQCTNILVSTATIVAIAADRYLTIVCVQRNRDARAYIPWSVAAIWLVSLVFPLPLFAYYFVEKVQIKDYLLYEKCVESWSSPVVKYTWNITLIVMQYIIPILVLSFVHGRIQNYLSSHKMSQRDARRAQREIERNRRTTILLTSIAVTFAICWLPWHVVNLLADFNYAGFQEPEYFYVVFGSCHAVAMSSACINPILYGWLNTNLRKEMTQAIPILLRKTSCPLRRSGNTGGGGVTGTNPTVESVSLLVHRPLRDDSCPGKDVPEEGGSKKNHHQTKTPFLKTESAKIGEVTDQNHDET**

**P1. Pigment dispersing hormone receptor I (from DS01-Homarus1_Transcript_14445)**

**MTYSIYLQACVDNYEHVNLSTHESWCNATWDLVLCWPPTPAGGSTRLPCPPVKGVDPSKYVYKHCDGSGRWTGKTPGDFTLPQGWTNYTVCFTKAIQEIMQELYKQSDEDAQTKLNIALGTRIMEIVGLSLSLASLCASLAIFFHFRSLKNNRTRIHRNLFVAMVIQVMIRLVLYIDQAIIRGHIVGHSPTNTNAPRQGIDNTPMLCEASYVLLEYARTAMFMWMFIEGLYLHNKITVTVFHHKFYYSVYHAVGWGVPVLLTAAWATATAMHYGNSRCWWGYNFTSYFWILEGPRFSVISMNMVFLLNIIRVLVTKLRQSNSSEALQVRKAVKAAIVLLPLLGITNVLNMVAAPLGRSAAEFGLWSYATHFLTSFQGFFIALLYCFLNGEVRTAVRKYVDNYLLHRSAGVRRGSGLSSVFLTTVTDLPRDRHGRTRHLCACLRGNHSPTHGSYL**

**P2. Pigment dispersing hormone receptor II (from DS01-Homarus1_Transcript_11293)**

**MDGSGMRAWEFTSYDQCVLYYQDQAQDGWCNATWDKILCWPPTPADTISRLPCPPLKGVDPSQVAERHCVAGGYWEGRTPGEVYTGGWTNYTTCLIPEIRILMDKLYAKSKEDAQLKLQVAEVSRVIETVGLSLSLASILVSLAIFSYFRSLRNNRTRMHFNLFVAMMIQLVVRLTLYIDQYVTRKTEQRSTGIDNTPVLCEVFYVLMEYARTAMFLWMFIEGHYLNSMLTVAVFTDQPNHTIYNLFGWGVPVVMTTVWAAVTAVQHTGTECWWGYNLSPYFWILEGPRLTVIITNFLFLLNILRVLITKLQASVSSETQQAKKAVRAAIVLLPLLGITNSLQMVHSPLEGNIVEFAAWSFVTTFLTAFQGFFVALLYCFLNNEVRGTITKCVANLYTQRLLPKRSKRSCINGNIASEAIGAEEPLTTFREEITIEHSEEPDVITTTV**

**Q1. Proctolin receptor I (from DS01-Homarus1_Transcript_26916)**

**MEVLEMFEWAPSGVINNSILLEDPNSTSVLLPYGHNTSLFNLDGDNYPYYPEDQNTSYISVEHHVGPPDKSSIPSTTSSNYASYERFMDESRHWVQRVLVPLVMCVGLVGNAVSMVVLTRRKMRSSTNNYLTALAISDLLYLVFVFSLSLQHHPDIKQPRHWFYWQYFRYGLWLTDASSSTSIWITVTFTIERYIAVSHPIKGKVLCTVSRAKKVVTVVYFLCFALTATTPHEWVVVTKTRPDTHQPYLALDYSSLGQDSTYRHTYYWFTAVTFILLPLCLLAVFNFFLIQAVRTSKLHRRKMTLVSERDHYSHQQEYKITVMLIAVVILALVCQMPTAVLLLYSTVYEPPPKTKTYAIMRGLGNIFNLLNAVNAAANFILYCAFSDRYRRTFLMTFVPCMYHQQPLAHSFVTSVTVGGSGGGGCVGGRGSDTTSIKSVCRRVSKSSTPSPRPPRDHSPRLASGSFKENGVGSLDARVTLQYGFGRSPRHLTLSPADAHPHVNNAHVRVNKHSHVSPISRNKRATQAFDNNINPGPSPELKKVDVGNHCEVSDEKVDLEVRKKYKEDEGVIIIEKAPTPSSSVSSVEENNLLEFKFIDDESCETSQSSPTSTTRAGEDENGDADGHLPSITLQKGDDSHLVNTSPLTPPKSSDTTNESTIHSPD**

**Q2. Proctolin receptor II (C-terminal partial protein from DS01-Homarus1_Transcript_45976)**

**+LAPPTLQPPITTDPYGNFSYYYNSTDLEATEASEQEIVDISGYEAFLDISRYVVQRVLVPMVLVVGVVGNAVTIVVLTRRQMRSSTNNYLTALAISDLLYLVFIFSLSIRHHPGMSRPHHWFYWHYFRYALWLTDASSSTSIWLTVTFTIERYIAVCHPIKGKVFCTESRAKRVIVAVFILCFALTATTPHEWVINEVTDATGQARLVMNYSVLGSNATYKKVFYWFTAVIFILLPLVLLAVFNSFLIHVVKLSRAQRRTMTNHRVERDNHSQSQENKITIMLIAVVLLALVCQLPVAVLLLYTTVYVSEPHSNSQYVELSLGNIFNLLAAINAACNFVLYCAMSDKYRRTFLRTFCSRWYRQPSPLHSWMATAYSNVEDGSPRFSRMSSMRMSRRSSYRHPREKTAATTGTRV**

**R. Pyrokinin receptor (C-terminal partial protein from DS01-Homarus1_Transcript_40714)**

**+QTSTPRNTFKKSLQTSAPGMSSPSQPERGVVVNLTEEVLGAVMTGVNNGSGGTEVNDTFDEHEYISRELGPQRVSYNTLLPLTVVYCVIAVGGVVGNALTCLVVARNHSMRTSTNYYLVNLAVADLLTLCLALPIEMYQMWVQYPWPWGDAACKIRAILPETLAHVSVLNILAVTGERYVAITDPVYARTTHTLARTARVLPVIWIVALLAATPWGYYQQVNLLLGPFGSLPQSAWCAIPYHDTSTSWSWLMWVSSVGAFILPMTILMTLYCKIGVVLSIDPPTRTPAAGAGAMHTRKVGIRMLVAVVVAFFVCWAPFHAQRLMFVIVTSYGKWTAHLRSVNTKLYYFTGICYYLNSAVNPILYNLTSTKFREAFLKLLSNDRRRRHLSRQSTFNTTGTSMSNGRSGSSRTIPTDLGSLKDATYPARVALAKCGRHASFDDFPAPRPNMARYGRQSSFAGTYCLPNSTNSNSNSVACSRQNSRAGENGVLSPLEARRSVEGEKRHSGEGRRLLEGERQSSSAFNTDMIEEERSSGGVSRLDSVRRDQQNKIATEMEKLLSDECLNTEAGDSKTMAGAPGDAVNEMNLKQGTGECKVEVEFPSNNDGVTSQDPGERGTTGKQDIASSSKENGDKDTITNTDNGKSVQFCDGVSTEFSVV**

**S1. Red pigment concentrating hormone receptor I (internal protein fragment from DS01-Homarus1_Transcript_57704)**

**+KMPPEMLFNSSHVITISAYSCLMVLSALGNISVLRSIAGHRSRTLASRVTLMILHLTIADLLVTFLLMPLEIGWAWTVSWEAGDIACRILAFFRTFGVFLSGFLLVAISVDRYYAVLRPLTVMEAKRRVRLMLWGAWGASGVCSIPQTLIFHVESHPDHPWFEQCVTFHSFPSPTYELLYNVAGFLAMYAIPLLTIIFCYGSIVIVLYRKDVSSREAGEERYGPSLGRTKTRTLHMTLIIVTVFFLCWTPYNIMSLWYFIDRQSAQLVDPRVQASLFIFAVANSTVNPLVYGYFNVRRSPKNANPRQEWRM+**

**S2. Red pigment concentrating hormone receptor II (internal protein fragment from DS01-Homarus1_Transcript_51875)**

**+EMKIGGGVSNWLERAFNDSECVIVPGNSDCQINATLTLGNFTTPIINSTSTVLPPSLGFDDNAMTDIIIYSVMFVVAAVGNLTVFITLFRNRHRKSRVNLMIMHLAAADLMVTLINFPLEVGWRITTQWVAGNLACKLFQFLRAFGLYLSSLVLVCISLDRYFAIVHPLKVNDAQRRGKIMLTFAWSIAAVCSLPQVSLTHTHTHTQTHTHTH+**

**T. Short neuropeptide F receptor (from DS01-Homarus1_Transcript_35046)**

**MTMNTSSATGVSVVLESAWSSSVVTTDINDTWPVSSSPLDLDNITLDDIATTPWDNTTSQVYNILNYPTTQAIFYIEYLTIFLLGVFGNCLVCYVVFRNKSMQNVTNYFITNLALADILLCVLAVPFTPLYTFMRQWMFGRVLCHLVTMAQGTSVYVSTLTLMSIAIDRFFVIIYPFRPRLRLSICYLIIVSIWLFSISATLPYALYVRQVEYQDRYYCEELWPSESIRQVFSGFTAIMQFVVPFIIILFCYVKISIRMNERVRAKPGTKNTRKEETDRERKRRTNRMLIAMVTIFGTSWLPVNVVHLVGDYYAPASEWSYYNLCFFITHVVAMSSTCYNPFLYAWLNENFRKEFQLVLPCFQQTPSTDRVGQWRSERTCNGNDTQQETLLQAGQCGGTGGDSIRSFNHQLSVTQESPTNMASPATVETHPMTTFIEAPSHTSGSTPDQAVQSLVNGEASEYV**

**U. SIFamide receptor (from DS01-Homarus1_Transcript_25154)**

**MTATPGGVAVHSLGTPVSVLSSSEVSATLYGTPESMSWTDPSHGETRNVRDTSNSNSSTLLVSVNEKVATVTHLSTFNSETPHHAAATTTLLLLNQTTNITTTGDQDLDYQDNLNASLASNYSDYDLSLDDLLYRHSFNTGLLLCLSYVVVFILGLIGNCFVIAVVFRTPRMRTPTNYFIVNLAMADVLVIVFCLPATLLSSIYYPWMLGWIMCKLVAYVQAVSVSASVNSLVAVSLDRFLAIWFPLKLQITTERARALIVIIWIMAVASAIPYSIYFETQVFYKQAPDLIVCVEMWPSRQAERFYFLIAHLLFCYLLPLLLIIFFYVMIWIRVASRHIPGDSRDAAVHEMQQRSKVKVVKMLVVVVIIFMLSWLPLYVIFTRIKLGDELGETEGAVLAIVTPMAQWLGSSNSCINPILYAFFNKKYRNGFLAIVKSRSCCATLRYDSYSYSTTRRSYYPSYRSTIRPEAGTVDPINHHKNSSANVYTLEGRANTRTRYHTGRPRTLANGLSPALHLADNFTKDASLEEMRPPQPILGGIEVVEVDEYSNSYADSIHTFTNGKLDAVD**

**V. Sulfakinin receptor (from DS01-Homarus1_Transcript_21140)**

**MMIFIVLVLPSSGLPSSSILSYVFLSCVLLLPGILGAGHPIDSSRFMIPPSRRSHGFPYKSVGDSDVRQGATYNYMFARKPPQHPLSLTPTTYSPDAPKPKAPTPTTLTSPYKRLRHTNSSLRTAGEVRIIRQERHRKSRVRGELFLPEASLNLSRYSRFAVYSEYSEVPAVLDSSSVRSHSLKSVSGPDKPSVTSIRDASESPLWGDRSADSNSRLGKVVTTERTGSDLASPEEHPTKSRGSEEQLNKENSEKYLSEPNRNIRGLDSDGREHLTPQLRSGEEVNFDLLNQKNLHDSEITVAIRHGIVNSLKRNNVRVAESSDKQSERTVDNGDFISSVGRTHKHIDKKAKGEVNSDVTRKSKSHNSFSETRDDKVDSEVLMNTVLTRVPEEDSSSGESQDTHKPPHVRGKEHDRRSINGPPETRKVMSVSGNSYERERERGENEEVITYMDREEEISKKKMTRETKTEENNEHQNEKRFVNAIVAGTIENSTTLQLPIWVSTYLPNSGFRDFHVSPFNPDQGFDSLEQANVDPLRRYQAGANPTVHTGEVGIDITNWLEGQVVSPPLVQSMTDADATLLDTRGELHTRRRSPTPTTDVRKNEGEAVEEENNNGINDEERRSIPTWEKATPVDEHTEIIVENLEADSHMDINKEQIVSNDAQLTRHNRLHYIYKKNKIERTADLLKIKTQKDDNNMNNARRGESQEPATDVQNIPPEHHPMILHEEPHEGAVEDPGSKHSKGTIPSVNRSTNRRKSQASSVPLLDTEGSVRIPCYVIIFLLGVIGNTLVIVTLLQNRKMRTITNVFLLNLAFSDLLLGVFCMPFTLVGSLLRDFIFGPVMCRLIPYFQAVSVSVSVWTLVAISLERYYAICQPLRSRGWQTLSHAYKIISLVWLLSLVFMAPIAALSQLLPVGDTSRHKCREMWPSLHLERGFSMFLGAGLLLVPLVIMVAAYSSITISLWHGMRLERETGQGTRPLISLTDLNGLAMVQQSLRRKHSRRVSKISIRFRKIPAQTRTCEHCCQPESVAIPEVVVHSHKSPTDLTHTPSSSQDGTHVCLHGRDCQNYKWHVESVRENGNLESPHRKEPKTGFKRLRSTHLEKSIEAKKHVIKMLFVVVLEFFLCWTPIFVVNIMCLYIPEQVYRVLGSFGISFMHLLSYASSCCNPITYCFMNKKFLQGFRHAFGCRKENERMGRLNGTAASFRSQSNNAFKLAMEIKKDTRETTV**

**W1. Tachykinin-like peptide receptor I (from DS01-Homarus1_Transcript_51762)**

**MGSVGEDNVSPTSESNATRRWLRKHLAHIMEASSGFTPSIEGKTHTLNPLRILATKTSRMVGGGLEDATPGSLVKDWLEGHSNQSTDLLTLLSMEGDAPDPHTLRRVLNNLLQVETTPYHNDDLENEASSDLPFQMPVYQQVLWVLAFGVMILGACIGNTIVIRIVLGNQRMRTTTNYFLVNLSVADLFMTLFNCIFNFVFMLKSHWAFGSIYCTISQFVAHASLAASVFTLAVISFDRFLNVMRPLKPRMSACVCRLLLCIIWVVAASIAAPILPYATTIQYDLGGRTICVILWPDGLASVSHMDYVYNVVILAVTYVVPMTAMVVCYAVIGCKLYDRRSAKTSRQADNFKSKRKVVQMLALLVGVFALCWLPYHVYFILVHHHPQLSTRPYVQHMYLAFYWLAMSNAMINPIVYYLLNARFRYFFRQLFAEMQGNLRSCVKTSDPSSSDFSADDDPFALHLQLIGDPGRCVGGRPPLLKHNNTAHSPLPLSPHHDHPVLP**

**W2. Tachykinin-like peptide receptor II (N-terminal partial protein from DS01-Homarus1_Transcript_23908)**

**MMMNTAERIYDDKSSQPHRDTSTTTTTIKPTAPDAEVLANYTQIYTNIVGCLVSESDSLNLTLGEGFWLPILYESLQLPYNVTTAAFSQPYTTTTVSYETYSFANGDDDQVRSTTSYWSGEQPGFPRDNDQLPLEVIREIWQRCFFRTPVMATHRPYLLVWWQQFLWTLVFGAMMAMAVGGNTLVMWIICAHRRMRTVTNYFLMNLSAADLFMSVLNCMFNFIYMLHSDWPFGAVYCTISNFMANVTISASVFTLMAISFDRYIAIVKPLEPRMSKTSARVFILVIWSSSMVLSLPCLLYSTTVSFTYKDDEVRRGCILRWPDGQTSSSQHEHIYNIVFFITTYLLPMLVMLVCYFLIGRELWGSRSIGELTDRQAASIRSKRRVVKMFIIIVTMFGLCWLPQQAFFLYTFHNSQILDTAHIQHIYLAFYWLAMANAMINPVIYYWMNARFKSCFKKVMLKRGITKQRERVREATTHSHDLSTGRFRSYIK+**

**W3. Tachykinin-like peptide receptor III (N-terminal partial protein from DS01-Homarus1_Transcript_26508)**

**MELLDYETTWNMSTIQVRDMAQNGTGQEYEDPTINLVYTMLNSTNSTGNVTLGEENKYQLPWWHQLIWSVFFGGMVVVATGGNLIVIWIVLADRRMRTVTNIFLVNLSVADAMVSTLNVVFNFTYMLNLHWRFGRIYCKISQFVSILSICASVFTLMAISFDRYIAIMHPLRPRMGRKATILIVVWIWVSSTCLSMPNMLFFTTAPLPLKDGDTRVVCYAQWPDGDQGYSQSEYVHTVVLMVLTYILPLLCMGFTYARIGMTLWGSRSIGEQTPRQVESIKSKRKVVKMMIVVVSIFAICWLPYHLYFILSNLMPEIAHTDYIQETYLAIYWLAMSNSMYNPMIYCWLNNXXXXXXXXXFRQGFKKVFSGWLPCVHYYEREPVEVTRVHTTRFSCSGSPETLHRIAYD+**
