# Supplementary material for: Neuropeptidergic Signaling in the American Lobster Homarus americanus: New Insights from High-Throughput Nucleotide Sequencing
Source: PLoS One. 2015 Dec 30;10(12):e0145964. doi: 10.1371/journal.pone.0145964 (PMC4696782; doi:10.1371/journal.pone.0145964)
Supplement: S5 Fig — (DOC) [file pone.0145964.s005.doc]

**A. Alignment of CCHamide receptor (CCHamideR) I and II**

**CCHamideR-I MEDLSSHQNSNMEAGSISNTTEKDQELSAGVVDVGPLTGGQTFLLD-PSITTRAYLWLST**

**CCHamideR-II MEDTAM--------------------LSPTL-----LFTNQTLDLDVERNTTHTNTLLDD**

***** : **. : * .**: ** **:: *.**

**CCHamideR-I TIKNESWWGPSDDYSLSLNLTEEYFTSVNVTNDGIYENDTNVTTGYVPYPQRPETYIVPM**

**CCHamideR-II TILDNTSW------------SFNYTNTSGGQDVGLSSNET-----YLPYRERPETYLVPI**

**** ::: * : :* .: . : *: .*:* *:** :*****:**:**

**CCHamideR-I LFAIIFIVGVIGNGALIVIFARNKTLRNVPNTYIISLALGDLLVLFFTVPFVSTIYTIES**

**CCHamideR-II VFALIFITGVVGNGALIFMFLKHPKLRSAPNTHLVSLAAGDLLMVLLTVPFTSIVYTVSS**

**:**:***.**:******.:* :: .**..***:::*** ****::::****.* :**:.***

**CCHamideR-I WPYGGFECKFSEFVRDISVGVTVFTLTALSADRYMAIVSPVKKAVGAARG-VTVRTAVSI**

**CCHamideR-II YPFGEAVCRASEFAKDLSLGITVFTLTALSADRYMAIVRPVTHHVSDSTGHVAIAVAIGI**

**:*:* *: ***.:*:*:*:***************** **.: *. : * *:: .*:.***

**CCHamideR-I WVVAVLLATPSAVFSSVRVFKVSNVKSISVCYPVPENFIDWYMPASILTKALIYYLLPLI**

**CCHamideR-II WVVAALLATPAAIFSNTPELVNPKGEKFHICTPYPEYLGAVYKQVHALVKAIMYYLLPLA**

******.*****:*:**.. : .: :.: :* * ** : * . *.**::********

**CCHamideR-I VIGTFYLLMARHLL--ASDVPGESHVFHKQIRTRRKVAKVVLCFVMIFAVCFLPTHVFLL**

**CCHamideR-II LIASFYVLMARHLFISAQFLPGEAAGQQRQAQARRKVAKMVLAFVTIFAICFLPLNVFNL**

**:*.:**:******: *. :***: ::* ::******:**.** ***:**** :** ***

**CCHamideR-I WFYFDPEGSNNYNDFWHALRIVGFCLGFINSCINPIALYCISGTFRKQYNRYLFCCCWGR**

**CCHamideR-II WWHFAPNSRETYDIYWHTFRIVGFCLSFINSCINPIALYCVSGTFRKYYNKHVFCWCTRH**

***::* *:. :.*: :**::*******.*************:****** **:::** * :**

**CCHamideR-I SGHRNINSLRSVRSSGSRYRCSTLRPSETITLTTL----------LHERTCAVSS-----**

**CCHamideR-II SGRRDWEGAESTGT-----RITTAVRTEQIPLKMVGTDNGTQQPPAHRLTLTNTTVLTTK**

****:*: :. .*. : * :* :* *.*. : *. * : ::**

**CCHamideR-I ---------**

**CCHamideR-II PHHNPASLV**

**B. Alignment of ecdysis-triggering hormone receptor (ETHR) I and II**

**ETHR-I ME---RVDYEDPFAEYRPQESLGITPGGPSFALLDATSQNPYPVSYPKSFLVSSYPPLLT**

**ETHR-II MEFGLDLVVSTPYLECGGGECGGVVP------------------------LPSLVPPAHN**

**** : . *: * *. *:.* * * ** .**

**ETHR-I TRADNITDLLYNGTDFGSETNTTTTATNMP--VFPEYIRVVSTVFCSVVLVVGVVGNVLV**

**ETHR-II TTSALLPWAPTTTTFSPAPLNGSTAQGALPNISFPAYMQGVYTAWCLVLLLVGLLGNVLV**

*** : :. . * : * :*: :* ** *:: * *.:* *:*:**::*******

**ETHR-I PVVILKDRDMRN-STNYFLMNLSVADLLVLLICLPPVLIELHSVQDLWVLGYTMCKLVPY**

**ETHR-II PLLVVRDRDLRGASTSVFIVNLVAADLLVLVVCLPALLSELYAPPAVWILPPSMCKVVPY**

***:::::***:*. **. *::** .******::***.:* **:: :*:* :***:*****

**ETHR-I VEMSVVHASALSLVVISLERYHVICQPLQAGYRCTKAKAVVAITIIWAISFISAGPLLMI**

**ETHR-II VEFTVAHASMLTILAISVERYRAICHPLTAAATCSRARAAVACFLVWVLATSVTSPVIAL**

****::*.*** *:::.**:***:.**:** *. *::*:*.** ::*.:: :.*:: :**

**ETHR-I VQYNIARFYDGTYKPQCIMPIHGNWIKSYFFATSILFFFLPLFLLVVLYTIIARQLLVDT**

**ETHR-II TEYTHVRYIDQSLVPVCYTRVDILWAKVFVVSSMVVLFFLPLLVLVVLYWRIARQLLLED**

**.:*. .*: * : * * :. * * :..:: :::*****::***** ******::**

**ETHR-I YELTHKKENPQMRARRQVVVMLATVVLFFFLCILPMRVLYFWIMTVPNETVTSLGIEGYY**

**ETHR-II KQLCKDKPNPNLQARKQVVVMLGTVVVVFFVCLLPHRVFSLWFIFTTKESEQSLGQEVYY**

**:* :.* **:::**:******.***:.**:*:** **: :*:: ..:*: *** * ****

**ETHR-I NLLYFCRIMYYINSSINPILYNMTSTKFRTAFRRVFRGKRGRLRRQHTYSNTSFNNPTVS**

**ETHR-II NLLYAFRILVYFNSAINPVLYNVTSSKFRGAFFRLVGVRRGEQVRW-SVQQTATNNSTVS**

****** **: *:**:***:***:**:*** ** *:. :**. * : .:*: **.*****

**ETHR-I NN-------------------LRLNY------GTNCSMVYKTLFSKTVVSNQYSYASTGS**

**ETHR-II NTTLTSSLNKSLPPWKSQRLLVRCSYTCLRGEGETCPLVDRRPVSPAVWGNTNHAHQPPS**

***. :* .* * .*.:* : .* :* .* .. ***

**ETHR-I NSSQVTRQTSLVSTNRSHVNTKDTFV**

**ETHR-II PATSPFLARS--SCTRDAVRQIESFV**

**::. * * .*. *. ::****

**C. Alignment of neuropeptide F receptor (NPFR) I, II, III and IV**

**NPFR-I M--------------------------DQGPDISVASQE-LPSDGFGSPEI---------**

**NPFR-II MFTLEWQTAG-----VGWKRLD-----GPPPSLNPSIMA-LDSLDFDS------------**

**NPFR-III ML--EESSHGGEGGVVGEK--DDGENVFEE---VSGVEV--------SCRDHGACGGFSP**

**NPFR-IV M---ENIS------------------LFSEPELCSGVMCPLQPDEYKALEGHVMSGTTIG**

*** . :**

**NPFR-I -----SSL-------DFLTLANFDNNLTNLAHNFSWLFNVSEGLNIDLINKFQRNRRVND**

**NPFR-II -----EDF-------NLMNLAALNENLTSLAHNLTHLFNLTGGINVDLLKKFEKNRRVGD**

**NPFR-III QEDLTNPFLPLEKLGKLLLEPSLPNNTNTSSHH--PLFNFSIHEAYDIISDT-QAGYLDG**

**NPFR-IV EED--NPF--TNQLESFFKSSVLPTGANQS---------LPWEEIMKILKETNQKSYLGT**

**. : .:: . : . . .. .::.. : :.**

**NPFR-I GAYYALIVVYSLLIVLGSTGNSLVVVAVIRKPAMRTARNVFIINLAISDLLLCLVTMPLT**

**NPFR-II GAFYTLIFAYSVLILLGATGNSLVVMAVIRKPAMRTARNVFIINLAISDLLLCLVTMPLT**

**NPFR-III VTEVVFIVCYVSLILFGVGGNMMVGWVIWRKRTMRTPRNLYIINLTVSDLSMCLVCMPVT**

**NPFR-IV AAKVSLITIYSLLITVGILGNLIVAFVIGHRPELRTARNVYIINLAVSDVSMCLVCMPFT**

**: :* * ** .* ** :* .: :: :**.**::****::**: :*** **.***

**NPFR-I LVELLSQYWPLGDHPFLCKLVGTLQATSIFVSTISITAIALDRYQVIVYPTKNSLKTVGA**

**NPFR-II LMELLSQYWPLGDTPFTCRLVGTLQATSIFVSTISITAIALDRYHVIVYPTKKSLQKVGA**

**NPFR-III LVGLLYKNWGMGS--LACKLVPVLQGANIMVSTSTVVAIAVDRYATIVKAGGSTRNKFHV**

**NPFR-IV LVGLLHKNWSLGN--FICKLVPVVQCTNILVSTATIVAIAADRYLTIVCVQRNRDARAYI**

***: ** : * :*. : *:** .:* :.*:*** ::.*** *** .** .**

**NPFR-I VLMLLLIWVISFILALPNFIWRTLKHHVINLPNLYSINFCFEEWPTEHGRGYYSVFVILV**

**NPFR-II VGSLLVVWLLSFLLALPNFIWRTLETHHVNLPGIEVVRFCFEDWPFEHGRAYYSVFVILV**

**NPFR-III AASICAIWVSSVLFALPLYFYYIVAQ--VKLQHILLYSRCVDHWPSRSAKNVWIIALLLT**

**NPFR-IV PWSVAAIWLVSLVFPLPLFAYYFVEK--VQIKDYLLYEKCVESWSSPVVKYTWNITLIVM**

**: :*: *.::.** : : : ::: *.: *. : : : :::**

**NPFR-I QYCLPIVTVSVAYAMICRKLKFRMANSTVRSSKKGERDDRRMKKTNKLLITIALIFCLSW**

**NPFR-II QYCLPILTVSIAYARICNKLKYRMTNASSRSA-RSRKEDLRMKKTNTLLVSISLIFCLSW**

**NPFR-III QYGIPIVVLSVVHARIKRYLSQHMMGQ--YDARRAQKEIERNRKTTILLSTIAVAFAVCW**

**NPFR-IV QYIIPILVLSFVHGRIQNYLSSHKMSQ--RDARRAQREIERNRRTTILLTSIAVTFAICW**

**** :**:.:*..:. * . *. : . .: :..:: * ::*. ** :*:: *.:.***

**NPFR-I LPLNLYNLVVDFHNPFGDDMETMLVVYAVCHMMGMSSACSNPLMYGWLNDNFRKEFLEIF**

**NPFR-II LPLNLYNVIVDLHNPFGEDTESMLIVYSVCHMAGMSSACSNPLLYGWLNDNFRKEFLEIF**

**NPFR-III LPWNIVNLLADFEYEGFKDPTHLYTVFGACHMIAMSSACINPVLYGWLNTNLRRELLEFL**

**NPFR-IV LPWHVVNLLADFNYAGFQEPEYFYVVFGSCHAVAMSSACINPILYGWLNTNLRKEMTQAI**

**** :: *::.*:. .: : *:. ** .***** **::***** *:*:*: : :**

**NPFR-I SRVLPCREP----------------QPQLQGSR-------RGQALVQEKTTPTPSKLPAT**

**NPFR-II GVVCPC-------------------CPVVTNAS-------RMNSLKTSRIGKEGGSLKSL**

**NPFR-III PPIFAKMGWILPESFRRRTGDSPTRQP--ESVTLLVFQGNQNNSIQTVAHPPQ-------**

**NPFR-IV PILLRKTSCPLRRSGNTGGGGVTGTNPTVESVSLLVHRPLRDDSCPGKDVPEEGGSKKN-**

**: * . : ::**

**NPFR-I PQGKDERPKVLYMKAKEAVEVNG-TCDTQGDE-TYITQVVTNATL**

**NPFR-II PLC--NHPVVLYTKAPEEQTCNGLSVDLQDQEVTFISQVVTTTTL**

**NPFR-III ------TITTTIIK----------------DD-T-----------**

**NPFR-IV ----HHQTKTPFLK-TESAKIGE-VTDQNHDE-T-----------**

**. * :: ***
