# Supplementary material for: Neuropeptidergic Signaling in the American Lobster Homarus americanus: New Insights from High-Throughput Nucleotide Sequencing
Source: PLoS One. 2015 Dec 30;10(12):e0145964. doi: 10.1371/journal.pone.0145964 (PMC4696782; doi:10.1371/journal.pone.0145964)
Supplement: S1 Table — (DOC) [file pone.0145964.s006.doc]

| Supplemental Table 1. *Homarus americanus* precursor-related and other peptides whose structures do not place them into any recognized family | |
| --- | --- |
| Family | Structure |
| ACP PRP | **SGGITGPLVTPGGGSDRGADPCKDVRLATLTQVASHLADLMDDTFDLPQDDAALALRLKHGLVA**  **MS** |
| AST-A PRP | **HDY_(SO3H)_LEDLDDPDTSRLLDVLQYYDTEPSYLYDYa**  **EGLYSLGLD**  **SVGDLPEVSKVEDGASPRT**  **DVSITEDTLED**  **ESSKN**  **DSGEE**  **EDDDMENRTQQYSFGLGKQDPDMEIE**  **ESDEDSD**  **DPDMDMD**  **ASSDEDDEERYYAYEQa**  **AFSETDDY_(SO3H)_DNVNDNDDGDDELELSDLEQY_(SO3H)_SDDL**  **SDAPDSGFa**  **SDSDSDQYTLa**  **EVSDDDHDEDEQDIGVEEEMSS** |
| AST-C PRP | **+LQAMMNHLHMNKQQQQQQQQQQQQQQQQQQQQQQQQQQQQQGEEEV**  **MFVPLSGLPGELPTI**  **KALPDQDPQVYGQMPHMLDPAGNHLIDDDGSLDAVLINYLFAKQMVERLRNNADIKDLQ** |
| CCHamide PRP | **DGDQYARQEPSPLYPEANQLPEFEQRQEDRLSVDEAVTDREIVANA**  **NWLAVLSHRLRQRTSPQSSPSAQSLGYFQ**  **AYVPVHPPVAPRPLLDVLLDALNTPTRSSHYSHARAANSVMGPRASYPEa**  **VKSPPTSDQLSDMGLDLRGEDYASGTNDDLESVGAIGGVRGSLDDTRDLAQDNVLYYGVLNDDY_(SO3H)_SDARY**  **SAVSLPSRGRLGASPPLGVANNAVPQDRPHIL**  **EEHTGKDEMDPKYLALASFPNWL** |
| Corazonin PRP | **SDPNVGVTELLADPP**  **LSAHSHPHPPTHTLPKNIEERLRALEAGLNAVLKANSINFSPGGDEEY_(SO3H)_YAEN** |
| CCAP PRP | **GPVA**  **DIGDLLEGKD**  **SDPSMEGLASSSELDALAKHVLAEAKLWEQLQSKMEMMRSYAS**  **MENHPVY**  **STPHTQPRQHLTSTPQQKVETEKQ** |
| DH31 PRP | **AAFNREA**  **AVVQIEDPDY_(SO3H)_VLELLTRLGHSII**  **ANELEXXXXA**  **SSDDGLDLHHDDNLYAQDQAADLAESS**  **ANELEKFVRSSGSA** |
| DH44 PRP | **1**  **NSNRSN**  **SNSSSGISGSNTSSNSNTNNNSPDTISMA**  **TWPNGFS**  **DVTRQLQQEGIQGVYQRGQ** |
| FLRFamide PRP | **APVPPVVAALDPPTDALLPAQSQEDDLFALPE**  **LLKYFLPASQAWGGDAYPIGQEGT**  **SDDNS**  **SDTNDY_(SO3H)_EGEEMPESPE**  **SGSPMEFATDLQEDVELPVEE**  **SVDRQLSSLSCEDCDEEQKAREFTSTPSPTTIQPLART**  **DVSAVLSDDSIESSVLRQINAHRI**  **AAAQNFYIPMAWASELQPEEDGIDVTSFEEPQVA**  **DGSDDY_(SO3H)_PSSSSSAESPAPVVVVRPVEYPRYV** |
| GSEFLamide PRP | **QYEPEFAHTLDYDT** |
| ILP PRP | **TY_(SO3H)_PTSEEEP**  **DGESEPGLPPEKYLDLLADPEEERGL**  **HHYLTSSQQASEDTPSEENEAPGSFFGSLSPQDLPHQSAVQEDEASSVHFPFLTEEEASQMVRVRPRS** |
| Intocin PRP | **SGPTAQLGRTRTCTACGPGLQGRCLGPEICCVLGIGCFLGTREA**  **MCHAENLVPVTCANRDLKSCa**  **MQEGRCAAAGLCCTEMKCEFDSSCTVEGREE**  **VGKQRAE**  **QHLTFLSSLPEDQWNL** |
| Leucokinin PRP | **LSSGAASVSFVTSEVMDVSPLALPHGRHPNLCTPDHVPSHPIIRCEVa**  **SSFKAAPGLPLSLREVYLALFQNARPRPPPPSEGEL**  **SDPLLPASQHEPNT**  **AAGYFTHDTNPLIIEEDLIPYIGVLSDDGEAEDVV**  **GSFPADDWEEEEPTDLFVLDGSLPYPPVDRLRY**  **EAETYTNTLVNSDDSGVKTEAENIKPDTKY_(SO3H)_DQTAEASSTTVA**  **PDLQVIEDAVRKMAEHEPKTTE**  **SSDVNLQDGEDDEPVSAWIa**  **LQDANTDD**  **SPSMDLSGNQD**  **SSGDELDDHFLD**  **AEGTLSRLSESTLKAALDENSPEDNVDNH**  **SESNE**  **+DE**  **SSETD**  **NNGGSDDPTHSNNPQQISSILQQLQHQGLEFLH**  **LPNNDWGN**  **ASPISEDSQLSDLYTSQL** |
| Myosuppressin PRP | **VCVGVGETMPPPICLSQQVPLSPFA**  **LCSALINISEFSRAMEEY_(SO3H)_LGAQAIERSMPVNEPEV**  **SQQ**  **VGETMPPPICLSQQVPLSPFA** |
| NPF PRP | **GNHGAQRTEELYDY_(SO3H)_** |
| Orcokinin PRP | **GDY_(SO3H)_DVYPE**  **VYGPRDIANLY**  **SAE**  **SSEDMDRLGFGFN**  **APARSSPQQDAAAGYTDGAPV**  **GPIKVRFLSAIFIPIAAPARSSPQQDAAAGYTDGAPV** |
| PDH PRP | **QELKYPEREVVAELAAQILRVIQGPWGPMAAGPH** |
| Proctolin PRP | **ADDTRLDEI**  **ELLREMLE**  **TAEGANSRISGSGYD**  **FMY**  **SVPEEGAAEMVQPALNLPQ** |
| Pyrokinin PRP | **+IFARCTTETLGLEDEWAGLPQASFAQYPPALDDTSEAQPLSLLYNMYPSVTSADTVPPKSQELQYNSQDTP**  **LYYSQRPa**  **SVDLYDDEDPE**  **QTPQHDNEPTDDNDDSTHRWWWPFVAV**  **SEFVFSSRPa** |
| SIFamide PRP | **AGADPREYTVFEPGKGLASVCQVAVEACAAWFPVQE** |
| Sulfakinin PRP | **VSAPARPSSLARVLAPVV**  **QRLEESHLPPALVEELVQDFEDPELLDFHDAAa**  **SLTHSDQHHHHDTTVN**  **APARPSSLARVLAPVV** |
| TRP PRP | **AGEGQDTPQDRE**  **DASTALDDNTAASEYSSLPDPYPLYGLRDNNLPMLFAVPWKT**  **SDEEVFSDATADNDLEILL**  **YYXXXXXXXXXXXXSSRSELPLDFWVCVA**  **LITVRTPT**  **YYDDDSDMDAYIQALTAVVDGQQQQ**  **AYYSENPDEEISMTGVD** |
| Other peptides | **DLPKVDTALK**  **KPKTEKK**  **AVLLPKKTEKK**  **EVEEPEAPAPPAK**  **LRVAPEEHPVLL**  **GPSGGFNGALAR** |
| **^1^LSLGGGRADTSSLLSLPHPQELSQDDLQPFLSRQGNTDSAGAPSSVADYTGYDKSEVLRGLEDPTSSSAYRLQEALSEAVAAAAAAAEGAEGVRD-GAAALSPTANEGVTLEDLVPYDPGYYLYPAFLNRGDEAMTGGSSGINSLRKV**  Peptides shown in red are novel discoveries for *H. americanus*, while those shown in blue are known *H. americanus* peptides that were rediscovered in this study; peptides shown in black are one known from *H. americanus* but not rediscovered here (additional peptide structures from, among others: [17-19,60-63]).  Peptide structures are presented in the order in which they appear in their respective precursor proteins (if present in more than one protein, the peptide is listed in the order it appears in the first precursor).  Abbreviations: ACP, adipokinetic-corazonin-like peptide; AST-A, allatostatin A; AST-C, allatostatin C; CCAP, crustacean cardioactive peptide; DH31, diuretic hormone 31; DH44, diuretic hormone 44; ILP, insulin-like peptide; NPF, neuropeptide F; PDH, pigment dispersing hormone; TRP, tachykinin-related peptide; PRP, precursor-related peptide.  In the peptide structures shown, “a” represents an carboxyl-terminal amide group, “Y_(SO3H)_” represents a sulfated tyrosine residue, and “C” represents a cysteine residue involved in a disulfide bond. A “+” at the amino-terminus of a sequence indicates that it is a partial peptide. “X”s in a sequence indicate uncalled amino acids.  The sulfation state of tyrosine residues and disulfide bridging between cysteine residues was predicted only for putative full-length peptides.  Disulfide bonding patterns in peptides with more than one bridge: first and second, third and fifth, and fourth and sixth cysteines bonded in the intocin linker/precursor-related peptide SGPTAQLGRTRTCTACGPGLQGRCLGPEICCVLGIGCFLGTREA; first and second, and fourth and fifth cysteines bonded in the intocin linker/precursor-related peptide MQEGRCAAAGLCCTEMKCEFDSSCTVEGREE. | |
