# Supplementary material for: Neuropeptidergic Signaling in the American Lobster Homarus americanus: New Insights from High-Throughput Nucleotide Sequencing
Source: PLoS One. 2015 Dec 30;10(12):e0145964. doi: 10.1371/journal.pone.0145964 (PMC4696782; doi:10.1371/journal.pone.0145964)
Supplement: S2 Table — (DOC) [file pone.0145964.s007.doc]

| Supplemental Table 2. Most similar *Drosophila melanogaster* protein to each *Homarus americanus* peptide receptor sequence | | | | | |
| --- | --- | --- | --- | --- | --- |
| *Homarus* receptor | Top FlyBase blastp hit | | | | |
|  | Protein name | Flybase accession No. | GenBank accession No. | BLAST score | E-value |
| ACPR | Adipokinetic hormone receptor, isoform C | FBpp0089220 | AAS64647 | 131.34 | 9.4e-31 |
| AST-AR | Allatostatin A receptor 1, isoform D | FBpp0305932 | AAG22404 | 327.79 | 1.4e-89 |
| AST-CR I | Allatostatin C receptor 2, isoform F | FBpp0312429 | - | 375.55 | 4.9e-104 |
| AST-CR II | Allatostatin C receptor 2, isoform F | FBpp0312429 | - | 394.43 | 9.4e-110 |
| AST-CR III | Allatostatin C receptor 2, isoform F | FBpp0312429 | - | 257.68 | 7.7e-69 |
| BursiconR I | Rickets | FBpp0080183 | AAF53367 | 535.03 | 1.1e-151 |
| BursiconR II | Rickets | FBpp0080183 | AAF53367 | 536.95 | 2.2e-152 |
| CCHamideR I | CCHamide-1 receptor | FBpp0086052 | AAF57819 | 372.86 | 3.6e-103 |
| CCHamideR II | CCHamide-1 receptor | FBpp0086052 | AAF57819 | 336.65 | 2.7e-92 |
| CorazoninR | Corazonin receptor, isoform B | FBpp0303102 | AGB94448 | 261.92 | 7.2e-70 |
| CCAPR | Crustacean cardioactive peptide receptor | FBpp0293006 | AAF56536 | 416.39 | 2.8e-116 |
| DH31R I | Diuretic hormone 31 receptor, isoform C | FBpp0303580 | AGB93483 | 204.53 | 3.0e-52 |
| DH31R II | Diuretic hormone 31 receptor, isoform C | FBpp0303580 | AGB93483 | 325.86 | 4.3e-89 |
| DH31R III | Diuretic hormone 31 receptor, isoform C | FBpp0303580 | AGB93483 | 294.66 | 9.9e-80 |
| DH44R I | Diuretic hormone 44 receptor 1 | FBpp0086614 | AAF58250 | 349.75 | 4.2e-96 |
| DH44R II | Diuretic hormone 44 receptor 2, isoform A | FBpp0087047 | AAF58501 | 189.89 | 5.1e-48 |
| ETHR I | ETHR, isoform C | FBpp0308331 | AHN57438 | 324.32 | 1.3e-88 |
| ETHR II | ETHR, isoform C | FBpp0308331 | AHN57438 | 290.81 | 1.9e-78 |
| ETHR III | ETHR, isoform C | FBpp0308331 | AHN57438 | 209.53 | 2.2e-54 |
| FLRFamideR | FMRFamide receptor, isoform B | FBpp0304395 | AGB94042 | 312.77 | 4.2e-85 |
| ILPR I | Insulin-like receptor, isoform D | FBpp0288671 | ACL83551 | 304.29 | 6.5e-82 |
| ILPR II | Insulin-like receptor, isoform D | FBpp0288671 | ACL83551 | 246.13 | 1.4e-64 |
| LeucokininR | Leucokinin receptor | FBpp0076853 | AAF50775 | 388.27 | 7.5e-108 |
| MyosuppressinR | Myosuppressin receptor 1, isoform B | FBpp0303220 | AGB94019 | 270.01 | 2.5e-72 |
| NPFR I | Neuropeptide F receptor, isoform A | FBpp0078239 | AAF51909 | 343.58 | 1.9e-94 |
| NPFR II | Neuropeptide F receptor, isoform D | FBpp0300637 | AFH06266 | 336.65 | 2.9e-92 |
| NPFR III | Neuropeptide F receptor, isoform A | FBpp0078239 | AAF51909 | 191.43 | 1.5e-48 |
| NPFR IV | Neuropeptide F receptor, isoform D | FBpp0300637 | AFH06266 | 188.35 | 1.2e-47 |
| PDHR I | Pigment-dispersing factor receptor, isoform D | FBpp0309084 | AHN59298 | 415.62 | 4.3e-116 |
| PDHR II | Pigment-dispersing factor receptor, isoform B | FBpp0300789 | AFH07215 | 385.96 | 3.5e-107 |
| ProctolinR I | Proctolin receptor, isoform E | FBpp0311480 | AHN59339 | 150.21 | 5.4e-36 |
| ProctolinR II | Proctolin receptor, isoform E | FBpp0311480 | AHN59339 | 141.35 | 1.6e-33 |
| PyrokininR | Pyrokinin 2 receptor 1 | FBpp0082213 | AAF54930 | 207.61 | 2.9e-53 |
| RPCHR I | Adipokinetic hormone receptor, isoform D | FBpp0304609 | AGB92685 | 275.79 | 3.4e-74 |
| RPCHR II | Adipokinetic hormone receptor, isoform C | FBpp0089220 | AAS64647 | 152.91 | 2.0e-37 |
| sNPFR | Short neuropeptide F receptor, isoform B | FBpp0304913 | AGB94779 | 223.02 | 4.3e-58 |
| SIFamideR | SIFamide receptor, isoform C | FBpp0291029 | ACZ94970 | 417.54 | 1.7e-116 |
| SulfakininR | Cholecystokinin-like receptor at 17D1 | FBpp0288959 | ABW09450 | 237.65 | 4.7e-62 |
| TRPR I | Tachykinin-like receptor at 86C, isoform B | FBpp0111701 | ABW08638 | 336.65 | 2.7e-92 |
| TRPR II | Tachykinin-like receptor at 86C, isoform B | FBpp0111701 | ABW08638 | 411.76 | 8.1e-115 |
| TRPR III | Tachykinin-like receptor at 99D, isoform B | FBpp0290870 | ACZ95066 | 413.30 | 1.8e-115 |
| Abbreviations: ACP, adipokinetic hormone-corazonin-like peptide; AST-A, allatostatin A; AST-B, allatostatin B; AST-C, allatostatin C; CCAP, crustacean cardioactive peptide; DH31, diuretic hormone 31; DH44, diuretic hormone 44; ETH, ecdysis-triggering hormone; ILP, insulin-like peptide; NPF, neuropeptide F; PDH, pigment dispersing hormone; RPCH, red pigment concentrating hormone; sNPF, short neuropeptide F; TRP, tachykinin-related peptide; R, receptor. | | | | | |
