# Supplementary material for: Neuropeptidergic Signaling in the American Lobster Homarus americanus: New Insights from High-Throughput Nucleotide Sequencing
Source: PLoS One. 2015 Dec 30;10(12):e0145964. doi: 10.1371/journal.pone.0145964 (PMC4696782; doi:10.1371/journal.pone.0145964)
Supplement: S3 Table — (DOC) [file pone.0145964.s008.doc]

| Supplemental Table 3. Most similar arthropod protein to each *Homarus americanus* peptide receptor sequence | | | | | |
| --- | --- | --- | --- | --- | --- |
| *Homarus* receptor | Top GenBank blastp hit | | | | |
|  | Protein name | Species | GenBank accession No. | BLAST score | E-value |
| ACPR | Adipokinetic hormone/corazonin-related peptide receptor variant C | *Rhodnius prolixus* | AKO62858 | 239 | 1e-73 |
| AST-AR | Allatostatin receptor | *Periplaneta americana* | AAK52473 | 390 | 3e-129 |
| AST-CR I | Allatostatin receptor 1 | *Neocaridina denticulata* | AIY69136 | 555 | 0.0 |
| AST-CR II | Allatostatin receptor 1 | *Neocaridina denticulata* | AIY69136 | 488 | 5e-170 |
| AST-CR III | Allatostatin receptor 1 | *Neocaridina denticulata* | AIY69136 | 297 | 6e-98 |
| BursiconR I | Lutropin-choriogonadotropic hormone receptor | *Melipona quadrifasciata* | KOX70070 | 686 | 0.0 |
| BursiconR II | Lutropin-choriogonadotropic hormone receptor-like isoform X2 | *Camponotus floridanus* | XP_011252745 | 655 | 0.0 |
| CCHamideR I | [Phe13]-bombesin receptor-like | *Acyrthosiphon pisum* | XP_008185421 | 384 | 5e-128 |
| CCHamideR II | Hypothetical protein L798_11307 | *Zootermopsis nevadensis* | KDR14578 | 389 | 6e-131 |
| CorazoninR | Gonadotropin-releasing hormone receptor | *Macrobrachium nipponense* | AHB33640 | 325 | 2e-105 |
| CCAPR | Crustacean cardioactive peptide receptor | *Cancer borealis* | AIT57587 | 565 | 0.0 |
| DH31R I | Calcitonin gene-related peptide type 1 receptor | *Zootermopsis nevadensis* | KDR16222 | 400 | 5e-127 |
| DH31R II | Calcitonin gene-related peptide type 1 receptor-like | *Limulus polyphemus* | XP_013789156 | 455 | 1e-155 |
| DH31R III | Calcitonin gene-related peptide type 1 receptor-like | *Limulus polyphemus* | XP_013789156 | 392 | 2e-131 |
| DH44R I | Class B secretin-like G-protein coupled receptor GPRdih1, putative | *Pediculus humanus corporis* | XP_002424517 | 376 | 8e-122 |
| DH44R II | Parathyroid hormone/parathyroid hormone-related peptide receptor | *Habropoda laboriosa* | KOC64449 | 399 | 2e-127 |
| ETHR I | Neuropeptide GPCR A6b | *Nilaparvata lugens* | BAO01056 | 354 | 2e-115 |
| ETHR II | Growth hormone secretagogue receptor type 1-like isoform X1 | *Athalia rosae* | XP_012257616 | 312 | 5e-99 |
| ETHR III | Growth hormone secretagogue receptor type 1-like | *Limulus polyphemus* | XP_013773953 | 229 | 3e-70 |
| FLRFamideR | FMRFamide receptor | *Tribolium castaneum* | NP_001280540 | 344 | 8e-112 |
| ILPR I | Insulin-like receptor | *Macrobrachium rosenbergii* | AKF17681 | 1052 | 0.0 |
| ILPR II | Insulin-like peptide receptor | *Zootermopsis nevadensis* | KDR10688 | 1036 | 0.0 |
| LeucokininR | Tachykinin-like peptides receptor 99D | *Limulus polyphemus* | XP_013780503 | 454 | 2e-155 |
| MyosuppressinR | Probable G-protein coupled receptor B0563.6 | *Harpegnathos saltator* | XP_011148909 | 345 | 1e-112 |
| NPFR I | Putative neuropeptide Y receptor | *Zootermopsis nevadensis* | KDR19960 | 470 | 7e-161 |
| NPFR II | Putative neuropeptide Y receptor | *Zootermopsis nevadensis* | KDR19960 | 463 | 5e-158 |
| NPFR III | Neuropeptide GPCR A38 | *Nilaparvata lugens* | BAO01088 | 315 | 8e-101 |
| NPFR IV | Neuropeptide GPCR A38 | *Nilaparvata lugens* | BAO01088 | 294 | 7e-93 |
| PDHR I | Neuropeptide GPCR B2 | *Nilaparvata lugens* | BAO01102 | 529 | 0.0 |
| PDHR II | AGAP003654-PA-like protein | *Anopheles sinensis* | KFB41328 | 459 | 7e-155 |
| ProctolinR I | FMRFamide receptor-like | *Limulus polyphemus* | XP_013772144 | 375 | 1e-119 |
| ProctolinR II | Sex peptide receptor-like | *Limulus polyphemus* | XP_013782861 | 409 | 4e-136 |
| PyrokininR | Neuromedin-U receptor 2-like | *Limulus polyphemus* | XP_013772711 | 318 | 5e-97 |
| RPCHR I | Gonadotropin-releasing hormone II receptor isoform X1 | *Orussus abietinus* | XP_012272105 | 364 | 8e-122 |
| RPCHR II | Adipokinetic hormone/corazonin-related peptide receptor variant A | *Rhodnius prolixus* | AKO62856 | 227 | 2e-71 |
| sNPFR | Neuropeptide GPCR A7 | *Nilaparvata lugens* | BAO01057 | 472 | 3e-161 |
| SIFamideR | Neuropeptide FF receptor 2 | *Tribolium castaneum* | XP_970225 | 435 | 2e-145 |
| SulfakininR | Perisulfakinin receptor | *Periplaneta americana* | AAX56942 | 389 | 2e-118 |
| TRPR I | Tachykinin-like peptides receptor 86C | *Lucilia cuprina* | KNC25564 | 338 | 1e-108 |
| TRPR II | Tachykinin-like peptides receptor 86C | *Athalia rosae* | XP_012260410 | 417 | 4e-140 |
| TRPR III | Hypothetical protein L798_01894 | *Zootermopsis nevadensis* | KDR21658 | 466 | 3e-161 |
| Abbreviations: ACP, adipokinetic hormone-corazonin-like peptide; AST-A, allatostatin A; AST-B, allatostatin B; AST-C, allatostatin C; CCAP, crustacean cardioactive peptide; DH31, diuretic hormone 31; DH44, diuretic hormone 44; ETH, ecdysis-triggering hormone; ILP, insulin-like peptide; NPF, neuropeptide F; PDH, pigment dispersing hormone; RPCH, red pigment concentrating hormone; sNPF, short neuropeptide F; TRP, tachykinin-related peptide; R, receptor. | | | | | |
